# Temperature-dependent oviposition and nymph performance reveal distinct thermal niches of coexisting planthoppers with similar thresholds for development

**DOI:** 10.1371/journal.pone.0235506

**Published:** 2020-06-30

**Authors:** Finbarr G. Horgan, Arriza Arida, Goli Ardestani, Maria Liberty P. Almazan

**Affiliations:** 1 EcoLaVerna Integral Restoration Ecology, Bridestown, Kildinan, Co. Cork, Ireland; 2 International Rice Research Institute, Metro Manila, Philippines; 3 Department of Veterinary and Animal Sciences, University of Massachusetts, Amherst, MA, United States of America; Guizhou University, CHINA

## Abstract

The brown planthopper (*Nilapavata lugens*: BPH) and whitebacked planthopper (*Sogatella furcifera*: WBPH) co-occur as the principal pests of rice in Asia. A review of previous studies suggests that the two species have similar temperature tolerances and similar temperature thresholds for development. However, the distribution and seasonality of WBPH suggest that its temperature optima for performance (survival, oviposition and growth) may be lower than for BPH. We compared adult longevity, oviposition, nymph survival and development success, as well as nymph biomass in both species across a gradient of constant temperatures from 15°C-40°C, at 5°C intervals. The most suitable temperatures for oviposition, nymph biomass and development success were 5–10°C lower for WBPH than for BPH. Furthermore, compared to BPH, WBPH demonstrated clear differences in oviposition on different rice subspecies and on rice at different growth stages at 25°C and 30°C, but not at other temperatures. The results suggest that aspects of herbivore performance within tolerable temperature ranges, which are not often included in temperature models, may be more useful than thermal tolerances or development thresholds in predicting the effects of global warming on pest damage to crops.

## Introduction

Global temperatures have increased by between 0.5 and 0.9°C since records began in the 1850s and are predicted to increase a further 1.0–2.1°C before 2100 [1,2]. Ectothermic species, such as insects and other arthropods, have already been affected by these changes [3–6]. Development times and temperature tolerances are widely used to describe insect responses to temperature and have become an important component for predictive models of insect distributions, voltinism, migration, and overwintering under global warming scenarios [7–9]. However, despite numerous studies conducted using climate chambers, relatively few have examined aspects of herbivore-plant interactions within tolerable temperature ranges. For example, many studies of herbivore temperature tolerances (high and low lethal temperatures) and temperature profiles (temperature-dependent development rates) have reared target herbivores on artificial diets or were conducted as short-term experiments without host plants [10–13]. Furthermore, although insect herbivores can develop over a range of temperatures and increase their development rates at higher constant temperatures (~25–34°C), they may also attain lower body weights, or display reduced fecundities under more rapid development or where their host plants are negatively affected by the same high temperatures [14,15]. Therefore, in insects the optimal temperatures for development may not always correspond with optima for other life history traits or ecosystem functions (e.g., egg quality, feeding efficiency, or dispersal capacity) [15–17].

Although some studies have also examined insect responses to temperature as they feed on their plant hosts as opposed to artificial diets, particularly in plant-sucking insects [14,18,19], few studies have examined the effects of developmental changes in the host plant (ontogeny) on insect fitness (survival × reproduction) across temperature gradients. These effects could be important in identifying realistic responses to temperature where host plant quality influences herbivore reaction norms [20]. As a further complication, different herbivore species from a single assemblage might respond differently to temperature gradients depending on preferences for host plants of a specific age or condition. Herbivores may also be affected by variability in the strength of host defenses across different temperatures [1,21,22]. Therefore, interactions between herbivores, plants and their ambient temperatures can be more influential in determining herbivore-herbivore and herbivore-plant interactions under a changing climate than are thermal tolerance limits or development rates and deserve increased research attention.

**Fig 1:**
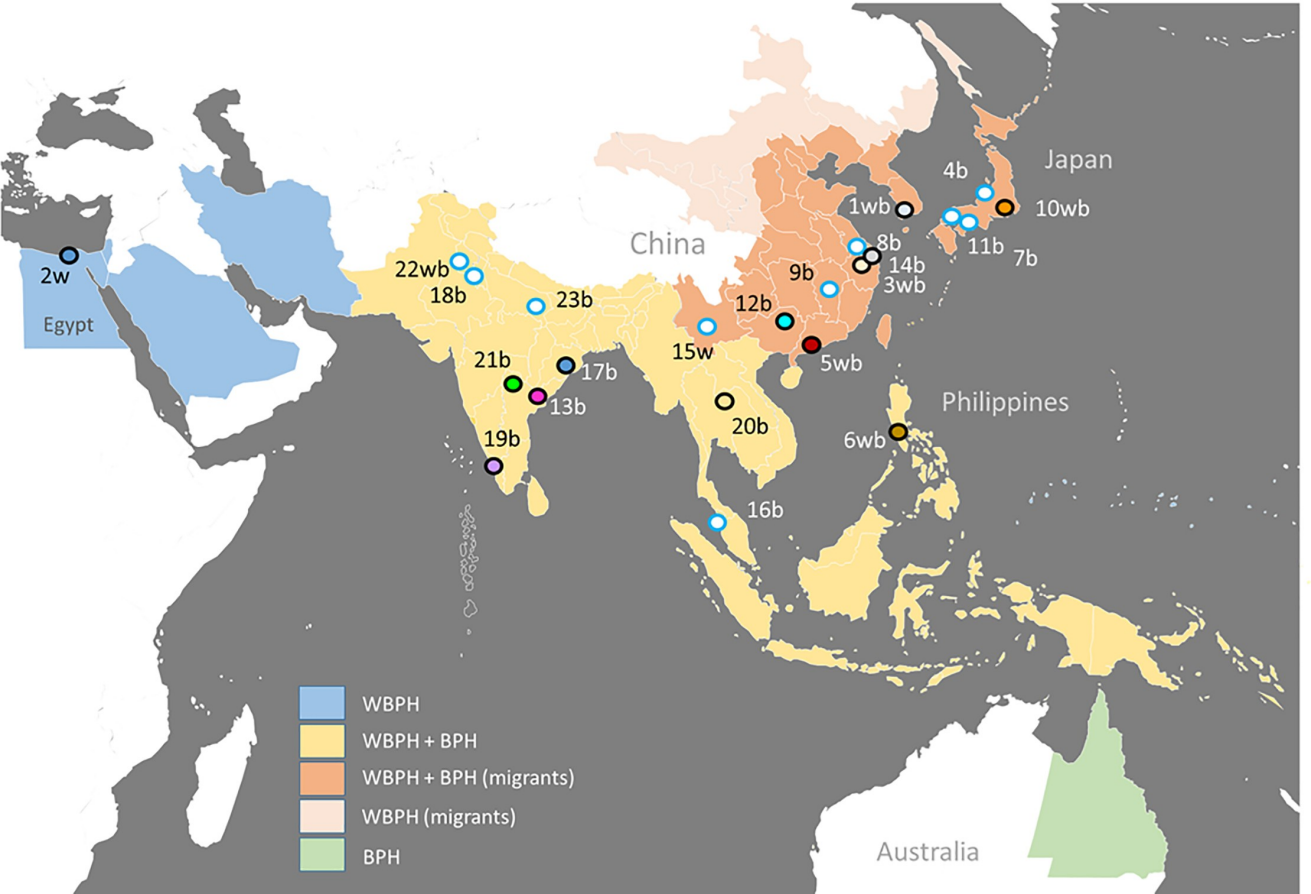
Approximate distribution of the whitebacked planthopper (*Sogatella furcifera*: WBPH) and brown planthopper (*Nilaparvata lugens*: BPH) around the Indian and Pacific Oceans. Distributions are based on national or provincial (Australia and China) records. The locations of population sources for planthopper colonies that have been used in climate and temperature studies are indicated. Open points with blue outlines were not used in our species level analyses (Section 2.3); colored points correspond with colored symbols used in the present paper. Numbers are regions where planthopper populations have been assessed for their responses to temperature. Some regions include multiple published studies. 1 = South Korea [35,36]; 2 = Kafr-el-Sheikh, Egypt [37]; 3 = Hangzhou, China [33,38–40]; 4 = Niigata, Japan [41]; 5 = Guangzhou, China [42]; 6 = Laguna, Philippines [43,44]; 7 = Kagawa, Japan; 8 = Jiangsu, China; 9 = Jiangxi, China; 10 = central Japan [34]; 11 = Hiroshima, Japan; 12 = Guangxi, China [45]; 13 = Godavari, India [46,47]; 14 = Shanghai, China; 15 = Yunnan, China; 16 = Pulau Pinang, Malaysia [10,11]; 17 = Odisha, India [48]; 18 = New Delhi and Haryana, India [19,49]; 19 = Tamil Nadu, India [50]; 20 = Khin Kaen, Thailand [51]; 21 = Rajendranagar, India [52,53]; 22 = Punjab, India [47,54]; 23 = Varanasi, India [55]. Sites are listed in chronological order of published studies; ‘b’ indicates studies with BPH, ‘w’ indicates studies with WBPH. The map was created using public domain information available through Natural Earth [56] and is not identical to any images from the provider.

Rice (*Oryza sativa*) is the staple food for over half the World’s population and is produced on more than 160 million hectares worldwide [23]. Climate models predict that, compared to other major crops such as wheat or maize, rice production will be less affected by climate change and yields may actually increase as a result of CO_2_ fertilization [24]. However, rice may be affected by increased levels of insect herbivory as the climate warms. For example, an increase in the abundance of planthoppers (Homoptera: Delphacidae) and leaffolders (Lepidoptera: Pyralidae) since the beginning of the millennium has been associated with increasing temperatures in Asia [25–27]. Furthermore, planthopper and leaffolder migrations in East Asia have occurred progressively earlier in recent decades and herbivore overwintering ranges in southern Asia have expanded poleward, and are predicted to expand even further in coming decades [28–30]. Among the most damaging pests of rice in Asia are the rice planthoppers (brown planthopper—BPH, *Nilaparvata lugens*; whitebacked planthopper—WBPH, *Sogatella furcifera*; and small brown planthopper—SBPH, *Laodelphax striatellus*) [31,32]. Because of their economic importance, a number of studies have assessed the temperature tolerances and temperature profiles of these planthopper species [10,11,33–35] (Fig 1). For example, the lower and upper temperature tolerances for adult female BPH have been estimated at 8–16°C and 36–41°C, respectively [10,11,33], and upper temperature tolerances of WBPH and SBPH were estimated as 37–41°C and ~40–41°C, respectively [33](Table 1).

**Table 1:** Key temperature extremes for WBPH and BPH based on published studies.

Parameter	Value estimates			
	WBPH		BPH	
	Minimum (°C)^a^	Maximum (°C)	Minimum (°C)^a^	Maximum (°C)
Eggs				
Effective temperature for egg development	12.6 [34]	na	8.1 [53]; 8.4 [46]; 12.3 [51]; 12.7 [34]	35.0 [51,53]
Nymphs				
Effective temperature for nymph development	11.2M, 11.2F [34]	na	8.1 [53]; 8.24 [46]; 9.4 [51]; 11.7M, 11.3F [34]; 12.5–17.6 [11]^b^	35.0 [51]; 34.9 [10]^c^; 34.2–37.2 [11]^b^
Coma temperature for nymphs	na	na	6.2–6.8 [11]^b^	37.7 [10]^c^; 37.6–41.0 [11]^b^
Lethal temperature for nymphs	na	na	0.5–3.6 [11]^b^	41.8 [10]^c^; 40.8–42.9 [11]^b^
Adults				
Effective temperature for adult male development	na	37.8 [33]^d^; 40.7 [33]^e^	8.8–16.4 [11]	38.0 [51]^f^; 37.7 [10]^c^; 36.6–37.7 [11]^b^; 37.8 [33]^d^; 40.6 [33]^e^
Coma temperature for adult males	na	41.8 [33]^d^; 42.6 [33]^e^	-0.3–3.5 [11]^b^	42.0 [10]; 40.6–43.2 [11]; 42.2 [33]^d^; 42.9 [33]^e^
Lethal temperature for adult males	na	39.0 [33]^d^	na	40.5 [33]^d^
Effective temperature for adult female development	na	37.8 [33]^d^; 40.8 [33]^e^	8.1–16.4 [11]^b^	37.0 [10]^c^; 36.0–37.3 [11]^b^ 38.5 [33]^d^; 40.8 [33]^e^
Coma temperature for adult females	na	41.9 [33]^d^; 42.7 [33]^e^	-1.2–2.9 [11]^b^	43.4 [10]^c^; 41.6–45.0 [11]^b^; 43.3 [33]^d^; 43.1 [33]^e^
Lethal temperature for adult females	na	41.2 [33]^d^	-2.7–2.1 [11]^b^	42.5 [10]^c^; 42.1–43.6 [11]^b^; 41.6 [33]^d^

^a^ M = male, F = female

^b^ Values range between cold acclimated and heat acclimated colonies

^c^ Values for colonies maintained at 23°C

^d^ For tests conducted without host plant

^e^ For tests conducted with host plant

^f^ Sexes not differentiated

BPH and WBPH are frequently the most abundant arthropod species in rice fields of South and Southeast Asia [57]. Studies of the development rates of these planthoppers across temperature gradients have generally indicated linear increases in development until reaching an optimum temperature (usually between 25–30°C), followed by a rapid decline in survival [53]. Furthermore, several researchers have assessed aspects of planthopper behavior and population development under temperature gradients, including studies of planthopper mating behavior, planthopper feeding responses, and planthopper population growth [49]. Despite these studies, there is still little knowledge of the potential interactions between planthoppers and their host plants under temperature gradients, including knowledge of oviposition, biomass accumulation and damage from planthoppers grown on different hosts (species or varieties [21,22]) or on plants at different growth stages. Furthermore, compared to BPH, few studies have assessed responses by WBPH to temperature gradients or to any other changes in global climate (Fig 1). WBPH has gained prominence in Asia over the last several decades due to high levels of adoption by Asian farmers of hybrid rice varieties, particularly those with a cytoplasmic male sterile lineage, that are highly susceptible to the planthopper [57]. However, it is also possible that gradual changes in global temperatures or other changes in regional climate might have contributed to the increasing prominence of WBPH in rice herbivore assemblages.

BPH and WBPH display strong oviposition preferences for rice at different stages of crop growth. In studies of oviposition, WBPH performed best on younger rice plants, with oviposition declining rapidly as plants developed beyond ~30 days after seeding (DAS). In contrast, although BPH also performs best on young rice seedlings, the species will continue to lay eggs as rice plants develop and grow [58]. There are also indications that WBPH may be more prevalent on varieties from the *O*. *sativa japonica* subspecies than from the *O*. *sativa indica* subspecies (henceforth *japonica* and *indica* rice, respectively) as damage by WBPH to *japonica* varieties is often more severe than damage to *indica* varieties [59] (but see [60]) and because WBPH lay more eggs on susceptible *japonica* than on susceptible *indica* lines [58]. The two species also differ in their distribution ranges. Compared to migrant BPH, migrant WBPH appear to disperse earlier in the spring [30] and distribute more widely in north temperate regions (Fig 1) where they also feed on wheat (*Triticum aestivum*), maize (*Zea mays*) and other grasses [61]. However, in comparative studies, BPH and WBPH display similar lower and upper temperature thresholds for development [33,34]. These observations suggest that BPH performs better than WBPH under warmer climates despite reports of similar thermal tolerances for the two species. Continuing gaps in knowledge of temperature profiles and planthopper response norms, particularly for WBPH, therefore hinder assessment of the relative risks from these planthoppers to crops under current and future climates.

The present study compares the responses by BPH and WBPH to ambient temperatures. We describe oviposition by BPH and WBPH on *japonica* and *indica* rice subspecies and at two stages of plant growth (20 and 30 DAS) across a gradient of temperatures. We also compare the performance of nymphs of both planthopper species across the same gradient of temperatures. Because nymphs demonstrated no apparent differences in performance on *indica* and *japonica* varieties in a previous study [58], we assess nymphs of both species only on *indica* rice. However, we focus on aspects of nymph-plant interactions that might reflect the functions (i.e., survival, weight gain and development to adult) of each planthopper species in rice production systems. Finally, we conduct a comprehensive review of previously published studies that examined the reactions of these two species to two or more ambient temperatures. We compared our results with results from these previous studies to identify general patterns in planthopper responses to temperature, to highlight continuing gaps in knowledge of temperature effects on the species’ life histories, and to develop predictions about the two species and their potential interactions under continuing changes in the global climate. We discuss our results in light of the importance of temperature tolerances and thresholds relative to defined behavioural responses to temperature as tools for predicting future pest assemblages.

## Materials and methods

### Literature review

We conducted a review of literature on the relations between temperature and the life-histories, survival, fecundity, and other aspects of the biology of BPH and WBPH. We conducted searches in Google Scholar for all papers until December 2019 using the keywords ‘temperature’, ‘climate’, ‘*Nilaparvata*’, ‘*Sogatella*’, and ‘planthopper’. We then screened the methods used in each paper to identify comparative studies that assessed planthopper performance at two or more temperatures. We also noted aspects of the study methods such as the origin of planthopper populations, the numbers of replications performed, the host plants and plant development stages used in the experiments, and other independent factors included in the studies (i.e., elevated or ambient CO_2_ concentrations, viliferous or non-viliferous planthoppers, levels of nitrogenous fertilizers, and ambient humidity, among others). For the purpose of our study, where factors other than temperature included two or more levels, we included only standardized or control treatments (e.g., ambient CO_2_, non-viliferous planthoppers, no or low nitrogen added). We retrieved a total of 72 papers, of which 35 included experiments conducted under controlled environments (in climate or environmental chambers). Finally, only 22 papers were included in an analysis of temperature effects on BPH and WBPH because these papers used controlled temperatures, presented sufficient details of experiments (e.g., mean temperatures, ambient humidity, duration of experiments, or numbers of insects used in experiments), avoided or explained confounding effects in their experiments, or presented new information not previously published. We were also unable to access some papers published in regional journals from China and South Korea.

### Herbivores

We used BPH and WBPH from colonies maintained at the International Rice Research Institute (IRRI). The colonies were initiated in 2009 (three years before the initiation of the present study) with > 500 wild-caught individuals of each species collected from Laguna Province (Philippines: 14°10′N, 121°13′E). We used the laboratory colonies because they were free of associated plant viruses and had largely synchronized development stages. The BPH colony had noted virulence against a range of resistance genes including *BPH1*, *BPH2*, *BPH5*, *BPH7*, *BPH8*, *BPH18*, *BPH25* and *BPH26* and displayed a high level of brachyptery [62]. The WBPH colony had noted virulence against *Wbph2*, *Wbph3*, *wbph4*, *Wbph6*, *WbphAR*, *WbphM1* and *WbphM2* [62]. The planthoppers were reared continuously on the susceptible variety TN1 (≥ 30-day old rice plants) in wire mesh cages (91.5 × 56.5 × 56.5 cm; H × L × W). The colonies were kept under greenhouse conditions (26–37°C, 12:12 day:night [D:N]) with feeding plants replaced every 3–5 days.

### Host plants

We used two rice varieties in our experiments. IR22 is a relatively modern (1969) *indica* rice variety that is susceptible to BPH and WBPH populations from South and Southeast Asia (moderately susceptible to populations from Bangladesh and Indonesia) [62,63]. T65 is a *japonica* variety that was first released in Taiwan in 1923. The variety is highly susceptible to BPH and WBPH from South and Southeast Asia and is closely related to TN1, the variety on which greenhouse colonies were maintained (see above)[62]. WBPH females lay significantly more eggs on T65 than on IR22, particularly under moderate nitrogen levels [58]. Seeds of the two varieties were acquired through the IRRI Germplasm Collection. The seeds were germinated in a greenhouse at staggered intervals (i.e., T, T+10 days) and planted at 5–6 days after sowing (DAS) (seedling stage S3, where the prophyll had emerged from the coleoptile [64]) to #0 pots (7 × 11 cm: H × D) filled with paddy soil. This produced seedlings of 20 and 30 DAS for use in bioassays at the same time. Sufficient numbers of plants were maintained to assess daily egg-laying and to replace plants exposed to nymphs when they showed symptoms of feeding damage (i.e., moderate yellowing) (see below). The pots and developing plants were placed in climate chambers at the same temperatures as those used in the final bioassays (see below) ten days before initial infestations to allow the plants to acclimatize.

### Temperature bioassays

Bioassays were conducted IRRI using environmental chambers with the Conviron CMP6050 Control System (Conviron, Winnipeg, Canada). To avoid pseudoreplication and control for errors due to spatial and temporal variability within climate chambers [65–67], the temperature treatments were rotated between four separate chambers and the temperature settings changed after each experimental run such that each chamber was used to replicate each of the test temperatures. Furthermore, each replicate (i.e., Run) included between three and five subsamples (i.e., rearing cages–see below) per variety and time treatment, with subsamples randomized within chambers. Temperatures ranged from 15 to 40°C, representing a low temperature at which nymphs can survive and develop [34,53] and a temperature at close to the upper lethal limits for survival of adult planthoppers [33], respectively. The bioassays were conducted as follows:

#### Oviposition experiments

Plants of each variety and age were individually covered with acetate rearing cages (50 × 10 cm: H × D). The cages had a mesh top to allow air circulation. A single mated gravid female was introduced to each cage at 20 or 30 DAS (i.e., plant age treatment) using a suction aspirator. All females used in the experiments were brachypterous. Temperatures were set at 15, 20, 25, 30, 35 and 40°C with relative humidity maintained at 80–85% and with a 12h:12h day:night light regime. Temperatures were replicated across the chambers (i.e., N = 4). Each replicate consisted of continual observations from one day to 20 days after caging the females. The plants under each acetate cage were changed daily and the condition of the adults noted (i.e., surviving or dead). Plants that were exposed to females were dissected to count the numbers of egg clusters and the numbers of eggs per cluster. The plants were cut above the soil and were dried in a forced draught oven at 60°C after which they were weighed. Replicates usually took several months to complete before any temperature was repeated in a new chamber. We examined the effects on female survival of manipulating the adults each day. Changing the host plant each day, as was conducted in each of our treatments, had a greater effect on BPH than on WBPH, with the greatest reduction in adult survival occurring at 30°C (S1 Fig). Because these observations were limited to one type of host plant (T65 at 20 DAS), we did not correct for the ‘manipulation effect’ in our analysis but applied the observation to the interpretation of results.

#### Nymph survival and development

Sufficient rice seedlings were prepared such that nymph development could be assessed daily for 15 days through destructive sampling for each variety during each temperature replicate (i.e., 15 days × 5 subsamples = 75 per variety per replicate). A number of replicates at 15 and 20°C were continued beyond 15 days to assess the time for nymphs to develop to adults. Temperatures (15, 20, 25, 30, 35, and 40°C) were each replicated four times as described above. Ten newly emerged nymphs were placed on rice plants of each variety and plant age and under each temperature treatment. Individual plants were covered with acetate rearing cages (50 × 10 cm: H × D) with mesh windows for ventilation. Nymphs were allowed to feed and develop for 15 days with plants arbitrarily selected for sampling across each temperature, each day. All plants were monitored for signs of yellowing due to nymph feeding. When the first and second leaves of the plants showed partial yellowing, the plants were replaced by fresh plants of the same variety and age. The number of survivors and their developmental stages were recorded and the insects were dried in an oven for five days and weighed to estimate total nymph biomass per plant. Development stages were recorded based on the examination of individuals from each cage under a binocular stereoscope with 10× magnification. The wing forms of emerged adults were also recorded. The plants were cut above the soil level, dried at 60°C in a forced draught oven and weighed. Each run usually took several months to complete.

### Data analyses

Results from the oviposition and nymph survival experiments were analyzed using repeated measures general linear models (GLM) with days after first exposure as the repeated measure and temperature, variety, plant stage, and their interactions as main factors. We conducted the analyses with planthopper species included as a main factor, and for each species separately. The results of analyses with species included are presented in S1, S2, S3 and S4 Tables. In the main text, we highlight the results of GLMs for each species alone because of species-specific differences in oviposition and survival rates, as well as large differences in body weights between the two species. Because each run took several months to complete, we included run as a blocking factor in each analysis to control for possible changes in the planthopper colonies over the course of the experiment (e.g., short-term acclimation to variable temperatures in the greenhouse). We did not include results from chambers at 40°C in repeated measures GLMs because of low survival of adults and nymphs at that temperature. Adults were alive across all other treatments and replicates for only ten days; therefore survival, the numbers of egg batches, and the numbers of eggs laid were analyzed up until ten days in the repeated measures analyses. In our analyses, we assessed female longevity as survival over time (units = %) in repeated measures GLMs but as maximum time before 0% survival (units = days) in univariate GLMs (see below). Nymph survival and nymph biomass were analyzed across replicates and treatments for 15 days (i.e., repeated measures GLM). Data for total batches and eggs were ranked, nymph and adult survival was arcsine-transformed and nymph biomass was log-transformed before analyses. Tukey post-hoc tests were performed for all significant temperature effects. Residuals were plotted following all parametric analyses and were normal and homogenous.

The maximum adult female longevity, and the total number of batches and eggs laid by the end of each experiment (including all 20 days), as well as nymph survival and maximum nymph biomass at the end of 15 days were further analyzed using univariate GLMs. Results from chambers maintained at 40°C were included in the analyses. For the analyses, maximum longevity was measured as the time in days to 0% survival. Nymph biomass was taken as the highest biomass attained over the 15 days (because nymphs tended to lose weight as they developed to adults at 25 and 30°C). The numbers of batches and eggs were log-transformed before analyses. Nymph development was analyzed as the time for 50% of nymphs to reach the second (N1), third (N2) and fourth instars (N3). The experiment was not sufficiently long to include development to fifth instars (N4) or adults (N5) for bioassays conducted at 15, 20 or 35°C (because < 50% of individuals reached these development stages at these temperatures). Nymph development times (N1, N2 and N3) were analyzed using multivariate GLM. The proportions of nymphs developing to adults at 25 and 30°C were analyzed using univariate GLMs. Runs were included in each analysis as a blocking factor, as explained above. Tukey post-hoc tests were performed for all significant temperature effects. Residuals were plotted following all parametric analyses and were normal and homogenous.

We assessed temperature-dependent development and life-history parameters of WBPH and BPH using published data combined with our own results. Across published studies, the values for different life-history parameters (i.e., survival rates, fecundities, etc.) often varied considerably. For example, Park and Hyun [35] reported > 600 eggs laid per BPH female, whereas Srinivas et al [52] reported ~130 eggs/female at optimal temperatures. Such differences may be related to aspects of colony maintenance (e.g., inbreeding, acclimation) or to the different conditions (e.g., relative humidity, light intensity) or rice varieties used in the experiments. To compare trends in BPH longevity, fecundity and hatchability, we therefore standardized values at different temperatures as a proportion of the highest values. Too few studies have examined adult longevity or fecundities in WBPH at different temperatures to make meaningful comparisons. We plotted data for each trait against temperature and used SigmaPlot (v. 13.0) to identify models that best fit the global data sets based on R^2^ and the highest associated F-values while fulfilling requirements for normality (Shapiro-Wilks test) and constant variance.

A number of studies have reported egg, nymph and adult development times for either BPH or WBPH, or have compared both. For egg development this included six published studies for BPH and three for WBPH. For nymph development this included nine published studies for BPH and four for WBPH. Stages in adult development have been divided into pre-oviposition period, post-reproductive period, age at first oviposition, or age at last oviposition in some studies; however, sufficient data was only available to examine pre-oviposition periods. For adult development this included five published studies for BPH and two for WBPH.

Most previous studies did not replicate their bioassays across temperatures (i.e., they used only one temperature chamber per test temperature, or sometimes used the same chamber in successive tests to assess development at different temperatures). We therefore combined the studies as ‘true’ replicates for each planthopper species and included the mean values from our own study in the analyses. This allowed some measure of variability for each temperature. We plotted the data against temperature and used the Thermal Summation Model (TSM) of Campbell et al. [68] to describe the relationships between development times and temperature. The TSM uses the reciprocal of development duration at each temperature as a measure of development rate. The linear model provides an estimate of the lower temperature threshold for development (T_min_) as the x-intercept. The model’s upper temperature limits were set as the temperatures before which development rates decline (i.e., where the relationship becomes non-linear). This is strongly affected by the intervals between test temperatures; however, in our analyses, because of the number of studies and temperature points, intervals were relatively narrow. Thermal constants (i.e., degree days required for development: k) were estimated as reciprocals of the fitted regression lines for each developmental stage [68].

## Results

### Effects of temperature on BPH longevity and oviposition

**Fig 2:**
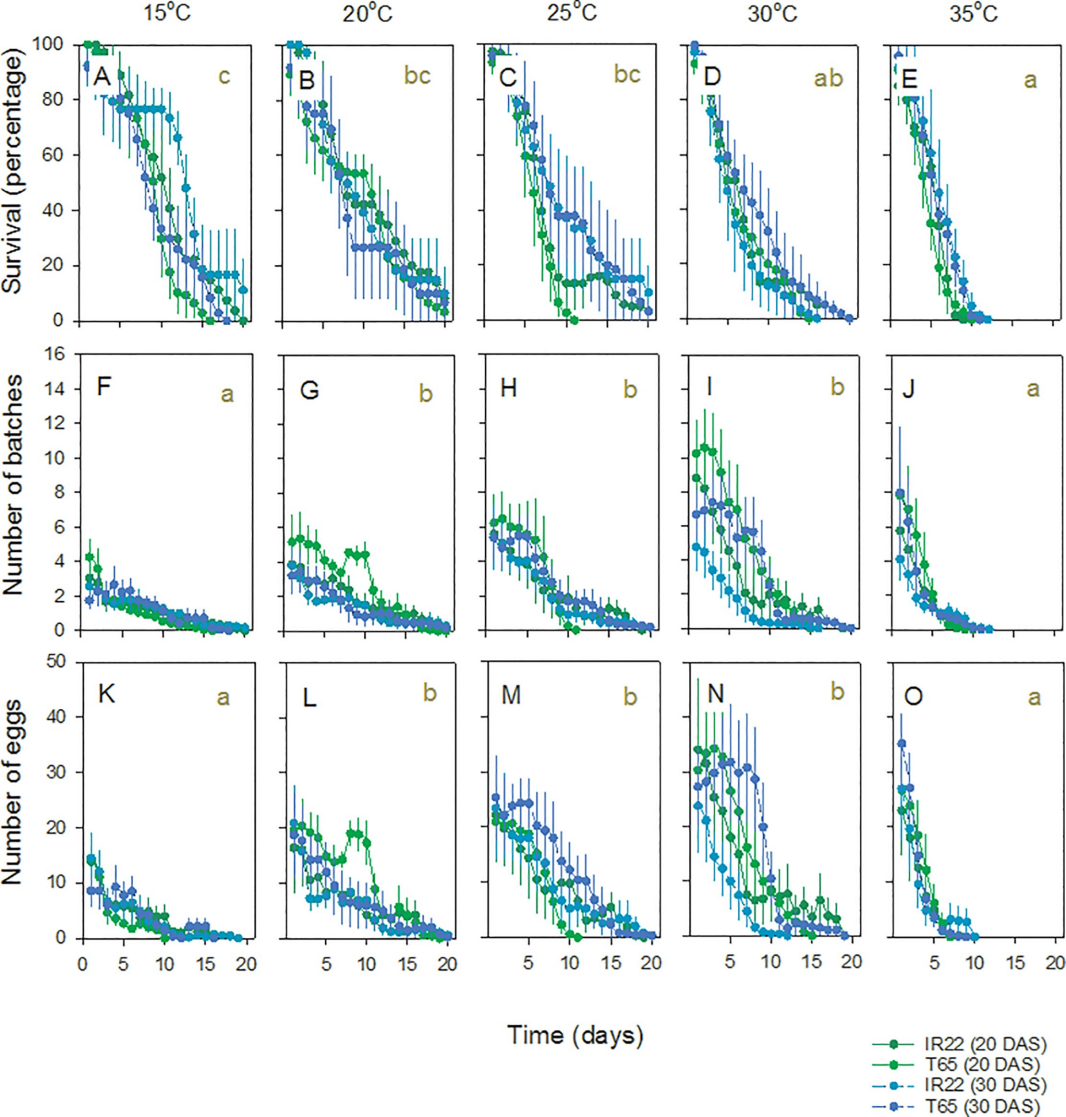
Effects of temperature on adult survival and egg-laying by BPH on *indica* (IR22) and *japonica* (T65) rice varieties. Infestations were initiated at 20 and 30 DAS; younger plants are indicated by green symbols and lines. Bioassays were conducted at 15°C (A,F,K), 20°C (B,G,L), 25°C (C,H,M), 30°C (D,I,N) and 35°C (E,J,O). Bioassays conducted at 40°C are not shown (see text). Results for adult survival during the experiment (A-E), the total numbers of batches produced (F-J), and the total number of eggs deposited (K-O) are presented. Standard errors are indicated (N = 4). Lowercase letters indicate homogenous temperature groups for each parameter (Tukey: P ≥ 0.05) based on repeated measure GLM. See also Table 2.

BPH adult survival and egg-laying declined over the course of the oviposition experiment with rates determined by temperature (Fig 2). Females survived longest at temperatures of 15–25°C (Fig 2A–2E). BPH produced more egg clusters (Fig 2F–2J) and more eggs (Fig 2K–2O) at between 20 and 30°C, compared to other temperatures (Table 2). There was no effect of variety or plant age on BPH survival (Fig 2A–2E)(Table 2). Over the course of the experiment, BPH tended to produce more egg batches and lay more eggs on T65 (Fig 2F–2O)(Table 2). Significant interactions between Time and Temperature (Table 2) were due to similar survival rates early in the experiment and similar numbers of eggs laid toward the end of the period analyzed (i.e., 10 days)(Fig 2). Significant three-way interactions between Time, Variety and either Temperature or Plant age (Table 2), were due to similar levels of female survival on both varieties and at both plant ages only at 30 and 35°C (Fig 2).

**Table 2:** Results from repeated measures GLMs of adult female survival and oviposition parameters (see Figs 2 and 4).

Sources of variation	DF	F-values^a^					
		BPH			WBPH		
		Adult survival (%)	Batches produced	Eggs laid	Adult survival (%)	Batches produced	Eggs laid
*Within subject effects*							
Time	9	209.485***	66.884***	65.050***	280.677***	65.875***	55.391***
Time*Variety	9	0.664ns	0.896ns	1.229ns	1.904*	1.536ns	1.405ns
Time*Plant age	9	1.259ns	1.353ns	0.713ns	3.440***	0.283ns	1.263ns
Time*Temperature	36	4.924***	5.062ns	4.802***	9.609***	5.226***	2.981***
Time*Run	27	5.697***	5.288ns	5.167***	2.714****	2.322***	1.495*
Time*Variety*Plant age	9	3.433***	0.605ns	0.728ns	1.563ns	1.582ns	1.581ns
Time*Variety*Temperature	36	1.511*	0.992ns	1.405ns	0.862ns	0.724ns	1.154ns
Time*Plant age*Temperature	36	1.338ns	0.944ns	1.076ns	1.075ns	1.392ns	1.240ns
Time*Variety*Plant age*Temperature	36	0.546ns	1.020ns	1.236ns	1.521*	0.718ns	0.985ns
Error	513						
*Between subject effects*							
Variety	1	0.944ns	8.920***	6.443**	5.442*	5.932**	8.011**
Plant age	1	1.587ns	1.665ns	0.004ns	6.966**	31.557***	34.902***
Temperature	4	9.474***	11.768***	12.898***	43.305***	3.725**	3.767**
Run	3	6.237***	6.532***	6.646***	4.065**	3.602**	4.254**
Variety*Plant age	1	0.279ns	0.001ns	0.415ns	3.211ns	0.790ns	2.185ns
Variety*Temperature	4	0.588ns	2.190ns	2.129ns	0.663ns	0.474ns	0.465ns
Plant age*Temperature	4	0.712ns	1.707ns	1.321ns	2.905*	7.238***	3.746**
Variety*Plant age*Temperature	4	0.438ns	1.354ns	1.526ns	0.159ns	0.038ns	0.172ns
Error	57						

^a^ ns = P > 0.05

* = P ≤ 0.05

** = P ≤ 0.01

*** = P ≤ 0.001

**Fig 3:**
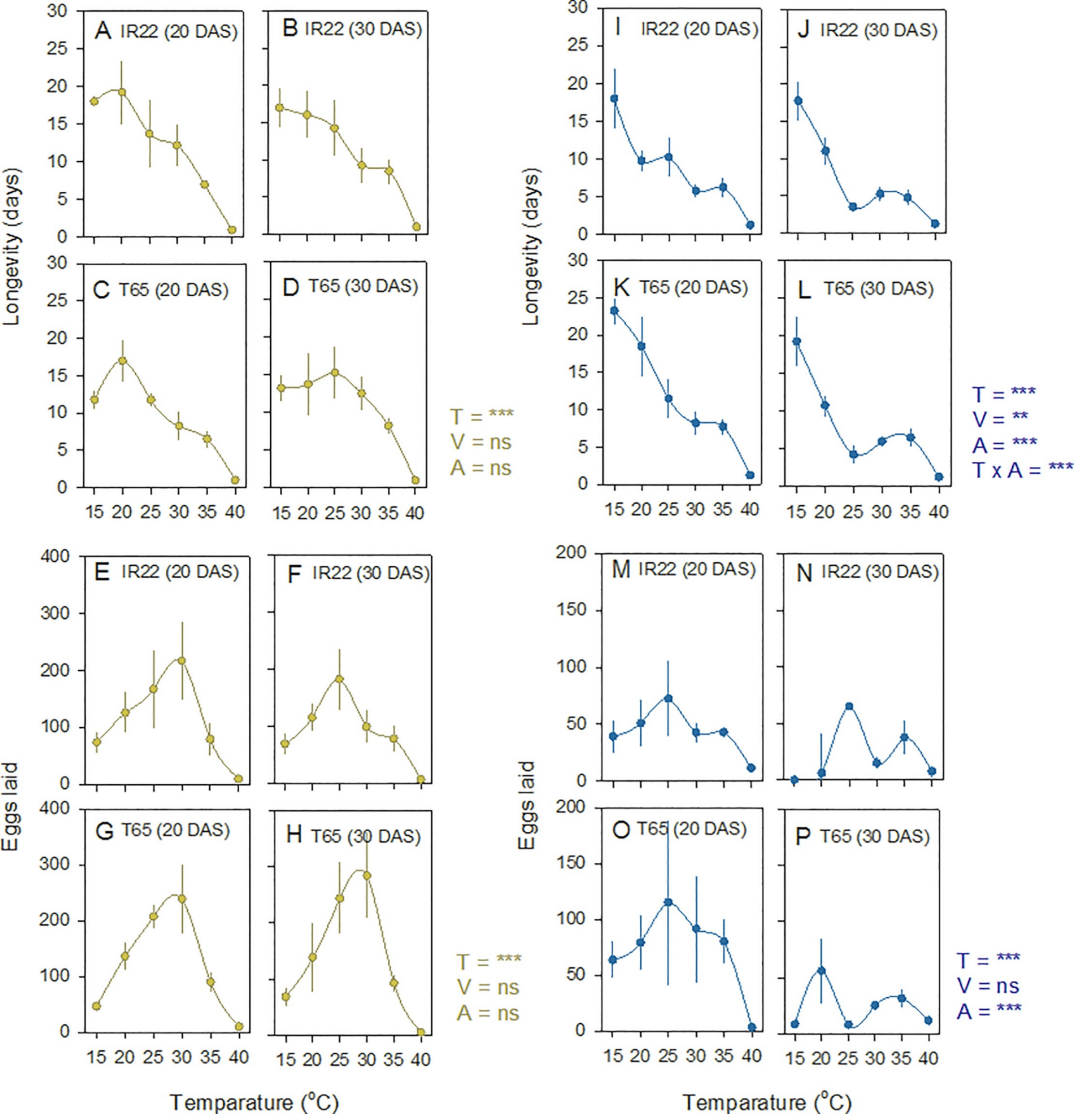
**Adult female longevity and egg-laying by BPH (brown symbols: A-H) and WBPH (blue symbols: I-P).** Planthoppers were maintained on *indica* (IR22: A,B,E,F,I,J,M,N) and *japonica* (T65: C,D,G,H,K,L,O,P) rice varieties initially infested at 20 DAS (A,C,E,G,I,K,M,O) and 30 DAS (B,D,F,H,J,L,N,P) across a gradient of temperatures. The times to 0% survival of adult females are indicated in A-D and I-L. The total numbers of eggs laid are indicated in E-H and M-P. Numbers are based on accumulated data over 20 days of the experiments. Bars indicate standard errors (N = 4). The effects of temperature (T), variety (V), and plant age (A) and significant interactions are indicated as ns (P > 0.05), ** (P ≤ 0.01), and *** (P ≤ 0.001). See also Table 3.

At the end of 20 days, BPH female longevity (time in days to 0% survival) was greatest at 20°C (Fig 3A–3D, Table 3); however the number of batches produced and egg-laying peaked at 20–30°C (Table 3) and 30°C (Fig 3A–3D, Table 3), respectively (i.e., batches were smaller at 20 and 25°C than at 30°C). BPH produced more egg batches on T65 plants initiated at 20 DAS, but these were generally smaller than batches produced on other plants such that there were no apparent effects of variety or plant age on final egg numbers (Figs 2 and 3E–3H, Table 3).

**Table 3:** Results from univariate GLMs for adult female longevity and oviposition at the end of 20 days (see Fig 3).

Source of variation	DF	F-values^a^					
		BPH			WBPH		
		Adult longevity (days)	Number of batches	Number of eggs	Adult longevity (days)	Number of batches	Number of eggs
Temperature	5	22.047***	61.150***	49.707***	44.991***	16.669***	12.104***
Variety	1	2.057ns	4.665*	2.173ns	6.580**	6.100**	3.480ns
Plant age	1	0.044ns	5.218*	1.222ns	10.674***	43.356***	33.356***
Temperature*Variety	5	0.858ns	1.024ns	0.513ns	0.685ns	0.962ns	1.342ns
Temperature*Plant age	5	0.968ns	0.915ns	1.285ns	1.665ns	11.169***	5.202***
Variety*Plant age	1	1.203ns	0.001ns	0.135ns	2.538ns	0.141ns	1.809ns
Temperature*Variety*Plant age	5	0.294ns	0.432ns	0.514ns	0.870ns	1.682ns	1.534ns
Error	75						

^a^ ns = P > 0.05

** = P ≤ 0.01

*** = P ≤ 0.001

### Effects of temperature on WBPH longevity and oviposition

**Fig 4:**
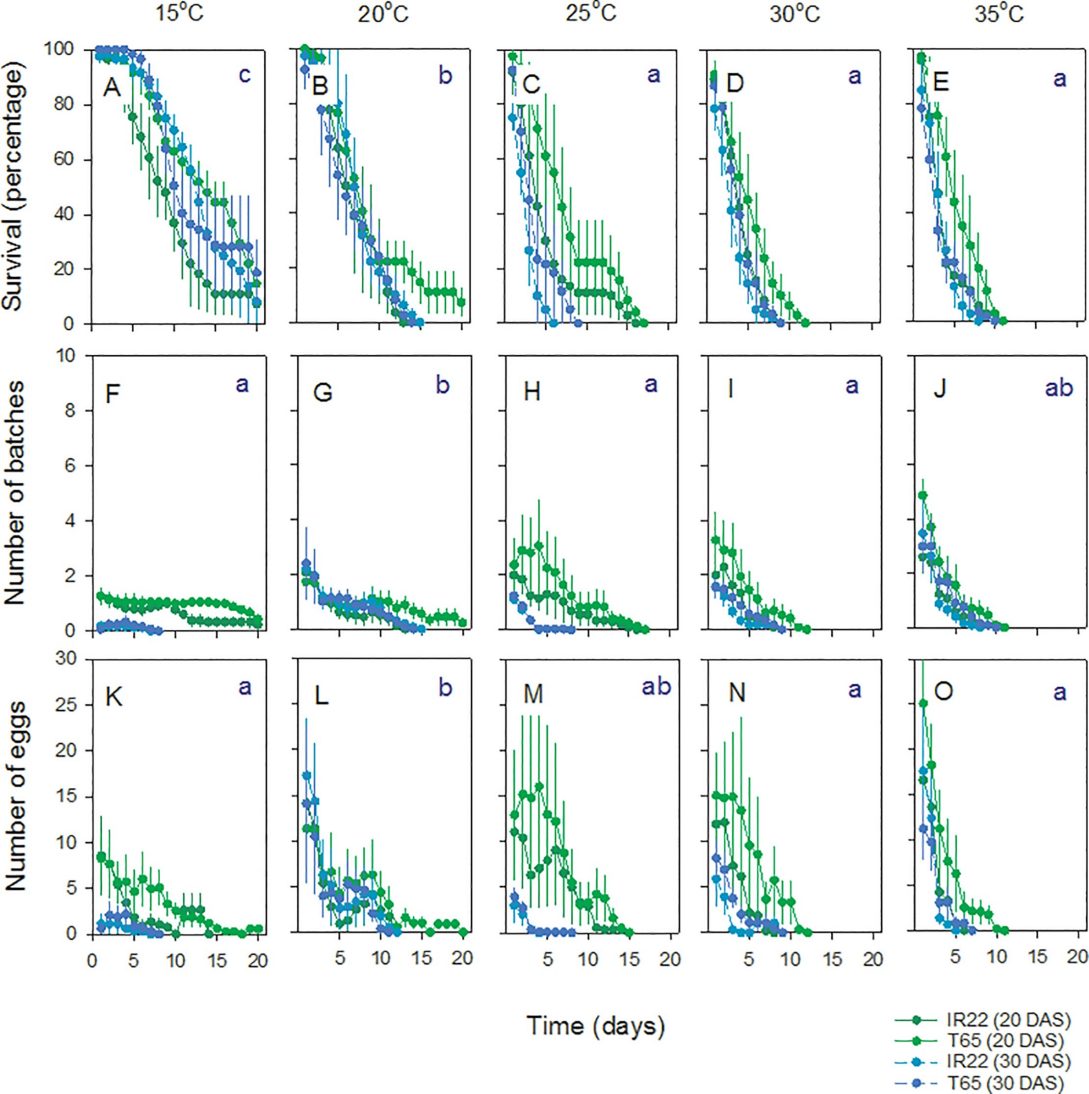
Adult WBPH female survival and egg-laying on *indica* (IR22) and *japonica* (T65) rice varieties. Infestations were initiated at 20 and 30 DAS; younger plants are indicated by green symbols and lines. Bioassays were conducted at 15°C (A,F,K), 20°C (B,G,L), 25°C (C,H,M), 30°C (D,I,N) and 35°C (E,J,O). Bioassays conducted at 40°C are not shown (see text). Results for adult survival during the experiment (A-E), the total numbers of batches produced (F-J), and the total number of eggs deposited (K-O) are presented. Standard errors are indicated (N = 4). Lowercase letters indicate homogenous temperature groups for each parameter (Tukey: P > 0.05) based on repeated measures GLMs. See also Table 2.

WBPH female survival, egg batch production and the number of eggs laid by WBPH declined over the course of the experiment (Fig 4). In general, the period of egg-laying was shorter in WBPH than in BPH, with most females ceasing to lay eggs at about 10–15 days. There were significant [Time*Temperature] interactions for batch and egg numbers because of different rates of egg-laying early in the experiment but similar low levels of oviposition at the end of the period analyzed (i.e., 10 days) (Fig 4F–4O; Table 2). The opposite occurred with female survival, with high survival at the beginning of the experiment and varying rates of decline across temperatures producing a significant [Time*Temperature] interaction (Fig 4A–4E; Table 2).

WBPH females survived for longest at 15°C (Fig 4A–4E; Table 2). At 25°C, female survival was greater on younger T65 plants than on other plants. WBPH produced more egg batches at 20 and 35°C. However, batches produced at 35°C were generally smaller, such that the highest numbers of eggs were produced at 20°C and 25°C (Fig 4F–4O). WBPH females produced more batches and laid more eggs on T65 and on younger rice plants (Fig 4F–4O; Table 2). There were significant [Temperature*Plant age] interactions for all three parameters because of similar values on plants initiated at 20 and 30 DAS at low (15°C for adult survival, 20°C for batches and eggs) and high (35°C for batches and eggs) temperatures, but greater differentiation in performance at 25 and 30°C (Fig 4, Table 2).

At the end of 20 days, WBPH female longevity (time to 0% survival) was greatest between 15–25°C (Fig 3I–3L). Females survived for longer on the *japonica* variety infested at 20 DAS (Fig 3I–3L). Egg laying in WBPH was highest at between 20–30°C, with a tendency for females to lay more eggs at 25°C (Fig 3M–3P; Table 3). Egg-laying by WBPH at the end of the experiment was highest on younger plants (initiated at 20 DAS), but was not affected by variety, although females produced more batches on T65 (Fig 3M–3P; Table 3). There were significant [Temperature*Plant age] interactions associated with WBPH oviposition because of low numbers of batches and eggs produced at 35–40°C, regardless of plant age (Fig 3M–3P; Table 3).

### Effects of temperature on growth and development of BPH nymphs

**Fig 5:**
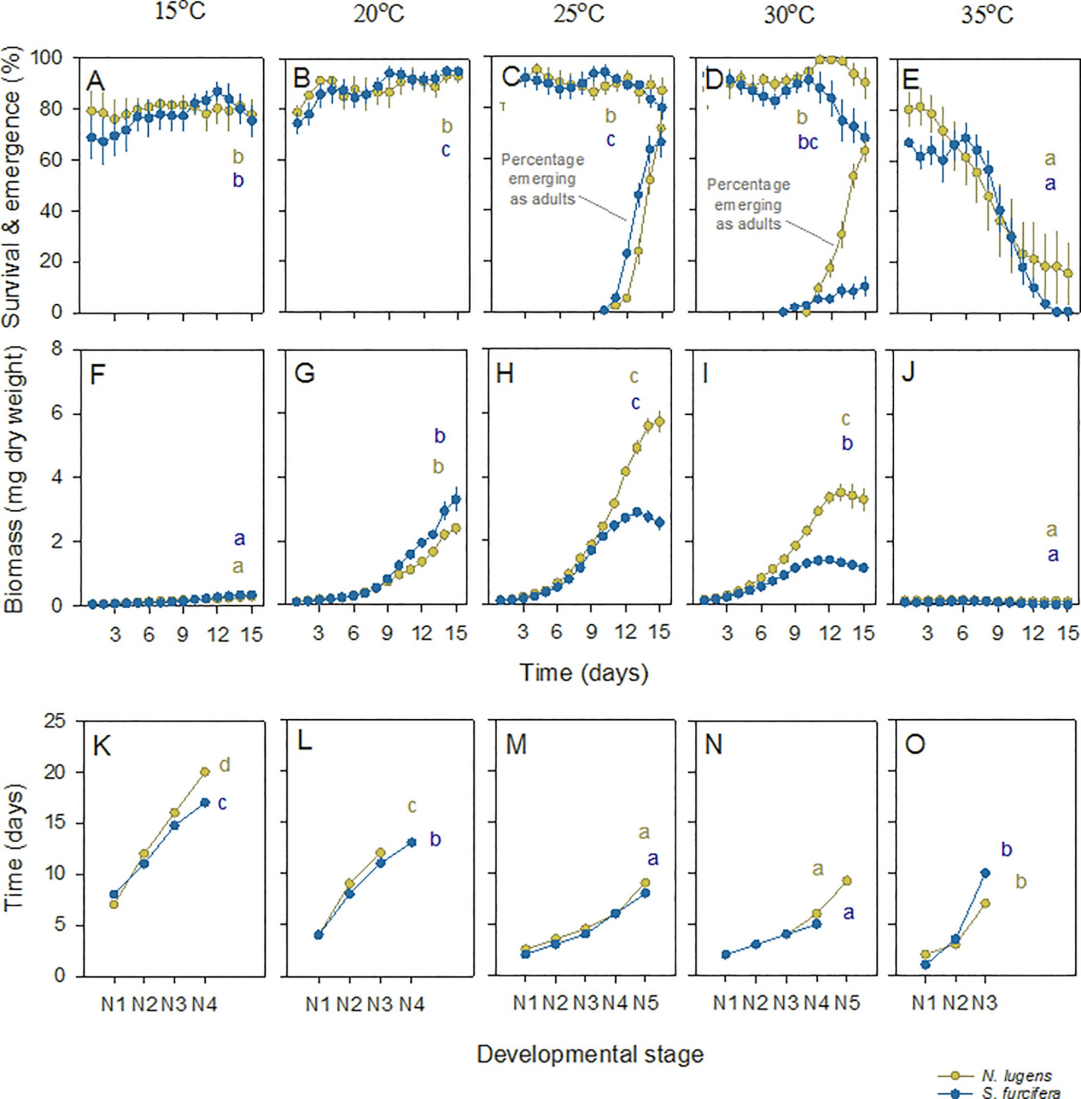
Effects of temperature on survival, weight gain and development of planthopper nymphs. BPH (brown symbols and lines) and WBPH (blue symbols and lines) nymphs were reared on IR22 (20 DAS) growing at 15°C (A,F,K), 20°C (B,G,L), 25°C (C,H,M), 30°C (D,I,N) and 35°C (E,J,O). Results for nymph survival (A-E), nymph biomass (F-J), and nymph developmental times (K-O) are indicated. The proportions of nymphs developing to adults are also indicated (C, D)(N1 = first instar, N2 = second instar, etc.). Standard errors are included (N = 4). Lowercase letters indicate homogenous temperature groups (Tukey, P > 0.05). See also Table 4. Note that analyses for K-O only include N1-N3.

Survival of BPH nymphs generally declined over time. High nymph survival at temperatures of between 15 and 30°C, but a rapid decline in survival at 35°C produced a significant [Time*Temperature] interaction (Fig 5A–5E; Table 4). BPH nymph biomass increased over the course of the experiment with large increases at between 20–30°C, but not at 15°C or 35°C, producing a significant [Time*Temperature] interaction (Fig 5F–5J; Table 4). BPH nymph development was generally faster at 25 and 30°C (multivariate GLM—N2 F_4,15_ = 1347.00, P < 0.001, N3 F_4,155_ = 1602.00, P < 0.001). Development of first instars showed a linear decline from 15 to 35°C (multivariate GLM—N1 F_4,15_ = 327.00, P < 0.001)(Fig 5K–5O).

**Table 4:** Results of repeated measures GLMs of nymph survival and biomass over 15 days (see Fig 5).

Source of variation	DF	F-values^a^			
		BPH		WBPH	
		Nymph survival	Nymph biomass	Nymph survival	Nymph biomass
*Within subject effects*					
Time	14	3.199***	367.900***	6.797***	221.146***
Time*Temperature	56	4.413***	73.532***	7.708***	41.469***
Time*Run	42	1.115ns	1.335ns	1.064ns	0.886ns
Error	168				
*Between subject effects*					
Temperature	4	22.955***	287.417***	65.636***	173.313***
Run	3	1.488ns	1.312ns	0.532ns	0.952ns
Error	12				

^a^ ns = P > 0.05

*** = P ≤ 0.001

Nymphs failed to develop beyond the fourth instar at 35°C and did not develop to adults at 15 and 20°C. We continued observations of nymph development in BPH until 30 and 23 days at 15°C and 20°C, respectively, without observing development to adults. There were no differences between the times for adult emergence (14.25 ± 0.25 days), the proportions of nymphs developing to adults before 15 days (0.87 ± 0.02), the proportions that were female (0.45 ± 0.04), and the proportions of brachypterous females (0.92 ± 0.06) or brachypterous males (0.38 ± 0.03) at 25 and 30°C (F_1,6_ ≤ 2.00, P ≥ 0.05)(Fig 5C and 5D).

**Fig 6:**
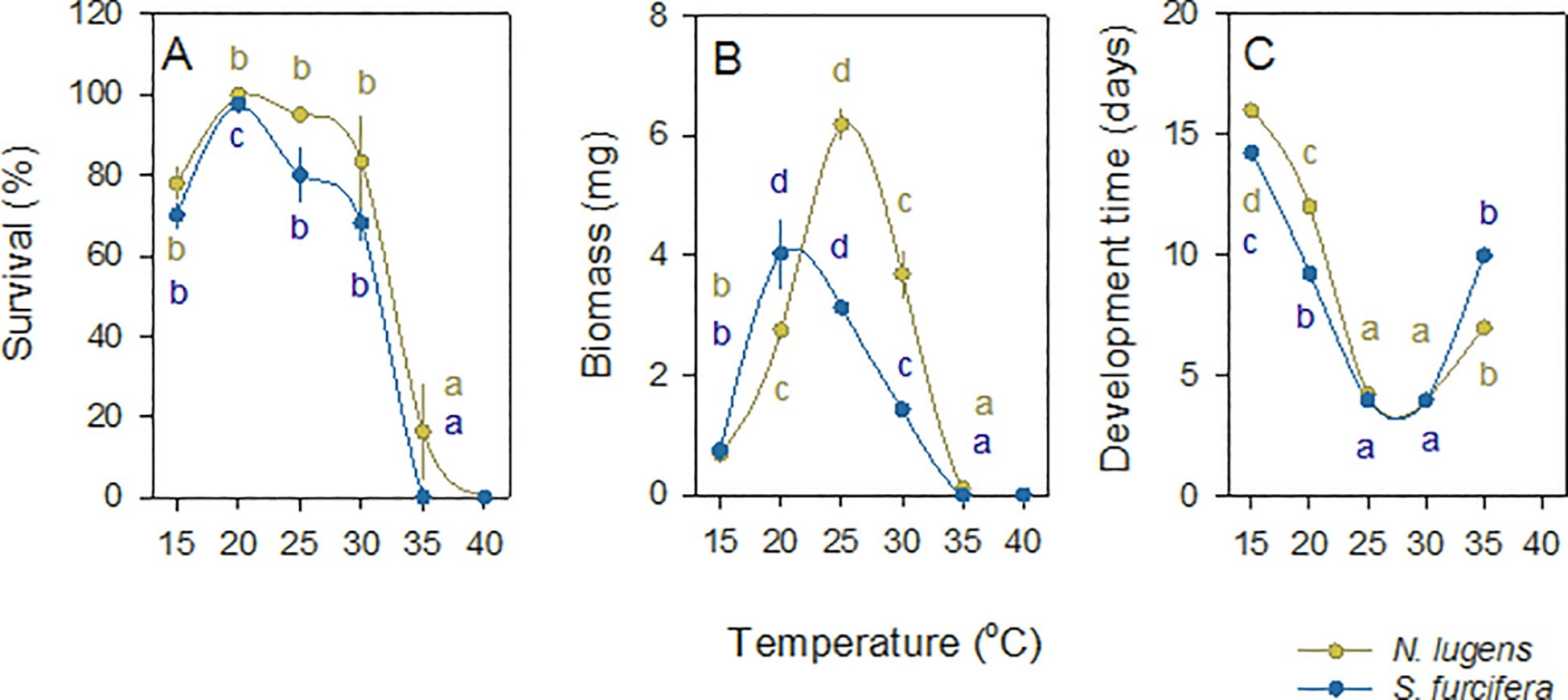
Effects of temperature on the survival and development of planthopper nymphs. BPH (brown symbols and lines) and WBPH (blue symbols and lines) nymphs were reared on IR22 at temperatures ranging from 15°C to 40°C. Temperature effects on (A) nymph survival, and (B) the maximum biomass of surviving nymphs, as well as (C) the time for 50% of nymphs to develop to third instars are indicated. Numbers are based on accumulated data over 15 days of the experiment. Bars indicate standard errors (N = 4). Lowercase letters indicate homogenous temperature groups (Tukey, P > 0.05) based on univariate (A and B) and multivariate (C) GLMs.

At the end of the experiment, BPH nymph survival was greatest at between 15–30°C (F_4,15_ = 19.384, P < 0.001) (Fig 6A). BPH nymphs showed a clear peak in biomass at 25°C (F_4,15_ = 163.867, P < 0.001)(Fig 6B) and nymph development was greatest at 25–30°C (Fig 6C).

### Effects of temperature on the growth and development of WBPH nymphs

Survival of WBPH nymphs declined over time (Fig 5A–5E). Survival was generally high at temperatures of between 15 and 25°C, but declined at temperatures above 30°C producing a significant [Time*Temperature] interaction (Fig 5A–5E; Table 4). There was a moderate decline in WBPH nymph survival towards the end of bioassays conducted at 30°C (Fig 5D).

Nymph biomass increased at 20–30°C producing a significant Time effect and a significant [Time*Temperature] interaction (Fig 5F–5J, Table 4). Second instar nymphs developed more quickly at 25 and 30°C (multivariate GLM—N2: F_4,15_ = 305.25, P < 0.001) and third instars at 25–35°C (N3: F_4,15_ = 833.752, P < 0.001). Development times of first instars was greatest at 35°C (N1: F_4,15_ = 117.00, P < 0.001)(Fig 5K–5O); however, nymphs failed to develop beyond the fourth instar at 35°C and did not develop to adults at 15 or 20°C during the 15 days of the experiments. We continued to monitor WBPH nymphs at 15°C for 30 days, during which < 1% of individuals developed to adults (after 27 days), and at 20°C for 23 days, by which time 4% had developed to adults.

Nymphs developed to adults at 25 and 30°C during the 15 days of observation. More nymphs developed to adults at 25°C (0.64±0.05) compared to 30°C (0.06+0.03) during the 15 days of observation (F_1,6_ = 107.769, P < 0.001) (Fig 5C and 5D). There was no effect of temperature (25 or 30°C) on the proportion of adults that were female (0.69±0.10: F_1,6_ = 1.062, P = 0.342) and all males were macropterous. All females that developed at 30°C were brachypterous, whereas at 25°C, 49±15% were brachypterous (F_1,6_ = 7.860, P = 0.031).

By 15 days, WBPH nymphs had the greatest survival at 20°C (F_4,15_ = 79.911, P < 0.001) (Fig 6A) with nymph biomass also peaking at 20°C (F_4,15_ = 128.423, P < 0.001)(Fig 6B) and nymph development fastest at 25–30°C (Fig 6C), but with lower adult emergence at the higher temperature.

### Temperature models for BPH and WBPH based on global data

**Fig 7:**
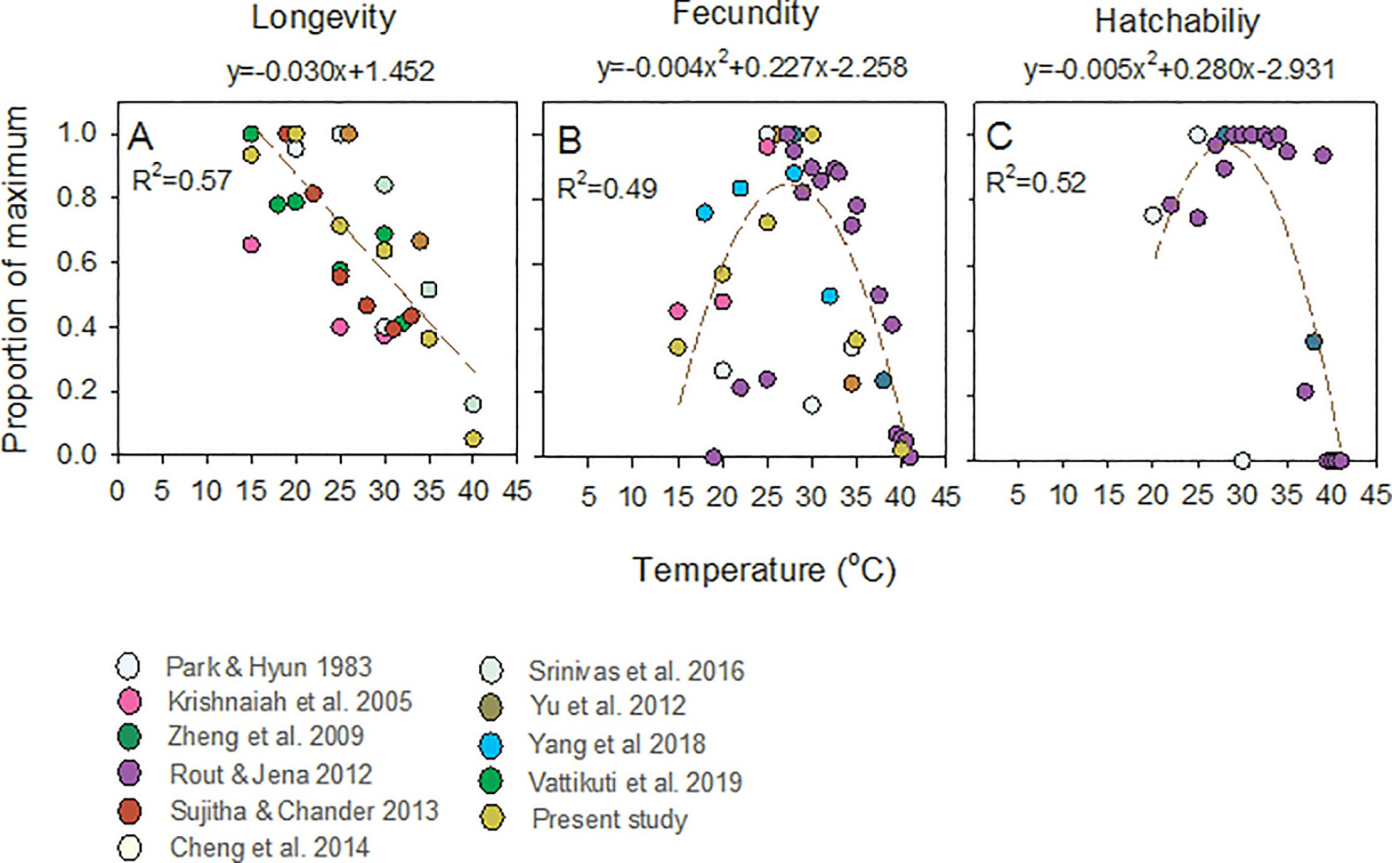
Temperature-dependent BPH (A) adult female longevity, (B) fecundity and (C) egg hatchability. Models are based on compiled published studies with means from the present study included. Lines, R^2^-values, and equations are for best fit models (based on averages at each temperature point [°C]; i.e., data for the same temperatures from different studies are averaged). For A, where studies distinguished male and female longevity, only females were included. Colored symbols represent data sources as indicated (see also Fig 1). Data from Bae and Pathak [44] were excluded because the lowest temperature used was 25°C.

There were insufficient published studies available to assess the effects of temperature on WBPH adult longevity or fecundity (i.e., less than 3 studies). However, for BPH, data were available from ten studies (Fig 7). Across the studies, BPH showed greatest longevity at between 15–25°C, declining in a linear manner until 40°C (Fig 7A). The relationship between temperature and longevity from the studies was best described by a linear model (six populations: F_1,28_ = 39.183, P < 0.001)(Fig 7A). Across eight populations, BPH fecundity peaked at between 25–32°C. The relationship was best described by a quadratic curve (F_2,39_ = 18.870, P < 0.001)(Fig 7B). We did not examine egg hatchability in our experiments; however, over 95% of eggs were viable in our study at between 20–30°C (data for other temperatures was not recorded). Among three studies that examined hatchability, a quadratic curve with a peak at between 25–34°C best described the relationship (F_2,18_ = 16.566, P < 0.001)(Fig 7C). Our standardized data for longevity and fecundity aligned closely with results from previous studies (Fig 7A and 7B).

**Fig 8:**
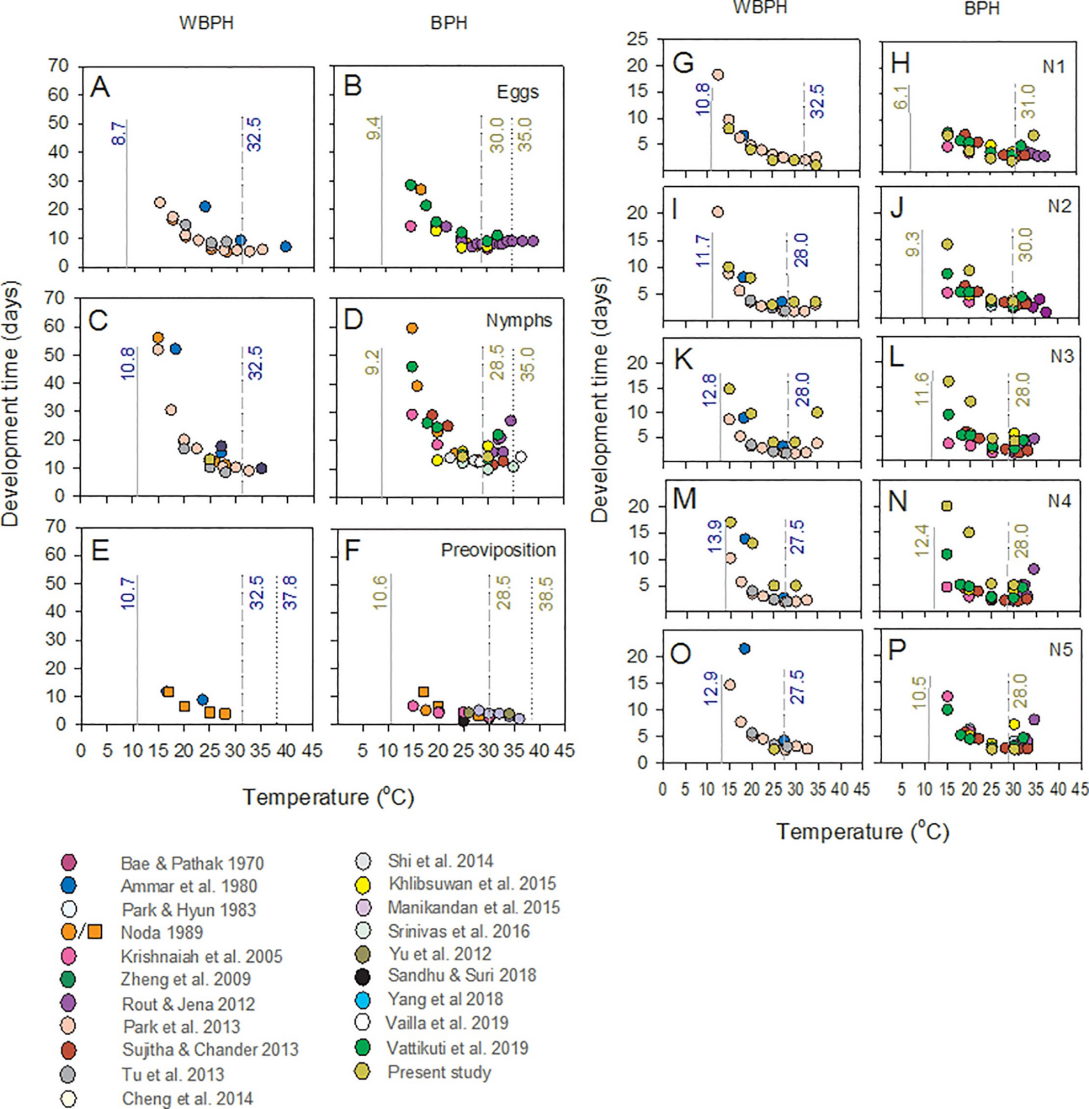
Temperature thresholds for development of BPH and WBPH. Responses to temperature for egg development in (A) WBPH and (B) BPH, nymph development in (C) WBPH and (D) BPH and the development of pre-ovipositional adult females of (E) WBPH and (F) BPH are indicated. Responses are based on published studies and include data from the present study. Development of first (G,H), second (I,J), third (K,L), fourth (M,N) and fifth (O,P) instars are also shown for WBPH (G,I,K,M,O) and BPH (H,J,L,N,P). Thresholds for developmental zero (solid lines), the limits of linear temperature-related increases in development (dashed lines), and maximum critical temperatures (dotted lines) are indicated where available. Numbers are thresholds for WBPH (blue) and BPH (brown)(see also Tables 1 and 5). Colored symbols represent data sources as indicated in the legend (see also Fig 1 for population sources).

**Fig 9:**
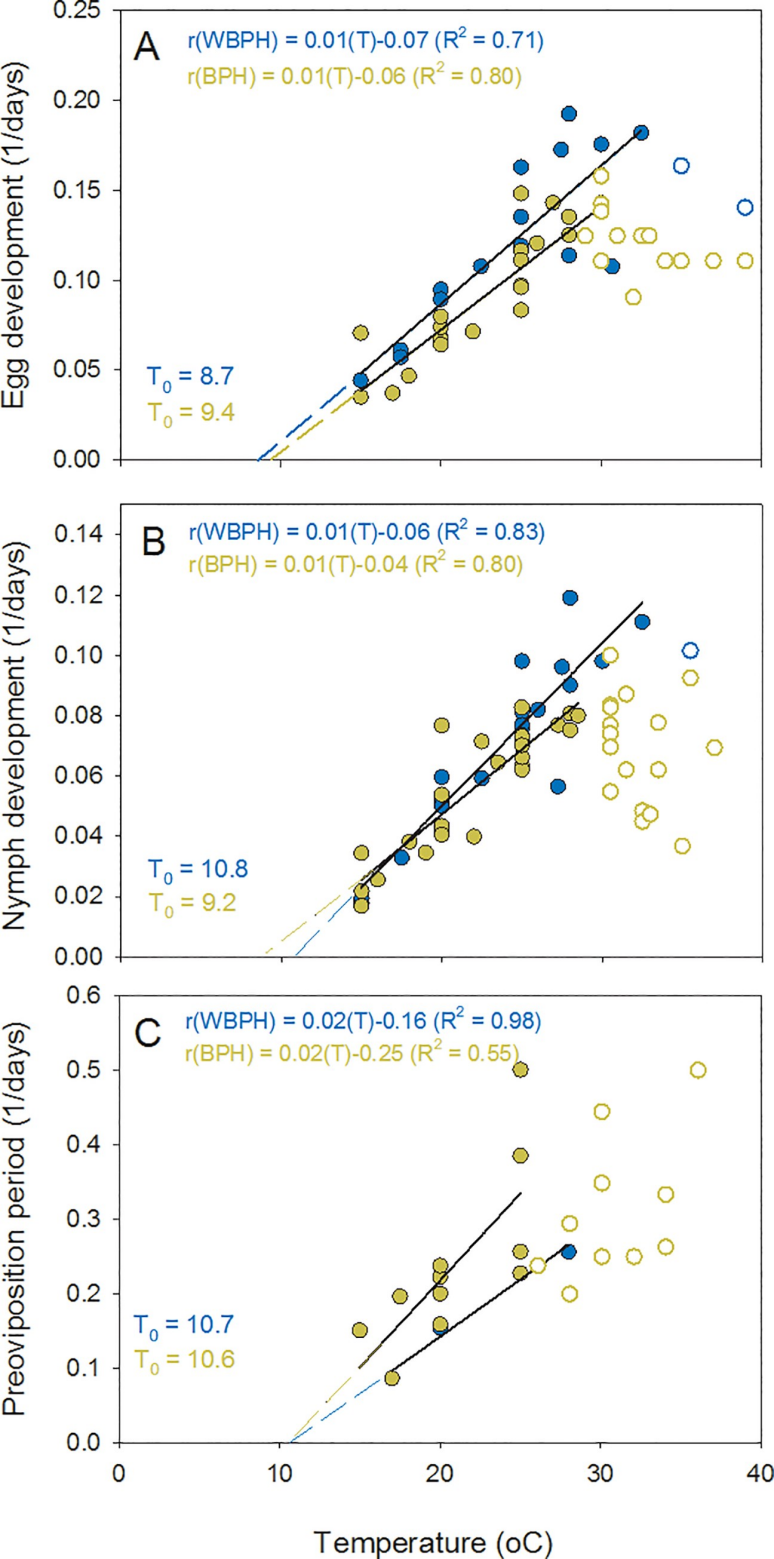
Linear relations between temperature and development rates. Graphs indicate (1/development time) for (A) eggs, (B) nymphs, and (C) pre-ovipositional adults of BPH (solid brown symbols) and WBPH (solid blue symbols). Non-linear portions of the relations are indicated by open brown symbols (BPH) and open blue symbols (WBPH). Estimates of developmental zeros (T_0_) are indicated based on the Campbell model with corresponding equations and R^2^s indicated in brown (BPH) and blue (WBPH) font.

BPH and WBPH had similar response curves for each development stage (Fig 8A–8F). Using the Campbell model, we estimated the lower thresholds for development of WBPH eggs, nymphs and adults as 8.7 (F_1,15_ = 34.586, P < 0.001), 10.8 (F_1,17_ = 80.193, P < 0.001), and 10.7°C (F_1,3_ = 91.102, P = 0.01), respectively, and for BPH eggs, nymphs and adults as 9.4 (F_1,24_ = 93.046, P < 0.001), 9.2 (F_1,25_ = 95.617, P < 0.001), and 10.6°C (F_1,10_ = 10.871, P = 0.009), respectively (Fig 9). The upper thresholds for development across studies were 30.0, 28.5, and 28.5°C for BPH eggs, nymphs and adults, respectively, and 32.5°C for eggs, nymphs and adults of WBPH. Using the global data sets, thermal constants were estimated as 147, 233, and 43 degree days, for BPH eggs, nymphs and adults (pre-oviposition period), respectively, and 130, 185, and 65 degree days, for WBPH eggs, nymphs and adults (pre-oviposition period), respectively. Our data for nymphs that successfully completed development (i.e., only at 25 and 30°C for both planthopper species) closely fitted the global model (Fig 7C and 7D). Our results for first instar development were similar to previous studies (Fig 8G and 8H), but for other instars, our estimates of development times were among the highest at low and high temperatures (i.e., ≤ 20°C and 35°C)(Fig 8I–8N). Whereas the analysis of nymph development indicated largely similar T_max_ values for BPH and WBPH (between 27.5–32.5°C, slightly higher in WBPH), the models also suggested that BPH have consistently lower T_min_ values than WBPH for each instar (i.e., ~2°C lower than WBPH); however, BPH nymphs required more degree days to complete each instar (Table 5).

**Table 5:** Threshold estimates based on Campbell model for nymph development using available data (see also Fig 8).

Species/instar	DF	F-value^a^	R^2^	b	T_min_ (°C)^b^	T_max_ (°C)^c^	k (degree days)^d^
BPH							
N1	10	59.236***	.894	.014	6.1	31.0	71.4
N2	8	91.322***	.938	.049	9.3	30.0	52.6
N3	9	68.921***	.932	.025	11.6	28.0	40.0
N4	7	57.055***	.919	.028	12.4	28.0	35.7
N5	8	102.653***	.954	.021	10.5	28.0	47.6
WBPH							
N1	6	329.164***	.973	.024	10.8	32.5	41.7
N2	7	35.276***	.815	.030	11.7	28.0	33.3
N3	6	30.516***	.813	.033	12.8	28.0	30.3
N4	7	30.957***	.838	.031	13.9	27.5	32.3
N5	6	20.725***	.775	.024	12.9	27.5	41.7

^a^ *** = P < 0.001

^b^ T_max_ estimated based on initial decline in mean 1/development time

^c^ T_min_ estimated as y = 0

^d^ Thermal constant (k) = 1/b, where b = slope of regression

## Discussion

A number of studies have shown that BPH and WBPH have similar temperature tolerances. We reviewed these previous studies and present their main findings in Table 1. Most notably, Noda [34] described low temperature development thresholds for BPH and WBPH eggs as 12.7 and 12.6°C, respectively, and for BPH and WBPH nymphs as 11.3 and 11.2°C, respectively. At the other extreme, in a recent comparative study, the effective lethal temperatures for adult female BPH and WBPH were estimated as 41.6 and 41.2°C, respectively [33]. The maximum effective temperature for BPH nymph development has been estimated by several authors at about 35°C [10,11,51]. Our results also indicate that the development of both BPH and WBPH nymphs is severely restricted at ≥ 35°C. Our assessment of temperature-dependent development further indicated that for both species the development of eggs, nymphs and pre-oviposition adults each showed similar responses to temperature—albeit with a lower estimate of developmental zero for eggs in WBPH and generally higher developmental zeros for WBPH nymphs and adults, compared to the respective development stages in BPH (Fig 9, Table 5). Together, these trends might suggest that WBPH and BPH respond similarly to ambient temperatures and that, compared to BPH, the apparently wider distribution range of WBPH in northern latitudes is due to the oligophagous nature of WBPH and the wide availability of food plants such as wheat and corn at high latitudes. However, whereas food availability may partially explain the distribution of these species, our results now clarify that WBPH performs better at lower temperatures than BPH within the ranges of tolerable temperatures, and may therefore be less restricted—or perform relatively better than BPH—at the cool temperatures of higher latitudes. Furthermore, estimated thermal constants based on the combined results of previous studies (Table 5), indicate that, despite generally higher T_min_ values than in BPH, WBPH may require fewer degree days to complete egg development and to complete the development of each nymph instar. These results demonstrate a decoupling of temperature tolerances and development thresholds from other aspects of planthopper life history (e.g., survival rates, biomass accumulation, and feeding and developmental success) that might better determine crop damage potentials under varying temperatures.

Temperature thresholds and nymph development rates may be similar between BPH and WBPH because they display similar molecular responses and tolerance mechanisms in coping with changing temperatures [69]. Furthermore, in the case of eggs and nymphs, upper thresholds for development are heavily determined by the detrimental effects of high temperatures on yeast like symbionts (YLS). YLS have been studied extensively in BPH and much is known of their biology and ecology [70]. BPH eggs and nymphs fail to develop when YLS numbers are depleted through heat (~35°C) treatment [71,72]. Similar YLS are known to occur in WBPH [70,73] and are probably responsible for the same upper limits to WBPH nymph survival as for BPH. In effect, 35°C is about the upper thermal tolerance for YLS. Survival rates, developmental success (as opposed to development rates) and feeding and food conversion efficiency (indicated by biomass accumulation) are also determined by the interactions between planthoppers and either their host plant or endosymbionts, or by interactions between all three (planthoppers, host plant and endosymbionts) within tolerable temperature limits. According to our results, although BPH and WBPH are subject to the same physiological restrictions at high and low temperatures, WBPH is better adapted than BPH to feed on rice at lower temperatures. Indeed, our results suggest that WBPH nymphs might gain some advantage from periods of low (~20°C) temperature, where they continue to feed and grow for longer (Fig 6), particularly if development accelerates during subsequent periods of higher temperature to produce larger individuals than would occur under consistently high (~ 25°C) temperatures (i.e., temperature-size rule [17,74]). In rice ecosystems, a decline in the efficiency of predators and parasitoids at cooler temperatures [75–78], further suggests that periods of low temperatures might be advantageous for WBPH, despite prolonged nymph development. These ideas require further testing.

Acclimation could potentially alter the shape of planthopper responses to temperature. For example, Piyaphongkul et al. [11] demonstrated that BPH temperature tolerances could be raised or lowered where planthoppers were acclimated to higher or lower temperatures, respectively. Furthermore, these authors suggested that BPH could acclimate better to low temperatures than to high temperatures [11]. In contrast, the results of several studies of temperature effects on BPH, as indicated in Fig 7, demonstrate largely similar longevity, fecundity and hatchability responses to temperature irrespective of population origin. Trade-offs between longevity and fecundity at high and low temperatures generally resulted in maximum oviposition by BPH at 30°C and by WBPH at 25°C in the present study. Although we did not estimate hatchability, optimal temperatures for oviposition in both species were below detrimental temperatures for hatching according to previous studies [35,37,43,48,52]. High egg production during relatively short time periods at 35°C in both species, as observed in the present study, suggests that females rapidly became ‘spent’ after intense egg-laying, or that rapid egg-laying was a stress response to the high temperature. The relatively low fecundities at between 20–25°C in a study by Rout and Jena [48] are likely related to the very low levels of relative humidity (RH) at these temperatures in that study (i.e., humidity was not standardized in the experiments and at 20–25°C, RHs were below 70%, which is regarded as a lower limit for BPH survival [79]). The source populations for studies ranged from cool temperate locations in Japan and Korea [34,35] to hot tropical locations in southern India [19,46](Fig 1). Despite the great range of ambient climates across these locations, the development responses by different planthopper populations to temperatures were remarkably similar (Fig 8). Our results for second, third and fourth instars, of BPH particularly, deviated most from the average models. This was probably reflective of the failure of BPH nymphs to develop to more advanced instars at these low temperatures in our study and suggests that our colonies may have been relatively poorly adapted to low temperatures due to intergenerational selection or inbreeding depression. This would have occurred because our source colonies were maintained for several years in a greenhouse that often reached high temperatures, but rarely fell below 25°C [80]. Where details of colony maintenance are available from the other studies, planthopper colonies were relatively newer, or the colonies had been maintained at relatively lower temperatures. Furthermore, in studies of temperature-dependent development, most researchers do not report on rates of nymph survival to adult (i.e., development success), but instead rely only on the survivors to calculate development rates (i.e., only Sandhu and Suri [54] reported nymph survival under different temperature gradients; but these authors did not assess development rates). Such studies may often begin with large numbers of test subjects to eventually attain data from a few surviving individuals from which to build temperature models. In contrast, our data for development until fourth instars at low and high temperatures mainly include individuals that would eventually fail to develop to adults. Nevertheless, it is noteworthy that our WBPH colony, which was maintained under the same conditions as the BPH colony, still displayed apparently higher performance under comparatively lower temperatures. Furthermore, our BPH response curves for adult longevity and fecundity were similar to those from previous studies (Fig 7A and 7B), and where nymphs did survive to adults (i.e., at 25 and 30°C), our estimates of development times were closely aligned with those from previously published studies (Fig 8C, 8D , 8O and 8P). These effects of temperature on adult longevity, fecundity, hatchability and nymph development rates are ultimately determined by interactions between the planthoppers and their biotic environment [21,70]. For example, poor nymph development at low temperatures could be partially due to lower sugar concentrations in rice sap at these temperatures with temperature-dependent changes in plant chemistry further affecting responses at temperatures other than optimal [22]. Life-history parameters may have appeared highly stable across populations in previous studies because relatively standard, susceptible rice seedlings were used in all the studies and the studies mainly focused on BPH. However, our results also indicate that varying host plant quality can alter the shape of reaction norms, particularly in experiments with WBPH.

Because of the wide distribution ranges of BPH and WBPH and the influence of vegetation, topography, wind or rainfall patterns, and distances to the sea on temperature isoclines, it is difficult to make general predictions regarding the effects of global temperature changes on planthopper abundance at different latitudes. Our results suggest that, compared to BPH, WBPH may be adversely affected by rising temperatures across a greater range of latitudes if average temperature during the cropping season increase beyond 25°C. Similarly, temperatures of above 30°C will adversely affect BPH populations. We used constant temperatures and constant humidity in our experiments. Studies have shown that results from experiments conducted under constant temperatures can differ from those conducted under fluctuating temperatures for some herbivore species [1]. We are unaware of the effects of daily temperature fluctuations on BPH or WBPH development, although female BPH deposit fewer eggs as temperatures drop (and humidity increases) during the tropical nighttime [58]. In tropical rice fields, maximum (daytime) temperatures and minimum (nighttime) temperatures can differ by > 10°C. Fluctuations tend to be of a lower magnitude in regions or during periods of cooler temperatures. Representing such complexities in climate chambers can be challenging. Using open-top field chambers with natural or elevated temperatures can produce more meaningful conditions [49], but will not give the temperature ranges that are possible from climate chamber studies. We expect that the patterns we observed will be largely maintained where temperatures fluctuate about the temperatures that we used in our experiments; however, we suggest that future studies might build on our results using fluctuating temperatures and humidity in environmental chambers, or by evaluating temperature effects in field cages.

Previous research has indicated that rice is more tolerant to damage (i.e., able to compensate for lost tissues) from WBPH than from BPH, and that rice sometimes overcompensates for damage from WBPH by increasing grain production [57]. Our results suggest that greater performance by female WBPH at relatively low temperatures (i.e., 20°C), at which plant growth rates are lower [40,81], could reduce the relative tolerance of rice to WBPH in cooler climates. This is because the size and growth rates of plant modules, such as rice tillers, are associated with tolerance to herbivores [57,82]. In contrast, faster growth rates of rice at higher temperatures, but a relatively low performance of WBPH at these same temperatures, could enhance plant tolerance. Similarly, at higher temperatures (~28–32°C), BPH will lay more eggs, but the damage they cause to rice plants is likely to decline (because of declining rates of feeding and weight gain), particularly if the plants also increase growth rates at higher temperatures. Such high temperatures have become increasingly prevalent in rice producing countries. For example, in 2019, temperatures of ≥ 30°C were experienced during 349 days at Kampong Chhnang, in Cambodia, where temperatures have increased by 0.23°C each decade since the 1950s [1]. Based on the results of the present study, such temperatures are predicted to favor BPH over WBPH because BPH continues to oviposit at night, whereas WBPH lay few eggs during darkness, irrespective of ambient temperatures [58]. High nighttime temperatures could, therefore, shift rice planthopper assemblages towards higher relative abundances of BPH.

There have been relatively few studies of the effects of temperature on the interactions between planthoppers and other components of the rice ecosystem. Some research has examined functional responses of the natural enemies of rice herbivores across temperature gradients [75,76], and studies have investigated the effects of temperature on interactions between planthoppers and the rice plant as mediated by nitrogen levels [43] or based on the presence of resistance genes [21,22]. WBPH outcompetes BPH for food and egg-laying resources where the species occur on the same rice plants [83,84]. Feeding by BPH also induces rice susceptibility to WBPH [84]. Our results indicate that, compared to WBPH, BPH is a more effective rice herbivore that performed well on *japonica* and *indica* rice varieties and on plants of two different ages. Differentiation between oviposition performance in the two planthopper species was greatest at 25–30°C (producing significant [Time*Temperature*Species] interactions for longevity, number of batches and number of eggs in the full-factorial analysis: S1 Table). This suggests that temperature could affect the partitioning of resources between BPH and WBPH in the rice ecosystem. In particular, resource partitioning may break down at temperatures that are lower or higher than optimal (Figs 2 and 4). We did not examine the performance of nymphs on different plants across temperatures (previous studies have been conducted at about optimal [58,84]); however, similar temperature-dependent differentiation in feeding responses to plant quality or condition might occur with nymphs. Therefore competition between the two species may be relaxed at optimal temperatures for the superior competitor, because only at optimal temperatures does host-plant quality (variety or age) differentially affect performance.

## Conclusions

Our results indicate the limitations of thermal tolerances and temperature thresholds for development in predicting the impact of global warming on the relative incidences or potential for outbreaks of two planthopper pests in rice. These traits are largely similar for BPH and WBPH, and whereas they may be used to model distribution and overwintering ranges of the two species, they say relatively little about the potential impacts of climate on relative damage by the pests or interactions between the pests over their normal distribution ranges. We determined that for WBPH, the optimal temperatures for adult and nymph survival, fecundity, nymph biomass gain and development success were all lower than for BPH. Whereas temperature tolerances may be affected by acclimation, a review of planthopper performance (longevity, fecundity and development rates) across multiple studies with different planthopper populations displayed remarkably similar responses to temperature. These factors are affected by host plant quality, including aspects of plant ontogeny. Our results with planthopper oviposition, suggest that plant quality will have its greatest effects at about the optimal temperatures for each planthopper, thereby increasing the potential for resource partitioning between different planthopper species and increasing the opportunities for planthopper coexistence at optimal temperatures. Differences in the growth rates of rice plants at different temperatures, and potentially different responses by different rice varieties to temperature, could determine relative levels of plant tolerance to planthopper damage under global warming. We suggest that further studies could examine the potential effects of temperature, and other climatic changes, on herbivore-herbivore interactions and herbivore-plant interactions to improve predictions about pest pressures under global warming.

## Supporting information

S1 Fig Effect of daily manipulation of feeding plants on longevity of adult female BPH and WBPH. (DOCX)

S1 Table Results from repeated measures GLM of adult female longevity and oviposition parameters with planthopper species included as an independent factor. (DOCX)

S2 Table Results from univariate GLMs of adult female longevity and oviposition at the end of 20 days with planthopper species included as an independent factor. (DOCX)

S3 Table Results of repeated measures GLMs of nymph survival and biomass over 15 days with planthopper species included as an independent factor. (DOCX)

S4 Table Results of multivariate GLM of nymph development time with species included as an independent factor. (DOCX)

S5 Table Data from environmental chamber studies of responses by ovipositing planthoppers to temperature. (DOCX)

S6 Table Data from environmental chamber studies of responses by planthopper nymphs to temperature. (DOCX)
