# Supplementary material for: Temperature-dependent oviposition and nymph performance reveal distinct thermal niches of coexisting planthoppers with similar thresholds for development
Source: PLoS One. 2020 Jun 30;15(6):e0235506. doi: 10.1371/journal.pone.0235506 (PMC7326231; doi:10.1371/journal.pone.0235506)
Supplement: S1 Fig — (DOCX) [file pone.0235506.s001.docx]

**Figure S1. Effect of daily manipulation of feeding plants on longevity of adult female BPH and WBPH.** An experiment was conducted with adult BPH and WBPH on potted rice seedlings (T65) in acetate cages. Each cage had a single rice seedling with a single gravid female of either planthopper species. The cages were placed in climate chambers at 20, 25, 30 or 35°C. Cages were divided into two groups. For the first group, plants were changed each day by dislodging the planthoppers and replacing the plant with a non-infested seedling of the same age. At the same time, the condition of the planthoppers was recorded. For the second group, plants were not replaced, but planthoppers were assessed daily. There were 3 replicates for each species × day × temperature treatment. The experiment was continued until all planthoppers had died (28 days). A univariate general liner model (GLM) of the differences between survival in corresponding manipulated and non-manipulated groups (Δ longevity) indicated significant effects of planthopper species (F_1,16_ = 82.571, P < 0.001), temperature (F_3,16_ = 23.714, P < 0.001) and their interaction (F_3,16_ = 53.048, P < 0.001) on survival. Manipulation reduced the survival of BPH more than WBPH, had a greater impact at 30°C, but was not different between species at 20 and 35°C.
