# Supplementary material for: Temperature-dependent oviposition and nymph performance reveal distinct thermal niches of coexisting planthoppers with similar thresholds for development
Source: PLoS One. 2020 Jun 30;15(6):e0235506. doi: 10.1371/journal.pone.0235506 (PMC7326231; doi:10.1371/journal.pone.0235506)
Supplement: S1 Table — (DOCX) [file pone.0235506.s002.docx]

**Table S1. Results from repeated measures GLM of adult female survival and oviposition parameters with planthopper species included as an independent factor** (see Figures 2 and 4 and Table 2)

| \| Sources of variation \| DF \| F-Values^a^ \| \| \| \| --- \| --- \| --- \| --- \| --- \| \|  \|  \| Adult survival \| Egg batches \| Eggs \| \| *Within subject effects* \| \| \| \| \| \| Time \| 9 \| 430.933*** \| 127.865*** \| 112.827*** \| \| Time*Species \| 9 \| 5.800*** \| 7.042*** \| 6.409*** \| \| Time*Variety \| 9 \| 1.169ns \| 1.632ns \| 1.801ns \| \| Time*Plant age \| 9 \| 2.94*** \| 1.204ns \| 1.446ns \| \| Time*Temperature \| 36 \| 9.257*** \| 9.164*** \| 6.010*** \| \| Time*Run \| 27 \| 3.827*** \| 4.749*** \| 3.733*** \| \| Time*Species*Variety \| 9 \| 1.179ns \| 0.523ns \| 0.534ns \| \| Time*Species*Plant age \| 9 \| 1.033ns \| 0.724ns \| 0.390ns \| \| Time*Species*Temperature \| 36 \| 3.107*** \| 1.553*** \| 1.765*** \| \| Time*Variety*Plant age \| 9 \| 1.424ns \| 1.47ns \| 0.905ns \| \| Time*Variety*Temperature \| 36 \| 0.924ns \| 0.925ns \| 1.057ns \| \| Time*Plant age*Temperature \| 36 \| 1.059ns \| 1.317ns \| 1.555* \| \| Time*Species*Variety*Plant age \| 9 \| 3.308*** \| 0.543ns \| 1.209ns \| \| Time*Species*Variety*Temperature \| 36 \| 1.269ns \| 0.817ns \| 1.539* \| \| Time*Species*Plant age*Temperature \| 36 \| 1.172ns \| 0.978ns \| 0.720ns \| \| Time*Variety*Plant age*Temperature \| 36 \| 0.907ns \| 1.115ns \| 0.979ns \| \| Time*Species*Variety*Plant age*Temperature \| 36 \| 0.841ns \| 0.837ns \| 1.268ns \| \| Error \| 1053 \|  \|  \|  \| \| *Between subject effects* \| \| \| \| \| \| Species \| 1 \| 20.338*** \| 149.011*** \| 101.069*** \| \| Variety \| 1 \| 0.636ns \| 8.805*** \| 9.163*** \| \| Plant age \| 1 \| 0.496ns \| 13.170*** \| 11.816*** \| \| Temperature \| 4 \| 34.935*** \| 5.498*** \| 8.714*** \| \| Run \| 3 \| 3.453** \| 0.584ns \| 1.681ns \| \| Species*Variety \| 1 \| 4.594* \| 0.002ns \| 0.036ns \| \| Species*Plant age \| 1 \| 5.998** \| 8.326*** \| 12.100*** \| \| Species*Temperature \| 4 \| 4.776*** \| 4.773*** \| 3.995*** \| \| Variety*Plant age \| 1 \| 0.597ns \| 0.323ns \| 0.338ns \| \| Variety*Temperature \| 4 \| 0.674ns \| 1.373ns \| 1.160ns \| \| Plant age*Temperature \| 4 \| 0.429ns \| 0.897ns \| 0.247ns \| \| Species*Variety*Plant age \| 1 \| 2.265ns \| 0.515ns \| 1.628ns \| \| Species*Variety*Temperature \| 4 \| 0.390ns \| 0.523ns \| 0.696ns \| \| Species*Plant age*Temperature \| 4 \| 2.498ns \| 4.703ns \| 3.306** \| \| Variety*Plant age*Temperature \| 4 \| 0.484ns \| 0.702ns \| 0.785ns \| \| Species*Variety*Plant age*Temperature \| 4 \| 0.069ns \| 0.565ns \| 0.640ns \| \| Errors \| 117 \|  \|  \|  \| |  |
| --- | --- | --- | --- | --- | --- | --- | --- | --- | --- | --- | --- | --- | --- | --- | --- | --- | --- | --- | --- | --- | --- | --- | --- | --- | --- | --- | --- | --- | --- | --- | --- | --- | --- | --- | --- | --- | --- | --- | --- | --- | --- | --- | --- | --- | --- | --- | --- | --- | --- | --- | --- | --- | --- | --- | --- | --- | --- | --- | --- | --- | --- | --- | --- | --- | --- | --- | --- | --- | --- | --- | --- | --- | --- | --- | --- | --- | --- | --- | --- | --- | --- | --- | --- | --- | --- | --- | --- | --- | --- | --- | --- | --- | --- | --- | --- | --- | --- | --- | --- | --- | --- | --- | --- | --- | --- | --- | --- | --- | --- | --- | --- | --- | --- | --- | --- | --- | --- | --- | --- | --- | --- | --- | --- | --- | --- | --- | --- | --- | --- | --- | --- | --- | --- | --- | --- | --- | --- | --- | --- | --- | --- | --- | --- | --- | --- | --- | --- | --- | --- | --- | --- | --- | --- | --- | --- | --- | --- | --- | --- | --- | --- | --- | --- | --- | --- | --- | --- | --- | --- | --- | --- | --- | --- | --- | --- | --- | --- | --- | --- | --- | --- | --- | --- | --- | --- | --- | --- | --- | --- | --- | --- | --- | --- | --- | --- | --- |

^a^ ns = P > 0.05, * P ≤ 0.05, ** = P ≤ 0.01, *** = P ≤ 0.001
