# Supplementary material for: Temperature-dependent oviposition and nymph performance reveal distinct thermal niches of coexisting planthoppers with similar thresholds for development
Source: PLoS One. 2020 Jun 30;15(6):e0235506. doi: 10.1371/journal.pone.0235506 (PMC7326231; doi:10.1371/journal.pone.0235506)
Supplement: S2 Table — (DOCX) [file pone.0235506.s003.docx]

**Table S2. Results from univariate GLMs of adult female longevity and oviposition at the end of 20 days with planthopper species included as an independent factor** (see Figure 3 and Table 3)

| Sources of variation | DF | F-Values^a^ | | | |
| --- | --- | --- | --- | --- | --- |
|  |  | Longevity (50%) | Longevity (0%) | Egg batches | Eggs |
| Temperature | 5 | 36.245*** | 55.155*** | 63.362*** | 45.258*** |
| Species | 1 | 7.839** | 8.655*** | 172.171*** | 95.635*** |
| Variety | 1 | 0.004ns | 0.201ns | 10.748*** | 5.652** |
| Plant age | 1 | 0.846ns | 3.404ns | 40.337*** | 27.678*** |
| Temperature*Species | 5 | 2.916** | 6.328*** | 12.050*** | 6.654*** |
| Temperature*Variety | 5 | 0.269ns | 0.358ns | 1.149ns | 0.821ns |
| Temperature*Plant age | 5 | 1.697ns | 0.648ns | 3.719*** | 1.718ns |
| Species*Variety | 1 | 3.308ns | 7.340** | 0.095ns | 0.346ns |
| Species*Plant age | 1 | 3.308ns | 4.741* | 10.301*** | 15.360*** |
| Variety*Plant age | 1 | 1.765ns | 0.014ns | 0.088ns | 1.668ns |
| Temperature*Species*Variety | 5 | 0.849ns | 1.227ns | 0.833ns | 1.253ns |
| Temperature*Species*Plant age | 5 | 0.601ns | 1.816ns | 8.921*** | 5.800*** |
| Temperature*Variety*Plant age | 5 | 1.046ns | 0.649ns | 1.138ns | 1.199ns |
| Species*Variety*Plant age | 1 | 0.401ns | 3.404ns | 0.062ns | 0.716ns |
| Temperature*Species*Variety*Plant age | 5 | 0.182ns | 0.376ns | 1.044ns | 1.117ns |
| Error | 144 |  |  |  |  |

^a^ ns = P > 0.05, * P ≤ 0.05, ** = P ≤ 0.01, *** = P ≤ 0.001
