# Supplementary material for: Temperature-dependent oviposition and nymph performance reveal distinct thermal niches of coexisting planthoppers with similar thresholds for development
Source: PLoS One. 2020 Jun 30;15(6):e0235506. doi: 10.1371/journal.pone.0235506 (PMC7326231; doi:10.1371/journal.pone.0235506)
Supplement: S3 Table — (DOCX) [file pone.0235506.s004.docx]

**Table S3. Results of repeated measures GLMs of nymph survival and biomass over 15 days with planthopper species included as an independent factor** (see Figure 3 and Table 4)

| Source of variation | DF | F-value^a^ | |
| --- | --- | --- | --- |
|  |  | Survival | Biomass |
| *Within subject effects* | | | |
| Time | 14 | 7.140*** | 561.131*** |
| Time*Species | 14 | 2.444*** | 10.930*** |
| Time*Temperature | 56 | 9.943*** | 102.738*** |
| Time*Run | 42 | 1.030ns | 0.958ns |
| Time*Species*Temperature | 56 | 1.445* | 10.927ns |
| Error | 378 |  |  |
| *Between subject effects* | | | |
| Species | 1 | 2.700ns | 42.187*** |
| Temperature | 4 | 67.596*** | 430.619*** |
| Run | 3 | 1.119ns | 0.921ns |
| Species*Temperature | 4 | 0.460ns | 21.742*** |
| Error | 27 |  |  |

^a^ ns = P > 0.05, * P ≤ 0.05, ** = P ≤ 0.01, *** = P ≤ 0.001
