# Supplementary material for: Temperature-dependent oviposition and nymph performance reveal distinct thermal niches of coexisting planthoppers with similar thresholds for development
Source: PLoS One. 2020 Jun 30;15(6):e0235506. doi: 10.1371/journal.pone.0235506 (PMC7326231; doi:10.1371/journal.pone.0235506)
Supplement: S4 Table — (DOCX) [file pone.0235506.s005.docx]

**Table S4. Results of multivariate GLM of nymph development time with species included as an independent factor** (see Figure 3)

| Source of variation | DF | F-values^a^ | | |
| --- | --- | --- | --- | --- |
|  |  | N1 | N2 | N3 |
| Species | 1 | 3.000ns | 112.665*** | 5.143* |
| Temperature | 4 | 1572.000*** | 1160.534*** | 2344.339*** |
| Run | 3 | 1.000ns | 0.310ns | 1.714ns |
| Species*Temperature | 4 | 18.000ns | 54.466*** | 100.768*** |
| Error | 27 |  |  |  |

^a^  ns = P > 0.05, *** = P ≤ 0.001
