# Supplementary material for: Temperature-dependent oviposition and nymph performance reveal distinct thermal niches of coexisting planthoppers with similar thresholds for development
Source: PLoS One. 2020 Jun 30;15(6):e0235506. doi: 10.1371/journal.pone.0235506 (PMC7326231; doi:10.1371/journal.pone.0235506)
Supplement: S5 Table — (DOCX) [file pone.0235506.s006.docx]

**Table S5. Data from environmental chamber studies of responses by ovipositing planthoppers to temperature** (N1 = fist instar; N2 = second instar, etc.; SW = short wing)

| Run | Rice Variety | Temperature (oC) | Species | Day of observation | % N1 | % N2 | % N3 | % N4 | % N5 | % adults | % females | % males | % SW females | % SW males | nymph biomass |
| --- | --- | --- | --- | --- | --- | --- | --- | --- | --- | --- | --- | --- | --- | --- | --- |
| 1 | IR22 | 15 | BPH | 1 | 0.05 | 0.42 | 0.53 | 0.00 | 0.00 | 0.00 | 0.00 | 0.00 |  |  | 0.06 |
| 2 | IR22 | 15 | BPH | 1 | 0.00 | 0.33 | 0.67 | 0.00 | 0.00 | 0.00 | 0.00 | 0.00 |  |  | 0.06 |
| 3 | IR22 | 15 | BPH | 1 | 0.36 | 0.57 | 0.07 | 0.00 | 0.00 | 0.00 | 0.00 | 0.00 |  |  | 0.04 |
| 4 | IR22 | 15 | BPH | 1 | 1.00 | 0.00 | 0.00 | 0.00 | 0.00 | 0.00 | 0.00 | 0.00 |  |  | 0.08 |
| 1 | IR22 | 15 | BPH | 2 | 0.09 | 0.55 | 0.36 | 0.00 | 0.00 | 0.00 | 0.00 | 0.00 |  |  | 0.04 |
| 2 | IR22 | 15 | BPH | 2 | 0.00 | 0.08 | 0.92 | 0.00 | 0.00 | 0.00 | 0.00 | 0.00 |  |  | 0.06 |
| 3 | IR22 | 15 | BPH | 2 | 0.00 | 0.35 | 0.65 | 0.00 | 0.00 | 0.00 | 0.00 | 0.00 |  |  | 0.07 |
| 4 | IR22 | 15 | BPH | 2 | 1.00 | 0.00 | 0.00 | 0.00 | 0.00 | 0.00 | 0.00 | 0.00 |  |  | 0.09 |
| 1 | IR22 | 15 | BPH | 3 | 0.11 | 0.17 | 0.61 | 0.11 | 0.00 | 0.00 | 0.00 | 0.00 |  |  | 0.08 |
| 2 | IR22 | 15 | BPH | 3 | 0.00 | 0.00 | 0.10 | 0.90 | 0.00 | 0.00 | 0.00 | 0.00 |  |  | 0.06 |
| 3 | IR22 | 15 | BPH | 3 | 0.00 | 0.19 | 0.81 | 0.00 | 0.00 | 0.00 | 0.00 | 0.00 |  |  | 0.07 |
| 4 | IR22 | 15 | BPH | 3 | 1.00 | 0.00 | 0.00 | 0.00 | 0.00 | 0.00 | 0.00 | 0.00 |  |  | 0.13 |
| 1 | IR22 | 15 | BPH | 4 | 0.06 | 0.19 | 0.44 | 0.31 | 0.00 | 0.00 | 0.00 | 0.00 |  |  | 0.09 |
| 2 | IR22 | 15 | BPH | 4 | 0.00 | 0.00 | 0.21 | 0.79 | 0.00 | 0.00 | 0.00 | 0.00 |  |  | 0.08 |
| 3 | IR22 | 15 | BPH | 4 | 0.00 | 0.00 | 0.14 | 0.86 | 0.00 | 0.00 | 0.00 | 0.00 |  |  | 0.08 |
| 4 | IR22 | 15 | BPH | 4 | 1.00 | 0.00 | 0.00 | 0.00 | 0.00 | 0.00 | 0.00 | 0.00 |  |  | 0.14 |
| 1 | IR22 | 15 | BPH | 5 | 0.00 | 0.00 | 0.40 | 0.60 | 0.00 | 0.00 | 0.00 | 0.00 |  |  | 0.11 |
| 2 | IR22 | 15 | BPH | 5 | 0.00 | 0.00 | 0.15 | 0.54 | 0.31 | 0.00 | 0.00 | 0.00 |  |  | 0.09 |
| 3 | IR22 | 15 | BPH | 5 | 0.00 | 0.00 | 0.29 | 0.47 | 0.24 | 0.00 | 0.00 | 0.00 |  |  | 0.12 |
| 4 | IR22 | 15 | BPH | 5 | 1.00 | 0.00 | 0.00 | 0.00 | 0.00 | 0.00 | 0.00 | 0.00 |  |  | 0.18 |
| 1 | IR22 | 15 | BPH | 6 | 0.13 | 0.20 | 0.13 | 0.53 | 0.00 | 0.00 | 0.00 | 0.00 |  |  | 0.10 |
| 2 | IR22 | 15 | BPH | 6 | 0.00 | 0.00 | 0.00 | 0.13 | 0.88 | 0.00 | 0.00 | 0.00 |  |  | 0.14 |
| 3 | IR22 | 15 | BPH | 6 | 0.00 | 0.00 | 0.00 | 0.11 | 0.89 | 0.00 | 0.00 | 0.00 |  |  | 0.14 |
| 4 | IR22 | 15 | BPH | 6 | 1.00 | 0.00 | 0.00 | 0.00 | 0.00 | 0.00 | 0.00 | 0.00 |  |  | 0.16 |
| 1 | IR22 | 15 | BPH | 7 | 0.00 | 0.00 | 0.06 | 0.38 | 0.56 | 0.00 | 0.00 | 0.00 |  |  | 0.15 |
| 2 | IR22 | 15 | BPH | 7 | 0.00 | 0.00 | 0.00 | 0.06 | 0.94 | 0.00 | 0.00 | 0.00 |  |  | 0.18 |
| 3 | IR22 | 15 | BPH | 7 | 0.00 | 0.00 | 0.00 | 0.00 | 1.00 | 0.00 | 0.00 | 0.00 |  |  | 0.15 |
| 4 | IR22 | 15 | BPH | 7 | 0.47 | 0.53 | 0.00 | 0.00 | 0.00 | 0.00 | 0.00 | 0.00 |  |  | 0.18 |
| 1 | IR22 | 15 | BPH | 8 | 0.00 | 0.00 | 0.00 | 0.06 | 0.94 | 0.00 | 0.00 | 0.00 |  |  | 0.18 |
| 2 | IR22 | 15 | BPH | 8 | 0.00 | 0.00 | 0.00 | 0.14 | 0.86 | 0.00 | 0.00 | 0.00 |  |  | 0.12 |
| 3 | IR22 | 15 | BPH | 8 | 0.00 | 0.00 | 0.00 | 0.00 | 1.00 | 0.00 | 0.00 | 0.00 |  |  | 0.17 |
| 4 | IR22 | 15 | BPH | 8 | 0.14 | 0.86 | 0.00 | 0.00 | 0.00 | 0.00 | 0.00 | 0.00 |  |  | 0.20 |
| 1 | IR22 | 15 | BPH | 9 | 0.00 | 0.00 | 0.00 | 0.00 | 1.00 | 0.00 | 0.00 | 0.00 |  |  | 0.18 |
| 2 | IR22 | 15 | BPH | 9 | 0.00 | 0.00 | 0.00 | 0.06 | 0.94 | 0.00 | 0.00 | 0.00 |  |  | 0.17 |
| 3 | IR22 | 15 | BPH | 9 | 0.00 | 0.00 | 0.00 | 0.00 | 1.00 | 0.00 | 0.00 | 0.00 |  |  | 0.18 |
| 4 | IR22 | 15 | BPH | 9 | 0.34 | 0.66 | 0.00 | 0.00 | 0.00 | 0.00 | 0.00 | 0.00 |  |  | 0.23 |
| 1 | IR22 | 15 | BPH | 10 | 0.00 | 0.00 | 0.00 | 0.00 | 1.00 | 0.00 | 0.00 | 0.00 |  |  | 0.22 |
| 2 | IR22 | 15 | BPH | 10 | 0.00 | 0.00 | 0.00 | 0.07 | 0.93 | 0.00 | 0.00 | 0.00 |  |  | 0.17 |
| 3 | IR22 | 15 | BPH | 10 | 0.00 | 0.00 | 0.00 | 0.00 | 1.00 | 0.00 | 0.00 | 0.00 |  |  | 0.15 |
| 4 | IR22 | 15 | BPH | 10 | 0.00 | 1.00 | 0.00 | 0.00 | 0.00 | 0.00 | 0.00 | 0.00 |  |  | 0.32 |
| 1 | IR22 | 15 | BPH | 11 | 0.00 | 0.00 | 0.00 | 0.00 | 1.00 | 0.00 | 0.00 | 0.00 |  |  | 0.18 |
| 2 | IR22 | 15 | BPH | 11 | 0.00 | 0.00 | 0.00 | 0.00 | 1.00 | 0.00 | 0.00 | 0.00 |  |  | 0.16 |
| 3 | IR22 | 15 | BPH | 11 | 0.00 | 0.00 | 0.00 | 0.00 | 1.00 | 0.00 | 0.00 | 0.00 |  |  | 0.17 |
| 4 | IR22 | 15 | BPH | 11 | 0.09 | 0.91 | 0.00 | 0.00 | 0.00 | 0.00 | 0.00 | 0.00 |  |  | 0.31 |
| 1 | IR22 | 15 | BPH | 12 | 0.00 | 0.00 | 0.00 | 0.07 | 0.93 | 0.00 | 0.00 | 0.00 |  |  | 0.16 |
| 2 | IR22 | 15 | BPH | 12 | 0.00 | 0.00 | 0.00 | 0.00 | 1.00 | 0.00 | 0.00 | 0.00 |  |  | 0.19 |
| 3 | IR22 | 15 | BPH | 12 | 0.00 | 0.00 | 0.00 | 0.00 | 1.00 | 0.00 | 0.00 | 0.00 |  |  | 0.07 |
| 4 | IR22 | 15 | BPH | 12 | 0.00 | 0.92 | 0.08 | 0.00 | 0.00 | 0.00 | 0.00 | 0.00 |  |  | 0.43 |
| 1 | IR22 | 15 | BPH | 13 | 0.00 | 0.00 | 0.00 | 0.12 | 0.88 | 0.00 | 0.00 | 0.00 |  |  | 0.23 |
| 2 | IR22 | 15 | BPH | 13 | 0.00 | 0.00 | 0.00 | 0.06 | 0.94 | 0.00 | 0.00 | 0.00 |  |  | 0.17 |
| 3 | IR22 | 15 | BPH | 13 | 0.00 | 0.00 | 0.00 | 0.00 | 1.00 | 0.00 | 0.00 | 0.00 |  |  | 0.20 |
| 4 | IR22 | 15 | BPH | 13 | 0.00 | 0.74 | 0.26 | 0.00 | 0.00 | 0.00 | 0.00 | 0.00 |  |  | 0.45 |
| 1 | IR22 | 15 | BPH | 14 | 0.00 | 0.00 | 0.00 | 0.00 | 1.00 | 0.00 | 0.00 | 0.00 |  |  | 0.25 |
| 2 | IR22 | 15 | BPH | 14 | 0.00 | 0.00 | 0.00 | 0.00 | 1.00 | 0.00 | 0.00 | 0.00 |  |  | 0.13 |
| 3 | IR22 | 15 | BPH | 14 | 0.00 | 0.00 | 0.00 | 0.00 | 1.00 | 0.00 | 0.00 | 0.00 |  |  | 0.18 |
| 4 | IR22 | 15 | BPH | 14 | 0.00 | 0.25 | 0.75 | 0.00 | 0.00 | 0.00 | 0.00 | 0.00 |  |  | 0.54 |
| 1 | IR22 | 15 | BPH | 15 | 0.00 | 0.00 | 0.00 | 0.00 | 1.00 | 0.00 | 0.00 | 0.00 |  |  | 0.28 |
| 2 | IR22 | 15 | BPH | 15 | 0.00 | 0.00 | 0.00 | 0.00 | 1.00 | 0.00 | 0.00 | 0.00 |  |  | 0.18 |
| 3 | IR22 | 15 | BPH | 15 | 0.00 | 0.00 | 0.00 | 0.00 | 1.00 | 0.00 | 0.00 | 0.00 |  |  | 0.25 |
| 4 | IR22 | 15 | BPH | 15 | 0.00 | 0.10 | 0.90 | 0.00 | 0.00 | 0.00 | 0.00 | 0.00 |  |  | 0.58 |
| 1 | IR22 | 15 | BPH | 16 | 0.00 | 0.00 | 0.00 | 0.00 | 1.00 | 0.00 | 0.00 | 0.00 |  |  | 0.49 |
| 2 | IR22 | 15 | BPH | 16 | 0.00 | 0.00 | 0.00 | 0.00 | 1.00 | 0.00 | 0.00 | 0.00 |  |  | 0.22 |
| 3 | IR22 | 15 | BPH | 16 | 0.00 | 0.00 | 0.00 | 0.00 | 1.00 | 0.00 | 0.00 | 0.00 |  |  | 0.23 |
| 4 | IR22 | 15 | BPH | 16 | 0.00 | 0.00 | 0.00 | 0.00 | 1.00 | 0.00 | 0.00 | 0.00 |  |  | 0.31 |
| 1 | IR22 | 15 | BPH | 17 | 0.00 | 0.00 | 0.00 | 0.00 | 1.00 | 0.00 | 0.00 | 0.00 |  |  | 0.43 |
| 2 | IR22 | 15 | BPH | 17 | 0.00 | 0.00 | 0.00 | 0.00 | 1.00 | 0.00 | 0.00 | 0.00 |  |  | 0.18 |
| 3 | IR22 | 15 | BPH | 17 | 0.00 | 0.00 | 0.00 | 0.00 | 1.00 | 0.00 | 0.00 | 0.00 |  |  | 0.13 |
| 4 | IR22 | 15 | BPH | 17 | 0.00 | 0.00 | 0.00 | 0.00 | 1.00 | 0.00 | 0.00 | 0.00 |  |  | 0.25 |
| 1 | IR22 | 15 | BPH | 18 | 0.00 | 0.00 | 0.00 | 0.00 | 1.00 | 0.00 | 0.00 | 0.00 |  |  | 0.52 |
| 2 | IR22 | 15 | BPH | 18 | 0.00 | 0.00 | 0.00 | 0.00 | 1.00 | 0.00 | 0.00 | 0.00 |  |  | 0.18 |
| 3 | IR22 | 15 | BPH | 18 | 0.00 | 0.00 | 0.00 | 0.07 | 0.93 | 0.00 | 0.00 | 0.00 |  |  | 0.30 |
| 4 | IR22 | 15 | BPH | 18 | 0.00 | 0.00 | 0.00 | 0.02 | 0.98 | 0.00 | 0.00 | 0.00 |  |  | 0.33 |
| 1 | IR22 | 15 | BPH | 19 | 0.00 | 0.00 | 0.00 | 0.00 | 1.00 | 0.00 | 0.00 | 0.00 |  |  | 0.41 |
| 2 | IR22 | 15 | BPH | 19 | 0.00 | 0.00 | 0.00 | 0.00 | 1.00 | 0.00 | 0.00 | 0.00 |  |  | 0.22 |
| 3 | IR22 | 15 | BPH | 19 | 0.00 | 0.00 | 0.00 | 0.06 | 0.94 | 0.00 | 0.00 | 0.00 |  |  | 0.30 |
| 4 | IR22 | 15 | BPH | 19 | 0.00 | 0.00 | 0.00 | 0.02 | 0.98 | 0.00 | 0.00 | 0.00 |  |  | 0.31 |
| 1 | IR22 | 15 | BPH | 20 | 0.00 | 0.00 | 0.00 | 0.00 | 1.00 | 0.00 | 0.00 | 0.00 |  |  | 0.76 |
| 2 | IR22 | 15 | BPH | 20 | 0.00 | 0.00 | 0.00 | 0.00 | 1.00 | 0.00 | 0.00 | 0.00 |  |  | 0.13 |
| 3 | IR22 | 15 | BPH | 20 | 0.00 | 0.00 | 0.00 | 0.00 | 1.00 | 0.00 | 0.00 | 0.00 |  |  | 0.17 |
| 4 | IR22 | 15 | BPH | 20 | 0.00 | 0.00 | 0.00 | 0.00 | 1.00 | 0.00 | 0.00 | 0.00 |  |  | 0.36 |
| 1 | IR22 | 15 | BPH | 21 | 0.00 | 0.00 | 0.00 | 0.00 | 1.00 | 0.00 | 0.00 | 0.00 |  |  | 0.54 |
| 2 | IR22 | 15 | BPH | 21 | 0.00 | 0.00 | 0.00 | 0.00 | 1.00 | 0.00 | 0.00 | 0.00 |  |  | 0.24 |
| 3 | IR22 | 15 | BPH | 21 | 0.00 | 0.00 | 0.00 | 0.00 | 1.00 | 0.00 | 0.00 | 0.00 |  |  | 0.31 |
| 4 | IR22 | 15 | BPH | 21 | 0.00 | 0.00 | 0.00 | 0.00 | 1.00 | 0.00 | 0.00 | 0.00 |  |  | 0.36 |
| 1 | IR22 | 15 | BPH | 22 | 0.00 | 0.00 | 0.00 | 0.00 | 1.00 | 0.00 | 0.00 | 0.00 |  |  | 0.57 |
| 2 | IR22 | 15 | BPH | 22 | 0.00 | 0.00 | 0.00 | 0.00 | 1.00 | 0.00 | 0.00 | 0.00 |  |  | 0.19 |
| 3 | IR22 | 15 | BPH | 22 | 0.00 | 0.00 | 0.00 | 0.00 | 1.00 | 0.00 | 0.00 | 0.00 |  |  | 0.16 |
| 4 | IR22 | 15 | BPH | 22 | 0.00 | 0.00 | 0.00 | 0.00 | 1.00 | 0.00 | 0.00 | 0.00 |  |  | 0.31 |
| 1 | IR22 | 15 | BPH | 23 | 0.00 | 0.00 | 0.00 | 0.00 | 1.00 | 0.00 | 0.00 | 0.00 |  |  | 0.63 |
| 2 | IR22 | 15 | BPH | 23 | 0.00 | 0.00 | 0.00 | 0.00 | 1.00 | 0.00 | 0.00 | 0.00 |  |  | 0.29 |
| 3 | IR22 | 15 | BPH | 23 | 0.00 | 0.00 | 0.00 | 0.00 | 1.00 | 0.00 | 0.00 | 0.00 |  |  | 0.27 |
| 4 | IR22 | 15 | BPH | 23 | 0.00 | 0.00 | 0.00 | 0.00 | 1.00 | 0.00 | 0.00 | 0.00 |  |  | 0.40 |
| 1 | IR22 | 15 | BPH | 24 | 0.00 | 0.00 | 0.00 | 0.00 | 1.00 | 0.00 | 0.00 | 0.00 |  |  | 0.62 |
| 2 | IR22 | 15 | BPH | 24 | 0.00 | 0.00 | 0.00 | 0.00 | 1.00 | 0.00 | 0.00 | 0.00 |  |  | 0.34 |
| 3 | IR22 | 15 | BPH | 24 | 0.00 | 0.00 | 0.00 | 0.00 | 1.00 | 0.00 | 0.00 | 0.00 |  |  | 0.31 |
| 4 | IR22 | 15 | BPH | 24 | 0.00 | 0.00 | 0.00 | 0.00 | 1.00 | 0.00 | 0.00 | 0.00 |  |  | 0.42 |
| 1 | IR22 | 15 | BPH | 25 | 0.00 | 0.00 | 0.00 | 0.00 | 1.00 | 0.00 | 0.00 | 0.00 |  |  | 0.42 |
| 2 | IR22 | 15 | BPH | 25 | 0.00 | 0.00 | 0.00 | 0.00 | 1.00 | 0.00 | 0.00 | 0.00 |  |  | 0.31 |
| 3 | IR22 | 15 | BPH | 25 | 0.00 | 0.00 | 0.00 | 0.00 | 1.00 | 0.00 | 0.00 | 0.00 |  |  | 0.33 |
| 4 | IR22 | 15 | BPH | 25 | 0.00 | 0.00 | 0.00 | 0.00 | 1.00 | 0.00 | 0.00 | 0.00 |  |  | 0.35 |
| 1 | IR22 | 15 | BPH | 26 | 0.00 | 0.00 | 0.00 | 0.00 | 1.00 | 0.00 | 0.00 | 0.00 |  |  | 0.87 |
| 2 | IR22 | 15 | BPH | 26 | 0.00 | 0.00 | 0.00 | 0.00 | 1.00 | 0.00 | 0.00 | 0.00 |  |  | 0.30 |
| 3 | IR22 | 15 | BPH | 26 | 0.00 | 0.00 | 0.00 | 0.00 | 1.00 | 0.00 | 0.00 | 0.00 |  |  | 0.40 |
| 4 | IR22 | 15 | BPH | 26 | 0.00 | 0.00 | 0.00 | 0.00 | 1.00 | 0.00 | 0.00 | 0.00 |  |  | 0.53 |
| 1 | IR22 | 15 | BPH | 27 | 0.00 | 0.00 | 0.00 | 0.00 | 1.00 | 0.00 | 0.00 | 0.00 |  |  | 0.23 |
| 2 | IR22 | 15 | BPH | 27 | 0.00 | 0.00 | 0.00 | 0.00 | 1.00 | 0.00 | 0.00 | 0.00 |  |  | 0.17 |
| 3 | IR22 | 15 | BPH | 27 | 0.00 | 0.00 | 0.00 | 0.00 | 1.00 | 0.00 | 0.00 | 0.00 |  |  | 0.43 |
| 4 | IR22 | 15 | BPH | 27 | 0.00 | 0.00 | 0.00 | 0.00 | 1.00 | 0.00 | 0.00 | 0.00 |  |  | 0.28 |
| 1 | IR22 | 15 | BPH | 28 | 0.00 | 0.00 | 0.00 | 0.00 | 1.00 | 0.00 | 0.00 | 0.00 |  |  | 0.72 |
| 2 | IR22 | 15 | BPH | 28 | 0.00 | 0.00 | 0.00 | 0.00 | 1.00 | 0.00 | 0.00 | 0.00 |  |  | 0.30 |
| 3 | IR22 | 15 | BPH | 28 | 0.00 | 0.00 | 0.00 | 0.00 | 1.00 | 0.00 | 0.00 | 0.00 |  |  | 0.47 |
| 4 | IR22 | 15 | BPH | 28 | 0.00 | 0.00 | 0.00 | 0.00 | 1.00 | 0.00 | 0.00 | 0.00 |  |  | 0.49 |
| 1 | IR22 | 15 | BPH | 29 | 0.00 | 0.00 | 0.00 | 0.00 | 1.00 | 0.00 | 0.00 | 0.00 |  |  | 1.25 |
| 2 | IR22 | 15 | BPH | 29 | 0.00 | 0.00 | 0.00 | 0.00 | 1.00 | 0.00 | 0.00 | 0.00 |  |  | 0.26 |
| 3 | IR22 | 15 | BPH | 29 | 0.00 | 0.00 | 0.00 | 0.00 | 1.00 | 0.00 | 0.00 | 0.00 |  |  | 0.51 |
| 4 | IR22 | 15 | BPH | 29 | 0.00 | 0.00 | 0.00 | 0.00 | 1.00 | 0.00 | 0.00 | 0.00 |  |  | 0.67 |
| 1 | IR22 | 15 | BPH | 30 | 0.00 | 0.00 | 0.00 | 0.00 | 1.00 | 0.00 | 0.00 | 0.00 |  |  | 0.91 |
| 2 | IR22 | 15 | BPH | 30 | 0.00 | 0.00 | 0.00 | 0.00 | 1.00 | 0.00 | 0.00 | 0.00 |  |  | 0.44 |
| 3 | IR22 | 15 | BPH | 30 | 0.00 | 0.00 | 0.00 | 0.00 | 1.00 | 0.00 | 0.00 | 0.00 |  |  | 0.70 |
| 4 | IR22 | 15 | BPH | 30 | 0.00 | 0.00 | 0.00 | 0.00 | 1.00 | 0.00 | 0.00 | 0.00 |  |  | 0.68 |
| 4 | IR22 | 20 | BPH | 1 | 1.00 | 0.00 | 0.00 | 0.00 | 0.00 | 0.00 | 0.00 | 0.00 |  |  | 0.06 |
| 4 | IR22 | 20 | BPH | 1 | 1.00 | 0.00 | 0.00 | 0.00 | 0.00 | 0.00 | 0.00 | 0.00 |  |  | 0.07 |
| 4 | IR22 | 20 | BPH | 1 | 1.00 | 0.00 | 0.00 | 0.00 | 0.00 | 0.00 | 0.00 | 0.00 |  |  | 0.07 |
| 4 | IR22 | 20 | BPH | 1 | 1.00 | 0.00 | 0.00 | 0.00 | 0.00 | 0.00 | 0.00 | 0.00 |  |  | 0.07 |
| 4 | IR22 | 20 | BPH | 2 | 1.00 | 0.00 | 0.00 | 0.00 | 0.00 | 0.00 | 0.00 | 0.00 |  |  | 0.13 |
| 4 | IR22 | 20 | BPH | 2 | 1.00 | 0.00 | 0.00 | 0.00 | 0.00 | 0.00 | 0.00 | 0.00 |  |  | 0.12 |
| 4 | IR22 | 20 | BPH | 2 | 1.00 | 0.00 | 0.00 | 0.00 | 0.00 | 0.00 | 0.00 | 0.00 |  |  | 0.12 |
| 4 | IR22 | 20 | BPH | 2 | 1.00 | 0.00 | 0.00 | 0.00 | 0.00 | 0.00 | 0.00 | 0.00 |  |  | 0.14 |
| 4 | IR22 | 20 | BPH | 3 | 1.00 | 0.00 | 0.00 | 0.00 | 0.00 | 0.00 | 0.00 | 0.00 |  |  | 0.21 |
| 4 | IR22 | 20 | BPH | 3 | 1.00 | 0.00 | 0.00 | 0.00 | 0.00 | 0.00 | 0.00 | 0.00 |  |  | 0.21 |
| 4 | IR22 | 20 | BPH | 3 | 1.00 | 0.00 | 0.00 | 0.00 | 0.00 | 0.00 | 0.00 | 0.00 |  |  | 0.20 |
| 4 | IR22 | 20 | BPH | 3 | 1.00 | 0.00 | 0.00 | 0.00 | 0.00 | 0.00 | 0.00 | 0.00 |  |  | 0.21 |
| 4 | IR22 | 20 | BPH | 4 | 0.00 | 1.00 | 0.00 | 0.00 | 0.00 | 0.00 | 0.00 | 0.00 |  |  | 0.19 |
| 4 | IR22 | 20 | BPH | 4 | 0.05 | 0.95 | 0.00 | 0.00 | 0.00 | 0.00 | 0.00 | 0.00 |  |  | 0.19 |
| 4 | IR22 | 20 | BPH | 4 | 0.25 | 0.75 | 0.00 | 0.00 | 0.00 | 0.00 | 0.00 | 0.00 |  |  | 0.13 |
| 4 | IR22 | 20 | BPH | 4 | 0.44 | 0.56 | 0.00 | 0.00 | 0.00 | 0.00 | 0.00 | 0.00 |  |  | 0.18 |
| 4 | IR22 | 20 | BPH | 5 | 0.11 | 0.89 | 0.00 | 0.00 | 0.00 | 0.00 | 0.00 | 0.00 |  |  | 0.20 |
| 4 | IR22 | 20 | BPH | 5 | 0.06 | 0.94 | 0.00 | 0.00 | 0.00 | 0.00 | 0.00 | 0.00 |  |  | 0.19 |
| 4 | IR22 | 20 | BPH | 5 | 0.11 | 0.89 | 0.00 | 0.00 | 0.00 | 0.00 | 0.00 | 0.00 |  |  | 0.20 |
| 4 | IR22 | 20 | BPH | 5 | 0.13 | 0.88 | 0.00 | 0.00 | 0.00 | 0.00 | 0.00 | 0.00 |  |  | 0.17 |
| 4 | IR22 | 20 | BPH | 6 | 0.00 | 1.00 | 0.00 | 0.00 | 0.00 | 0.00 | 0.00 | 0.00 |  |  | 0.31 |
| 4 | IR22 | 20 | BPH | 6 | 0.00 | 1.00 | 0.00 | 0.00 | 0.00 | 0.00 | 0.00 | 0.00 |  |  | 0.25 |
| 4 | IR22 | 20 | BPH | 6 | 0.00 | 1.00 | 0.00 | 0.00 | 0.00 | 0.00 | 0.00 | 0.00 |  |  | 0.33 |
| 4 | IR22 | 20 | BPH | 6 | 0.00 | 1.00 | 0.00 | 0.00 | 0.00 | 0.00 | 0.00 | 0.00 |  |  | 0.16 |
| 4 | IR22 | 20 | BPH | 7 | 0.00 | 1.00 | 0.00 | 0.00 | 0.00 | 0.00 | 0.00 | 0.00 |  |  | 0.45 |
| 4 | IR22 | 20 | BPH | 7 | 0.00 | 1.00 | 0.00 | 0.00 | 0.00 | 0.00 | 0.00 | 0.00 |  |  | 0.50 |
| 4 | IR22 | 20 | BPH | 7 | 0.00 | 1.00 | 0.00 | 0.00 | 0.00 | 0.00 | 0.00 | 0.00 |  |  | 0.44 |
| 4 | IR22 | 20 | BPH | 7 | 0.00 | 1.00 | 0.00 | 0.00 | 0.00 | 0.00 | 0.00 | 0.00 |  |  | 0.42 |
| 4 | IR22 | 20 | BPH | 8 | 0.00 | 0.84 | 0.00 | 0.00 | 0.00 | 0.00 | 0.00 | 0.00 |  |  | 0.45 |
| 4 | IR22 | 20 | BPH | 8 | 0.00 | 0.67 | 0.00 | 0.00 | 0.00 | 0.00 | 0.00 | 0.00 |  |  | 0.42 |
| 4 | IR22 | 20 | BPH | 8 | 0.00 | 0.90 | 0.00 | 0.00 | 0.00 | 0.00 | 0.00 | 0.00 |  |  | 0.53 |
| 4 | IR22 | 20 | BPH | 8 | 0.00 | 1.00 | 0.00 | 0.00 | 0.00 | 0.00 | 0.00 | 0.00 |  |  | 0.47 |
| 4 | IR22 | 20 | BPH | 9 | 0.00 | 0.00 | 1.00 | 0.00 | 0.00 | 0.00 | 0.00 | 0.00 |  |  | 0.95 |
| 4 | IR22 | 20 | BPH | 9 | 0.00 | 0.06 | 0.94 | 0.00 | 0.00 | 0.00 | 0.00 | 0.00 |  |  | 0.72 |
| 4 | IR22 | 20 | BPH | 9 | 0.00 | 0.22 | 0.78 | 0.00 | 0.00 | 0.00 | 0.00 | 0.00 |  |  | 0.45 |
| 4 | IR22 | 20 | BPH | 9 | 0.00 | 0.29 | 0.71 | 0.00 | 0.00 | 0.00 | 0.00 | 0.00 |  |  | 0.34 |
| 4 | IR22 | 20 | BPH | 10 | 0.00 | 0.00 | 0.85 | 0.15 | 0.00 | 0.00 | 0.00 | 0.00 |  |  | 1.23 |
| 4 | IR22 | 20 | BPH | 10 | 0.00 | 0.00 | 0.71 | 0.59 | 0.00 | 0.00 | 0.00 | 0.00 |  |  | 1.00 |
| 4 | IR22 | 20 | BPH | 10 | 0.00 | 0.00 | 0.30 | 0.70 | 0.00 | 0.00 | 0.00 | 0.00 |  |  | 1.10 |
| 4 | IR22 | 20 | BPH | 10 | 0.00 | 0.00 | 1.00 | 0.00 | 0.00 | 0.00 | 0.00 | 0.00 |  |  | 0.99 |
| 4 | IR22 | 20 | BPH | 11 | 0.00 | 0.00 | 0.83 | 0.17 | 0.00 | 0.00 | 0.00 | 0.00 |  |  | 1.05 |
| 4 | IR22 | 20 | BPH | 11 | 0.00 | 0.00 | 0.80 | 0.20 | 0.00 | 0.00 | 0.00 | 0.00 |  |  | 1.24 |
| 4 | IR22 | 20 | BPH | 11 | 0.00 | 0.00 | 0.75 | 0.25 | 0.00 | 0.00 | 0.00 | 0.00 |  |  | 0.98 |
| 4 | IR22 | 20 | BPH | 11 | 0.00 | 0.00 | 0.80 | 0.20 | 0.00 | 0.00 | 0.00 | 0.00 |  |  | 1.20 |
| 4 | IR22 | 20 | BPH | 12 | 0.00 | 0.00 | 0.20 | 0.80 | 0.00 | 0.00 | 0.00 | 0.00 |  |  | 0.90 |
| 4 | IR22 | 20 | BPH | 12 | 0.00 | 0.00 | 0.22 | 0.78 | 0.00 | 0.00 | 0.00 | 0.00 |  |  | 1.16 |
| 4 | IR22 | 20 | BPH | 12 | 0.00 | 0.00 | 0.00 | 1.00 | 0.00 | 0.00 | 0.00 | 0.00 |  |  | 1.25 |
| 4 | IR22 | 20 | BPH | 12 | 0.00 | 0.00 | 0.11 | 0.89 | 0.00 | 0.00 | 0.00 | 0.00 |  |  | 1.11 |
| 4 | IR22 | 20 | BPH | 13 | 0.00 | 0.00 | 0.15 | 0.85 | 0.00 | 0.00 | 0.00 | 0.00 |  |  | 1.64 |
| 4 | IR22 | 20 | BPH | 13 | 0.00 | 0.00 | 0.29 | 0.71 | 0.00 | 0.00 | 0.00 | 0.00 |  |  | 1.39 |
| 4 | IR22 | 20 | BPH | 13 | 0.00 | 0.00 | 0.00 | 1.00 | 0.00 | 0.00 | 0.00 | 0.00 |  |  | 2.34 |
| 4 | IR22 | 20 | BPH | 13 | 0.00 | 0.00 | 0.00 | 1.00 | 0.00 | 0.00 | 0.00 | 0.00 |  |  | 1.92 |
| 4 | IR22 | 20 | BPH | 14 | 0.00 | 0.00 | 0.14 | 0.86 | 0.00 | 0.00 | 0.00 | 0.00 |  |  | 1.68 |
| 4 | IR22 | 20 | BPH | 14 | 0.00 | 0.00 | 0.00 | 0.95 | 0.05 | 0.00 | 0.00 | 0.00 |  |  | 2.64 |
| 4 | IR22 | 20 | BPH | 14 | 0.00 | 0.00 | 0.00 | 1.00 | 0.00 | 0.00 | 0.00 | 0.00 |  |  | 1.93 |
| 4 | IR22 | 20 | BPH | 14 | 0.00 | 0.00 | 0.11 | 0.89 | 0.00 | 0.00 | 0.00 | 0.00 |  |  | 1.87 |
| 4 | IR22 | 20 | BPH | 15 | 0.00 | 0.00 | 0.00 | 0.70 | 0.30 | 0.00 | 0.00 | 0.00 |  |  | 2.94 |
| 4 | IR22 | 20 | BPH | 15 | 0.00 | 0.00 | 0.00 | 0.95 | 0.05 | 0.00 | 0.00 | 0.00 |  |  | 2.91 |
| 4 | IR22 | 20 | BPH | 15 | 0.00 | 0.00 | 0.00 | 1.00 | 0.00 | 0.00 | 0.00 | 0.00 |  |  | 2.29 |
| 4 | IR22 | 20 | BPH | 15 | 0.00 | 0.00 | 0.00 | 0.70 | 0.30 | 0.00 | 0.00 | 0.00 |  |  | 2.89 |
| 1 | IR22 | 25 | BPH | 1 | 0.33 | 0.44 | 0.22 | 0.00 | 0.00 | 0.00 | 0.00 | 0.00 |  |  | 0.14 |
| 2 | IR22 | 25 | BPH | 1 | 0.95 | 0.05 | 0.00 | 0.00 | 0.00 | 0.00 | 0.00 | 0.00 |  |  | 0.12 |
| 3 | IR22 | 25 | BPH | 1 | 0.86 | 0.14 | 0.00 | 0.00 | 0.00 | 0.00 | 0.00 | 0.00 |  |  | 0.10 |
| 4 | IR22 | 25 | BPH | 1 | 0.71 | 0.21 | 0.07 | 0.00 | 0.00 | 0.00 | 0.00 | 0.00 |  |  | 0.12 |
| 1 | IR22 | 25 | BPH | 2 | 0.00 | 0.28 | 0.44 | 0.28 | 0.00 | 0.00 | 0.00 | 0.00 |  |  | 0.18 |
| 2 | IR22 | 25 | BPH | 2 | 0.00 | 0.65 | 0.35 | 0.00 | 0.00 | 0.00 | 0.00 | 0.00 |  |  | 0.18 |
| 3 | IR22 | 25 | BPH | 2 | 0.00 | 0.00 | 1.00 | 0.00 | 0.00 | 0.00 | 0.00 | 0.00 |  |  | 0.15 |
| 4 | IR22 | 25 | BPH | 2 | 0.00 | 0.31 | 0.60 | 0.09 | 0.00 | 0.00 | 0.00 | 0.00 |  |  | 0.17 |
| 1 | IR22 | 25 | BPH | 3 | 0.00 | 0.15 | 0.85 | 0.00 | 0.00 | 0.00 | 0.00 | 0.00 |  |  | 0.23 |
| 2 | IR22 | 25 | BPH | 3 | 0.00 | 0.45 | 0.55 | 0.00 | 0.00 | 0.00 | 0.00 | 0.00 |  |  | 0.21 |
| 3 | IR22 | 25 | BPH | 3 | 0.00 | 0.06 | 0.76 | 0.18 | 0.00 | 0.00 | 0.00 | 0.00 |  |  | 0.17 |
| 4 | IR22 | 25 | BPH | 3 | 0.00 | 0.22 | 0.72 | 0.06 | 0.00 | 0.00 | 0.00 | 0.00 |  |  | 0.20 |
| 1 | IR22 | 25 | BPH | 4 | 0.00 | 0.00 | 0.35 | 0.65 | 0.00 | 0.00 | 0.00 | 0.00 |  |  | 0.28 |
| 2 | IR22 | 25 | BPH | 4 | 0.00 | 0.00 | 0.47 | 0.53 | 0.00 | 0.00 | 0.00 | 0.00 |  |  | 0.38 |
| 3 | IR22 | 25 | BPH | 4 | 0.00 | 0.00 | 0.15 | 0.75 | 0.10 | 0.00 | 0.00 | 0.00 |  |  | 0.38 |
| 4 | IR22 | 25 | BPH | 4 | 0.00 | 0.00 | 0.32 | 0.64 | 0.03 | 0.00 | 0.00 | 0.00 |  |  | 0.35 |
| 1 | IR22 | 25 | BPH | 5 | 0.00 | 0.00 | 0.05 | 0.90 | 0.05 | 0.00 | 0.00 | 0.00 |  |  | 0.51 |
| 2 | IR22 | 25 | BPH | 5 | 0.00 | 0.00 | 0.00 | 0.13 | 0.88 | 0.00 | 0.00 | 0.00 |  |  | 0.40 |
| 3 | IR22 | 25 | BPH | 5 | 0.00 | 0.00 | 0.00 | 0.42 | 0.58 | 0.00 | 0.00 | 0.00 |  |  | 0.44 |
| 4 | IR22 | 25 | BPH | 5 | 0.00 | 0.00 | 0.02 | 0.48 | 0.50 | 0.00 | 0.00 | 0.00 |  |  | 0.45 |
| 1 | IR22 | 25 | BPH | 6 | 0.00 | 0.00 | 0.00 | 0.33 | 0.67 | 0.00 | 0.00 | 0.00 |  |  | 0.68 |
| 2 | IR22 | 25 | BPH | 6 | 0.00 | 0.00 | 0.00 | 0.00 | 1.00 | 0.00 | 0.00 | 0.00 |  |  | 0.34 |
| 3 | IR22 | 25 | BPH | 6 | 0.00 | 0.00 | 0.00 | 0.00 | 1.00 | 0.00 | 0.00 | 0.00 |  |  | 0.52 |
| 4 | IR22 | 25 | BPH | 6 | 0.00 | 0.00 | 0.00 | 0.11 | 0.89 | 0.00 | 0.00 | 0.00 |  |  | 0.51 |
| 1 | IR22 | 25 | BPH | 7 | 0.00 | 0.00 | 0.00 | 0.00 | 1.00 | 0.00 | 0.00 | 0.00 |  |  | 1.14 |
| 2 | IR22 | 25 | BPH | 7 | 0.00 | 0.00 | 0.00 | 0.00 | 1.00 | 0.00 | 0.00 | 0.00 |  |  | 0.96 |
| 3 | IR22 | 25 | BPH | 7 | 0.00 | 0.00 | 0.00 | 0.00 | 1.00 | 0.00 | 0.00 | 0.00 |  |  | 1.08 |
| 4 | IR22 | 25 | BPH | 7 | 0.00 | 0.00 | 0.00 | 0.00 | 1.00 | 0.00 | 0.00 | 0.00 |  |  | 1.06 |
| 1 | IR22 | 25 | BPH | 8 | 0.00 | 0.00 | 0.00 | 0.00 | 1.00 | 0.00 | 0.00 | 0.00 |  |  | 1.59 |
| 2 | IR22 | 25 | BPH | 8 | 0.00 | 0.00 | 0.00 | 0.00 | 1.00 | 0.00 | 0.00 | 0.00 |  |  | 1.15 |
| 3 | IR22 | 25 | BPH | 8 | 0.00 | 0.00 | 0.00 | 0.05 | 0.95 | 0.00 | 0.00 | 0.00 |  |  | 1.12 |
| 4 | IR22 | 25 | BPH | 8 | 0.00 | 0.00 | 0.00 | 0.02 | 0.98 | 0.00 | 0.00 | 0.00 |  |  | 1.29 |
| 1 | IR22 | 25 | BPH | 9 | 0.00 | 0.00 | 0.00 | 0.00 | 1.00 | 0.00 | 0.00 | 0.00 |  |  | 2.10 |
| 2 | IR22 | 25 | BPH | 9 | 0.00 | 0.00 | 0.00 | 0.00 | 1.00 | 0.00 | 0.00 | 0.00 |  |  | 2.15 |
| 3 | IR22 | 25 | BPH | 9 | 0.00 | 0.00 | 0.00 | 0.00 | 1.00 | 0.00 | 0.00 | 0.00 |  |  | 1.78 |
| 4 | IR22 | 25 | BPH | 9 | 0.00 | 0.00 | 0.00 | 0.00 | 1.00 | 0.00 | 0.00 | 0.00 |  |  | 2.01 |
| 1 | IR22 | 25 | BPH | 10 | 0.00 | 0.00 | 0.00 | 0.00 | 1.00 | 0.00 | 0.00 | 0.00 |  |  | 2.58 |
| 2 | IR22 | 25 | BPH | 10 | 0.00 | 0.00 | 0.00 | 0.00 | 1.00 | 0.00 | 0.00 | 0.00 |  |  | 2.46 |
| 3 | IR22 | 25 | BPH | 10 | 0.00 | 0.00 | 0.00 | 0.00 | 1.00 | 0.00 | 0.00 | 0.00 |  |  | 1.97 |
| 4 | IR22 | 25 | BPH | 10 | 0.00 | 0.00 | 0.00 | 0.00 | 1.00 | 0.00 | 0.00 | 0.00 |  |  | 2.33 |
| 1 | IR22 | 25 | BPH | 11 | 0.00 | 0.00 | 0.00 | 0.00 | 1.00 | 0.00 | 0.00 | 0.00 |  |  | 2.78 |
| 2 | IR22 | 25 | BPH | 11 | 0.00 | 0.00 | 0.00 | 0.00 | 1.00 | 0.00 | 0.00 | 0.00 |  |  | 3.11 |
| 3 | IR22 | 25 | BPH | 11 | 0.00 | 0.00 | 0.00 | 0.00 | 1.00 | 0.00 | 0.00 | 0.00 |  |  | 3.10 |
| 4 | IR22 | 25 | BPH | 11 | 0.00 | 0.00 | 0.00 | 0.00 | 1.00 | 0.00 | 0.00 | 0.00 |  |  | 3.00 |
| 1 | IR22 | 25 | BPH | 12 | 0.00 | 0.00 | 0.00 | 0.00 | 1.00 | 0.00 | 0.00 | 0.00 |  |  | 4.02 |
| 2 | IR22 | 25 | BPH | 12 | 0.00 | 0.00 | 0.00 | 0.00 | 1.00 | 0.00 | 0.00 | 0.00 |  |  | 3.91 |
| 3 | IR22 | 25 | BPH | 12 | 0.00 | 0.00 | 0.00 | 0.00 | 0.89 | 0.11 | 1.00 | 0.00 | 1.00 |  | 4.54 |
| 4 | IR22 | 25 | BPH | 12 | 0.00 | 0.00 | 0.00 | 0.00 | 0.96 | 0.04 | 0.33 | 0.00 | 1.00 |  | 4.15 |
| 1 | IR22 | 25 | BPH | 13 | 0.00 | 0.00 | 0.00 | 0.00 | 0.95 | 0.05 | 0.50 | 0.00 | 1.00 |  | 5.30 |
| 2 | IR22 | 25 | BPH | 13 | 0.00 | 0.00 | 0.00 | 0.00 | 0.88 | 0.12 | 1.00 | 0.00 | 1.00 |  | 5.46 |
| 3 | IR22 | 25 | BPH | 13 | 0.00 | 0.00 | 0.00 | 0.00 | 0.85 | 0.15 | 1.50 | 0.00 | 1.00 |  | 5.22 |
| 4 | IR22 | 25 | BPH | 13 | 0.00 | 0.00 | 0.00 | 0.00 | 0.89 | 0.11 | 1.00 | 0.00 | 1.00 |  | 5.33 |
| 1 | IR22 | 25 | BPH | 14 | 0.00 | 0.00 | 0.00 | 0.00 | 0.68 | 0.32 | 1.00 | 2.00 | 1.00 | 0.50 | 6.13 |
| 2 | IR22 | 25 | BPH | 14 | 0.00 | 0.00 | 0.00 | 0.00 | 0.47 | 0.53 | 3.00 | 1.50 | 1.00 | 0.67 | 5.72 |
| 3 | IR22 | 25 | BPH | 14 | 0.00 | 0.00 | 0.00 | 0.00 | 0.18 | 0.82 | 3.50 | 1.00 | 1.00 | 0.50 | 3.93 |
| 4 | IR22 | 25 | BPH | 14 | 0.00 | 0.00 | 0.00 | 0.00 | 0.45 | 0.55 | 2.50 | 1.50 | 1.00 | 0.56 | 5.26 |
| 1 | IR22 | 25 | BPH | 15 | 0.00 | 0.00 | 0.00 | 0.00 | 0.10 | 0.90 | 6.00 | 3.00 | 0.50 | 0.33 | 6.90 |
| 2 | IR22 | 25 | BPH | 15 | 0.00 | 0.00 | 0.00 | 0.00 | 0.22 | 0.78 | 2.00 | 5.00 | 1.00 | 0.30 | 5.67 |
| 3 | IR22 | 25 | BPH | 15 | 0.00 | 0.00 | 0.00 | 0.00 | 0.05 | 0.95 | 4.00 | 5.00 | 1.00 | 0.60 | 5.99 |
| 4 | IR22 | 25 | BPH | 15 | 0.00 | 0.00 | 0.00 | 0.00 | 0.12 | 0.88 | 4.00 | 4.33 | 0.83 | 0.41 | 6.18 |
| 1 | IR22 | 30 | BPH | 1 | 0.35 | 0.45 | 0.20 | 0.00 | 0.00 | 0.00 | 0.00 | 0.00 |  |  | 0.17 |
| 2 | IR22 | 30 | BPH | 1 | 0.74 | 0.26 | 0.00 | 0.00 | 0.00 | 0.00 | 0.00 | 0.00 |  |  | 0.14 |
| 3 | IR22 | 30 | BPH | 1 | 0.77 | 0.23 | 0.00 | 0.00 | 0.00 | 0.00 | 0.00 | 0.00 |  |  | 0.10 |
| 4 | IR22 | 30 | BPH | 1 | 0.62 | 0.31 | 0.07 | 0.00 | 0.00 | 0.00 | 0.00 | 0.00 |  |  | 0.14 |
| 1 | IR22 | 30 | BPH | 2 | 0.05 | 0.65 | 0.30 | 0.00 | 0.00 | 0.00 | 0.00 | 0.00 |  |  | 0.24 |
| 2 | IR22 | 30 | BPH | 2 | 0.00 | 0.05 | 0.95 | 0.00 | 0.00 | 0.00 | 0.00 | 0.00 |  |  | 0.21 |
| 3 | IR22 | 30 | BPH | 2 | 0.00 | 0.00 | 0.92 | 0.08 | 0.00 | 0.00 | 0.00 | 0.00 |  |  | 0.13 |
| 4 | IR22 | 30 | BPH | 2 | 0.02 | 0.23 | 0.72 | 0.03 | 0.00 | 0.00 | 0.00 | 0.00 |  |  | 0.19 |
| 1 | IR22 | 30 | BPH | 3 | 0.00 | 0.00 | 0.26 | 0.74 | 0.00 | 0.00 | 0.00 | 0.00 |  |  | 0.41 |
| 2 | IR22 | 30 | BPH | 3 | 0.00 | 0.00 | 0.42 | 0.53 | 0.05 | 0.00 | 0.00 | 0.00 |  |  | 0.31 |
| 3 | IR22 | 30 | BPH | 3 | 0.00 | 0.00 | 0.22 | 0.78 | 0.00 | 0.00 | 0.00 | 0.00 |  |  | 0.28 |
| 4 | IR22 | 30 | BPH | 3 | 0.00 | 0.00 | 0.30 | 0.68 | 0.02 | 0.00 | 0.00 | 0.00 |  |  | 0.33 |
| 1 | IR22 | 30 | BPH | 4 | 0.00 | 0.00 | 0.00 | 0.65 | 0.35 | 0.00 | 0.00 | 0.00 |  |  | 0.47 |
| 2 | IR22 | 30 | BPH | 4 | 0.00 | 0.00 | 0.00 | 0.40 | 0.60 | 0.00 | 0.00 | 0.00 |  |  | 0.47 |
| 3 | IR22 | 30 | BPH | 4 | 0.00 | 0.00 | 0.00 | 0.75 | 0.25 | 0.00 | 0.00 | 0.00 |  |  | 0.30 |
| 4 | IR22 | 30 | BPH | 4 | 0.00 | 0.00 | 0.00 | 0.60 | 0.40 | 0.00 | 0.00 | 0.00 |  |  | 0.41 |
| 1 | IR22 | 30 | BPH | 5 | 0.00 | 0.00 | 0.00 | 0.37 | 0.63 | 0.00 | 0.00 | 0.00 |  |  | 0.58 |
| 2 | IR22 | 30 | BPH | 5 | 0.00 | 0.00 | 0.00 | 0.00 | 1.00 | 0.00 | 0.00 | 0.00 |  |  | 0.59 |
| 3 | IR22 | 30 | BPH | 5 | 0.00 | 0.00 | 0.00 | 0.11 | 0.89 | 0.00 | 0.00 | 0.00 |  |  | 0.55 |
| 4 | IR22 | 30 | BPH | 5 | 0.00 | 0.00 | 0.00 | 0.16 | 0.84 | 0.00 | 0.00 | 0.00 |  |  | 0.57 |
| 1 | IR22 | 30 | BPH | 6 | 0.00 | 0.00 | 0.00 | 0.10 | 0.90 | 0.00 | 0.00 | 0.00 |  |  | 1.06 |
| 2 | IR22 | 30 | BPH | 6 | 0.00 | 0.00 | 0.00 | 0.00 | 1.00 | 0.00 | 0.00 | 0.00 |  |  | 0.81 |
| 3 | IR22 | 30 | BPH | 6 | 0.00 | 0.00 | 0.00 | 0.00 | 1.00 | 0.00 | 0.00 | 0.00 |  |  | 0.66 |
| 4 | IR22 | 30 | BPH | 6 | 0.00 | 0.00 | 0.00 | 0.03 | 0.97 | 0.00 | 0.00 | 0.00 |  |  | 0.84 |
| 1 | IR22 | 30 | BPH | 7 | 0.00 | 0.00 | 0.10 | 0.05 | 0.85 | 0.00 | 0.00 | 0.00 |  |  | 1.04 |
| 2 | IR22 | 30 | BPH | 7 | 0.00 | 0.00 | 0.00 | 0.00 | 1.00 | 0.00 | 0.00 | 0.00 |  |  | 1.29 |
| 3 | IR22 | 30 | BPH | 7 | 0.00 | 0.00 | 0.00 | 0.00 | 1.00 | 0.00 | 0.00 | 0.00 |  |  | 1.07 |
| 4 | IR22 | 30 | BPH | 7 | 0.00 | 0.00 | 0.03 | 0.02 | 0.95 | 0.00 | 0.00 | 0.00 |  |  | 1.13 |
| 1 | IR22 | 30 | BPH | 8 | 0.00 | 0.00 | 0.00 | 0.05 | 0.95 | 0.00 | 0.00 | 0.00 |  |  | 1.45 |
| 2 | IR22 | 30 | BPH | 8 | 0.00 | 0.00 | 0.00 | 0.00 | 1.00 | 0.00 | 0.00 | 0.00 |  |  | 1.53 |
| 3 | IR22 | 30 | BPH | 8 | 0.00 | 0.00 | 0.00 | 0.00 | 1.00 | 0.00 | 0.00 | 0.00 |  |  | 1.24 |
| 4 | IR22 | 30 | BPH | 8 | 0.00 | 0.00 | 0.00 | 0.02 | 0.98 | 0.00 | 0.00 | 0.00 |  |  | 1.41 |
| 1 | IR22 | 30 | BPH | 9 | 0.00 | 0.00 | 0.00 | 0.00 | 1.00 | 0.00 | 0.00 | 0.00 |  |  | 1.88 |
| 2 | IR22 | 30 | BPH | 9 | 0.00 | 0.00 | 0.00 | 0.00 | 1.00 | 0.00 | 0.00 | 0.00 |  |  | 2.05 |
| 3 | IR22 | 30 | BPH | 9 | 0.00 | 0.00 | 0.00 | 0.00 | 1.00 | 0.00 | 0.00 | 0.00 |  |  | 1.40 |
| 4 | IR22 | 30 | BPH | 9 | 0.00 | 0.00 | 0.00 | 0.00 | 1.00 | 0.00 | 0.00 | 0.00 |  |  | 1.77 |
| 1 | IR22 | 30 | BPH | 10 | 0.00 | 0.00 | 0.00 | 0.00 | 1.00 | 0.00 | 0.00 | 0.00 |  |  | 2.40 |
| 2 | IR22 | 30 | BPH | 10 | 0.00 | 0.00 | 0.00 | 0.00 | 1.00 | 0.00 | 0.00 | 0.00 |  |  | 2.67 |
| 3 | IR22 | 30 | BPH | 10 | 0.00 | 0.00 | 0.00 | 0.00 | 1.00 | 0.00 | 0.00 | 0.00 |  |  | 2.13 |
| 4 | IR22 | 30 | BPH | 10 | 0.00 | 0.00 | 0.00 | 0.00 | 1.00 | 0.00 | 0.00 | 0.00 |  |  | 2.40 |
| 1 | IR22 | 30 | BPH | 11 | 0.00 | 0.00 | 0.00 | 0.00 | 1.00 | 0.00 | 0.00 | 0.00 |  |  | 2.23 |
| 2 | IR22 | 30 | BPH | 11 | 0.00 | 0.00 | 0.00 | 0.00 | 1.00 | 0.00 | 0.00 | 0.00 |  |  | 3.05 |
| 3 | IR22 | 30 | BPH | 11 | 0.00 | 0.00 | 0.00 | 0.00 | 1.00 | 0.00 | 0.00 | 0.00 |  |  | 3.18 |
| 4 | IR22 | 30 | BPH | 11 | 0.00 | 0.00 | 0.00 | 0.00 | 1.00 | 0.00 | 0.00 | 0.00 |  |  | 2.82 |
| 1 | IR22 | 30 | BPH | 12 | 0.00 | 0.00 | 0.00 | 0.00 | 0.65 | 0.35 | 1.00 | 2.50 | 1.00 | 0.20 | 3.89 |
| 2 | IR22 | 30 | BPH | 12 | 0.00 | 0.00 | 0.00 | 0.00 | 0.90 | 0.10 | 0.50 | 0.50 | 1.00 | 1.00 | 3.76 |
| 3 | IR22 | 30 | BPH | 12 | 0.00 | 0.00 | 0.00 | 0.00 | 0.89 | 0.11 | 0.50 | 0.50 | 1.00 | 0.00 | 3.22 |
| 4 | IR22 | 30 | BPH | 12 | 0.00 | 0.00 | 0.00 | 0.00 | 0.81 | 0.19 | 0.67 | 1.17 | 1.00 | 0.40 | 3.62 |
| 1 | IR22 | 30 | BPH | 13 | 0.00 | 0.00 | 0.00 | 0.00 | 0.75 | 0.25 | 0.50 | 2.00 | 1.00 | 0.25 | 2.91 |
| 2 | IR22 | 30 | BPH | 13 | 0.00 | 0.00 | 0.00 | 0.00 | 0.50 | 0.50 | 3.00 | 2.00 | 1.00 | 1.00 | 4.48 |
| 3 | IR22 | 30 | BPH | 13 | 0.00 | 0.00 | 0.00 | 0.00 | 0.75 | 0.25 | 1.50 | 1.00 | 1.00 | 0.00 | 3.67 |
| 4 | IR22 | 30 | BPH | 13 | 0.00 | 0.00 | 0.00 | 0.00 | 0.67 | 0.33 | 1.67 | 1.67 | 1.00 | 0.42 | 3.69 |
| 1 | IR22 | 30 | BPH | 14 | 0.00 | 0.00 | 0.00 | 0.00 | 0.70 | 0.30 | 1.00 | 2.00 | 0.00 | 0.50 | 4.13 |
| 2 | IR22 | 30 | BPH | 14 | 0.00 | 0.00 | 0.00 | 0.00 | 0.63 | 0.37 | 1.00 | 2.50 | 1.00 | 0.60 | 3.41 |
| 3 | IR22 | 30 | BPH | 14 | 0.00 | 0.00 | 0.00 | 0.00 | 0.47 | 0.53 | 0.00 | 5.00 |  | 0.40 | 2.31 |
| 4 | IR22 | 30 | BPH | 14 | 0.00 | 0.00 | 0.00 | 0.00 | 0.60 | 0.40 | 0.67 | 3.17 | 0.50 | 0.50 | 3.28 |
| 1 | IR22 | 30 | BPH | 15 | 0.00 | 0.00 | 0.00 | 0.00 | 0.10 | 0.90 | 2.00 | 2.50 | 1.00 | 0.40 | 2.20 |
| 2 | IR22 | 30 | BPH | 15 | 0.00 | 0.00 | 0.00 | 0.00 | 0.25 | 0.75 | 4.00 | 3.50 | 1.00 | 0.29 | 3.91 |
| 3 | IR22 | 30 | BPH | 15 | 0.00 | 0.00 | 0.00 | 0.00 | 0.05 | 0.95 | 3.00 | 6.50 | 1.00 | 0.38 | 3.92 |
| 4 | IR22 | 30 | BPH | 15 | 0.00 | 0.00 | 0.00 | 0.00 | 0.13 | 0.87 | 3.00 | 4.17 | 1.00 | 0.36 | 3.34 |
| 1 | IR22 | 35 | BPH | 1 | 0.00 | 0.16 | 0.53 | 0.32 | 0.00 | 0.00 | 0.00 | 0.00 |  |  | 0.16 |
| 2 | IR22 | 35 | BPH | 1 | 0.00 | 0.31 | 0.69 | 0.00 | 0.00 | 0.00 | 0.00 | 0.00 |  |  | 0.12 |
| 3 | IR22 | 35 | BPH | 1 | 0.17 | 0.83 | 0.00 | 0.00 | 0.00 | 0.00 | 0.00 | 0.00 |  |  | 0.10 |
| 4 | IR22 | 35 | BPH | 1 | 1.00 | 0.00 | 0.00 | 0.00 | 0.00 | 0.00 | 0.00 | 0.00 |  |  | 0.08 |
| 1 | IR22 | 35 | BPH | 2 | 0.00 | 0.06 | 0.50 | 0.44 | 0.00 | 0.00 | 0.00 | 0.00 |  |  | 0.17 |
| 2 | IR22 | 35 | BPH | 2 | 0.00 | 0.08 | 0.92 | 0.00 | 0.00 | 0.00 | 0.00 | 0.00 |  |  | 0.10 |
| 3 | IR22 | 35 | BPH | 2 | 0.00 | 0.00 | 1.00 | 0.00 | 0.00 | 0.00 | 0.00 | 0.00 |  |  | 0.14 |
| 4 | IR22 | 35 | BPH | 2 | 1.00 | 0.00 | 0.00 | 0.00 | 0.00 | 0.00 | 0.00 | 0.00 |  |  | 0.12 |
| 1 | IR22 | 35 | BPH | 3 | 0.00 | 0.00 | 0.50 | 0.50 | 0.00 | 0.00 | 0.00 | 0.00 |  |  | 0.19 |
| 2 | IR22 | 35 | BPH | 3 | 0.00 | 0.00 | 0.11 | 0.83 | 0.06 | 0.00 | 0.00 | 0.00 |  |  | 0.14 |
| 3 | IR22 | 35 | BPH | 3 | 0.00 | 0.00 | 0.00 | 0.56 | 0.44 | 0.00 | 0.00 | 0.00 |  |  | 0.19 |
| 4 | IR22 | 35 | BPH | 3 | 1.00 | 0.00 | 0.00 | 0.00 | 0.00 | 0.00 | 0.00 | 0.00 |  |  | 0.08 |
| 1 | IR22 | 35 | BPH | 4 | 0.00 | 0.00 | 0.11 | 0.89 | 0.00 | 0.00 | 0.00 | 0.00 |  |  | 0.17 |
| 2 | IR22 | 35 | BPH | 4 | 0.00 | 0.00 | 0.00 | 0.08 | 0.92 | 0.00 | 0.00 | 0.00 |  |  | 0.17 |
| 3 | IR22 | 35 | BPH | 4 | 0.00 | 0.00 | 0.00 | 0.07 | 0.93 | 0.00 | 0.00 | 0.00 |  |  | 0.12 |
| 4 | IR22 | 35 | BPH | 4 | 1.00 | 0.00 | 0.00 | 0.00 | 0.00 | 0.00 | 0.00 | 0.00 |  |  | 0.08 |
| 1 | IR22 | 35 | BPH | 5 | 0.00 | 0.00 | 0.00 | 0.94 | 0.06 | 0.00 | 0.00 | 0.00 |  |  | 0.28 |
| 2 | IR22 | 35 | BPH | 5 | 0.00 | 0.00 | 0.00 | 0.00 | 1.00 | 0.00 | 0.00 | 0.00 |  |  | 0.17 |
| 3 | IR22 | 35 | BPH | 5 | 0.00 | 0.00 | 0.00 | 0.15 | 0.85 | 0.00 | 0.00 | 0.00 |  |  | 0.16 |
| 4 | IR22 | 35 | BPH | 5 | 0.94 | 0.06 | 0.00 | 0.00 | 0.00 | 0.00 | 0.00 | 0.00 |  |  | 0.05 |
| 1 | IR22 | 35 | BPH | 6 | 0.00 | 0.00 | 0.00 | 0.67 | 0.33 | 0.00 | 0.00 | 0.00 |  |  | 0.18 |
| 2 | IR22 | 35 | BPH | 6 | 0.00 | 0.00 | 0.00 | 0.00 | 1.00 | 0.00 | 0.00 | 0.00 |  |  | 0.26 |
| 3 | IR22 | 35 | BPH | 6 | 0.00 | 0.00 | 0.00 | 0.00 | 1.00 | 0.00 | 0.00 | 0.00 |  |  | 0.17 |
| 4 | IR22 | 35 | BPH | 6 | 1.00 | 0.00 | 0.00 | 0.00 | 0.00 | 0.00 | 0.00 | 0.00 |  |  | 0.07 |
| 1 | IR22 | 35 | BPH | 7 | 0.00 | 0.00 | 0.13 | 0.69 | 0.19 | 0.00 | 0.00 | 0.00 |  |  | 0.17 |
| 2 | IR22 | 35 | BPH | 7 | 0.00 | 0.00 | 0.00 | 0.00 | 1.00 | 0.00 | 0.00 | 0.00 |  |  | 0.22 |
| 3 | IR22 | 35 | BPH | 7 | 0.00 | 0.00 | 0.00 | 0.00 | 1.00 | 0.00 | 0.00 | 0.00 |  |  | 0.09 |
| 4 | IR22 | 35 | BPH | 7 | 1.00 | 0.00 | 0.00 | 0.00 | 0.00 | 0.00 | 0.00 | 0.00 |  |  | 0.05 |
| 1 | IR22 | 35 | BPH | 8 | 0.00 | 0.00 | 0.00 | 0.08 | 0.92 | 0.00 | 0.00 | 0.00 |  |  | 0.19 |
| 2 | IR22 | 35 | BPH | 8 | 0.00 | 0.00 | 0.00 | 0.00 | 1.00 | 0.00 | 0.00 | 0.00 |  |  | 0.23 |
| 3 | IR22 | 35 | BPH | 8 | 0.00 | 0.00 | 0.00 | 0.00 | 1.00 | 0.00 | 0.00 | 0.00 |  |  | 0.06 |
| 4 | IR22 | 35 | BPH | 8 |  |  |  |  |  |  | 0.00 | 0.00 |  |  | 0.00 |
| 1 | IR22 | 35 | BPH | 9 | 0.00 | 0.00 | 0.00 | 0.00 | 1.00 | 0.00 | 0.00 | 0.00 |  |  | 0.15 |
| 2 | IR22 | 35 | BPH | 9 | 0.00 | 0.00 | 0.00 | 0.00 | 1.00 | 0.00 | 0.00 | 0.00 |  |  | 0.25 |
| 3 | IR22 | 35 | BPH | 9 | 0.00 | 0.00 | 0.00 | 0.00 | 1.00 | 0.00 | 0.00 | 0.00 |  |  | 0.07 |
| 4 | IR22 | 35 | BPH | 9 |  |  |  |  |  |  | 0.00 | 0.00 |  |  | 0.00 |
| 1 | IR22 | 35 | BPH | 10 | 0.00 | 0.00 | 0.00 | 0.00 | 1.00 | 0.00 | 0.00 | 0.00 |  |  | 0.10 |
| 2 | IR22 | 35 | BPH | 10 | 0.00 | 0.00 | 0.00 | 0.00 | 1.00 | 0.00 | 0.00 | 0.00 |  |  | 0.16 |
| 3 | IR22 | 35 | BPH | 10 | 0.00 | 0.00 | 0.00 | 0.00 | 1.00 | 0.00 | 0.00 | 0.00 |  |  | 0.05 |
| 4 | IR22 | 35 | BPH | 10 |  |  |  |  |  |  | 0.00 | 0.00 |  |  | 0.00 |
| 1 | IR22 | 35 | BPH | 11 | 0.00 | 0.00 | 0.00 | 0.00 | 1.00 | 0.00 | 0.00 | 0.00 |  |  | 0.08 |
| 2 | IR22 | 35 | BPH | 11 | 0.00 | 0.00 | 0.00 | 0.00 | 1.00 | 0.00 | 0.00 | 0.00 |  |  | 0.41 |
| 3 | IR22 | 35 | BPH | 11 |  |  |  |  |  |  | 0.00 | 0.00 |  |  | 0.00 |
| 4 | IR22 | 35 | BPH | 11 |  |  |  |  |  |  | 0.00 | 0.00 |  |  | 0.00 |
| 1 | IR22 | 35 | BPH | 12 | 0.00 | 0.00 | 0.00 | 0.00 | 1.00 | 0.00 | 0.00 | 0.00 |  |  | 0.03 |
| 2 | IR22 | 35 | BPH | 12 | 0.00 | 0.00 | 0.00 | 0.00 | 1.00 | 0.00 | 0.00 | 0.00 |  |  | 0.21 |
| 3 | IR22 | 35 | BPH | 12 | 0.00 | 0.00 | 0.00 | 0.00 | 1.00 | 0.00 | 0.00 | 0.00 |  |  | 0.04 |
| 4 | IR22 | 35 | BPH | 12 |  |  |  |  |  |  | 0.00 | 0.00 |  |  | 0.00 |
| 1 | IR22 | 35 | BPH | 13 |  |  |  |  |  |  | 0.00 | 0.00 |  |  | 0.00 |
| 2 | IR22 | 35 | BPH | 13 | 0.00 | 0.00 | 0.00 | 0.00 | 1.00 | 0.00 | 0.00 | 0.00 |  |  | 0.42 |
| 3 | IR22 | 35 | BPH | 13 | 0.00 | 0.00 | 0.00 | 0.00 | 1.00 | 0.00 | 0.00 | 0.00 |  |  | 0.03 |
| 4 | IR22 | 35 | BPH | 13 |  |  |  |  |  |  | 0.00 | 0.00 |  |  | 0.00 |
| 1 | IR22 | 35 | BPH | 14 |  |  |  |  |  |  | 0.00 | 0.00 |  |  | 0.00 |
| 2 | IR22 | 35 | BPH | 14 | 0.00 | 0.00 | 0.00 | 0.00 | 1.00 | 0.00 | 0.00 | 0.00 |  |  | 0.38 |
| 3 | IR22 | 35 | BPH | 14 | 0.00 | 0.00 | 0.00 | 0.00 | 1.00 | 0.00 | 0.00 | 0.00 |  |  | 0.00 |
| 4 | IR22 | 35 | BPH | 14 |  |  |  |  |  |  | 0.00 | 0.00 |  |  | 0.00 |
| 1 | IR22 | 35 | BPH | 15 | 0.00 | 0.00 | 0.00 | 0.00 | 1.00 | 0.00 | 0.00 | 0.00 |  |  | 0.04 |
| 2 | IR22 | 35 | BPH | 15 | 0.00 | 0.00 | 0.00 | 0.00 | 1.00 | 0.00 | 0.00 | 0.00 |  |  | 0.40 |
| 3 | IR22 | 35 | BPH | 15 | 0.00 | 0.00 | 0.00 | 0.00 | 1.00 | 0.00 | 0.00 | 0.00 |  |  | 0.01 |
| 4 | IR22 | 35 | BPH | 15 |  |  |  |  |  |  | 0.00 | 0.00 |  |  | 0.00 |
| 4 | IR22 | 40 | BPH | 1 |  |  |  |  |  |  | 0.00 | 0.00 |  |  | 0.00 |
| 4 | IR22 | 40 | BPH | 1 |  |  |  |  |  |  | 0.00 | 0.00 |  |  | 0.00 |
| 4 | IR22 | 40 | BPH | 1 |  |  |  |  |  |  | 0.00 | 0.00 |  |  | 0.00 |
| 4 | IR22 | 40 | BPH | 1 |  |  |  |  |  |  | 0.00 | 0.00 |  |  | 0.00 |
| 4 | IR22 | 40 | BPH | 2 |  |  |  |  |  |  | 0.00 | 0.00 |  |  | 0.00 |
| 4 | IR22 | 40 | BPH | 2 |  |  |  |  |  |  | 0.00 | 0.00 |  |  | 0.00 |
| 4 | IR22 | 40 | BPH | 2 |  |  |  |  |  |  | 0.00 | 0.00 |  |  | 0.00 |
| 4 | IR22 | 40 | BPH | 2 |  |  |  |  |  |  | 0.00 | 0.00 |  |  | 0.00 |
| 4 | IR22 | 40 | BPH | 3 |  |  |  |  |  |  | 0.00 | 0.00 |  |  | 0.00 |
| 4 | IR22 | 40 | BPH | 3 |  |  |  |  |  |  | 0.00 | 0.00 |  |  | 0.00 |
| 4 | IR22 | 40 | BPH | 3 |  |  |  |  |  |  | 0.00 | 0.00 |  |  | 0.00 |
| 4 | IR22 | 40 | BPH | 3 |  |  |  |  |  |  | 0.00 | 0.00 |  |  | 0.00 |
| 4 | IR22 | 40 | BPH | 4 |  |  |  |  |  |  | 0.00 | 0.00 |  |  | 0.00 |
| 4 | IR22 | 40 | BPH | 4 |  |  |  |  |  |  | 0.00 | 0.00 |  |  | 0.00 |
| 4 | IR22 | 40 | BPH | 4 |  |  |  |  |  |  | 0.00 | 0.00 |  |  | 0.00 |
| 4 | IR22 | 40 | BPH | 4 |  |  |  |  |  |  | 0.00 | 0.00 |  |  | 0.00 |
| 4 | IR22 | 40 | BPH | 5 |  |  |  |  |  |  | 0.00 | 0.00 |  |  | 0.00 |
| 4 | IR22 | 40 | BPH | 5 |  |  |  |  |  |  | 0.00 | 0.00 |  |  | 0.00 |
| 4 | IR22 | 40 | BPH | 5 |  |  |  |  |  |  | 0.00 | 0.00 |  |  | 0.00 |
| 4 | IR22 | 40 | BPH | 5 |  |  |  |  |  |  | 0.00 | 0.00 |  |  | 0.00 |
| 4 | IR22 | 40 | BPH | 6 |  |  |  |  |  |  | 0.00 | 0.00 |  |  | 0.00 |
| 4 | IR22 | 40 | BPH | 6 |  |  |  |  |  |  | 0.00 | 0.00 |  |  | 0.00 |
| 4 | IR22 | 40 | BPH | 6 |  |  |  |  |  |  | 0.00 | 0.00 |  |  | 0.00 |
| 4 | IR22 | 40 | BPH | 6 |  |  |  |  |  |  | 0.00 | 0.00 |  |  | 0.00 |
| 4 | IR22 | 40 | BPH | 7 |  |  |  |  |  |  | 0.00 | 0.00 |  |  | 0.00 |
| 4 | IR22 | 40 | BPH | 7 |  |  |  |  |  |  | 0.00 | 0.00 |  |  | 0.00 |
| 4 | IR22 | 40 | BPH | 7 |  |  |  |  |  |  | 0.00 | 0.00 |  |  | 0.00 |
| 4 | IR22 | 40 | BPH | 7 |  |  |  |  |  |  | 0.00 | 0.00 |  |  | 0.00 |
| 4 | IR22 | 40 | BPH | 8 |  |  |  |  |  |  | 0.00 | 0.00 |  |  | 0.00 |
| 4 | IR22 | 40 | BPH | 8 |  |  |  |  |  |  | 0.00 | 0.00 |  |  | 0.00 |
| 4 | IR22 | 40 | BPH | 8 |  |  |  |  |  |  | 0.00 | 0.00 |  |  | 0.00 |
| 4 | IR22 | 40 | BPH | 8 |  |  |  |  |  |  | 0.00 | 0.00 |  |  | 0.00 |
| 4 | IR22 | 40 | BPH | 9 |  |  |  |  |  |  | 0.00 | 0.00 |  |  | 0.00 |
| 4 | IR22 | 40 | BPH | 9 |  |  |  |  |  |  | 0.00 | 0.00 |  |  | 0.00 |
| 4 | IR22 | 40 | BPH | 9 |  |  |  |  |  |  | 0.00 | 0.00 |  |  | 0.00 |
| 4 | IR22 | 40 | BPH | 9 |  |  |  |  |  |  | 0.00 | 0.00 |  |  | 0.00 |
| 4 | IR22 | 40 | BPH | 10 |  |  |  |  |  |  | 0.00 | 0.00 |  |  | 0.00 |
| 4 | IR22 | 40 | BPH | 10 |  |  |  |  |  |  | 0.00 | 0.00 |  |  | 0.00 |
| 4 | IR22 | 40 | BPH | 10 |  |  |  |  |  |  | 0.00 | 0.00 |  |  | 0.00 |
| 4 | IR22 | 40 | BPH | 10 |  |  |  |  |  |  | 0.00 | 0.00 |  |  | 0.00 |
| 4 | IR22 | 40 | BPH | 11 |  |  |  |  |  |  | 0.00 | 0.00 |  |  | 0.00 |
| 4 | IR22 | 40 | BPH | 11 |  |  |  |  |  |  | 0.00 | 0.00 |  |  | 0.00 |
| 4 | IR22 | 40 | BPH | 11 |  |  |  |  |  |  | 0.00 | 0.00 |  |  | 0.00 |
| 4 | IR22 | 40 | BPH | 11 |  |  |  |  |  |  | 0.00 | 0.00 |  |  | 0.00 |
| 4 | IR22 | 40 | BPH | 12 |  |  |  |  |  |  | 0.00 | 0.00 |  |  | 0.00 |
| 4 | IR22 | 40 | BPH | 12 |  |  |  |  |  |  | 0.00 | 0.00 |  |  | 0.00 |
| 4 | IR22 | 40 | BPH | 12 |  |  |  |  |  |  | 0.00 | 0.00 |  |  | 0.00 |
| 4 | IR22 | 40 | BPH | 12 |  |  |  |  |  |  | 0.00 | 0.00 |  |  | 0.00 |
| 4 | IR22 | 40 | BPH | 13 |  |  |  |  |  |  | 0.00 | 0.00 |  |  | 0.00 |
| 4 | IR22 | 40 | BPH | 13 |  |  |  |  |  |  | 0.00 | 0.00 |  |  | 0.00 |
| 4 | IR22 | 40 | BPH | 13 |  |  |  |  |  |  | 0.00 | 0.00 |  |  | 0.00 |
| 4 | IR22 | 40 | BPH | 13 |  |  |  |  |  |  | 0.00 | 0.00 |  |  | 0.00 |
| 4 | IR22 | 40 | BPH | 14 |  |  |  |  |  |  | 0.00 | 0.00 |  |  | 0.00 |
| 4 | IR22 | 40 | BPH | 14 |  |  |  |  |  |  | 0.00 | 0.00 |  |  | 0.00 |
| 4 | IR22 | 40 | BPH | 14 |  |  |  |  |  |  | 0.00 | 0.00 |  |  | 0.00 |
| 4 | IR22 | 40 | BPH | 14 |  |  |  |  |  |  | 0.00 | 0.00 |  |  | 0.00 |
| 4 | IR22 | 40 | BPH | 15 |  |  |  |  |  |  | 0.00 | 0.00 |  |  | 0.00 |
| 4 | IR22 | 40 | BPH | 15 |  |  |  |  |  |  | 0.00 | 0.00 |  |  | 0.00 |
| 4 | IR22 | 40 | BPH | 15 |  |  |  |  |  |  | 0.00 | 0.00 |  |  | 0.00 |
| 4 | IR22 | 40 | BPH | 15 |  |  |  |  |  |  | 0.00 | 0.00 |  |  | 0.00 |
| 1 | IR22 | 15 | WBPH | 1 | 0.00 | 0.38 | 0.62 | 0.00 | 0.00 | 0.00 | 0.00 | 0.00 |  |  | 0.04 |
| 2 | IR22 | 15 | WBPH | 1 | 0.00 | 0.36 | 0.64 | 0.00 | 0.00 | 0.00 | 0.00 | 0.00 |  |  | 0.03 |
| 3 | IR22 | 15 | WBPH | 1 | 0.36 | 0.57 | 0.07 | 0.00 | 0.00 | 0.00 | 0.00 | 0.00 |  |  | 0.04 |
| 4 | IR22 | 15 | WBPH | 1 | 1.00 | 0.00 | 0.00 | 0.00 | 0.00 | 0.00 | 0.00 | 0.00 |  |  | 0.09 |
| 1 | IR22 | 15 | WBPH | 2 | 0.41 | 0.35 | 0.24 | 0.00 | 0.00 | 0.00 | 0.00 | 0.00 |  |  | 0.07 |
| 2 | IR22 | 15 | WBPH | 2 | 0.00 | 0.29 | 0.71 | 0.00 | 0.00 | 0.00 | 0.00 | 0.00 |  |  | 0.02 |
| 3 | IR22 | 15 | WBPH | 2 | 0.18 | 0.55 | 0.27 | 0.00 | 0.00 | 0.00 | 0.00 | 0.00 |  |  | 0.03 |
| 4 | IR22 | 15 | WBPH | 2 | 1.00 | 0.00 | 0.00 | 0.00 | 0.00 | 0.00 | 0.00 | 0.00 |  |  | 0.10 |
| 1 | IR22 | 15 | WBPH | 3 | 0.07 | 0.07 | 0.87 | 0.00 | 0.00 | 0.00 | 0.00 | 0.00 |  |  | 0.09 |
| 2 | IR22 | 15 | WBPH | 3 | 0.00 | 0.00 | 0.83 | 0.17 | 0.00 | 0.00 | 0.00 | 0.00 |  |  | 0.02 |
| 3 | IR22 | 15 | WBPH | 3 | 0.00 | 0.13 | 0.87 | 0.00 | 0.00 | 0.00 | 0.00 | 0.00 |  |  | 0.06 |
| 4 | IR22 | 15 | WBPH | 3 | 1.00 | 0.00 | 0.00 | 0.00 | 0.00 | 0.00 | 0.00 | 0.00 |  |  | 0.10 |
| 1 | IR22 | 15 | WBPH | 4 | 0.00 | 0.07 | 0.47 | 0.47 | 0.00 | 0.00 | 0.00 | 0.00 |  |  | 0.09 |
| 2 | IR22 | 15 | WBPH | 4 | 0.00 | 0.06 | 0.31 | 0.63 | 0.00 | 0.00 | 0.00 | 0.00 |  |  | 0.06 |
| 3 | IR22 | 15 | WBPH | 4 | 0.00 | 0.06 | 0.19 | 0.75 | 0.00 | 0.00 | 0.00 | 0.00 |  |  | 0.07 |
| 4 | IR22 | 15 | WBPH | 4 | 1.00 | 0.00 | 0.00 | 0.00 | 0.00 | 0.00 | 0.00 | 0.00 |  |  | 0.15 |
| 1 | IR22 | 15 | WBPH | 5 | 0.00 | 0.00 | 0.16 | 0.84 | 0.00 | 0.00 | 0.00 | 0.00 |  |  | 0.13 |
| 2 | IR22 | 15 | WBPH | 5 | 0.00 | 0.07 | 0.13 | 0.53 | 0.27 | 0.00 | 0.00 | 0.00 |  |  | 0.06 |
| 3 | IR22 | 15 | WBPH | 5 | 0.00 | 0.00 | 0.14 | 0.86 | 0.00 | 0.00 | 0.00 | 0.00 |  |  | 0.04 |
| 4 | IR22 | 15 | WBPH | 5 | 0.98 | 0.02 | 0.00 | 0.00 | 0.00 | 0.00 | 0.00 | 0.00 |  |  | 0.15 |
| 1 | IR22 | 15 | WBPH | 6 | 0.00 | 0.00 | 0.24 | 0.76 | 0.00 | 0.00 | 0.00 | 0.00 |  |  | 0.12 |
| 2 | IR22 | 15 | WBPH | 6 | 0.00 | 0.00 | 0.00 | 0.47 | 0.53 | 0.00 | 0.00 | 0.00 |  |  | 0.06 |
| 3 | IR22 | 15 | WBPH | 6 | 0.00 | 0.00 | 0.00 | 0.24 | 0.76 | 0.00 | 0.00 | 0.00 |  |  | 0.09 |
| 4 | IR22 | 15 | WBPH | 6 | 0.84 | 0.16 | 0.00 | 0.00 | 0.00 | 0.00 | 0.00 | 0.00 |  |  | 0.11 |
| 1 | IR22 | 15 | WBPH | 7 | 0.00 | 0.00 | 0.06 | 0.44 | 0.50 | 0.00 | 0.00 | 0.00 |  |  | 0.14 |
| 2 | IR22 | 15 | WBPH | 7 | 0.00 | 0.00 | 0.00 | 0.42 | 0.58 | 0.00 | 0.00 | 0.00 |  |  | 0.08 |
| 3 | IR22 | 15 | WBPH | 7 | 0.00 | 0.00 | 0.00 | 0.00 | 1.00 | 0.00 | 0.00 | 0.00 |  |  | 0.11 |
| 4 | IR22 | 15 | WBPH | 7 | 0.53 | 0.47 | 0.00 | 0.00 | 0.00 | 0.00 | 0.00 | 0.00 |  |  | 0.12 |
| 1 | IR22 | 15 | WBPH | 8 | 0.00 | 0.00 | 0.00 | 0.00 | 1.00 | 0.00 | 0.00 | 0.00 |  |  | 0.10 |
| 2 | IR22 | 15 | WBPH | 8 | 0.00 | 0.00 | 0.00 | 0.23 | 0.77 | 0.00 | 0.00 | 0.00 |  |  | 0.07 |
| 3 | IR22 | 15 | WBPH | 8 | 0.00 | 0.00 | 0.00 | 0.00 | 1.00 | 0.00 | 0.00 | 0.00 |  |  | 0.14 |
| 4 | IR22 | 15 | WBPH | 8 | 0.37 | 0.63 | 0.00 | 0.00 | 0.00 | 0.00 | 0.00 | 0.00 |  |  | 0.10 |
| 1 | IR22 | 15 | WBPH | 9 | 0.00 | 0.00 | 0.00 | 0.19 | 0.81 | 0.00 | 0.00 | 0.00 |  |  | 0.11 |
| 2 | IR22 | 15 | WBPH | 9 | 0.00 | 0.00 | 0.00 | 0.00 | 1.00 | 0.00 | 0.00 | 0.00 |  |  | 0.08 |
| 3 | IR22 | 15 | WBPH | 9 | 0.00 | 0.00 | 0.00 | 0.00 | 1.00 | 0.00 | 0.00 | 0.00 |  |  | 0.11 |
| 4 | IR22 | 15 | WBPH | 9 | 0.06 | 0.42 | 0.53 | 0.00 | 0.00 | 0.00 | 0.00 | 0.00 |  |  | 0.34 |
| 1 | IR22 | 15 | WBPH | 10 | 0.00 | 0.00 | 0.00 | 0.00 | 1.00 | 0.00 | 0.00 | 0.00 |  |  | 0.13 |
| 2 | IR22 | 15 | WBPH | 10 | 0.00 | 0.00 | 0.00 | 0.00 | 1.00 | 0.00 | 0.00 | 0.00 |  |  | 0.08 |
| 3 | IR22 | 15 | WBPH | 10 | 0.00 | 0.00 | 0.00 | 0.00 | 1.00 | 0.00 | 0.00 | 0.00 |  |  | 0.11 |
| 4 | IR22 | 15 | WBPH | 10 | 0.00 | 0.33 | 0.67 | 0.00 | 0.00 | 0.00 | 0.00 | 0.00 |  |  | 0.44 |
| 1 | IR22 | 15 | WBPH | 11 | 0.00 | 0.00 | 0.00 | 0.00 | 1.00 | 0.00 | 0.00 | 0.00 |  |  | 0.21 |
| 2 | IR22 | 15 | WBPH | 11 | 0.00 | 0.00 | 0.00 | 0.06 | 0.94 | 0.00 | 0.00 | 0.00 |  |  | 0.10 |
| 3 | IR22 | 15 | WBPH | 11 | 0.00 | 0.00 | 0.00 | 0.00 | 1.00 | 0.00 | 0.00 | 0.00 |  |  | 0.14 |
| 4 | IR22 | 15 | WBPH | 11 | 0.00 | 0.13 | 0.88 | 0.00 | 0.00 | 0.00 | 0.00 | 0.00 |  |  | 0.59 |
| 1 | IR22 | 15 | WBPH | 12 | 0.00 | 0.00 | 0.00 | 0.00 | 1.00 | 0.00 | 0.00 | 0.00 |  |  | 0.18 |
| 2 | IR22 | 15 | WBPH | 12 | 0.00 | 0.00 | 0.00 | 0.06 | 0.94 | 0.00 | 0.00 | 0.00 |  |  | 0.11 |
| 3 | IR22 | 15 | WBPH | 12 | 0.00 | 0.00 | 0.00 | 0.00 | 1.00 | 0.00 | 0.00 | 0.00 |  |  | 0.16 |
| 4 | IR22 | 15 | WBPH | 12 | 0.00 | 0.10 | 0.83 | 0.08 | 0.00 | 0.00 | 0.00 | 0.00 |  |  | 0.61 |
| 1 | IR22 | 15 | WBPH | 13 | 0.00 | 0.00 | 0.00 | 0.00 | 1.00 | 0.00 | 0.00 | 0.00 |  |  | 0.31 |
| 2 | IR22 | 15 | WBPH | 13 | 0.00 | 0.00 | 0.00 | 0.00 | 1.00 | 0.00 | 0.00 | 0.00 |  |  | 0.09 |
| 3 | IR22 | 15 | WBPH | 13 | 0.00 | 0.00 | 0.00 | 0.00 | 1.00 | 0.00 | 0.00 | 0.00 |  |  | 0.23 |
| 4 | IR22 | 15 | WBPH | 13 | 0.00 | 0.00 | 0.73 | 0.27 | 0.00 | 0.00 | 0.00 | 0.00 |  |  | 0.69 |
| 1 | IR22 | 15 | WBPH | 14 | 0.00 | 0.00 | 0.00 | 0.00 | 1.00 | 0.00 | 0.00 | 0.00 |  |  | 0.32 |
| 2 | IR22 | 15 | WBPH | 14 | 0.00 | 0.00 | 0.00 | 0.00 | 1.00 | 0.00 | 0.00 | 0.00 |  |  | 0.09 |
| 3 | IR22 | 15 | WBPH | 14 | 0.00 | 0.00 | 0.00 | 0.00 | 1.00 | 0.00 | 0.00 | 0.00 |  |  | 0.27 |
| 4 | IR22 | 15 | WBPH | 14 | 0.00 | 0.02 | 0.76 | 0.22 | 0.00 | 0.00 | 0.00 | 0.00 |  |  | 0.74 |
| 1 | IR22 | 15 | WBPH | 15 | 0.00 | 0.00 | 0.00 | 0.00 | 1.00 | 0.00 | 0.00 | 0.00 |  |  | 0.25 |
| 2 | IR22 | 15 | WBPH | 15 | 0.00 | 0.00 | 0.00 | 0.00 | 1.00 | 0.00 | 0.00 | 0.00 |  |  | 0.10 |
| 3 | IR22 | 15 | WBPH | 15 | 0.00 | 0.00 | 0.00 | 0.00 | 1.00 | 0.00 | 0.00 | 0.00 |  |  | 0.21 |
| 4 | IR22 | 15 | WBPH | 15 | 0.00 | 0.00 | 0.33 | 0.67 | 0.00 | 0.00 | 0.00 | 0.00 |  |  | 0.84 |
| 1 | IR22 | 15 | WBPH | 16 | 0.00 | 0.00 | 0.00 | 0.00 | 1.00 | 0.00 | 0.00 | 0.00 |  |  | 0.28 |
| 2 | IR22 | 15 | WBPH | 16 | 0.00 | 0.00 | 0.00 | 0.00 | 1.00 | 0.00 | 0.00 | 0.00 |  |  | 0.13 |
| 3 | IR22 | 15 | WBPH | 16 | 0.00 | 0.00 | 0.00 | 0.00 | 1.00 | 0.00 | 0.00 | 0.00 |  |  | 0.22 |
| 4 | IR22 | 15 | WBPH | 16 | 0.00 | 0.00 | 0.00 | 0.00 | 1.00 | 0.00 | 0.00 | 0.00 |  |  | 0.21 |
| 1 | IR22 | 15 | WBPH | 17 | 0.00 | 0.00 | 0.00 | 0.00 | 1.00 | 0.00 | 0.00 | 0.00 |  |  | 0.40 |
| 2 | IR22 | 15 | WBPH | 17 | 0.00 | 0.00 | 0.00 | 0.00 | 1.00 | 0.00 | 0.00 | 0.00 |  |  | 0.18 |
| 3 | IR22 | 15 | WBPH | 17 | 0.00 | 0.00 | 0.00 | 0.00 | 1.00 | 0.00 | 0.00 | 0.00 |  |  | 0.25 |
| 4 | IR22 | 15 | WBPH | 17 | 0.00 | 0.00 | 0.00 | 0.00 | 1.00 | 0.00 | 0.00 | 0.00 |  |  | 0.27 |
| 1 | IR22 | 15 | WBPH | 18 | 0.00 | 0.00 | 0.00 | 0.00 | 1.00 | 0.00 | 0.00 | 0.00 |  |  | 0.39 |
| 2 | IR22 | 15 | WBPH | 18 | 0.00 | 0.00 | 0.00 | 0.00 | 1.00 | 0.00 | 0.00 | 0.00 |  |  | 0.29 |
| 3 | IR22 | 15 | WBPH | 18 | 0.00 | 0.00 | 0.00 | 0.00 | 1.00 | 0.00 | 0.00 | 0.00 |  |  | 0.17 |
| 4 | IR22 | 15 | WBPH | 18 | 0.00 | 0.00 | 0.00 | 0.00 | 1.00 | 0.00 | 0.00 | 0.00 |  |  | 0.29 |
| 1 | IR22 | 15 | WBPH | 19 | 0.00 | 0.00 | 0.00 | 0.00 | 1.00 | 0.00 | 0.00 | 0.00 |  |  | 0.36 |
| 2 | IR22 | 15 | WBPH | 19 | 0.00 | 0.00 | 0.00 | 0.00 | 1.00 | 0.00 | 0.00 | 0.00 |  |  | 0.26 |
| 3 | IR22 | 15 | WBPH | 19 | 0.00 | 0.00 | 0.00 | 0.00 | 1.00 | 0.00 | 0.00 | 0.00 |  |  | 0.20 |
| 4 | IR22 | 15 | WBPH | 19 | 0.00 | 0.00 | 0.00 | 0.00 | 1.00 | 0.00 | 0.00 | 0.00 |  |  | 0.27 |
| 1 | IR22 | 15 | WBPH | 20 | 0.00 | 0.00 | 0.00 | 0.00 | 1.00 | 0.00 | 0.00 | 0.00 |  |  | 0.32 |
| 2 | IR22 | 15 | WBPH | 20 | 0.00 | 0.00 | 0.00 | 0.00 | 1.00 | 0.00 | 0.00 | 0.00 |  |  | 0.24 |
| 3 | IR22 | 15 | WBPH | 20 | 0.00 | 0.00 | 0.00 | 0.07 | 0.93 | 0.00 | 0.00 | 0.00 |  |  | 0.30 |
| 4 | IR22 | 15 | WBPH | 20 | 0.00 | 0.00 | 0.00 | 0.02 | 0.98 | 0.00 | 0.00 | 0.00 |  |  | 0.29 |
| 1 | IR22 | 15 | WBPH | 21 | 0.00 | 0.00 | 0.00 | 0.00 | 1.00 | 0.00 | 0.00 | 0.00 |  |  | 0.63 |
| 2 | IR22 | 15 | WBPH | 21 | 0.00 | 0.00 | 0.00 | 0.00 | 1.00 | 0.00 | 0.00 | 0.00 |  |  | 0.29 |
| 3 | IR22 | 15 | WBPH | 21 | 0.00 | 0.00 | 0.00 | 0.00 | 1.00 | 0.00 | 0.00 | 0.00 |  |  | 0.36 |
| 4 | IR22 | 15 | WBPH | 21 | 0.00 | 0.00 | 0.00 | 0.00 | 1.00 | 0.00 | 0.00 | 0.00 |  |  | 0.42 |
| 1 | IR22 | 15 | WBPH | 22 | 0.00 | 0.00 | 0.00 | 0.00 | 1.00 | 0.00 | 0.00 | 0.00 |  |  | 0.35 |
| 2 | IR22 | 15 | WBPH | 22 | 0.00 | 0.00 | 0.00 | 0.00 | 1.00 | 0.00 | 0.00 | 0.00 |  |  | 0.27 |
| 3 | IR22 | 15 | WBPH | 22 | 0.00 | 0.00 | 0.00 | 0.00 | 1.00 | 0.00 | 0.00 | 0.00 |  |  | 0.21 |
| 4 | IR22 | 15 | WBPH | 22 | 0.00 | 0.00 | 0.00 | 0.00 | 1.00 | 0.00 | 0.00 | 0.00 |  |  | 0.27 |
| 1 | IR22 | 15 | WBPH | 23 | 0.00 | 0.00 | 0.00 | 0.00 | 1.00 | 0.00 | 0.00 | 0.00 |  |  | 0.49 |
| 2 | IR22 | 15 | WBPH | 23 | 0.00 | 0.00 | 0.00 | 0.00 | 1.00 | 0.00 | 0.00 | 0.00 |  |  | 0.24 |
| 3 | IR22 | 15 | WBPH | 23 | 0.00 | 0.00 | 0.00 | 0.00 | 1.00 | 0.00 | 0.00 | 0.00 |  |  | 0.56 |
| 4 | IR22 | 15 | WBPH | 23 | 0.00 | 0.00 | 0.00 | 0.00 | 1.00 | 0.00 | 0.00 | 0.00 |  |  | 0.43 |
| 1 | IR22 | 15 | WBPH | 24 | 0.00 | 0.00 | 0.00 | 0.00 | 1.00 | 0.00 | 0.00 | 0.00 |  |  | 0.65 |
| 2 | IR22 | 15 | WBPH | 24 | 0.00 | 0.00 | 0.00 | 0.00 | 1.00 | 0.00 | 0.00 | 0.00 |  |  | 0.44 |
| 3 | IR22 | 15 | WBPH | 24 | 0.00 | 0.00 | 0.00 | 0.00 | 1.00 | 0.00 | 0.00 | 0.00 |  |  | 0.28 |
| 4 | IR22 | 15 | WBPH | 24 | 0.00 | 0.00 | 0.00 | 0.00 | 1.00 | 0.00 | 0.00 | 0.00 |  |  | 0.46 |
| 1 | IR22 | 15 | WBPH | 25 | 0.00 | 0.00 | 0.00 | 0.00 | 1.00 | 0.00 | 0.00 | 0.00 |  |  | 0.49 |
| 2 | IR22 | 15 | WBPH | 25 | 0.00 | 0.00 | 0.00 | 0.00 | 1.00 | 0.00 | 0.00 | 0.00 |  |  | 0.29 |
| 3 | IR22 | 15 | WBPH | 25 | 0.00 | 0.00 | 0.00 | 0.00 | 1.00 | 0.00 | 0.00 | 0.00 |  |  | 0.32 |
| 4 | IR22 | 15 | WBPH | 25 | 0.00 | 0.00 | 0.00 | 0.00 | 1.00 | 0.00 | 0.00 | 0.00 |  |  | 0.37 |
| 1 | IR22 | 15 | WBPH | 26 | 0.00 | 0.00 | 0.00 | 0.00 | 1.00 | 0.00 | 0.00 | 0.00 |  |  | 0.53 |
| 2 | IR22 | 15 | WBPH | 26 | 0.00 | 0.00 | 0.00 | 0.00 | 1.00 | 0.00 | 0.00 | 0.00 |  |  | 0.43 |
| 3 | IR22 | 15 | WBPH | 26 | 0.00 | 0.00 | 0.00 | 0.00 | 1.00 | 0.00 | 0.00 | 0.00 |  |  | 0.44 |
| 4 | IR22 | 15 | WBPH | 26 | 0.00 | 0.00 | 0.00 | 0.00 | 1.00 | 0.00 | 0.00 | 0.00 |  |  | 0.47 |
| 1 | IR22 | 15 | WBPH | 27 | 0.00 | 0.00 | 0.00 | 0.00 | 0.94 | 0.00 | 0.00 | 0.00 |  |  | 0.73 |
| 2 | IR22 | 15 | WBPH | 27 | 0.00 | 0.00 | 0.00 | 0.00 | 1.00 | 0.00 | 0.00 | 0.00 |  |  | 0.35 |
| 3 | IR22 | 15 | WBPH | 27 | 0.00 | 0.00 | 0.00 | 0.00 | 1.00 | 0.00 | 0.00 | 0.00 |  |  | 0.45 |
| 4 | IR22 | 15 | WBPH | 27 | 0.00 | 0.00 | 0.00 | 0.00 | 0.98 | 0.00 | 0.00 | 0.00 |  |  | 0.51 |
| 1 | IR22 | 15 | WBPH | 28 | 0.00 | 0.00 | 0.00 | 0.00 | 1.00 | 0.00 | 0.00 | 0.00 |  |  | 0.96 |
| 2 | IR22 | 15 | WBPH | 28 | 0.00 | 0.00 | 0.00 | 0.00 | 1.00 | 0.00 | 0.00 | 0.00 |  |  | 0.33 |
| 3 | IR22 | 15 | WBPH | 28 | 0.00 | 0.00 | 0.00 | 0.00 | 1.00 | 0.00 | 0.00 | 0.00 |  |  | 0.74 |
| 4 | IR22 | 15 | WBPH | 28 | 0.00 | 0.00 | 0.00 | 0.00 | 1.00 | 0.00 | 0.00 | 0.00 |  |  | 0.68 |
| 1 | IR22 | 15 | WBPH | 29 | 0.00 | 0.00 | 0.00 | 0.00 | 1.00 | 0.00 | 0.00 | 0.00 |  |  | 0.91 |
| 2 | IR22 | 15 | WBPH | 29 | 0.00 | 0.00 | 0.00 | 0.00 | 1.00 | 0.00 | 0.00 | 0.00 |  |  | 0.81 |
| 3 | IR22 | 15 | WBPH | 29 | 0.00 | 0.00 | 0.00 | 0.00 | 1.00 | 0.00 | 0.00 | 0.00 |  |  | 0.68 |
| 4 | IR22 | 15 | WBPH | 29 | 0.00 | 0.00 | 0.00 | 0.00 | 1.00 | 0.00 | 0.00 | 0.00 |  |  | 0.80 |
| 1 | IR22 | 15 | WBPH | 30 | 0.00 | 0.00 | 0.00 | 0.00 | 1.00 | 0.00 | 0.00 | 0.00 |  |  | 0.91 |
| 2 | IR22 | 15 | WBPH | 30 | 0.00 | 0.00 | 0.00 | 0.00 | 1.00 | 0.00 | 0.00 | 0.00 |  |  | 0.70 |
| 3 | IR22 | 15 | WBPH | 30 | 0.00 | 0.00 | 0.00 | 0.00 | 1.00 | 0.00 | 0.00 | 0.00 |  |  | 0.62 |
| 4 | IR22 | 15 | WBPH | 30 | 0.00 | 0.00 | 0.00 | 0.00 | 1.00 | 0.00 | 0.00 | 0.00 |  |  | 0.75 |
| 4 | IR22 | 20 | WBPH | 1 | 1.00 | 0.00 | 0.00 | 0.00 | 0.00 | 0.00 | 0.00 | 0.00 |  |  | 0.05 |
| 4 | IR22 | 20 | WBPH | 1 | 1.00 | 0.00 | 0.00 | 0.00 | 0.00 | 0.00 | 0.00 | 0.00 |  |  | 0.06 |
| 4 | IR22 | 20 | WBPH | 1 | 1.00 | 0.00 | 0.00 | 0.00 | 0.00 | 0.00 | 0.00 | 0.00 |  |  | 0.07 |
| 4 | IR22 | 20 | WBPH | 1 | 1.00 | 0.00 | 0.00 | 0.00 | 0.00 | 0.00 | 0.00 | 0.00 |  |  | 0.04 |
| 4 | IR22 | 20 | WBPH | 2 | 1.00 | 0.00 | 0.00 | 0.00 | 0.00 | 0.00 | 0.00 | 0.00 |  |  | 0.11 |
| 4 | IR22 | 20 | WBPH | 2 | 1.00 | 0.00 | 0.00 | 0.00 | 0.00 | 0.00 | 0.00 | 0.00 |  |  | 0.09 |
| 4 | IR22 | 20 | WBPH | 2 | 1.00 | 0.00 | 0.00 | 0.00 | 0.00 | 0.00 | 0.00 | 0.00 |  |  | 0.13 |
| 4 | IR22 | 20 | WBPH | 2 | 1.00 | 0.00 | 0.00 | 0.00 | 0.00 | 0.00 | 0.00 | 0.00 |  |  | 0.12 |
| 4 | IR22 | 20 | WBPH | 3 | 0.84 | 0.16 | 0.00 | 0.00 | 0.00 | 0.00 | 0.00 | 0.00 |  |  | 0.14 |
| 4 | IR22 | 20 | WBPH | 3 | 0.88 | 0.12 | 0.00 | 0.00 | 0.00 | 0.00 | 0.00 | 0.00 |  |  | 0.15 |
| 4 | IR22 | 20 | WBPH | 3 | 1.00 | 0.00 | 0.00 | 0.00 | 0.00 | 0.00 | 0.00 | 0.00 |  |  | 0.10 |
| 4 | IR22 | 20 | WBPH | 3 | 0.89 | 0.11 | 0.00 | 0.00 | 0.00 | 0.00 | 0.00 | 0.00 |  |  | 0.15 |
| 4 | IR22 | 20 | WBPH | 4 | 0.06 | 0.94 | 0.00 | 0.00 | 0.00 | 0.00 | 0.00 | 0.00 |  |  | 0.14 |
| 4 | IR22 | 20 | WBPH | 4 | 0.05 | 0.95 | 0.00 | 0.00 | 0.00 | 0.00 | 0.00 | 0.00 |  |  | 0.19 |
| 4 | IR22 | 20 | WBPH | 4 | 0.00 | 1.00 | 0.00 | 0.00 | 0.00 | 0.00 | 0.00 | 0.00 |  |  | 0.18 |
| 4 | IR22 | 20 | WBPH | 4 | 0.00 | 1.00 | 0.00 | 0.00 | 0.00 | 0.00 | 0.00 | 0.00 |  |  | 0.17 |
| 4 | IR22 | 20 | WBPH | 5 | 0.00 | 1.00 | 0.00 | 0.00 | 0.00 | 0.00 | 0.00 | 0.00 |  |  | 0.29 |
| 4 | IR22 | 20 | WBPH | 5 | 0.00 | 1.00 | 0.00 | 0.00 | 0.00 | 0.00 | 0.00 | 0.00 |  |  | 0.23 |
| 4 | IR22 | 20 | WBPH | 5 | 0.00 | 1.00 | 0.00 | 0.00 | 0.00 | 0.00 | 0.00 | 0.00 |  |  | 0.28 |
| 4 | IR22 | 20 | WBPH | 5 | 0.00 | 1.00 | 0.00 | 0.00 | 0.00 | 0.00 | 0.00 | 0.00 |  |  | 0.20 |
| 4 | IR22 | 20 | WBPH | 6 | 0.00 | 1.00 | 0.00 | 0.00 | 0.00 | 0.00 | 0.00 | 0.00 |  |  | 0.33 |
| 4 | IR22 | 20 | WBPH | 6 | 0.00 | 1.00 | 0.00 | 0.00 | 0.00 | 0.00 | 0.00 | 0.00 |  |  | 0.27 |
| 4 | IR22 | 20 | WBPH | 6 | 0.00 | 1.00 | 0.00 | 0.00 | 0.00 | 0.00 | 0.00 | 0.00 |  |  | 0.25 |
| 4 | IR22 | 20 | WBPH | 6 | 0.00 | 1.00 | 0.00 | 0.00 | 0.00 | 0.00 | 0.00 | 0.00 |  |  | 0.24 |
| 4 | IR22 | 20 | WBPH | 7 | 0.00 | 0.67 | 0.33 | 0.00 | 0.00 | 0.00 | 0.00 | 0.00 |  |  | 0.33 |
| 4 | IR22 | 20 | WBPH | 7 | 0.00 | 0.60 | 0.40 | 0.00 | 0.00 | 0.00 | 0.00 | 0.00 |  |  | 0.34 |
| 4 | IR22 | 20 | WBPH | 7 | 0.00 | 0.67 | 0.33 | 0.00 | 0.00 | 0.00 | 0.00 | 0.00 |  |  | 0.30 |
| 4 | IR22 | 20 | WBPH | 7 | 0.00 | 0.75 | 0.25 | 0.00 | 0.00 | 0.00 | 0.00 | 0.00 |  |  | 0.32 |
| 4 | IR22 | 20 | WBPH | 8 | 0.00 | 0.11 | 0.84 | 0.05 | 0.00 | 0.00 | 0.00 | 0.00 |  |  | 0.49 |
| 4 | IR22 | 20 | WBPH | 8 | 0.00 | 0.00 | 1.00 | 0.00 | 0.00 | 0.00 | 0.00 | 0.00 |  |  | 0.49 |
| 4 | IR22 | 20 | WBPH | 8 | 0.00 | 0.11 | 0.89 | 0.00 | 0.00 | 0.00 | 0.00 | 0.00 |  |  | 0.42 |
| 4 | IR22 | 20 | WBPH | 8 | 0.00 | 0.10 | 0.90 | 0.00 | 0.00 | 0.00 | 0.00 | 0.00 |  |  | 0.48 |
| 4 | IR22 | 20 | WBPH | 9 | 0.00 | 0.00 | 0.72 | 0.28 | 0.00 | 0.00 | 0.00 | 0.00 |  |  | 0.76 |
| 4 | IR22 | 20 | WBPH | 9 | 0.00 | 0.00 | 0.83 | 0.17 | 0.00 | 0.00 | 0.00 | 0.00 |  |  | 0.62 |
| 4 | IR22 | 20 | WBPH | 9 | 0.00 | 0.00 | 0.70 | 0.30 | 0.00 | 0.00 | 0.00 | 0.00 |  |  | 0.78 |
| 4 | IR22 | 20 | WBPH | 9 | 0.00 | 0.00 | 0.40 | 0.60 | 0.00 | 0.00 | 0.00 | 0.00 |  |  | 1.02 |
| 4 | IR22 | 20 | WBPH | 10 | 0.00 | 0.00 | 0.17 | 0.83 | 0.00 | 0.00 | 0.00 | 0.00 |  |  | 1.00 |
| 4 | IR22 | 20 | WBPH | 10 | 0.00 | 0.00 | 0.33 | 0.67 | 0.00 | 0.00 | 0.00 | 0.00 |  |  | 1.12 |
| 4 | IR22 | 20 | WBPH | 10 | 0.00 | 0.00 | 0.30 | 0.70 | 0.00 | 0.00 | 0.00 | 0.00 |  |  | 1.20 |
| 4 | IR22 | 20 | WBPH | 10 | 0.00 | 0.00 | 0.10 | 0.90 | 0.00 | 0.00 | 0.00 | 0.00 |  |  | 1.16 |
| 4 | IR22 | 20 | WBPH | 11 | 0.00 | 0.00 | 0.00 | 0.89 | 0.11 | 0.00 | 0.00 | 0.00 |  |  | 1.93 |
| 4 | IR22 | 20 | WBPH | 11 | 0.00 | 0.00 | 0.00 | 0.83 | 0.17 | 0.00 | 0.00 | 0.00 |  |  | 1.80 |
| 4 | IR22 | 20 | WBPH | 11 | 0.00 | 0.00 | 0.00 | 1.00 | 0.00 | 0.00 | 0.00 | 0.00 |  |  | 1.70 |
| 4 | IR22 | 20 | WBPH | 11 | 0.00 | 0.00 | 0.00 | 1.00 | 0.00 | 0.00 | 0.00 | 0.00 |  |  | 1.71 |
| 4 | IR22 | 20 | WBPH | 12 | 0.00 | 0.00 | 0.00 | 0.76 | 0.24 | 0.00 | 0.00 | 0.00 |  |  | 1.75 |
| 4 | IR22 | 20 | WBPH | 12 | 0.00 | 0.00 | 0.00 | 0.83 | 0.17 | 0.00 | 0.00 | 0.00 |  |  | 1.79 |
| 4 | IR22 | 20 | WBPH | 12 | 0.00 | 0.00 | 0.00 | 1.00 | 0.00 | 0.00 | 0.00 | 0.00 |  |  | 1.52 |
| 4 | IR22 | 20 | WBPH | 12 | 0.00 | 0.00 | 0.00 | 0.80 | 0.20 | 0.00 | 0.00 | 0.00 |  |  | 2.16 |
| 4 | IR22 | 20 | WBPH | 13 | 0.00 | 0.00 | 0.00 | 0.42 | 0.58 | 0.00 | 0.00 | 0.00 |  |  | 2.08 |
| 4 | IR22 | 20 | WBPH | 13 | 0.00 | 0.00 | 0.00 | 0.37 | 0.63 | 0.00 | 0.00 | 0.00 |  |  | 2.05 |
| 4 | IR22 | 20 | WBPH | 13 | 0.00 | 0.00 | 0.00 | 0.22 | 0.78 | 0.00 | 0.00 | 0.00 |  |  | 2.05 |
| 4 | IR22 | 20 | WBPH | 13 | 0.00 | 0.00 | 0.00 | 0.20 | 0.80 | 0.00 | 0.00 | 0.00 |  |  | 2.71 |
| 4 | IR22 | 20 | WBPH | 14 | 0.00 | 0.00 | 0.00 | 0.06 | 0.94 | 0.00 | 0.00 | 0.00 |  |  | 2.26 |
| 4 | IR22 | 20 | WBPH | 14 | 0.00 | 0.00 | 0.00 | 0.26 | 0.74 | 0.00 | 0.00 | 0.00 |  |  | 2.50 |
| 4 | IR22 | 20 | WBPH | 14 | 0.00 | 0.00 | 0.00 | 0.11 | 0.89 | 0.00 | 0.00 | 0.00 |  |  | 3.10 |
| 4 | IR22 | 20 | WBPH | 14 | 0.00 | 0.00 | 0.00 | 0.30 | 0.70 | 0.00 | 0.00 | 0.00 |  |  | 2.43 |
| 4 | IR22 | 20 | WBPH | 15 | 0.00 | 0.00 | 0.00 | 0.05 | 0.95 | 0.00 | 0.00 | 0.00 |  |  | 3.12 |
| 4 | IR22 | 20 | WBPH | 15 | 0.00 | 0.00 | 0.00 | 0.05 | 0.95 | 0.00 | 0.00 | 0.00 |  |  | 3.50 |
| 4 | IR22 | 20 | WBPH | 15 | 0.00 | 0.00 | 0.00 | 0.00 | 1.00 | 0.00 | 0.00 | 0.00 |  |  | 3.71 |
| 4 | IR22 | 20 | WBPH | 15 | 0.00 | 0.00 | 0.00 | 0.00 | 1.00 | 0.00 | 0.00 | 0.00 |  |  | 5.76 |
| 1 | IR22 | 25 | WBPH | 1 | 0.53 | 0.47 | 0.00 | 0.00 | 0.00 | 0.00 | 0.00 | 0.00 |  |  | 0.10 |
| 2 | IR22 | 25 | WBPH | 1 | 0.75 | 0.25 | 0.00 | 0.00 | 0.00 | 0.00 | 0.00 | 0.00 |  |  | 0.09 |
| 3 | IR22 | 25 | WBPH | 1 | 0.71 | 0.29 | 0.00 | 0.00 | 0.00 | 0.00 | 0.00 | 0.00 |  |  | 0.12 |
| 4 | IR22 | 25 | WBPH | 1 | 0.66 | 0.34 | 0.00 | 0.00 | 0.00 | 0.00 | 0.00 | 0.00 |  |  | 0.11 |
| 1 | IR22 | 25 | WBPH | 2 | 0.06 | 0.56 | 0.39 | 0.00 | 0.00 | 0.00 | 0.00 | 0.00 |  |  | 0.14 |
| 2 | IR22 | 25 | WBPH | 2 | 0.00 | 0.88 | 0.12 | 0.00 | 0.00 | 0.00 | 0.00 | 0.00 |  |  | 0.14 |
| 3 | IR22 | 25 | WBPH | 2 | 0.00 | 0.00 | 1.00 | 0.00 | 0.00 | 0.00 | 0.00 | 0.00 |  |  | 0.14 |
| 4 | IR22 | 25 | WBPH | 2 | 0.02 | 0.48 | 0.50 | 0.00 | 0.00 | 0.00 | 0.00 | 0.00 |  |  | 0.14 |
| 1 | IR22 | 25 | WBPH | 3 | 0.05 | 0.16 | 0.58 | 0.21 | 0.00 | 0.00 | 0.00 | 0.00 |  |  | 0.17 |
| 2 | IR22 | 25 | WBPH | 3 | 0.00 | 0.29 | 0.41 | 0.29 | 0.00 | 0.00 | 0.00 | 0.00 |  |  | 0.18 |
| 3 | IR22 | 25 | WBPH | 3 | 0.00 | 0.26 | 0.68 | 0.05 | 0.00 | 0.00 | 0.00 | 0.00 |  |  | 0.15 |
| 4 | IR22 | 25 | WBPH | 3 | 0.02 | 0.24 | 0.56 | 0.19 | 0.00 | 0.00 | 0.00 | 0.00 |  |  | 0.17 |
| 1 | IR22 | 25 | WBPH | 4 | 0.00 | 0.00 | 0.20 | 0.80 | 0.00 | 0.00 | 0.00 | 0.00 |  |  | 0.19 |
| 2 | IR22 | 25 | WBPH | 4 | 0.00 | 0.00 | 0.05 | 0.75 | 0.20 | 0.00 | 0.00 | 0.00 |  |  | 0.22 |
| 3 | IR22 | 25 | WBPH | 4 | 0.00 | 0.00 | 0.00 | 0.50 | 0.50 | 0.00 | 0.00 | 0.00 |  |  | 0.32 |
| 4 | IR22 | 25 | WBPH | 4 | 0.00 | 0.00 | 0.08 | 0.68 | 0.23 | 0.00 | 0.00 | 0.00 |  |  | 0.25 |
| 1 | IR22 | 25 | WBPH | 5 | 0.00 | 0.00 | 0.19 | 0.44 | 0.38 | 0.00 | 0.00 | 0.00 |  |  | 0.30 |
| 2 | IR22 | 25 | WBPH | 5 | 0.00 | 0.00 | 0.00 | 0.28 | 0.72 | 0.00 | 0.00 | 0.00 |  |  | 0.38 |
| 3 | IR22 | 25 | WBPH | 5 | 0.00 | 0.00 | 0.00 | 0.32 | 0.68 | 0.00 | 0.00 | 0.00 |  |  | 0.37 |
| 4 | IR22 | 25 | WBPH | 5 | 0.00 | 0.00 | 0.06 | 0.34 | 0.59 | 0.00 | 0.00 | 0.00 |  |  | 0.35 |
| 1 | IR22 | 25 | WBPH | 6 | 0.00 | 0.00 | 0.06 | 0.24 | 0.71 | 0.00 | 0.00 | 0.00 |  |  | 0.53 |
| 2 | IR22 | 25 | WBPH | 6 | 0.00 | 0.00 | 0.00 | 0.06 | 0.94 | 0.00 | 0.00 | 0.00 |  |  | 0.51 |
| 3 | IR22 | 25 | WBPH | 6 | 0.00 | 0.00 | 0.00 | 0.00 | 1.00 | 0.00 | 0.00 | 0.00 |  |  | 0.62 |
| 4 | IR22 | 25 | WBPH | 6 | 0.00 | 0.00 | 0.02 | 0.10 | 0.88 | 0.00 | 0.00 | 0.00 |  |  | 0.55 |
| 1 | IR22 | 25 | WBPH | 7 | 0.00 | 0.00 | 0.00 | 0.00 | 1.00 | 0.00 | 0.00 | 0.00 |  |  | 0.60 |
| 2 | IR22 | 25 | WBPH | 7 | 0.00 | 0.00 | 0.00 | 0.00 | 1.00 | 0.00 | 0.00 | 0.00 |  |  | 0.87 |
| 3 | IR22 | 25 | WBPH | 7 | 0.00 | 0.00 | 0.00 | 0.00 | 1.00 | 0.00 | 0.00 | 0.00 |  |  | 0.72 |
| 4 | IR22 | 25 | WBPH | 7 | 0.00 | 0.00 | 0.00 | 0.00 | 1.00 | 0.00 | 0.00 | 0.00 |  |  | 0.73 |
| 1 | IR22 | 25 | WBPH | 8 | 0.00 | 0.00 | 0.00 | 0.00 | 1.00 | 0.00 | 0.00 | 0.00 |  |  | 1.01 |
| 2 | IR22 | 25 | WBPH | 8 | 0.00 | 0.00 | 0.00 | 0.00 | 1.00 | 0.00 | 0.00 | 0.00 |  |  | 1.26 |
| 3 | IR22 | 25 | WBPH | 8 | 0.00 | 0.00 | 0.00 | 0.00 | 1.00 | 0.00 | 0.00 | 0.00 |  |  | 0.97 |
| 4 | IR22 | 25 | WBPH | 8 | 0.00 | 0.00 | 0.00 | 0.00 | 1.00 | 0.00 | 0.00 | 0.00 |  |  | 1.08 |
| 1 | IR22 | 25 | WBPH | 9 | 0.00 | 0.00 | 0.00 | 0.00 | 1.00 | 0.00 | 0.00 | 0.00 |  |  | 1.66 |
| 2 | IR22 | 25 | WBPH | 9 | 0.00 | 0.00 | 0.00 | 0.00 | 1.00 | 0.00 | 0.00 | 0.00 |  |  | 1.89 |
| 3 | IR22 | 25 | WBPH | 9 | 0.00 | 0.00 | 0.00 | 0.00 | 1.00 | 0.00 | 0.00 | 0.00 |  |  | 1.31 |
| 4 | IR22 | 25 | WBPH | 9 | 0.00 | 0.00 | 0.00 | 0.00 | 1.00 | 0.00 | 0.00 | 0.00 |  |  | 1.62 |
| 1 | IR22 | 25 | WBPH | 10 | 0.00 | 0.00 | 0.00 | 0.00 | 1.00 | 0.00 | 0.00 | 0.00 |  |  | 2.55 |
| 2 | IR22 | 25 | WBPH | 10 | 0.00 | 0.00 | 0.00 | 0.00 | 1.00 | 0.00 | 0.00 | 0.00 |  |  | 2.57 |
| 3 | IR22 | 25 | WBPH | 10 | 0.00 | 0.00 | 0.00 | 0.00 | 1.00 | 0.00 | 0.00 | 0.00 |  |  | 2.06 |
| 4 | IR22 | 25 | WBPH | 10 | 0.00 | 0.00 | 0.00 | 0.00 | 1.00 | 0.00 | 0.00 | 0.00 |  |  | 2.39 |
| 1 | IR22 | 25 | WBPH | 11 | 0.00 | 0.00 | 0.00 | 0.00 | 1.00 | 0.00 | 0.00 | 0.00 |  |  | 2.68 |
| 2 | IR22 | 25 | WBPH | 11 | 0.00 | 0.00 | 0.00 | 0.00 | 1.00 | 0.00 | 0.00 | 0.00 |  |  | 2.00 |
| 3 | IR22 | 25 | WBPH | 11 | 0.00 | 0.00 | 0.00 | 0.00 | 1.00 | 0.00 | 0.00 | 0.00 |  |  | 2.57 |
| 4 | IR22 | 25 | WBPH | 11 | 0.00 | 0.00 | 0.00 | 0.00 | 1.00 | 0.00 | 0.00 | 0.00 |  |  | 2.42 |
| 1 | IR22 | 25 | WBPH | 12 | 0.00 | 0.00 | 0.00 | 0.00 | 0.76 | 0.18 | 1.50 | 0.05 | 1.00 | 0.00 | 3.11 |
| 2 | IR22 | 25 | WBPH | 12 | 0.00 | 0.00 | 0.00 | 0.00 | 0.89 | 0.11 | 0.50 | 0.50 | 1.00 | 0.00 | 2.29 |
| 3 | IR22 | 25 | WBPH | 12 | 0.00 | 0.00 | 0.00 | 0.00 | 1.00 | 0.00 |  |  |  |  | 2.38 |
| 4 | IR22 | 25 | WBPH | 12 | 0.00 | 0.00 | 0.00 | 0.00 | 0.88 | 0.10 | 1.00 | 0.28 | 1.00 | 0.00 | 2.59 |
| 1 | IR22 | 25 | WBPH | 13 | 0.00 | 0.00 | 0.00 | 0.00 | 0.35 | 0.65 | 4.00 | 1.50 | 0.75 | 0.00 | 3.11 |
| 2 | IR22 | 25 | WBPH | 13 | 0.00 | 0.00 | 0.00 | 0.00 | 0.50 | 0.50 | 2.00 | 2.50 | 1.00 | 0.00 | 3.36 |
| 3 | IR22 | 25 | WBPH | 13 | 0.00 | 0.00 | 0.00 | 0.00 | 0.42 | 0.58 | 2.00 | 3.50 | 0.75 | 0.00 | 2.91 |
| 4 | IR22 | 25 | WBPH | 13 | 0.00 | 0.00 | 0.00 | 0.00 | 0.42 | 0.58 | 2.67 | 2.50 | 0.83 | 0.00 | 3.13 |
| 1 | IR22 | 25 | WBPH | 14 | 0.00 | 0.00 | 0.00 | 0.00 | 0.16 | 0.84 | 3.00 | 5.00 | 0.33 | 0.00 | 3.29 |
| 2 | IR22 | 25 | WBPH | 14 | 0.00 | 0.00 | 0.00 | 0.00 | 0.26 | 0.74 | 4.00 | 3.00 | 0.88 | 0.00 | 3.05 |
| 3 | IR22 | 25 | WBPH | 14 | 0.00 | 0.00 | 0.00 | 0.00 | 0.53 | 0.47 | 1.50 | 2.50 | 1.00 | 0.00 | 2.55 |
| 4 | IR22 | 25 | WBPH | 14 | 0.00 | 0.00 | 0.00 | 0.00 | 0.32 | 0.68 | 2.83 | 3.50 | 0.74 | 0.00 | 2.96 |
| 1 | IR22 | 25 | WBPH | 15 | 0.00 | 0.00 | 0.00 | 0.00 | 0.29 | 0.71 | 3.00 | 3.00 | 0.33 | 0.00 | 2.87 |
| 2 | IR22 | 25 | WBPH | 15 | 0.00 | 0.00 | 0.00 | 0.00 | 0.30 | 0.70 | 1.00 | 2.50 | 1.00 | 0.00 | 1.22 |
| 3 | IR22 | 25 | WBPH | 15 | 0.00 | 0.00 | 0.00 | 0.00 | 0.50 | 0.50 | 3.50 | 0.00 | 0.43 |  | 2.38 |
| 4 | IR22 | 25 | WBPH | 15 | 0.00 | 0.00 | 0.00 | 0.00 | 0.36 | 0.64 | 2.50 | 1.83 | 0.59 | 0.00 | 2.15 |
| 1 | IR22 | 30 | WBPH | 1 | 0.38 | 0.56 | 0.06 | 0.00 | 0.00 | 0.00 | 0.00 | 0.00 |  |  | 0.09 |
| 2 | IR22 | 30 | WBPH | 1 | 1.00 | 0.00 | 0.00 | 0.00 | 0.00 | 0.00 | 0.00 | 0.00 |  |  | 0.11 |
| 3 | IR22 | 30 | WBPH | 1 | 0.53 | 0.47 | 0.00 | 0.00 | 0.00 | 0.00 | 0.00 | 0.00 |  |  | 0.13 |
| 4 | IR22 | 30 | WBPH | 1 | 0.63 | 0.35 | 0.02 | 0.00 | 0.00 | 0.00 | 0.00 | 0.00 |  |  | 0.11 |
| 1 | IR22 | 30 | WBPH | 2 | 0.00 | 0.45 | 0.55 | 0.00 | 0.00 | 0.00 | 0.00 | 0.00 |  |  | 0.17 |
| 2 | IR22 | 30 | WBPH | 2 | 0.05 | 0.42 | 0.53 | 0.00 | 0.00 | 0.00 | 0.00 | 0.00 |  |  | 0.17 |
| 3 | IR22 | 30 | WBPH | 2 | 0.00 | 0.11 | 0.89 | 0.00 | 0.00 | 0.00 | 0.00 | 0.00 |  |  | 0.14 |
| 4 | IR22 | 30 | WBPH | 2 | 0.02 | 0.33 | 0.66 | 0.00 | 0.00 | 0.00 | 0.00 | 0.00 |  |  | 0.16 |
| 1 | IR22 | 30 | WBPH | 3 | 0.00 | 0.20 | 0.53 | 0.27 | 0.00 | 0.00 | 0.00 | 0.00 |  |  | 0.24 |
| 2 | IR22 | 30 | WBPH | 3 | 0.00 | 0.00 | 0.21 | 0.79 | 0.00 | 0.00 | 0.00 | 0.00 |  |  | 0.31 |
| 3 | IR22 | 30 | WBPH | 3 | 0.00 | 0.00 | 0.56 | 0.44 | 0.00 | 0.00 | 0.00 | 0.00 |  |  | 0.21 |
| 4 | IR22 | 30 | WBPH | 3 | 0.00 | 0.07 | 0.43 | 0.50 | 0.00 | 0.00 | 0.00 | 0.00 |  |  | 0.25 |
| 1 | IR22 | 30 | WBPH | 4 | 0.00 | 0.06 | 0.29 | 0.47 | 0.18 | 0.00 | 0.00 | 0.00 |  |  | 0.26 |
| 2 | IR22 | 30 | WBPH | 4 | 0.00 | 0.00 | 0.10 | 0.55 | 0.35 | 0.00 | 0.00 | 0.00 |  |  | 0.38 |
| 3 | IR22 | 30 | WBPH | 4 | 0.00 | 0.00 | 0.17 | 0.44 | 0.39 | 0.00 | 0.00 | 0.00 |  |  | 0.33 |
| 4 | IR22 | 30 | WBPH | 4 | 0.00 | 0.02 | 0.19 | 0.49 | 0.31 | 0.00 | 0.00 | 0.00 |  |  | 0.32 |
| 1 | IR22 | 30 | WBPH | 5 | 0.00 | 0.00 | 0.11 | 0.61 | 0.28 | 0.00 | 0.00 | 0.00 |  |  | 0.40 |
| 2 | IR22 | 30 | WBPH | 5 | 0.00 | 0.00 | 0.00 | 0.07 | 0.93 | 0.00 | 0.00 | 0.00 |  |  | 0.46 |
| 3 | IR22 | 30 | WBPH | 5 | 0.00 | 0.00 | 0.00 | 0.10 | 0.90 | 0.00 | 0.00 | 0.00 |  |  | 0.58 |
| 4 | IR22 | 30 | WBPH | 5 | 0.00 | 0.00 | 0.04 | 0.26 | 0.70 | 0.00 | 0.00 | 0.00 |  |  | 0.48 |
| 1 | IR22 | 30 | WBPH | 6 | 0.00 | 0.00 | 0.00 | 0.13 | 0.87 | 0.00 | 0.00 | 0.00 |  |  | 0.55 |
| 2 | IR22 | 30 | WBPH | 6 | 0.00 | 0.00 | 0.00 | 0.06 | 0.94 | 0.00 | 0.00 | 0.00 |  |  | 0.59 |
| 3 | IR22 | 30 | WBPH | 6 | 0.00 | 0.00 | 0.00 | 0.07 | 0.93 | 0.00 | 0.00 | 0.00 |  |  | 0.55 |
| 4 | IR22 | 30 | WBPH | 6 | 0.00 | 0.00 | 0.00 | 0.09 | 0.91 | 0.00 | 0.00 | 0.00 |  |  | 0.56 |
| 1 | IR22 | 30 | WBPH | 7 | 0.00 | 0.00 | 0.00 | 0.25 | 0.75 | 0.00 | 0.00 | 0.00 |  |  | 0.47 |
| 2 | IR22 | 30 | WBPH | 7 | 0.00 | 0.00 | 0.00 | 0.05 | 0.95 | 0.00 | 0.00 | 0.00 |  |  | 0.88 |
| 3 | IR22 | 30 | WBPH | 7 | 0.00 | 0.00 | 0.00 | 0.00 | 1.00 | 0.00 | 0.00 | 0.00 |  |  | 0.73 |
| 4 | IR22 | 30 | WBPH | 7 | 0.00 | 0.00 | 0.00 | 0.10 | 0.90 | 0.00 | 0.00 | 0.00 |  |  | 0.69 |
| 1 | IR22 | 30 | WBPH | 8 | 0.00 | 0.00 | 0.00 | 0.07 | 0.93 | 0.00 | 0.00 | 0.00 |  |  | 0.74 |
| 2 | IR22 | 30 | WBPH | 8 | 0.00 | 0.00 | 0.00 | 0.00 | 1.00 | 0.00 | 0.00 | 0.00 |  |  | 1.18 |
| 3 | IR22 | 30 | WBPH | 8 | 0.00 | 0.00 | 0.00 | 0.00 | 1.00 | 0.00 | 0.00 | 0.00 |  |  | 1.07 |
| 4 | IR22 | 30 | WBPH | 8 | 0.00 | 0.00 | 0.00 | 0.02 | 0.98 | 0.00 | 0.00 | 0.00 |  |  | 1.00 |
| 1 | IR22 | 30 | WBPH | 9 | 0.00 | 0.00 | 0.00 | 0.17 | 0.83 | 0.00 | 0.00 | 0.00 |  |  | 0.90 |
| 2 | IR22 | 30 | WBPH | 9 | 0.00 | 0.00 | 0.00 | 0.00 | 1.00 | 0.00 | 0.00 | 0.00 |  |  | 1.27 |
| 3 | IR22 | 30 | WBPH | 9 | 0.00 | 0.00 | 0.00 | 0.00 | 1.00 | 0.00 | 0.00 | 0.00 |  |  | 1.19 |
| 4 | IR22 | 30 | WBPH | 9 | 0.00 | 0.00 | 0.00 | 0.06 | 0.94 | 0.00 | 0.00 | 0.00 |  |  | 1.12 |
| 1 | IR22 | 30 | WBPH | 10 | 0.00 | 0.00 | 0.00 | 0.00 | 0.95 | 0.05 | 0.00 | 0.50 |  | 0.00 | 1.44 |
| 2 | IR22 | 30 | WBPH | 10 | 0.00 | 0.00 | 0.00 | 0.00 | 1.00 | 0.00 | 0.00 | 0.00 |  |  | 1.24 |
| 3 | IR22 | 30 | WBPH | 10 | 0.00 | 0.00 | 0.00 | 0.00 | 0.94 | 0.06 | 0.00 | 0.50 |  | 0.00 | 1.46 |
| 4 | IR22 | 30 | WBPH | 10 | 0.00 | 0.00 | 0.00 | 0.00 | 0.96 | 0.04 | 0.00 | 0.33 |  | 0.00 | 1.38 |
| 1 | IR22 | 30 | WBPH | 11 | 0.00 | 0.00 | 0.00 | 0.00 | 1.00 | 0.00 | 0.00 | 0.00 |  |  | 1.47 |
| 2 | IR22 | 30 | WBPH | 11 | 0.00 | 0.00 | 0.00 | 0.00 | 0.95 | 0.05 | 0.00 | 0.50 |  | 0.00 | 1.56 |
| 3 | IR22 | 30 | WBPH | 11 | 0.00 | 0.00 | 0.00 | 0.00 | 0.93 | 0.07 | 0.00 | 0.50 |  | 0.00 | 1.25 |
| 4 | IR22 | 30 | WBPH | 11 | 0.00 | 0.00 | 0.00 | 0.00 | 0.96 | 0.04 | 0.00 | 0.33 |  | 0.00 | 1.43 |
| 1 | IR22 | 30 | WBPH | 12 | 0.00 | 0.00 | 0.00 | 0.00 | 0.88 | 0.12 | 0.00 | 1.00 |  | 0.00 | 1.38 |
| 2 | IR22 | 30 | WBPH | 12 | 0.00 | 0.00 | 0.00 | 0.00 | 1.00 | 0.00 | 0.00 | 0.00 |  |  | 1.08 |
| 3 | IR22 | 30 | WBPH | 12 | 0.00 | 0.00 | 0.00 | 0.00 | 0.90 | 0.10 | 0.50 | 0.50 | 1.00 | 0.00 | 1.69 |
| 4 | IR22 | 30 | WBPH | 12 | 0.00 | 0.00 | 0.00 | 0.00 | 0.93 | 0.07 | 0.17 | 0.50 | 1.00 | 0.00 | 1.38 |
| 1 | IR22 | 30 | WBPH | 13 | 0.00 | 0.00 | 0.00 | 0.00 | 1.00 | 0.00 | 0.00 | 0.00 |  |  | 1.57 |
| 2 | IR22 | 30 | WBPH | 13 | 0.00 | 0.00 | 0.00 | 0.00 | 0.92 | 0.08 | 0.00 | 0.50 |  | 0.00 | 1.22 |
| 3 | IR22 | 30 | WBPH | 13 | 0.00 | 0.00 | 0.00 | 0.00 | 0.95 | 0.05 | 0.50 | 0.00 | 1.00 |  | 1.51 |
| 4 | IR22 | 30 | WBPH | 13 | 0.00 | 0.00 | 0.00 | 0.00 | 0.96 | 0.04 | 0.17 | 0.17 | 1.00 | 0.00 | 1.43 |
| 1 | IR22 | 30 | WBPH | 14 | 0.00 | 0.00 | 0.00 | 0.00 | 0.83 | 0.17 | 0.50 | 0.50 | 1.00 | 0.00 | 1.18 |
| 2 | IR22 | 30 | WBPH | 14 | 0.00 | 0.00 | 0.00 | 0.00 | 0.75 | 0.25 | 0.50 | 0.50 | 1.00 | 1.00 | 0.84 |
| 3 | IR22 | 30 | WBPH | 14 | 0.00 | 0.00 | 0.00 | 0.00 | 1.00 | 0.00 | 0.00 | 0.00 |  |  | 1.50 |
| 4 | IR22 | 30 | WBPH | 14 | 0.00 | 0.00 | 0.00 | 0.00 | 0.86 | 0.14 | 0.33 | 0.33 | 1.00 | 0.50 | 1.18 |
| 1 | IR22 | 30 | WBPH | 15 | 0.00 | 0.00 | 0.00 | 0.00 | 1.00 | 0.00 | 0.00 | 0.00 |  |  | 0.92 |
| 2 | IR22 | 30 | WBPH | 15 | 0.00 | 0.00 | 0.00 | 0.00 | 0.87 | 0.13 | 0.50 | 0.50 | 1.00 | 0.00 | 1.44 |
| 3 | IR22 | 30 | WBPH | 15 | 0.00 | 0.00 | 0.00 | 0.00 | 0.94 | 0.06 | 0.50 | 0.00 | 1.00 |  | 1.11 |
| 4 | IR22 | 30 | WBPH | 15 | 0.00 | 0.00 | 0.00 | 0.00 | 0.94 | 0.06 | 0.33 | 0.17 | 1.00 | 0.00 | 1.15 |
| 1 | IR22 | 35 | WBPH | 1 | 0.45 | 0.27 | 0.27 | 0.00 | 0.00 | 0.00 | 0.00 | 0.00 |  |  | 0.06 |
| 2 | IR22 | 35 | WBPH | 1 | 0.10 | 0.50 | 0.40 | 0.00 | 0.00 | 0.00 | 0.00 | 0.00 |  |  | 0.07 |
| 3 | IR22 | 35 | WBPH | 1 | 0.33 | 0.42 | 0.25 | 0.00 | 0.00 | 0.00 | 0.00 | 0.00 |  |  | 0.08 |
| 4 | IR22 | 35 | WBPH | 1 | 1.00 | 0.00 | 0.00 | 0.00 | 0.00 | 0.00 | 0.00 | 0.00 |  |  | 0.06 |
| 1 | IR22 | 35 | WBPH | 2 | 0.06 | 0.25 | 0.56 | 0.13 | 0.00 | 0.00 | 0.00 | 0.00 |  |  | 0.08 |
| 2 | IR22 | 35 | WBPH | 2 | 0.00 | 0.13 | 0.87 | 0.00 | 0.00 | 0.00 | 0.00 | 0.00 |  |  | 0.07 |
| 3 | IR22 | 35 | WBPH | 2 | 0.07 | 0.27 | 0.67 | 0.00 | 0.00 | 0.00 | 0.00 | 0.00 |  |  | 0.07 |
| 4 | IR22 | 35 | WBPH | 2 | 0.90 | 0.10 | 0.00 | 0.00 | 0.00 | 0.00 | 0.00 | 0.00 |  |  | 0.09 |
| 1 | IR22 | 35 | WBPH | 3 | 0.09 | 0.18 | 0.73 | 0.00 | 0.00 | 0.00 | 0.00 | 0.00 |  |  | 0.06 |
| 2 | IR22 | 35 | WBPH | 3 | 0.00 | 0.00 | 0.64 | 0.36 | 0.00 | 0.00 | 0.00 | 0.00 |  |  | 0.09 |
| 3 | IR22 | 35 | WBPH | 3 | 0.00 | 0.00 | 0.18 | 0.82 | 0.00 | 0.00 | 0.00 | 0.00 |  |  | 0.07 |
| 4 | IR22 | 35 | WBPH | 3 | 0.54 | 0.46 | 0.00 | 0.00 | 0.00 | 0.00 | 0.00 | 0.00 |  |  | 0.03 |
| 1 | IR22 | 35 | WBPH | 4 | 0.00 | 0.00 | 0.56 | 0.44 | 0.00 | 0.00 | 0.00 | 0.00 |  |  | 0.10 |
| 2 | IR22 | 35 | WBPH | 4 | 0.00 | 0.00 | 0.00 | 0.38 | 0.62 | 0.00 | 0.00 | 0.00 |  |  | 0.09 |
| 3 | IR22 | 35 | WBPH | 4 | 0.00 | 0.00 | 0.00 | 0.33 | 0.67 | 0.00 | 0.00 | 0.00 |  |  | 0.08 |
| 4 | IR22 | 35 | WBPH | 4 | 0.26 | 0.74 | 0.00 | 0.00 | 0.00 | 0.00 | 0.00 | 0.00 |  |  | 0.08 |
| 1 | IR22 | 35 | WBPH | 5 | 0.00 | 0.13 | 0.63 | 0.25 | 0.00 | 0.00 | 0.00 | 0.00 |  |  | 0.11 |
| 2 | IR22 | 35 | WBPH | 5 | 0.00 | 0.00 | 0.00 | 0.25 | 0.75 | 0.00 | 0.00 | 0.00 |  |  | 0.10 |
| 3 | IR22 | 35 | WBPH | 5 | 0.00 | 0.00 | 0.00 | 0.22 | 0.78 | 0.00 | 0.00 | 0.00 |  |  | 0.10 |
| 4 | IR22 | 35 | WBPH | 5 | 0.43 | 0.57 | 0.00 | 0.00 | 0.00 | 0.00 | 0.00 | 0.00 |  |  | 0.11 |
| 1 | IR22 | 35 | WBPH | 6 | 0.00 | 0.00 | 0.06 | 0.75 | 0.19 | 0.00 | 0.00 | 0.00 |  |  | 0.11 |
| 2 | IR22 | 35 | WBPH | 6 | 0.00 | 0.00 | 0.00 | 0.15 | 0.85 | 0.00 | 0.00 | 0.00 |  |  | 0.07 |
| 3 | IR22 | 35 | WBPH | 6 | 0.00 | 0.00 | 0.00 | 0.14 | 0.86 | 0.00 | 0.00 | 0.00 |  |  | 0.11 |
| 4 | IR22 | 35 | WBPH | 6 | 0.03 | 0.94 | 0.03 | 0.00 | 0.00 | 0.00 | 0.00 | 0.00 |  |  | 0.13 |
| 1 | IR22 | 35 | WBPH | 7 | 0.00 | 0.00 | 0.08 | 0.42 | 0.50 | 0.00 | 0.00 | 0.00 |  |  | 0.09 |
| 2 | IR22 | 35 | WBPH | 7 | 0.00 | 0.00 | 0.00 | 0.00 | 1.00 | 0.00 | 0.00 | 0.00 |  |  | 0.13 |
| 3 | IR22 | 35 | WBPH | 7 | 0.00 | 0.00 | 0.00 | 0.00 | 1.00 | 0.00 | 0.00 | 0.00 |  |  | 0.15 |
| 4 | IR22 | 35 | WBPH | 7 | 0.11 | 0.80 | 0.09 | 0.00 | 0.00 | 0.00 | 0.00 | 0.00 |  |  | 0.16 |
| 1 | IR22 | 35 | WBPH | 8 | 0.00 | 0.00 | 0.08 | 0.38 | 0.54 | 0.00 | 0.00 | 0.00 |  |  | 0.08 |
| 2 | IR22 | 35 | WBPH | 8 | 0.00 | 0.00 | 0.00 | 0.00 | 1.00 | 0.00 | 0.00 | 0.00 |  |  | 0.06 |
| 3 | IR22 | 35 | WBPH | 8 | 0.00 | 0.00 | 0.00 | 0.00 | 1.00 | 0.00 | 0.00 | 0.00 |  |  | 0.12 |
| 4 | IR22 | 35 | WBPH | 8 | 0.00 | 0.76 | 0.24 | 0.00 | 0.00 | 0.00 | 0.00 | 0.00 |  |  | 0.11 |
| 1 | IR22 | 35 | WBPH | 9 | 0.00 | 0.00 | 0.00 | 0.20 | 0.80 | 0.00 | 0.00 | 0.00 |  |  | 0.07 |
| 2 | IR22 | 35 | WBPH | 9 | 0.00 | 0.00 | 0.00 | 0.00 | 1.00 | 0.00 | 0.00 | 0.00 |  |  | 0.14 |
| 3 | IR22 | 35 | WBPH | 9 | 0.00 | 0.00 | 0.00 | 0.00 | 1.00 | 0.00 | 0.00 | 0.00 |  |  | 0.05 |
| 4 | IR22 | 35 | WBPH | 9 | 0.00 | 0.85 | 0.15 | 0.00 | 0.00 | 0.00 | 0.00 | 0.00 |  |  | 0.04 |
| 1 | IR22 | 35 | WBPH | 10 | 0.00 | 0.00 | 0.00 | 0.00 | 1.00 | 0.00 | 0.00 | 0.00 |  |  | 0.08 |
| 2 | IR22 | 35 | WBPH | 10 | 0.00 | 0.00 | 0.00 | 0.00 | 1.00 | 0.00 | 0.00 | 0.00 |  |  | 0.04 |
| 3 | IR22 | 35 | WBPH | 10 | 0.00 | 0.00 | 0.00 | 0.00 | 1.00 | 0.00 | 0.00 | 0.00 |  |  | 0.06 |
| 4 | IR22 | 35 | WBPH | 10 | 0.00 | 0.00 | 1.00 | 0.00 | 0.00 | 0.00 | 0.00 | 0.00 |  |  | 0.00 |
| 1 | IR22 | 35 | WBPH | 11 | 0.00 | 0.00 | 0.17 | 0.17 | 0.67 | 0.00 | 0.00 | 0.00 |  |  | 0.07 |
| 2 | IR22 | 35 | WBPH | 11 | 0.00 | 0.00 | 0.00 | 0.00 | 1.00 | 0.00 | 0.00 | 0.00 |  |  | 0.08 |
| 3 | IR22 | 35 | WBPH | 11 | 0.00 | 0.00 | 0.00 | 0.00 | 1.00 | 0.00 | 0.00 | 0.00 |  |  | 0.04 |
| 4 | IR22 | 35 | WBPH | 11 |  |  |  |  |  |  | 0.00 | 0.00 |  |  | 0.00 |
| 1 | IR22 | 35 | WBPH | 12 | 0.00 | 0.00 | 0.00 | 0.00 | 1.00 | 0.00 | 0.00 | 0.00 |  |  | 0.03 |
| 2 | IR22 | 35 | WBPH | 12 | 0.00 | 0.00 | 0.00 | 0.00 | 1.00 | 0.00 | 0.00 | 0.00 |  |  | 0.08 |
| 3 | IR22 | 35 | WBPH | 12 | 0.00 | 0.00 | 0.00 | 0.00 | 1.00 | 0.00 | 0.00 | 0.00 |  |  | 0.00 |
| 4 | IR22 | 35 | WBPH | 12 |  |  |  |  |  |  | 0.00 | 0.00 |  |  | 0.00 |
| 1 | IR22 | 35 | WBPH | 13 |  |  |  |  |  |  | 0.00 | 0.00 |  |  | 0.00 |
| 2 | IR22 | 35 | WBPH | 13 |  |  |  |  |  |  | 0.00 | 0.00 |  |  | 0.00 |
| 3 | IR22 | 35 | WBPH | 13 |  |  |  |  |  |  | 0.00 | 0.00 |  |  | 0.00 |
| 4 | IR22 | 35 | WBPH | 13 |  |  |  |  |  |  | 0.00 | 0.00 |  |  | 0.00 |
| 1 | IR22 | 35 | WBPH | 14 |  |  |  |  |  |  | 0.00 | 0.00 |  |  | 0.00 |
| 2 | IR22 | 35 | WBPH | 14 | 0.00 | 0.00 | 0.00 | 0.00 | 1.00 | 0.00 | 0.00 | 0.00 |  |  | 0.04 |
| 3 | IR22 | 35 | WBPH | 14 |  |  |  |  |  |  | 0.00 | 0.00 |  |  | 0.00 |
| 4 | IR22 | 35 | WBPH | 14 |  |  |  |  |  |  | 0.00 | 0.00 |  |  | 0.00 |
| 1 | IR22 | 35 | WBPH | 15 |  |  |  |  |  |  | 0.00 | 0.00 |  |  | 0.00 |
| 2 | IR22 | 35 | WBPH | 15 |  |  |  |  |  |  | 0.00 | 0.00 |  |  | 0.00 |
| 3 | IR22 | 35 | WBPH | 15 |  |  |  |  |  |  | 0.00 | 0.00 |  |  | 0.00 |
| 4 | IR22 | 35 | WBPH | 15 |  |  |  |  |  |  | 0.00 | 0.00 |  |  | 0.00 |
| 4 | IR22 | 40 | WBPH | 1 | 1.00 | 0.00 | 0.00 | 0.00 | 0.00 | 0.00 | 0.00 | 0.00 |  |  | 0.04 |
| 4 | IR22 | 40 | WBPH | 1 | 0.75 | 0.00 | 0.00 | 0.00 | 0.00 | 0.00 | 0.00 | 0.00 |  |  | 0.06 |
| 4 | IR22 | 40 | WBPH | 1 | 3.00 | 0.00 | 0.00 | 0.00 | 0.00 | 0.00 | 0.00 | 0.00 |  |  | 0.02 |
| 4 | IR22 | 40 | WBPH | 1 | 3.00 | 0.00 | 0.00 | 0.00 | 0.00 | 0.00 | 0.00 | 0.00 |  |  | 0.01 |
| 4 | IR22 | 40 | WBPH | 2 |  |  |  |  |  |  | 0.00 | 0.00 |  |  | 0.00 |
| 4 | IR22 | 40 | WBPH | 2 |  |  |  |  |  |  | 0.00 | 0.00 |  |  | 0.00 |
| 4 | IR22 | 40 | WBPH | 2 |  |  |  |  |  |  | 0.00 | 0.00 |  |  | 0.00 |
| 4 | IR22 | 40 | WBPH | 2 |  |  |  |  |  |  | 0.00 | 0.00 |  |  | 0.00 |
| 4 | IR22 | 40 | WBPH | 3 |  |  |  |  |  |  | 0.00 | 0.00 |  |  | 0.00 |
| 4 | IR22 | 40 | WBPH | 3 |  |  |  |  |  |  | 0.00 | 0.00 |  |  | 0.00 |
| 4 | IR22 | 40 | WBPH | 3 |  |  |  |  |  |  | 0.00 | 0.00 |  |  | 0.00 |
| 4 | IR22 | 40 | WBPH | 3 |  |  |  |  |  |  | 0.00 | 0.00 |  |  | 0.00 |
| 4 | IR22 | 40 | WBPH | 4 |  |  |  |  |  |  | 0.00 | 0.00 |  |  | 0.00 |
| 4 | IR22 | 40 | WBPH | 4 |  |  |  |  |  |  | 0.00 | 0.00 |  |  | 0.00 |
| 4 | IR22 | 40 | WBPH | 4 |  |  |  |  |  |  | 0.00 | 0.00 |  |  | 0.00 |
| 4 | IR22 | 40 | WBPH | 4 |  |  |  |  |  |  | 0.00 | 0.00 |  |  | 0.00 |
| 4 | IR22 | 40 | WBPH | 5 |  |  |  |  |  |  | 0.00 | 0.00 |  |  | 0.00 |
| 4 | IR22 | 40 | WBPH | 5 |  |  |  |  |  |  | 0.00 | 0.00 |  |  | 0.00 |
| 4 | IR22 | 40 | WBPH | 5 |  |  |  |  |  |  | 0.00 | 0.00 |  |  | 0.00 |
| 4 | IR22 | 40 | WBPH | 5 |  |  |  |  |  |  | 0.00 | 0.00 |  |  | 0.00 |
| 4 | IR22 | 40 | WBPH | 6 |  |  |  |  |  |  | 0.00 | 0.00 |  |  | 0.00 |
| 4 | IR22 | 40 | WBPH | 6 |  |  |  |  |  |  | 0.00 | 0.00 |  |  | 0.00 |
| 4 | IR22 | 40 | WBPH | 6 |  |  |  |  |  |  | 0.00 | 0.00 |  |  | 0.00 |
| 4 | IR22 | 40 | WBPH | 6 |  |  |  |  |  |  | 0.00 | 0.00 |  |  | 0.00 |
| 4 | IR22 | 40 | WBPH | 7 |  |  |  |  |  |  | 0.00 | 0.00 |  |  | 0.00 |
| 4 | IR22 | 40 | WBPH | 7 |  |  |  |  |  |  | 0.00 | 0.00 |  |  | 0.00 |
| 4 | IR22 | 40 | WBPH | 7 |  |  |  |  |  |  | 0.00 | 0.00 |  |  | 0.00 |
| 4 | IR22 | 40 | WBPH | 7 |  |  |  |  |  |  | 0.00 | 0.00 |  |  | 0.00 |
| 4 | IR22 | 40 | WBPH | 8 |  |  |  |  |  |  | 0.00 | 0.00 |  |  | 0.00 |
| 4 | IR22 | 40 | WBPH | 8 |  |  |  |  |  |  | 0.00 | 0.00 |  |  | 0.00 |
| 4 | IR22 | 40 | WBPH | 8 |  |  |  |  |  |  | 0.00 | 0.00 |  |  | 0.00 |
| 4 | IR22 | 40 | WBPH | 8 |  |  |  |  |  |  | 0.00 | 0.00 |  |  | 0.00 |
| 4 | IR22 | 40 | WBPH | 9 |  |  |  |  |  |  | 0.00 | 0.00 |  |  | 0.00 |
| 4 | IR22 | 40 | WBPH | 9 |  |  |  |  |  |  | 0.00 | 0.00 |  |  | 0.00 |
| 4 | IR22 | 40 | WBPH | 9 |  |  |  |  |  |  | 0.00 | 0.00 |  |  | 0.00 |
| 4 | IR22 | 40 | WBPH | 9 |  |  |  |  |  |  | 0.00 | 0.00 |  |  | 0.00 |
| 4 | IR22 | 40 | WBPH | 10 |  |  |  |  |  |  | 0.00 | 0.00 |  |  | 0.00 |
| 4 | IR22 | 40 | WBPH | 10 |  |  |  |  |  |  | 0.00 | 0.00 |  |  | 0.00 |
| 4 | IR22 | 40 | WBPH | 10 |  |  |  |  |  |  | 0.00 | 0.00 |  |  | 0.00 |
| 4 | IR22 | 40 | WBPH | 10 |  |  |  |  |  |  | 0.00 | 0.00 |  |  | 0.00 |
| 4 | IR22 | 40 | WBPH | 11 |  |  |  |  |  |  | 0.00 | 0.00 |  |  | 0.00 |
| 4 | IR22 | 40 | WBPH | 11 |  |  |  |  |  |  | 0.00 | 0.00 |  |  | 0.00 |
| 4 | IR22 | 40 | WBPH | 11 |  |  |  |  |  |  | 0.00 | 0.00 |  |  | 0.00 |
| 4 | IR22 | 40 | WBPH | 11 |  |  |  |  |  |  | 0.00 | 0.00 |  |  | 0.00 |
| 4 | IR22 | 40 | WBPH | 12 |  |  |  |  |  |  | 0.00 | 0.00 |  |  | 0.00 |
| 4 | IR22 | 40 | WBPH | 12 |  |  |  |  |  |  | 0.00 | 0.00 |  |  | 0.00 |
| 4 | IR22 | 40 | WBPH | 12 |  |  |  |  |  |  | 0.00 | 0.00 |  |  | 0.00 |
| 4 | IR22 | 40 | WBPH | 12 |  |  |  |  |  |  | 0.00 | 0.00 |  |  | 0.00 |
| 4 | IR22 | 40 | WBPH | 13 |  |  |  |  |  |  | 0.00 | 0.00 |  |  | 0.00 |
| 4 | IR22 | 40 | WBPH | 13 |  |  |  |  |  |  | 0.00 | 0.00 |  |  | 0.00 |
| 4 | IR22 | 40 | WBPH | 13 |  |  |  |  |  |  | 0.00 | 0.00 |  |  | 0.00 |
| 4 | IR22 | 40 | WBPH | 13 |  |  |  |  |  |  | 0.00 | 0.00 |  |  | 0.00 |
| 4 | IR22 | 40 | WBPH | 14 |  |  |  |  |  |  | 0.00 | 0.00 |  |  | 0.00 |
| 4 | IR22 | 40 | WBPH | 14 |  |  |  |  |  |  | 0.00 | 0.00 |  |  | 0.00 |
| 4 | IR22 | 40 | WBPH | 14 |  |  |  |  |  |  | 0.00 | 0.00 |  |  | 0.00 |
| 4 | IR22 | 40 | WBPH | 14 |  |  |  |  |  |  | 0.00 | 0.00 |  |  | 0.00 |
| 4 | IR22 | 40 | WBPH | 15 |  |  |  |  |  |  | 0.00 | 0.00 |  |  | 0.00 |
| 4 | IR22 | 40 | WBPH | 15 |  |  |  |  |  |  | 0.00 | 0.00 |  |  | 0.00 |
| 4 | IR22 | 40 | WBPH | 15 |  |  |  |  |  |  | 0.00 | 0.00 |  |  | 0.00 |
| 4 | IR22 | 40 | WBPH | 15 |  |  |  |  |  |  | 0.00 | 0.00 |  |  | 0.00 |
|  |  |  |  |  |  |  |  |  |  |  |  |  |  |  |  |
