# Supplementary material for: Temperature-dependent oviposition and nymph performance reveal distinct thermal niches of coexisting planthoppers with similar thresholds for development
Source: PLoS One. 2020 Jun 30;15(6):e0235506. doi: 10.1371/journal.pone.0235506 (PMC7326231; doi:10.1371/journal.pone.0235506)
Supplement: S6 Table — (DOCX) [file pone.0235506.s007.docx]

**Table S6. Data from environmental chamber studies of responses by planthopper nymphs to temperature**

| Day of observation | Species | Rice variety | initial age of plant | Temperature (oC) | Run | Number of subreplicates | Number of egg batches | Total number of eggs | % survival |
| --- | --- | --- | --- | --- | --- | --- | --- | --- | --- |
| 1 | BPH | IR22 | 20 | 15 | 1 | 5 | 0.40 | 2.40 | 100.00 |
| 1 | BPH | IR22 | 20 | 15 | 2 | 3 | 4.00 | 27.00 | 100.00 |
| 1 | BPH | IR22 | 20 | 15 | 3 | 3 | 9.00 | 26.00 | 100.00 |
| 1 | BPH | IR22 | 20 | 15 | 4 | 3 | 4.67 | 26.33 | 100.00 |
| 1 | BPH | IR22 | 20 | 20 | 1 | 5 | 0.80 | 3.40 | 100.00 |
| 1 | BPH | IR22 | 20 | 20 | 2 | 5 | 1.40 | 9.20 | 100.00 |
| 1 | BPH | IR22 | 20 | 20 | 3 | 3 | 10.33 | 51.00 | 100.00 |
| 1 | BPH | IR22 | 20 | 20 | 4 | 3 | 6.67 | 26.00 | 100.00 |
| 1 | BPH | IR22 | 20 | 25 | 1 | 5 | 2.20 | 7.00 | 100.00 |
| 1 | BPH | IR22 | 20 | 25 | 2 | 5 | 3.60 | 26.20 | 100.00 |
| 1 | BPH | IR22 | 20 | 25 | 3 | 3 | 7.00 | 19.00 | 100.00 |
| 1 | BPH | IR22 | 20 | 25 | 4 | 3 | 12.00 | 42.67 | 100.00 |
| 1 | BPH | IR22 | 20 | 30 | 1 | 5 | 0.80 | 1.20 | 100.00 |
| 1 | BPH | IR22 | 20 | 30 | 2 | 5 | 5.40 | 28.80 | 100.00 |
| 1 | BPH | IR22 | 20 | 30 | 3 | 3 | 9.00 | 34.00 | 100.00 |
| 1 | BPH | IR22 | 20 | 30 | 4 | 3 | 19.33 | 71.33 | 100.00 |
| 1 | BPH | IR22 | 20 | 35 | 1 | 5 | 9.40 | 40.80 | 100.00 |
| 1 | BPH | IR22 | 20 | 35 | 2 | 3 | 9.00 | 30.00 | 100.00 |
| 1 | BPH | IR22 | 20 | 35 | 3 | 3 | 5.00 | 15.67 | 100.00 |
| 1 | BPH | IR22 | 20 | 35 | 4 | 3 | 3.00 | 10.33 | 100.00 |
| 1 | BPH | IR22 | 20 | 40 | 1 | 2 |  | 14.50 | 0.00 |
| 1 | BPH | IR22 | 20 | 40 | 2 | 4 |  | 5.50 | 0.00 |
| 1 | BPH | IR22 | 20 | 40 | 3 | 5 |  | 0.00 | 0.00 |
| 1 | BPH | IR22 | 20 | 40 | 4 | 6 |  | 19.00 | 0.00 |
| 1 | BPH | IR22 | 30 | 15 | 1 | 5 | 0.00 | 0.00 | 100.00 |
| 1 | BPH | IR22 | 30 | 15 | 2 | 3 | 4.67 | 31.33 | 100.00 |
| 1 | BPH | IR22 | 30 | 15 | 3 | 3 | 4.33 | 23.33 | 100.00 |
| 1 | BPH | IR22 | 30 | 15 | 4 | 3 | 2.33 | 22.33 | 100.00 |
| 1 | BPH | IR22 | 30 | 20 | 1 | 5 | 2.00 | 7.60 | 100.00 |
| 1 | BPH | IR22 | 30 | 20 | 2 | 5 | 8.40 | 48.20 | 100.00 |
| 1 | BPH | IR22 | 30 | 20 | 3 | 3 | 5.00 | 44.33 | 100.00 |
| 1 | BPH | IR22 | 30 | 20 | 4 | 3 | 3.67 | 23.67 | 100.00 |
| 1 | BPH | IR22 | 30 | 25 | 1 | 5 | 1.20 | 4.60 | 100.00 |
| 1 | BPH | IR22 | 30 | 25 | 2 | 5 | 13.00 | 66.60 | 100.00 |
| 1 | BPH | IR22 | 30 | 25 | 3 | 3 | 9.00 | 36.00 | 100.00 |
| 1 | BPH | IR22 | 30 | 25 | 4 | 3 | 5.00 | 24.00 | 100.00 |
| 1 | BPH | IR22 | 30 | 30 | 1 | 5 | 0.20 | 0.20 | 100.00 |
| 1 | BPH | IR22 | 30 | 30 | 2 | 5 | 9.00 | 57.40 | 100.00 |
| 1 | BPH | IR22 | 30 | 30 | 3 | 3 | 5.33 | 21.00 | 100.00 |
| 1 | BPH | IR22 | 30 | 30 | 4 | 3 | 8.00 | 46.33 | 100.00 |
| 1 | BPH | IR22 | 30 | 35 | 1 | 5 | 11.80 | 73.80 | 100.00 |
| 1 | BPH | IR22 | 30 | 35 | 2 | 3 | 4.67 | 17.00 | 100.00 |
| 1 | BPH | IR22 | 30 | 35 | 3 | 3 | 3.00 | 24.33 | 100.00 |
| 1 | BPH | IR22 | 30 | 35 | 4 | 3 | 4.00 | 36.33 | 100.00 |
| 1 | BPH | IR22 | 30 | 40 | 1 | 2 |  | 0.00 | 0.00 |
| 1 | BPH | IR22 | 30 | 40 | 2 | 4 |  | 18.50 | 0.00 |
| 1 | BPH | IR22 | 30 | 40 | 3 | 5 |  | 6.00 | 0.00 |
| 1 | BPH | IR22 | 30 | 40 | 4 | 6 |  | 0.00 | 0.00 |
| 1 | BPH | T65 | 20 | 15 | 1 | 5 | 2.20 | 7.40 | 100.00 |
| 1 | BPH | T65 | 20 | 15 | 2 | 3 | 8.00 | 33.67 | 100.00 |
| 1 | BPH | T65 | 20 | 15 | 3 | 3 | 10.33 | 27.00 | 100.00 |
| 1 | BPH | T65 | 20 | 15 | 4 | 3 | 7.00 | 25.00 | 100.00 |
| 1 | BPH | T65 | 20 | 20 | 1 | 5 | 3.80 | 12.80 | 100.00 |
| 1 | BPH | T65 | 20 | 20 | 2 | 5 | 3.40 | 15.20 | 100.00 |
| 1 | BPH | T65 | 20 | 20 | 3 | 3 | 12.00 | 36.67 | 100.00 |
| 1 | BPH | T65 | 20 | 20 | 4 | 3 | 5.67 | 29.33 | 100.00 |
| 1 | BPH | T65 | 20 | 25 | 1 | 5 | 2.00 | 7.40 | 100.00 |
| 1 | BPH | T65 | 20 | 25 | 2 | 5 | 6.20 | 22.20 | 100.00 |
| 1 | BPH | T65 | 20 | 25 | 3 | 3 | 9.00 | 23.33 | 100.00 |
| 1 | BPH | T65 | 20 | 25 | 4 | 3 | 10.67 | 36.33 | 100.00 |
| 1 | BPH | T65 | 20 | 30 | 1 | 5 | 2.40 | 7.40 | 100.00 |
| 1 | BPH | T65 | 20 | 30 | 2 | 5 | 11.80 | 35.20 | 100.00 |
| 1 | BPH | T65 | 20 | 30 | 3 | 3 | 9.67 | 21.33 | 100.00 |
| 1 | BPH | T65 | 20 | 30 | 4 | 3 | 12.00 | 42.33 | 100.00 |
| 1 | BPH | T65 | 20 | 35 | 1 | 5 | 4.80 | 17.80 | 100.00 |
| 1 | BPH | T65 | 20 | 35 | 2 | 3 | 16.33 | 36.67 | 100.00 |
| 1 | BPH | T65 | 20 | 35 | 3 | 3 | 6.33 | 23.33 | 100.00 |
| 1 | BPH | T65 | 20 | 35 | 4 | 3 | 7.00 | 30.00 | 100.00 |
| 1 | BPH | T65 | 20 | 40 | 1 | 2 |  | 8.50 | 0.00 |
| 1 | BPH | T65 | 20 | 40 | 2 | 4 |  | 3.00 | 0.00 |
| 1 | BPH | T65 | 20 | 40 | 3 | 5 |  | 18.00 | 0.00 |
| 1 | BPH | T65 | 20 | 40 | 4 | 6 |  | 9.00 | 0.00 |
| 1 | BPH | T65 | 30 | 15 | 1 | 5 | 0.20 | 0.20 | 100.00 |
| 1 | BPH | T65 | 30 | 15 | 2 | 3 | 3.33 | 21.33 | 100.00 |
| 1 | BPH | T65 | 30 | 15 | 3 | 3 | 2.00 | 6.33 | 100.00 |
| 1 | BPH | T65 | 30 | 15 | 4 | 3 | 2.33 | 19.33 | 100.00 |
| 1 | BPH | T65 | 30 | 20 | 1 | 5 | 1.20 | 3.40 | 100.00 |
| 1 | BPH | T65 | 30 | 20 | 2 | 5 | 8.20 | 48.40 | 100.00 |
| 1 | BPH | T65 | 30 | 20 | 3 | 3 | 2.33 | 16.33 | 100.00 |
| 1 | BPH | T65 | 30 | 20 | 4 | 3 | 5.33 | 29.67 | 100.00 |
| 1 | BPH | T65 | 30 | 25 | 1 | 5 | 1.80 | 5.20 | 100.00 |
| 1 | BPH | T65 | 30 | 25 | 2 | 5 | 11.40 | 60.60 | 100.00 |
| 1 | BPH | T65 | 30 | 25 | 3 | 3 | 9.00 | 27.67 | 100.00 |
| 1 | BPH | T65 | 30 | 25 | 4 | 3 | 3.67 | 25.00 | 100.00 |
| 1 | BPH | T65 | 30 | 30 | 1 | 5 | 1.80 | 8.60 | 100.00 |
| 1 | BPH | T65 | 30 | 30 | 2 | 5 | 11.40 | 62.00 | 100.00 |
| 1 | BPH | T65 | 30 | 30 | 3 | 3 | 8.67 | 28.33 | 100.00 |
| 1 | BPH | T65 | 30 | 30 | 4 | 3 | 8.67 | 32.33 | 100.00 |
| 1 | BPH | T65 | 30 | 35 | 1 | 5 | 8.60 | 53.60 | 100.00 |
| 1 | BPH | T65 | 30 | 35 | 2 | 3 | 26.33 | 62.67 | 100.00 |
| 1 | BPH | T65 | 30 | 35 | 3 | 3 | 5.67 | 37.33 | 100.00 |
| 1 | BPH | T65 | 30 | 35 | 4 | 3 | 2.67 | 25.33 | 100.00 |
| 1 | BPH | T65 | 30 | 40 | 1 | 2 |  | 7.50 | 0.00 |
| 1 | BPH | T65 | 30 | 40 | 2 | 4 |  | 12.50 | 0.00 |
| 1 | BPH | T65 | 30 | 40 | 3 | 5 |  | 0.00 | 0.00 |
| 1 | BPH | T65 | 30 | 40 | 4 | 6 |  | 0.00 | 0.00 |
| 1 | WBPH | IR22 | 20 | 15 | 1 | 5 | 0.20 | 1.40 | 100.00 |
| 1 | WBPH | IR22 | 20 | 15 | 2 | 3 | 2.00 | 13.33 | 100.00 |
| 1 | WBPH | IR22 | 20 | 15 | 3 | 3 | 1.00 | 0.00 | 100.00 |
| 1 | WBPH | IR22 | 20 | 15 | 4 | 3 | 2.33 | 26.67 | 100.00 |
| 1 | WBPH | IR22 | 20 | 20 | 1 | 5 | 0.20 | 1.00 | 100.00 |
| 1 | WBPH | IR22 | 20 | 20 | 2 | 5 | 3.20 | 26.60 | 100.00 |
| 1 | WBPH | IR22 | 20 | 20 | 3 | 3 | 3.33 | 23.33 | 100.00 |
| 1 | WBPH | IR22 | 20 | 20 | 4 | 3 | 3.67 | 24.67 | 100.00 |
| 1 | WBPH | IR22 | 20 | 25 | 1 | 5 | 1.80 | 7.80 | 100.00 |
| 1 | WBPH | IR22 | 20 | 25 | 2 | 5 | 5.60 | 40.60 | 100.00 |
| 1 | WBPH | IR22 | 20 | 25 | 3 | 3 | 1.67 | 5.67 | 100.00 |
| 1 | WBPH | IR22 | 20 | 25 | 4 | 3 | 1.67 | 7.00 | 100.00 |
| 1 | WBPH | IR22 | 20 | 30 | 1 | 5 | 1.60 | 11.40 | 100.00 |
| 1 | WBPH | IR22 | 20 | 30 | 2 | 5 | 4.60 | 38.40 | 100.00 |
| 1 | WBPH | IR22 | 20 | 30 | 3 | 3 | 1.33 | 2.00 | 100.00 |
| 1 | WBPH | IR22 | 20 | 30 | 4 | 3 | 2.00 | 9.33 | 100.00 |
| 1 | WBPH | IR22 | 20 | 35 | 1 | 5 | 7.20 | 43.40 | 100.00 |
| 1 | WBPH | IR22 | 20 | 35 | 2 | 3 | 4.00 | 27.00 | 100.00 |
| 1 | WBPH | IR22 | 20 | 35 | 3 | 3 | 3.33 | 27.33 | 100.00 |
| 1 | WBPH | IR22 | 20 | 35 | 4 | 3 | 2.33 | 24.33 | 100.00 |
| 1 | WBPH | IR22 | 20 | 40 | 1 | 2 |  | 13.00 | 0.00 |
| 1 | WBPH | IR22 | 20 | 40 | 2 | 4 |  | 16.50 | 0.00 |
| 1 | WBPH | IR22 | 20 | 40 | 3 | 5 |  | 3.00 | 1.00 |
| 1 | WBPH | IR22 | 20 | 40 | 4 | 6 |  | 14.00 | 0.00 |
| 1 | WBPH | IR22 | 30 | 15 | 1 | 1 | 0.00 | 0.00 | 100.00 |
| 1 | WBPH | IR22 | 30 | 15 | 2 | 1 | 0.00 | 0.00 | 100.00 |
| 1 | WBPH | IR22 | 30 | 15 | 3 | 1 | 0.00 | 0.00 | 100.00 |
| 1 | WBPH | IR22 | 30 | 15 | 4 | 1 | 0.00 | 0.00 | 100.00 |
| 1 | WBPH | IR22 | 30 | 20 | 1 | 5 | 2.00 | 10.20 | 100.00 |
| 1 | WBPH | IR22 | 30 | 20 | 2 | 5 | 2.20 | 19.40 | 100.00 |
| 1 | WBPH | IR22 | 30 | 20 | 3 | 3 | 4.00 | 20.67 | 100.00 |
| 1 | WBPH | IR22 | 30 | 20 | 4 | 3 | 4.67 | 52.67 | 100.00 |
| 1 | WBPH | IR22 | 30 | 25 | 1 | 5 | 1.60 | 12.80 | 100.00 |
| 1 | WBPH | IR22 | 30 | 25 | 2 | 5 | 1.00 | 2.20 | 100.00 |
| 1 | WBPH | IR22 | 30 | 25 | 3 | 5 | 1.00 | 2.20 | 100.00 |
| 1 | WBPH | IR22 | 30 | 25 | 4 | 5 | 1.00 | 2.20 | 100.00 |
| 1 | WBPH | IR22 | 30 | 30 | 1 | 5 | 0.80 | 3.40 | 100.00 |
| 1 | WBPH | IR22 | 30 | 30 | 2 | 5 | 3.20 | 26.00 | 100.00 |
| 1 | WBPH | IR22 | 30 | 30 | 3 | 3 | 3.67 | 12.67 | 100.00 |
| 1 | WBPH | IR22 | 30 | 30 | 4 | 3 | 1.00 | 1.33 | 100.00 |
| 1 | WBPH | IR22 | 30 | 35 | 1 | 5 | 3.40 | 20.40 | 100.00 |
| 1 | WBPH | IR22 | 30 | 35 | 2 | 3 | 10.67 | 61.00 | 100.00 |
| 1 | WBPH | IR22 | 30 | 35 | 3 | 3 | 9.00 | 49.00 | 100.00 |
| 1 | WBPH | IR22 | 30 | 35 | 4 | 3 | 1.00 | 2.33 | 100.00 |
| 1 | WBPH | IR22 | 30 | 40 | 1 | 2 |  | 0.00 | 0.00 |
| 1 | WBPH | IR22 | 30 | 40 | 2 | 4 |  | 7.00 | 0.50 |
| 1 | WBPH | IR22 | 30 | 40 | 3 | 5 |  | 0.00 | 0.00 |
| 1 | WBPH | IR22 | 30 | 40 | 4 | 6 |  | 23.00 | 0.00 |
| 1 | WBPH | T65 | 20 | 15 | 1 | 5 | 0.20 | 0.80 | 100.00 |
| 1 | WBPH | T65 | 20 | 15 | 2 | 3 | 1.67 | 13.33 | 100.00 |
| 1 | WBPH | T65 | 20 | 15 | 3 | 3 | 1.67 | 5.67 | 100.00 |
| 1 | WBPH | T65 | 20 | 15 | 4 | 3 | 2.00 | 19.00 | 100.00 |
| 1 | WBPH | T65 | 20 | 20 | 1 | 5 | 0.20 | 0.40 | 100.00 |
| 1 | WBPH | T65 | 20 | 20 | 2 | 5 | 4.20 | 32.60 | 100.00 |
| 1 | WBPH | T65 | 20 | 20 | 3 | 3 | 3.67 | 20.33 | 100.00 |
| 1 | WBPH | T65 | 20 | 20 | 4 | 3 | 2.67 | 15.67 | 100.00 |
| 1 | WBPH | T65 | 20 | 25 | 1 | 5 | 0.60 | 3.20 | 100.00 |
| 1 | WBPH | T65 | 20 | 25 | 2 | 5 | 3.00 | 14.80 | 100.00 |
| 1 | WBPH | T65 | 20 | 25 | 3 | 3 | 1.33 | 3.33 | 100.00 |
| 1 | WBPH | T65 | 20 | 25 | 4 | 3 | 5.00 | 32.67 | 100.00 |
| 1 | WBPH | T65 | 20 | 30 | 1 | 5 | 0.60 | 2.00 | 100.00 |
| 1 | WBPH | T65 | 20 | 30 | 2 | 5 | 3.00 | 20.40 | 100.00 |
| 1 | WBPH | T65 | 20 | 30 | 3 | 3 | 1.00 | 0.00 | 100.00 |
| 1 | WBPH | T65 | 20 | 30 | 4 | 3 | 3.67 | 21.00 | 100.00 |
| 1 | WBPH | T65 | 20 | 35 | 1 | 5 | 9.00 | 54.20 | 100.00 |
| 1 | WBPH | T65 | 20 | 35 | 2 | 3 | 4.33 | 12.00 | 100.00 |
| 1 | WBPH | T65 | 20 | 35 | 3 | 3 | 5.33 | 28.00 | 100.00 |
| 1 | WBPH | T65 | 20 | 35 | 4 | 3 | 8.00 | 39.00 | 100.00 |
| 1 | WBPH | T65 | 20 | 40 | 1 | 2 |  | 12.50 | 0.50 |
| 1 | WBPH | T65 | 20 | 40 | 2 | 4 |  | 0.00 | 0.50 |
| 1 | WBPH | T65 | 20 | 40 | 3 | 5 |  | 0.00 | 0.00 |
| 1 | WBPH | T65 | 20 | 40 | 4 | 6 |  | 0.00 | 0.00 |
| 1 | WBPH | T65 | 30 | 15 | 1 | 1 | 0.50 | 5.00 | 100.00 |
| 1 | WBPH | T65 | 30 | 15 | 2 | 1 | 0.00 | 0.00 | 100.00 |
| 1 | WBPH | T65 | 30 | 15 | 3 | 1 | 0.00 | 0.00 | 100.00 |
| 1 | WBPH | T65 | 30 | 15 | 4 | 1 | 0.00 | 0.00 | 100.00 |
| 1 | WBPH | T65 | 30 | 20 | 1 | 5 | 0.80 | 3.40 | 100.00 |
| 1 | WBPH | T65 | 30 | 20 | 2 | 5 | 1.40 | 4.80 | 100.00 |
| 1 | WBPH | T65 | 30 | 20 | 3 | 3 | 11.00 | 65.33 | 100.00 |
| 1 | WBPH | T65 | 30 | 20 | 4 | 3 | 1.67 | 16.67 | 100.00 |
| 1 | WBPH | T65 | 30 | 25 | 1 | 5 | 0.80 | 3.20 | 100.00 |
| 1 | WBPH | T65 | 30 | 25 | 2 | 5 | 1.80 | 8.60 | 100.00 |
| 1 | WBPH | T65 | 30 | 25 | 3 | 5 | 1.80 | 8.60 | 100.00 |
| 1 | WBPH | T65 | 30 | 25 | 4 | 5 | 1.80 | 8.60 | 100.00 |
| 1 | WBPH | T65 | 30 | 30 | 1 | 5 | 0.40 | 1.20 | 100.00 |
| 1 | WBPH | T65 | 30 | 30 | 2 | 5 | 2.80 | 20.40 | 100.00 |
| 1 | WBPH | T65 | 30 | 30 | 3 | 3 | 1.67 | 12.33 | 100.00 |
| 1 | WBPH | T65 | 30 | 30 | 4 | 3 | 2.33 | 10.00 | 100.00 |
| 1 | WBPH | T65 | 30 | 35 | 1 | 5 | 2.80 | 11.60 | 100.00 |
| 1 | WBPH | T65 | 30 | 35 | 2 | 3 | 7.67 | 28.33 | 100.00 |
| 1 | WBPH | T65 | 30 | 35 | 3 | 3 | 7.00 | 31.00 | 100.00 |
| 1 | WBPH | T65 | 30 | 35 | 4 | 3 | 1.00 | 9.00 | 100.00 |
| 1 | WBPH | T65 | 30 | 40 | 1 | 2 |  | 19.50 | 50.00 |
| 1 | WBPH | T65 | 30 | 40 | 2 | 4 |  | 10.50 | 100.00 |
| 1 | WBPH | T65 | 30 | 40 | 3 | 5 |  | 18.00 | 100.00 |
| 1 | WBPH | T65 | 30 | 40 | 4 | 6 |  | 0.00 | 100.00 |
| 2 | BPH | IR22 | 20 | 15 | 1 | 5 | 0.40 | 0.80 | 100.00 |
| 2 | BPH | IR22 | 20 | 15 | 2 | 3 | 2.33 | 10.67 | 100.00 |
| 2 | BPH | IR22 | 20 | 15 | 3 | 3 | 2.67 | 8.00 | 100.00 |
| 2 | BPH | IR22 | 20 | 15 | 4 | 3 | 1.00 | 9.33 | 100.00 |
| 2 | BPH | IR22 | 20 | 20 | 1 | 5 | 1.00 | 1.60 | 100.00 |
| 2 | BPH | IR22 | 20 | 20 | 2 | 5 | 4.20 | 15.80 | 100.00 |
| 2 | BPH | IR22 | 20 | 20 | 3 | 3 | 5.33 | 24.00 | 100.00 |
| 2 | BPH | IR22 | 20 | 20 | 4 | 3 | 1.00 | 0.00 | 100.00 |
| 2 | BPH | IR22 | 20 | 25 | 1 | 5 | 5.60 | 17.00 | 100.00 |
| 2 | BPH | IR22 | 20 | 25 | 2 | 5 | 10.25 | 47.25 | 80.00 |
| 2 | BPH | IR22 | 20 | 25 | 3 | 3 | 1.67 | 4.67 | 100.00 |
| 2 | BPH | IR22 | 20 | 25 | 4 | 3 | 2.33 | 13.00 | 100.00 |
| 2 | BPH | IR22 | 20 | 30 | 1 | 5 | 6.20 | 22.20 | 100.00 |
| 2 | BPH | IR22 | 20 | 30 | 2 | 5 | 16.00 | 59.75 | 80.00 |
| 2 | BPH | IR22 | 20 | 30 | 3 | 3 | 2.67 | 6.67 | 100.00 |
| 2 | BPH | IR22 | 20 | 30 | 4 | 3 | 11.00 | 48.33 | 100.00 |
| 2 | BPH | IR22 | 20 | 35 | 1 | 5 | 12.00 | 44.75 | 80.00 |
| 2 | BPH | IR22 | 20 | 35 | 2 | 3 | 1.00 | 3.00 | 33.33 |
| 2 | BPH | IR22 | 20 | 35 | 3 | 3 | 2.67 | 10.67 | 100.00 |
| 2 | BPH | IR22 | 20 | 35 | 4 | 3 | 4.00 | 28.50 | 66.67 |
| 2 | BPH | IR22 | 20 | 40 | 1 | 2 |  | 0.00 | 0.00 |
| 2 | BPH | IR22 | 20 | 40 | 2 | 4 |  | 0.00 | 0.00 |
| 2 | BPH | IR22 | 20 | 40 | 3 | 5 |  | 0.00 | 0.00 |
| 2 | BPH | IR22 | 20 | 40 | 4 | 6 |  | 0.00 | 0.00 |
| 2 | BPH | IR22 | 30 | 15 | 1 | 5 | 2.50 | 6.50 | 40.00 |
| 2 | BPH | IR22 | 30 | 15 | 2 | 3 | 3.00 | 17.33 | 100.00 |
| 2 | BPH | IR22 | 30 | 15 | 3 | 3 | 1.67 | 7.00 | 100.00 |
| 2 | BPH | IR22 | 30 | 15 | 4 | 3 | 2.00 | 8.00 | 100.00 |
| 2 | BPH | IR22 | 30 | 20 | 1 | 5 | 1.00 | 4.60 | 100.00 |
| 2 | BPH | IR22 | 30 | 20 | 2 | 5 | 4.60 | 18.40 | 100.00 |
| 2 | BPH | IR22 | 30 | 20 | 3 | 3 | 4.67 | 17.33 | 100.00 |
| 2 | BPH | IR22 | 30 | 20 | 4 | 3 | 1.00 | 2.67 | 100.00 |
| 2 | BPH | IR22 | 30 | 25 | 1 | 5 | 5.20 | 14.00 | 100.00 |
| 2 | BPH | IR22 | 30 | 25 | 2 | 5 | 2.60 | 14.60 | 100.00 |
| 2 | BPH | IR22 | 30 | 25 | 3 | 3 | 6.00 | 26.00 | 66.67 |
| 2 | BPH | IR22 | 30 | 25 | 4 | 3 | 1.00 | 1.67 | 100.00 |
| 2 | BPH | IR22 | 30 | 30 | 1 | 5 | 5.20 | 22.20 | 100.00 |
| 2 | BPH | IR22 | 30 | 30 | 2 | 5 | 5.75 | 25.75 | 80.00 |
| 2 | BPH | IR22 | 30 | 30 | 3 | 3 | 2.33 | 7.00 | 100.00 |
| 2 | BPH | IR22 | 30 | 30 | 4 | 3 | 2.33 | 10.00 | 100.00 |
| 2 | BPH | IR22 | 30 | 35 | 1 | 5 | 3.40 | 18.00 | 100.00 |
| 2 | BPH | IR22 | 30 | 35 | 2 | 3 | 1.00 | 0.00 | 33.33 |
| 2 | BPH | IR22 | 30 | 35 | 3 | 3 | 2.33 | 16.00 | 100.00 |
| 2 | BPH | IR22 | 30 | 35 | 4 | 3 | 2.67 | 30.33 | 100.00 |
| 2 | BPH | IR22 | 30 | 40 | 1 | 2 |  | 0.00 | 0.00 |
| 2 | BPH | IR22 | 30 | 40 | 2 | 4 |  | 0.00 | 0.00 |
| 2 | BPH | IR22 | 30 | 40 | 3 | 5 |  | 0.00 | 0.00 |
| 2 | BPH | IR22 | 30 | 40 | 4 | 6 |  | 0.00 | 0.00 |
| 2 | BPH | T65 | 20 | 15 | 1 | 5 | 0.60 | 1.40 | 100.00 |
| 2 | BPH | T65 | 20 | 15 | 2 | 3 | 3.00 | 14.33 | 100.00 |
| 2 | BPH | T65 | 20 | 15 | 3 | 3 | 1.33 | 2.00 | 100.00 |
| 2 | BPH | T65 | 20 | 15 | 4 | 3 | 1.67 | 3.33 | 100.00 |
| 2 | BPH | T65 | 20 | 20 | 1 | 5 | 5.20 | 12.20 | 100.00 |
| 2 | BPH | T65 | 20 | 20 | 2 | 5 | 3.50 | 10.00 | 80.00 |
| 2 | BPH | T65 | 20 | 20 | 3 | 3 | 6.67 | 16.00 | 100.00 |
| 2 | BPH | T65 | 20 | 20 | 4 | 3 | 1.00 | 25.00 | 33.33 |
| 2 | BPH | T65 | 20 | 25 | 1 | 5 | 8.00 | 24.40 | 100.00 |
| 2 | BPH | T65 | 20 | 25 | 2 | 5 | 5.75 | 28.00 | 80.00 |
| 2 | BPH | T65 | 20 | 25 | 3 | 3 | 1.50 | 3.00 | 66.67 |
| 2 | BPH | T65 | 20 | 25 | 4 | 3 | 6.67 | 23.33 | 100.00 |
| 2 | BPH | T65 | 20 | 30 | 1 | 5 | 14.40 | 39.80 | 100.00 |
| 2 | BPH | T65 | 20 | 30 | 2 | 5 | 12.50 | 43.00 | 80.00 |
| 2 | BPH | T65 | 20 | 30 | 3 | 3 | 6.50 | 13.00 | 66.67 |
| 2 | BPH | T65 | 20 | 30 | 4 | 3 | 12.67 | 40.33 | 100.00 |
| 2 | BPH | T65 | 20 | 35 | 1 | 5 | 6.33 | 31.33 | 60.00 |
| 2 | BPH | T65 | 20 | 35 | 2 | 3 | 13.00 | 39.50 | 66.67 |
| 2 | BPH | T65 | 20 | 35 | 3 | 3 | 4.33 | 15.00 | 100.00 |
| 2 | BPH | T65 | 20 | 35 | 4 | 3 | 4.33 | 19.33 | 100.00 |
| 2 | BPH | T65 | 20 | 40 | 1 | 2 |  | 0.00 | 0.00 |
| 2 | BPH | T65 | 20 | 40 | 2 | 4 |  | 0.00 | 0.00 |
| 2 | BPH | T65 | 20 | 40 | 3 | 5 |  | 0.00 | 0.00 |
| 2 | BPH | T65 | 20 | 40 | 4 | 6 |  | 0.00 | 0.00 |
| 2 | BPH | T65 | 30 | 15 | 1 | 5 | 1.00 | 2.80 | 100.00 |
| 2 | BPH | T65 | 30 | 15 | 2 | 3 | 1.00 | 7.00 | 66.67 |
| 2 | BPH | T65 | 30 | 15 | 3 | 3 | 2.50 | 6.50 | 66.67 |
| 2 | BPH | T65 | 30 | 15 | 4 | 3 | 1.67 | 5.00 | 100.00 |
| 2 | BPH | T65 | 30 | 20 | 1 | 5 | 1.60 | 6.00 | 100.00 |
| 2 | BPH | T65 | 30 | 20 | 2 | 5 | 4.00 | 24.00 | 100.00 |
| 2 | BPH | T65 | 30 | 20 | 3 | 3 | 2.00 | 22.00 | 33.33 |
| 2 | BPH | T65 | 30 | 20 | 4 | 3 | 1.00 | 0.00 | 100.00 |
| 2 | BPH | T65 | 30 | 25 | 1 | 5 | 3.80 | 13.80 | 100.00 |
| 2 | BPH | T65 | 30 | 25 | 2 | 5 | 5.00 | 30.80 | 100.00 |
| 2 | BPH | T65 | 30 | 25 | 3 | 3 | 3.67 | 16.67 | 100.00 |
| 2 | BPH | T65 | 30 | 25 | 4 | 3 | 4.50 | 23.00 | 66.67 |
| 2 | BPH | T65 | 30 | 30 | 1 | 5 | 5.80 | 20.00 | 100.00 |
| 2 | BPH | T65 | 30 | 30 | 2 | 5 | 4.80 | 31.20 | 100.00 |
| 2 | BPH | T65 | 30 | 30 | 3 | 3 | 8.00 | 18.00 | 100.00 |
| 2 | BPH | T65 | 30 | 30 | 4 | 3 | 4.33 | 17.33 | 100.00 |
| 2 | BPH | T65 | 30 | 35 | 1 | 5 | 3.80 | 16.60 | 100.00 |
| 2 | BPH | T65 | 30 | 35 | 2 | 3 | 12.33 | 48.33 | 100.00 |
| 2 | BPH | T65 | 30 | 35 | 3 | 3 | 2.00 | 7.67 | 100.00 |
| 2 | BPH | T65 | 30 | 35 | 4 | 3 | 2.50 | 29.50 | 66.67 |
| 2 | BPH | T65 | 30 | 40 | 1 | 2 |  | 0.00 | 0.00 |
| 2 | BPH | T65 | 30 | 40 | 2 | 4 |  | 0.00 | 0.00 |
| 2 | BPH | T65 | 30 | 40 | 3 | 5 |  | 0.00 | 0.00 |
| 2 | BPH | T65 | 30 | 40 | 4 | 6 |  | 0.00 | 0.00 |
| 2 | WBPH | IR22 | 20 | 15 | 1 | 5 | 1.00 | 5.50 | 80.00 |
| 2 | WBPH | IR22 | 20 | 15 | 2 | 3 | 1.67 | 14.00 | 100.00 |
| 2 | WBPH | IR22 | 20 | 15 | 3 | 3 | 1.00 | 4.00 | 100.00 |
| 2 | WBPH | IR22 | 20 | 15 | 4 | 3 | 1.00 | 3.67 | 100.00 |
| 2 | WBPH | IR22 | 20 | 20 | 1 | 5 | 0.20 | 1.00 | 100.00 |
| 2 | WBPH | IR22 | 20 | 20 | 2 | 5 | 2.00 | 9.80 | 100.00 |
| 2 | WBPH | IR22 | 20 | 20 | 3 | 3 | 1.67 | 10.33 | 100.00 |
| 2 | WBPH | IR22 | 20 | 20 | 4 | 3 | 2.33 | 16.33 | 100.00 |
| 2 | WBPH | IR22 | 20 | 25 | 1 | 5 | 0.80 | 4.40 | 100.00 |
| 2 | WBPH | IR22 | 20 | 25 | 2 | 5 | 1.40 | 11.00 | 100.00 |
| 2 | WBPH | IR22 | 20 | 25 | 3 | 3 | 1.00 | 2.00 | 33.33 |
| 2 | WBPH | IR22 | 20 | 25 | 4 | 3 | 2.00 | 10.00 | 100.00 |
| 2 | WBPH | IR22 | 20 | 30 | 1 | 5 | 0.40 | 1.60 | 100.00 |
| 2 | WBPH | IR22 | 20 | 30 | 2 | 5 | 1.50 | 7.75 | 80.00 |
| 2 | WBPH | IR22 | 20 | 30 | 3 | 3 | 1.00 | 0.00 | 66.67 |
| 2 | WBPH | IR22 | 20 | 30 | 4 | 3 | 3.50 | 24.50 | 66.67 |
| 2 | WBPH | IR22 | 20 | 35 | 1 | 5 | 0.25 | 0.75 | 80.00 |
| 2 | WBPH | IR22 | 20 | 35 | 2 | 3 | 1.33 | 6.67 | 100.00 |
| 2 | WBPH | IR22 | 20 | 35 | 3 | 3 | 1.00 | 0.00 | 100.00 |
| 2 | WBPH | IR22 | 20 | 35 | 4 | 3 | 1.33 | 3.33 | 100.00 |
| 2 | WBPH | IR22 | 20 | 40 | 1 | 2 |  | 0.00 | 0.00 |
| 2 | WBPH | IR22 | 20 | 40 | 2 | 4 |  | 0.00 | 0.00 |
| 2 | WBPH | IR22 | 20 | 40 | 3 | 5 |  | 0.00 | 0.00 |
| 2 | WBPH | IR22 | 20 | 40 | 4 | 6 |  | 0.00 | 0.00 |
| 2 | WBPH | IR22 | 30 | 15 | 1 | 1 | 1.50 | 9.00 | 100.00 |
| 2 | WBPH | IR22 | 30 | 15 | 2 | 1 | 0.00 | 0.00 | 100.00 |
| 2 | WBPH | IR22 | 30 | 15 | 3 | 1 | 0.00 | 0.00 | 80.00 |
| 2 | WBPH | IR22 | 30 | 15 | 4 | 1 | 0.00 | 0.00 | 100.00 |
| 2 | WBPH | IR22 | 30 | 20 | 1 | 5 | 0.40 | 2.40 | 100.00 |
| 2 | WBPH | IR22 | 30 | 20 | 2 | 5 | 1.00 | 4.25 | 80.00 |
| 2 | WBPH | IR22 | 30 | 20 | 3 | 3 | 2.00 | 10.33 | 100.00 |
| 2 | WBPH | IR22 | 30 | 20 | 4 | 3 | 1.33 | 17.33 | 100.00 |
| 2 | WBPH | IR22 | 30 | 25 | 1 | 5 | 1.25 | 4.50 | 80.00 |
| 2 | WBPH | IR22 | 30 | 25 | 2 | 5 | 1.00 | 0.00 | 40.00 |
| 2 | WBPH | IR22 | 30 | 25 | 3 | 5 | 1.00 | 0.00 | 40.00 |
| 2 | WBPH | IR22 | 30 | 25 | 4 | 5 | 1.00 | 0.00 | 40.00 |
| 2 | WBPH | IR22 | 30 | 30 | 1 | 5 | 0.60 | 2.00 | 100.00 |
| 2 | WBPH | IR22 | 30 | 30 | 2 | 5 | 1.33 | 1.67 | 60.00 |
| 2 | WBPH | IR22 | 30 | 30 | 3 | 3 | 1.00 | 0.00 | 33.33 |
| 2 | WBPH | IR22 | 30 | 30 | 4 | 3 | 1.00 | 0.00 | 33.33 |
| 2 | WBPH | IR22 | 30 | 35 | 1 | 5 | 1.00 | 0.75 | 80.00 |
| 2 | WBPH | IR22 | 30 | 35 | 2 | 3 | 1.33 | 3.67 | 100.00 |
| 2 | WBPH | IR22 | 30 | 35 | 3 | 3 | 1.33 | 3.67 | 100.00 |
| 2 | WBPH | IR22 | 30 | 35 | 4 | 3 | 0.00 | 0.00 | 0.00 |
| 2 | WBPH | IR22 | 30 | 40 | 1 | 2 |  | 0.00 | 0.00 |
| 2 | WBPH | IR22 | 30 | 40 | 2 | 4 |  | 0.00 | 0.00 |
| 2 | WBPH | IR22 | 30 | 40 | 3 | 5 |  | 0.00 | 0.00 |
| 2 | WBPH | IR22 | 30 | 40 | 4 | 6 |  | 0.00 | 0.00 |
| 2 | WBPH | T65 | 20 | 15 | 1 | 5 | 0.60 | 2.40 | 100.00 |
| 2 | WBPH | T65 | 20 | 15 | 2 | 3 | 1.67 | 9.33 | 100.00 |
| 2 | WBPH | T65 | 20 | 15 | 3 | 3 | 1.33 | 8.33 | 100.00 |
| 2 | WBPH | T65 | 20 | 15 | 4 | 3 | 1.00 | 7.00 | 100.00 |
| 2 | WBPH | T65 | 20 | 20 | 1 | 5 | 0.00 | 0.00 | 100.00 |
| 2 | WBPH | T65 | 20 | 20 | 2 | 5 | 0.80 | 5.00 | 100.00 |
| 2 | WBPH | T65 | 20 | 20 | 3 | 3 | 1.33 | 16.67 | 100.00 |
| 2 | WBPH | T65 | 20 | 20 | 4 | 3 | 1.00 | 0.00 | 100.00 |
| 2 | WBPH | T65 | 20 | 25 | 1 | 5 | 1.40 | 6.00 | 100.00 |
| 2 | WBPH | T65 | 20 | 25 | 2 | 5 | 1.00 | 4.50 | 80.00 |
| 2 | WBPH | T65 | 20 | 25 | 3 | 3 | 1.33 | 4.33 | 100.00 |
| 2 | WBPH | T65 | 20 | 25 | 4 | 3 | 5.33 | 34.33 | 100.00 |
| 2 | WBPH | T65 | 20 | 30 | 1 | 5 | 2.60 | 10.80 | 100.00 |
| 2 | WBPH | T65 | 20 | 30 | 2 | 5 | 8.33 | 25.67 | 60.00 |
| 2 | WBPH | T65 | 20 | 30 | 3 | 3 | 3.00 | 14.33 | 100.00 |
| 2 | WBPH | T65 | 20 | 30 | 4 | 3 | 4.00 | 26.00 | 66.67 |
| 2 | WBPH | T65 | 20 | 35 | 1 | 5 | 3.20 | 15.80 | 100.00 |
| 2 | WBPH | T65 | 20 | 35 | 2 | 3 | 3.00 | 10.50 | 66.67 |
| 2 | WBPH | T65 | 20 | 35 | 3 | 3 | 3.33 | 19.00 | 100.00 |
| 2 | WBPH | T65 | 20 | 35 | 4 | 3 | 2.67 | 22.00 | 100.00 |
| 2 | WBPH | T65 | 20 | 40 | 1 | 2 |  | 0.00 | 0.00 |
| 2 | WBPH | T65 | 20 | 40 | 2 | 4 |  | 0.00 | 0.00 |
| 2 | WBPH | T65 | 20 | 40 | 3 | 5 |  | 0.00 | 0.00 |
| 2 | WBPH | T65 | 20 | 40 | 4 | 6 |  | 0.00 | 0.00 |
| 2 | WBPH | T65 | 30 | 15 | 1 | 1 | 0.00 | 0.00 | 100.00 |
| 2 | WBPH | T65 | 30 | 15 | 2 | 1 | 0.00 | 0.00 | 100.00 |
| 2 | WBPH | T65 | 30 | 15 | 3 | 1 | 0.00 | 0.00 | 100.00 |
| 2 | WBPH | T65 | 30 | 15 | 4 | 1 | 0.00 | 0.00 | 100.00 |
| 2 | WBPH | T65 | 30 | 20 | 1 | 5 | 0.40 | 2.40 | 100.00 |
| 2 | WBPH | T65 | 30 | 20 | 2 | 5 | 1.00 | 0.00 | 40.00 |
| 2 | WBPH | T65 | 30 | 20 | 3 | 3 | 1.67 | 8.67 | 100.00 |
| 2 | WBPH | T65 | 30 | 20 | 4 | 3 | 1.33 | 11.00 | 100.00 |
| 2 | WBPH | T65 | 30 | 25 | 1 | 5 | 0.80 | 2.60 | 100.00 |
| 2 | WBPH | T65 | 30 | 25 | 2 | 5 | 1.00 | 0.00 | 80.00 |
| 2 | WBPH | T65 | 30 | 25 | 3 | 5 | 1.00 | 0.00 | 80.00 |
| 2 | WBPH | T65 | 30 | 25 | 4 | 5 | 1.00 | 0.00 | 80.00 |
| 2 | WBPH | T65 | 30 | 30 | 1 | 5 | 1.60 | 5.60 | 100.00 |
| 2 | WBPH | T65 | 30 | 30 | 2 | 5 | 1.67 | 6.33 | 60.00 |
| 2 | WBPH | T65 | 30 | 30 | 3 | 3 | 1.00 | 5.00 | 66.67 |
| 2 | WBPH | T65 | 30 | 30 | 4 | 3 | 1.00 | 4.50 | 66.67 |
| 2 | WBPH | T65 | 30 | 35 | 1 | 5 | 1.00 | 1.33 | 60.00 |
| 2 | WBPH | T65 | 30 | 35 | 2 | 3 | 2.00 | 5.00 | 33.33 |
| 2 | WBPH | T65 | 30 | 35 | 3 | 3 | 1.50 | 3.50 | 66.67 |
| 2 | WBPH | T65 | 30 | 35 | 4 | 3 | 1.00 | 0.00 | 66.67 |
| 2 | WBPH | T65 | 30 | 40 | 1 | 2 |  | 0.00 | 0.00 |
| 2 | WBPH | T65 | 30 | 40 | 2 | 4 |  | 0.00 | 0.00 |
| 2 | WBPH | T65 | 30 | 40 | 3 | 5 |  | 0.00 | 0.00 |
| 2 | WBPH | T65 | 30 | 40 | 4 | 6 |  | 0.00 | 0.00 |
| 3 | BPH | IR22 | 20 | 15 | 1 | 5 | 0.20 | 0.20 | 100.00 |
| 3 | BPH | IR22 | 20 | 15 | 2 | 3 | 1.67 | 6.00 | 100.00 |
| 3 | BPH | IR22 | 20 | 15 | 3 | 3 | 4.67 | 15.00 | 100.00 |
| 3 | BPH | IR22 | 20 | 15 | 4 | 3 | 2.33 | 11.33 | 100.00 |
| 3 | BPH | IR22 | 20 | 20 | 1 | 5 | 3.00 | 10.20 | 100.00 |
| 3 | BPH | IR22 | 20 | 20 | 2 | 5 | 4.60 | 25.20 | 100.00 |
| 3 | BPH | IR22 | 20 | 20 | 3 | 3 | 4.33 | 16.67 | 100.00 |
| 3 | BPH | IR22 | 20 | 20 | 4 | 3 | 1.50 | 9.00 | 66.67 |
| 3 | BPH | IR22 | 20 | 25 | 1 | 5 | 6.00 | 15.00 | 100.00 |
| 3 | BPH | IR22 | 20 | 25 | 2 | 5 | 4.00 | 16.75 | 80.00 |
| 3 | BPH | IR22 | 20 | 25 | 3 | 3 | 2.00 | 4.67 | 100.00 |
| 3 | BPH | IR22 | 20 | 25 | 4 | 3 | 4.00 | 23.33 | 100.00 |
| 3 | BPH | IR22 | 20 | 30 | 1 | 5 | 7.60 | 24.00 | 100.00 |
| 3 | BPH | IR22 | 20 | 30 | 2 | 5 | 7.75 | 32.25 | 80.00 |
| 3 | BPH | IR22 | 20 | 30 | 3 | 3 | 1.50 | 4.50 | 66.67 |
| 3 | BPH | IR22 | 20 | 30 | 4 | 3 | 11.00 | 44.67 | 100.00 |
| 3 | BPH | IR22 | 20 | 35 | 1 | 5 | 6.50 | 29.50 | 80.00 |
| 3 | BPH | IR22 | 20 | 35 | 2 | 3 | 1.00 | 0.00 | 33.33 |
| 3 | BPH | IR22 | 20 | 35 | 3 | 3 | 1.33 | 3.00 | 100.00 |
| 3 | BPH | IR22 | 20 | 35 | 4 | 3 | 1.00 | 0.00 | 66.67 |
| 3 | BPH | IR22 | 30 | 15 | 1 | 5 | 0.00 | 0.00 | 40.00 |
| 3 | BPH | IR22 | 30 | 15 | 2 | 3 | 2.00 | 5.33 | 100.00 |
| 3 | BPH | IR22 | 30 | 15 | 3 | 3 | 3.00 | 11.00 | 100.00 |
| 3 | BPH | IR22 | 30 | 15 | 4 | 3 | 2.33 | 12.33 | 100.00 |
| 3 | BPH | IR22 | 30 | 20 | 1 | 5 | 1.40 | 4.20 | 100.00 |
| 3 | BPH | IR22 | 30 | 20 | 2 | 5 | 2.20 | 6.80 | 100.00 |
| 3 | BPH | IR22 | 30 | 20 | 3 | 3 | 1.00 | 5.00 | 100.00 |
| 3 | BPH | IR22 | 30 | 20 | 4 | 3 | 1.67 | 5.00 | 100.00 |
| 3 | BPH | IR22 | 30 | 25 | 1 | 5 | 4.20 | 14.00 | 100.00 |
| 3 | BPH | IR22 | 30 | 25 | 2 | 5 | 4.40 | 23.20 | 100.00 |
| 3 | BPH | IR22 | 30 | 25 | 3 | 3 | 6.00 | 32.00 | 66.67 |
| 3 | BPH | IR22 | 30 | 25 | 4 | 3 | 2.00 | 7.00 | 100.00 |
| 3 | BPH | IR22 | 30 | 30 | 1 | 5 | 8.60 | 29.20 | 100.00 |
| 3 | BPH | IR22 | 30 | 30 | 2 | 5 | 3.25 | 17.50 | 80.00 |
| 3 | BPH | IR22 | 30 | 30 | 3 | 3 | 3.00 | 13.00 | 33.33 |
| 3 | BPH | IR22 | 30 | 30 | 4 | 3 | 0.67 | 3.00 | 66.67 |
| 3 | BPH | IR22 | 30 | 35 | 1 | 5 | 2.60 | 17.40 | 100.00 |
| 3 | BPH | IR22 | 30 | 35 | 2 | 3 | 1.00 | 0.00 | 33.33 |
| 3 | BPH | IR22 | 30 | 35 | 3 | 3 | 1.00 | 0.00 | 100.00 |
| 3 | BPH | IR22 | 30 | 35 | 4 | 3 | 1.00 | 2.67 | 100.00 |
| 3 | BPH | T65 | 20 | 15 | 1 | 5 | 0.00 | 0.00 | 100.00 |
| 3 | BPH | T65 | 20 | 15 | 2 | 3 | 2.00 | 7.67 | 100.00 |
| 3 | BPH | T65 | 20 | 15 | 3 | 3 | 6.00 | 10.50 | 66.67 |
| 3 | BPH | T65 | 20 | 15 | 4 | 3 | 1.00 | 0.00 | 100.00 |
| 3 | BPH | T65 | 20 | 20 | 1 | 5 | 7.60 | 16.60 | 100.00 |
| 3 | BPH | T65 | 20 | 20 | 2 | 5 | 8.33 | 35.67 | 60.00 |
| 3 | BPH | T65 | 20 | 20 | 3 | 3 | 2.00 | 7.33 | 100.00 |
| 3 | BPH | T65 | 20 | 20 | 4 | 3 | 5.00 | 27.00 | 33.33 |
| 3 | BPH | T65 | 20 | 25 | 1 | 5 | 8.80 | 23.40 | 100.00 |
| 3 | BPH | T65 | 20 | 25 | 2 | 5 | 9.00 | 33.00 | 80.00 |
| 3 | BPH | T65 | 20 | 25 | 3 | 3 | 2.00 | 3.00 | 66.67 |
| 3 | BPH | T65 | 20 | 25 | 4 | 3 | 7.67 | 16.33 | 100.00 |
| 3 | BPH | T65 | 20 | 30 | 1 | 5 | 5.20 | 15.40 | 100.00 |
| 3 | BPH | T65 | 20 | 30 | 2 | 5 | 18.33 | 74.33 | 60.00 |
| 3 | BPH | T65 | 20 | 30 | 3 | 3 | 13.00 | 37.00 | 33.33 |
| 3 | BPH | T65 | 20 | 30 | 4 | 3 | 8.67 | 31.00 | 100.00 |
| 3 | BPH | T65 | 20 | 35 | 1 | 5 | 4.67 | 17.33 | 60.00 |
| 3 | BPH | T65 | 20 | 35 | 2 | 3 | 14.00 | 44.00 | 66.67 |
| 3 | BPH | T65 | 20 | 35 | 3 | 3 | 2.00 | 7.00 | 33.33 |
| 3 | BPH | T65 | 20 | 35 | 4 | 3 | 1.00 | 4.00 | 100.00 |
| 3 | BPH | T65 | 30 | 15 | 1 | 5 | 1.00 | 2.60 | 100.00 |
| 3 | BPH | T65 | 30 | 15 | 2 | 3 | 1.00 | 0.00 | 66.67 |
| 3 | BPH | T65 | 30 | 15 | 3 | 3 | 8.50 | 18.00 | 66.67 |
| 3 | BPH | T65 | 30 | 15 | 4 | 3 | 3.00 | 13.67 | 100.00 |
| 3 | BPH | T65 | 30 | 20 | 1 | 5 | 3.60 | 10.80 | 100.00 |
| 3 | BPH | T65 | 30 | 20 | 2 | 5 | 3.40 | 21.60 | 100.00 |
| 3 | BPH | T65 | 30 | 20 | 3 | 3 | 1.00 | 8.00 | 33.33 |
| 3 | BPH | T65 | 30 | 20 | 4 | 3 | 7.50 | 22.00 | 66.67 |
| 3 | BPH | T65 | 30 | 25 | 1 | 5 | 5.60 | 17.80 | 100.00 |
| 3 | BPH | T65 | 30 | 25 | 2 | 5 | 3.20 | 28.00 | 100.00 |
| 3 | BPH | T65 | 30 | 25 | 3 | 3 | 4.67 | 15.00 | 100.00 |
| 3 | BPH | T65 | 30 | 25 | 4 | 3 | 1.00 | 3.00 | 66.67 |
| 3 | BPH | T65 | 30 | 30 | 1 | 5 | 8.00 | 31.75 | 80.00 |
| 3 | BPH | T65 | 30 | 30 | 2 | 5 | 5.80 | 37.40 | 100.00 |
| 3 | BPH | T65 | 30 | 30 | 3 | 3 | 9.50 | 29.00 | 66.67 |
| 3 | BPH | T65 | 30 | 30 | 4 | 3 | 6.33 | 22.00 | 100.00 |
| 3 | BPH | T65 | 30 | 35 | 1 | 5 | 3.40 | 15.60 | 100.00 |
| 3 | BPH | T65 | 30 | 35 | 2 | 3 | 3.33 | 7.67 | 100.00 |
| 3 | BPH | T65 | 30 | 35 | 3 | 3 | 1.00 | 7.00 | 66.67 |
| 3 | BPH | T65 | 30 | 35 | 4 | 3 | 3.50 | 14.00 | 66.67 |
| 3 | WBPH | IR22 | 20 | 15 | 1 | 5 | 0.25 | 1.00 | 80.00 |
| 3 | WBPH | IR22 | 20 | 15 | 2 | 3 | 1.00 | 3.67 | 100.00 |
| 3 | WBPH | IR22 | 20 | 15 | 3 | 3 | 1.00 | 5.67 | 100.00 |
| 3 | WBPH | IR22 | 20 | 15 | 4 | 3 | 1.33 | 13.00 | 100.00 |
| 3 | WBPH | IR22 | 20 | 20 | 1 | 5 | 0.67 | 1.33 | 60.00 |
| 3 | WBPH | IR22 | 20 | 20 | 2 | 5 | 1.00 | 4.60 | 100.00 |
| 3 | WBPH | IR22 | 20 | 20 | 3 | 3 | 1.00 | 0.00 | 100.00 |
| 3 | WBPH | IR22 | 20 | 20 | 4 | 3 | 3.00 | 17.67 | 100.00 |
| 3 | WBPH | IR22 | 20 | 25 | 1 | 5 | 0.50 | 0.75 | 80.00 |
| 3 | WBPH | IR22 | 20 | 25 | 2 | 5 | 1.75 | 10.75 | 80.00 |
| 3 | WBPH | IR22 | 20 | 25 | 3 | 3 | 1.00 | 0.00 | 33.33 |
| 3 | WBPH | IR22 | 20 | 25 | 4 | 3 | 3.00 | 25.00 | 33.33 |
| 3 | WBPH | IR22 | 20 | 30 | 1 | 5 | 0.00 | 0.00 | 100.00 |
| 3 | WBPH | IR22 | 20 | 30 | 2 | 5 | 1.00 | 6.00 | 40.00 |
| 3 | WBPH | IR22 | 20 | 30 | 3 | 3 | 4.00 | 16.50 | 66.67 |
| 3 | WBPH | IR22 | 20 | 30 | 4 | 3 | 6.50 | 27.50 | 66.67 |
| 3 | WBPH | IR22 | 20 | 35 | 1 | 5 | 0.00 | 0.00 | 20.00 |
| 3 | WBPH | IR22 | 20 | 35 | 2 | 3 | 1.00 | 0.00 | 33.33 |
| 3 | WBPH | IR22 | 20 | 35 | 3 | 3 | 2.00 | 12.00 | 33.33 |
| 3 | WBPH | IR22 | 20 | 35 | 4 | 3 | 5.00 | 19.00 | 33.33 |
| 3 | WBPH | IR22 | 30 | 15 | 1 | 1 | 0.50 | 4.00 | 100.00 |
| 3 | WBPH | IR22 | 30 | 15 | 2 | 1 | 0.00 | 0.00 | 100.00 |
| 3 | WBPH | IR22 | 30 | 15 | 3 | 1 | 0.00 | 0.00 | 100.00 |
| 3 | WBPH | IR22 | 30 | 15 | 4 | 1 | 0.00 | 0.00 | 100.00 |
| 3 | WBPH | IR22 | 30 | 20 | 1 | 5 | 0.40 | 2.00 | 100.00 |
| 3 | WBPH | IR22 | 30 | 20 | 2 | 5 | 1.00 | 3.33 | 60.00 |
| 3 | WBPH | IR22 | 30 | 20 | 3 | 3 | 1.00 | 0.67 | 100.00 |
| 3 | WBPH | IR22 | 30 | 20 | 4 | 3 | 3.33 | 28.67 | 100.00 |
| 3 | WBPH | IR22 | 30 | 25 | 1 | 5 | 0.00 | 0.00 | 60.00 |
| 3 | WBPH | IR22 | 30 | 25 | 2 | 5 | 0.00 | 0.00 | 0.00 |
| 3 | WBPH | IR22 | 30 | 25 | 3 | 5 | 0.00 | 0.00 | 0.00 |
| 3 | WBPH | IR22 | 30 | 25 | 4 | 5 | 0.00 | 0.00 | 0.00 |
| 3 | WBPH | IR22 | 30 | 30 | 1 | 5 | 0.00 | 0.00 | 60.00 |
| 3 | WBPH | IR22 | 30 | 30 | 2 | 5 | 1.00 | 0.00 | 40.00 |
| 3 | WBPH | IR22 | 30 | 30 | 3 | 3 | 0.00 | 0.00 | 0.00 |
| 3 | WBPH | IR22 | 30 | 30 | 4 | 3 | 1.00 | 0.00 | 33.33 |
| 3 | WBPH | IR22 | 30 | 35 | 1 | 5 | 1.00 | 0.67 | 60.00 |
| 3 | WBPH | IR22 | 30 | 35 | 2 | 3 | 1.50 | 3.00 | 66.67 |
| 3 | WBPH | IR22 | 30 | 35 | 3 | 3 | 1.50 | 4.50 | 66.67 |
| 3 | WBPH | IR22 | 30 | 35 | 4 | 3 | 0.00 | 0.00 | 0.00 |
| 3 | WBPH | T65 | 20 | 15 | 1 | 5 | 0.40 | 1.80 | 100.00 |
| 3 | WBPH | T65 | 20 | 15 | 2 | 3 | 1.00 | 1.33 | 100.00 |
| 3 | WBPH | T65 | 20 | 15 | 3 | 3 | 1.67 | 10.67 | 100.00 |
| 3 | WBPH | T65 | 20 | 15 | 4 | 3 | 1.00 | 12.33 | 100.00 |
| 3 | WBPH | T65 | 20 | 20 | 1 | 5 | 0.20 | 0.80 | 100.00 |
| 3 | WBPH | T65 | 20 | 20 | 2 | 5 | 1.50 | 7.50 | 80.00 |
| 3 | WBPH | T65 | 20 | 20 | 3 | 3 | 1.33 | 5.00 | 100.00 |
| 3 | WBPH | T65 | 20 | 20 | 4 | 3 | 3.33 | 28.33 | 100.00 |
| 3 | WBPH | T65 | 20 | 25 | 1 | 5 | 0.80 | 3.40 | 100.00 |
| 3 | WBPH | T65 | 20 | 25 | 2 | 5 | 5.00 | 23.00 | 40.00 |
| 3 | WBPH | T65 | 20 | 25 | 3 | 3 | 1.00 | 0.00 | 100.00 |
| 3 | WBPH | T65 | 20 | 25 | 4 | 3 | 9.00 | 52.33 | 100.00 |
| 3 | WBPH | T65 | 20 | 30 | 1 | 5 | 1.80 | 7.60 | 100.00 |
| 3 | WBPH | T65 | 20 | 30 | 2 | 5 | 0.00 | 0.00 | 20.00 |
| 3 | WBPH | T65 | 20 | 30 | 3 | 3 | 1.50 | 7.50 | 66.67 |
| 3 | WBPH | T65 | 20 | 30 | 4 | 3 | 5.50 | 42.00 | 66.67 |
| 3 | WBPH | T65 | 20 | 35 | 1 | 5 | 1.20 | 5.20 | 100.00 |
| 3 | WBPH | T65 | 20 | 35 | 2 | 3 | 1.00 | 0.00 | 66.67 |
| 3 | WBPH | T65 | 20 | 35 | 3 | 3 | 1.00 | 2.50 | 66.67 |
| 3 | WBPH | T65 | 20 | 35 | 4 | 3 | 2.33 | 11.00 | 100.00 |
| 3 | WBPH | T65 | 30 | 15 | 1 | 1 | 0.50 | 4.50 | 100.00 |
| 3 | WBPH | T65 | 30 | 15 | 2 | 1 | 0.00 | 0.00 | 100.00 |
| 3 | WBPH | T65 | 30 | 15 | 3 | 1 | 0.00 | 0.00 | 100.00 |
| 3 | WBPH | T65 | 30 | 15 | 4 | 1 | 2.00 | 15.00 | 100.00 |
| 3 | WBPH | T65 | 30 | 20 | 1 | 5 | 0.20 | 1.20 | 100.00 |
| 3 | WBPH | T65 | 30 | 20 | 2 | 5 | 1.00 | 0.00 | 40.00 |
| 3 | WBPH | T65 | 30 | 20 | 3 | 3 | 1.00 | 7.33 | 100.00 |
| 3 | WBPH | T65 | 30 | 20 | 4 | 3 | 1.33 | 5.00 | 100.00 |
| 3 | WBPH | T65 | 30 | 25 | 1 | 5 | 0.40 | 1.20 | 100.00 |
| 3 | WBPH | T65 | 30 | 25 | 2 | 5 | 0.00 | 0.00 | 0.00 |
| 3 | WBPH | T65 | 30 | 25 | 3 | 5 | 0.00 | 0.00 | 0.00 |
| 3 | WBPH | T65 | 30 | 25 | 4 | 5 | 0.00 | 0.00 | 0.00 |
| 3 | WBPH | T65 | 30 | 30 | 1 | 5 | 2.00 | 7.25 | 80.00 |
| 3 | WBPH | T65 | 30 | 30 | 2 | 5 | 1.00 | 5.00 | 40.00 |
| 3 | WBPH | T65 | 30 | 30 | 3 | 3 | 1.00 | 0.00 | 66.67 |
| 3 | WBPH | T65 | 30 | 30 | 4 | 3 | 1.50 | 5.50 | 66.67 |
| 3 | WBPH | T65 | 30 | 35 | 1 | 5 | 2.00 | 6.00 | 20.00 |
| 3 | WBPH | T65 | 30 | 35 | 2 | 3 | 6.00 | 13.00 | 33.33 |
| 3 | WBPH | T65 | 30 | 35 | 3 | 3 | 4.00 | 8.00 | 33.33 |
| 3 | WBPH | T65 | 30 | 35 | 4 | 3 | 0.00 | 0.00 | 0.00 |
| 4 | BPH | IR22 | 20 | 15 | 1 | 5 | 1.00 | 2.00 | 100.00 |
| 4 | BPH | IR22 | 20 | 15 | 2 | 3 | 2.00 | 12.50 | 66.67 |
| 4 | BPH | IR22 | 20 | 15 | 3 | 3 | 1.00 | 2.00 | 100.00 |
| 4 | BPH | IR22 | 20 | 15 | 4 | 3 | 1.00 | 0.33 | 100.00 |
| 4 | BPH | IR22 | 20 | 20 | 1 | 5 | 4.00 | 12.00 | 100.00 |
| 4 | BPH | IR22 | 20 | 20 | 2 | 5 | 0.60 | 2.20 | 100.00 |
| 4 | BPH | IR22 | 20 | 20 | 3 | 3 | 3.00 | 9.67 | 100.00 |
| 4 | BPH | IR22 | 20 | 20 | 4 | 3 | 1.00 | 0.00 | 33.33 |
| 4 | BPH | IR22 | 20 | 25 | 1 | 5 | 5.60 | 13.40 | 100.00 |
| 4 | BPH | IR22 | 20 | 25 | 2 | 5 | 5.75 | 34.25 | 80.00 |
| 4 | BPH | IR22 | 20 | 25 | 3 | 3 | 1.00 | 0.00 | 100.00 |
| 4 | BPH | IR22 | 20 | 25 | 4 | 3 | 6.67 | 34.33 | 100.00 |
| 4 | BPH | IR22 | 20 | 30 | 1 | 5 | 6.00 | 19.60 | 100.00 |
| 4 | BPH | IR22 | 20 | 30 | 2 | 5 | 4.33 | 20.00 | 60.00 |
| 4 | BPH | IR22 | 20 | 30 | 3 | 3 | 2.00 | 3.00 | 33.33 |
| 4 | BPH | IR22 | 20 | 30 | 4 | 3 | 6.00 | 18.50 | 66.67 |
| 4 | BPH | IR22 | 20 | 35 | 1 | 5 | 5.00 | 16.25 | 80.00 |
| 4 | BPH | IR22 | 20 | 35 | 2 | 3 | 1.00 | 0.00 | 33.33 |
| 4 | BPH | IR22 | 20 | 35 | 3 | 3 | 3.33 | 11.33 | 100.00 |
| 4 | BPH | IR22 | 20 | 35 | 4 | 3 | 2.00 | 4.00 | 66.67 |
| 4 | BPH | IR22 | 30 | 15 | 1 | 5 | 2.50 | 8.00 | 40.00 |
| 4 | BPH | IR22 | 30 | 15 | 2 | 3 | 1.00 | 0.00 | 100.00 |
| 4 | BPH | IR22 | 30 | 15 | 3 | 3 | 1.50 | 4.50 | 66.67 |
| 4 | BPH | IR22 | 30 | 15 | 4 | 3 | 1.00 | 0.00 | 100.00 |
| 4 | BPH | IR22 | 30 | 20 | 1 | 5 | 2.60 | 10.00 | 100.00 |
| 4 | BPH | IR22 | 30 | 20 | 2 | 5 | 2.20 | 7.60 | 100.00 |
| 4 | BPH | IR22 | 30 | 20 | 3 | 3 | 1.00 | 0.00 | 100.00 |
| 4 | BPH | IR22 | 30 | 20 | 4 | 3 | 1.50 | 5.00 | 66.67 |
| 4 | BPH | IR22 | 30 | 25 | 1 | 5 | 5.00 | 20.40 | 100.00 |
| 4 | BPH | IR22 | 30 | 25 | 2 | 5 | 3.20 | 21.60 | 100.00 |
| 4 | BPH | IR22 | 30 | 25 | 3 | 3 | 5.50 | 16.00 | 66.67 |
| 4 | BPH | IR22 | 30 | 25 | 4 | 3 | 5.00 | 31.00 | 33.33 |
| 4 | BPH | IR22 | 30 | 30 | 1 | 5 | 3.60 | 13.60 | 100.00 |
| 4 | BPH | IR22 | 30 | 30 | 2 | 5 | 4.00 | 21.50 | 80.00 |
| 4 | BPH | IR22 | 30 | 30 | 3 | 3 | 1.00 | 5.00 | 33.33 |
| 4 | BPH | IR22 | 30 | 30 | 4 | 3 | 1.00 | 6.00 | 33.33 |
| 4 | BPH | IR22 | 30 | 35 | 1 | 5 | 3.40 | 14.60 | 100.00 |
| 4 | BPH | IR22 | 30 | 35 | 2 | 3 | 0.00 | 0.00 | 0.00 |
| 4 | BPH | IR22 | 30 | 35 | 3 | 3 | 1.67 | 9.33 | 100.00 |
| 4 | BPH | IR22 | 30 | 35 | 4 | 3 | 2.00 | 6.67 | 100.00 |
| 4 | BPH | T65 | 20 | 15 | 1 | 4 | 1.00 | 3.25 | 80.00 |
| 4 | BPH | T65 | 20 | 15 | 2 | 3 | 2.00 | 6.67 | 100.00 |
| 4 | BPH | T65 | 20 | 15 | 3 | 3 | 1.50 | 2.00 | 66.67 |
| 4 | BPH | T65 | 20 | 15 | 4 | 3 | 2.00 | 3.67 | 100.00 |
| 4 | BPH | T65 | 20 | 20 | 1 | 5 | 5.20 | 15.80 | 100.00 |
| 4 | BPH | T65 | 20 | 20 | 2 | 5 | 4.67 | 19.00 | 60.00 |
| 4 | BPH | T65 | 20 | 20 | 3 | 3 | 7.00 | 23.00 | 66.67 |
| 4 | BPH | T65 | 20 | 20 | 4 | 3 | 4.00 | 22.00 | 33.33 |
| 4 | BPH | T65 | 20 | 25 | 1 | 5 | 7.80 | 28.60 | 100.00 |
| 4 | BPH | T65 | 20 | 25 | 2 | 5 | 3.50 | 22.50 | 80.00 |
| 4 | BPH | T65 | 20 | 25 | 3 | 3 | 4.50 | 15.50 | 66.67 |
| 4 | BPH | T65 | 20 | 25 | 4 | 3 | 6.50 | 26.00 | 66.67 |
| 4 | BPH | T65 | 20 | 30 | 1 | 5 | 3.25 | 9.00 | 80.00 |
| 4 | BPH | T65 | 20 | 30 | 2 | 5 | 14.33 | 57.67 | 60.00 |
| 4 | BPH | T65 | 20 | 30 | 3 | 3 | 6.00 | 23.00 | 33.33 |
| 4 | BPH | T65 | 20 | 30 | 4 | 3 | 9.00 | 27.67 | 100.00 |
| 4 | BPH | T65 | 20 | 35 | 1 | 5 | 2.67 | 7.33 | 60.00 |
| 4 | BPH | T65 | 20 | 35 | 2 | 3 | 1.00 | 0.00 | 66.67 |
| 4 | BPH | T65 | 20 | 35 | 3 | 3 | 8.00 | 23.00 | 33.33 |
| 4 | BPH | T65 | 20 | 35 | 4 | 3 | 4.50 | 13.00 | 66.67 |
| 4 | BPH | T65 | 30 | 15 | 1 | 5 | 1.40 | 3.80 | 100.00 |
| 4 | BPH | T65 | 30 | 15 | 2 | 3 | 1.00 | 2.00 | 66.67 |
| 4 | BPH | T65 | 30 | 15 | 3 | 3 | 1.00 | 0.50 | 66.67 |
| 4 | BPH | T65 | 30 | 15 | 4 | 3 | 2.33 | 9.67 | 100.00 |
| 4 | BPH | T65 | 30 | 20 | 1 | 5 | 4.20 | 14.20 | 100.00 |
| 4 | BPH | T65 | 30 | 20 | 2 | 5 | 3.00 | 26.00 | 100.00 |
| 4 | BPH | T65 | 30 | 20 | 3 | 3 | 2.00 | 14.00 | 33.33 |
| 4 | BPH | T65 | 30 | 20 | 4 | 3 | 1.50 | 1.50 | 66.67 |
| 4 | BPH | T65 | 30 | 25 | 1 | 5 | 11.80 | 32.20 | 100.00 |
| 4 | BPH | T65 | 30 | 25 | 2 | 5 | 5.80 | 38.80 | 100.00 |
| 4 | BPH | T65 | 30 | 25 | 3 | 3 | 6.00 | 25.67 | 100.00 |
| 4 | BPH | T65 | 30 | 25 | 4 | 3 | 7.00 | 41.00 | 33.33 |
| 4 | BPH | T65 | 30 | 30 | 1 | 5 | 9.25 | 36.25 | 80.00 |
| 4 | BPH | T65 | 30 | 30 | 2 | 5 | 5.80 | 46.60 | 100.00 |
| 4 | BPH | T65 | 30 | 30 | 3 | 3 | 19.00 | 62.00 | 33.33 |
| 4 | BPH | T65 | 30 | 30 | 4 | 3 | 2.00 | 5.50 | 66.67 |
| 4 | BPH | T65 | 30 | 35 | 1 | 5 | 3.25 | 7.00 | 80.00 |
| 4 | BPH | T65 | 30 | 35 | 2 | 3 | 2.00 | 4.00 | 66.67 |
| 4 | BPH | T65 | 30 | 35 | 3 | 3 | 1.00 | 1.50 | 66.67 |
| 4 | BPH | T65 | 30 | 35 | 4 | 3 | 2.50 | 18.00 | 66.67 |
| 4 | WBPH | IR22 | 20 | 15 | 1 | 5 | 0.50 | 4.00 | 80.00 |
| 4 | WBPH | IR22 | 20 | 15 | 2 | 3 | 1.00 | 4.50 | 66.67 |
| 4 | WBPH | IR22 | 20 | 15 | 3 | 3 | 1.00 | 0.00 | 66.67 |
| 4 | WBPH | IR22 | 20 | 15 | 4 | 3 | 1.00 | 5.00 | 100.00 |
| 4 | WBPH | IR22 | 20 | 20 | 1 | 5 | 0.33 | 0.67 | 60.00 |
| 4 | WBPH | IR22 | 20 | 20 | 2 | 5 | 0.00 | 0.00 | 80.00 |
| 4 | WBPH | IR22 | 20 | 20 | 3 | 3 | 1.00 | 0.00 | 66.67 |
| 4 | WBPH | IR22 | 20 | 20 | 4 | 3 | 1.00 | 2.33 | 100.00 |
| 4 | WBPH | IR22 | 20 | 25 | 1 | 5 | 0.25 | 0.25 | 80.00 |
| 4 | WBPH | IR22 | 20 | 25 | 2 | 5 | 2.33 | 11.67 | 60.00 |
| 4 | WBPH | IR22 | 20 | 25 | 3 | 3 | 0.00 | 0.00 | 0.00 |
| 4 | WBPH | IR22 | 20 | 25 | 4 | 3 | 1.00 | 0.00 | 33.33 |
| 4 | WBPH | IR22 | 20 | 30 | 1 | 5 | 0.00 | 0.00 | 40.00 |
| 4 | WBPH | IR22 | 20 | 30 | 2 | 5 | 0.00 | 0.00 | 40.00 |
| 4 | WBPH | IR22 | 20 | 30 | 3 | 3 | 1.50 | 4.00 | 66.67 |
| 4 | WBPH | IR22 | 20 | 30 | 4 | 3 | 0.00 | 0.00 | 0.00 |
| 4 | WBPH | IR22 | 20 | 35 | 1 | 5 | 0.00 | 0.00 | 0.00 |
| 4 | WBPH | IR22 | 20 | 35 | 2 | 3 | 0.00 | 0.00 | 0.00 |
| 4 | WBPH | IR22 | 20 | 35 | 3 | 3 | 1.00 | 0.00 | 33.33 |
| 4 | WBPH | IR22 | 20 | 35 | 4 | 3 | 2.00 | 9.00 | 33.33 |
| 4 | WBPH | IR22 | 30 | 15 | 1 | 1 | 0.00 | 0.00 | 100.00 |
| 4 | WBPH | IR22 | 30 | 15 | 2 | 1 | 0.00 | 0.00 | 100.00 |
| 4 | WBPH | IR22 | 30 | 15 | 3 | 1 | 0.00 | 0.00 | 80.00 |
| 4 | WBPH | IR22 | 30 | 15 | 4 | 1 | 0.00 | 0.00 | 100.00 |
| 4 | WBPH | IR22 | 30 | 20 | 1 | 5 | 0.20 | 0.40 | 100.00 |
| 4 | WBPH | IR22 | 30 | 20 | 2 | 5 | 1.00 | 0.00 | 40.00 |
| 4 | WBPH | IR22 | 30 | 20 | 3 | 3 | 1.00 | 0.00 | 100.00 |
| 4 | WBPH | IR22 | 30 | 20 | 4 | 3 | 1.67 | 6.00 | 100.00 |
| 4 | WBPH | IR22 | 30 | 25 | 1 | 5 | 0.00 | 0.00 | 60.00 |
| 4 | WBPH | IR22 | 30 | 25 | 2 | 5 | 0.00 | 0.00 | 0.00 |
| 4 | WBPH | IR22 | 30 | 25 | 3 | 5 | 0.00 | 0.00 | 0.00 |
| 4 | WBPH | IR22 | 30 | 25 | 4 | 5 | 0.00 | 0.00 | 0.00 |
| 4 | WBPH | IR22 | 30 | 30 | 1 | 5 | 0.00 | 0.00 | 60.00 |
| 4 | WBPH | IR22 | 30 | 30 | 2 | 5 | 1.00 | 0.00 | 40.00 |
| 4 | WBPH | IR22 | 30 | 30 | 3 | 3 | 0.00 | 0.00 | 0.00 |
| 4 | WBPH | IR22 | 30 | 30 | 4 | 3 | 1.00 | 0.00 | 33.33 |
| 4 | WBPH | IR22 | 30 | 35 | 1 | 5 | 1.00 | 0.00 | 20.00 |
| 4 | WBPH | IR22 | 30 | 35 | 2 | 3 | 1.00 | 0.00 | 33.33 |
| 4 | WBPH | IR22 | 30 | 35 | 3 | 3 | 1.00 | 0.00 | 33.33 |
| 4 | WBPH | IR22 | 30 | 35 | 4 | 3 | 0.00 | 0.00 | 0.00 |
| 4 | WBPH | T65 | 20 | 15 | 1 | 5 | 0.00 | 0.00 | 100.00 |
| 4 | WBPH | T65 | 20 | 15 | 2 | 3 | 1.67 | 5.00 | 100.00 |
| 4 | WBPH | T65 | 20 | 15 | 3 | 3 | 1.67 | 10.00 | 100.00 |
| 4 | WBPH | T65 | 20 | 15 | 4 | 3 | 1.00 | 0.00 | 66.67 |
| 4 | WBPH | T65 | 20 | 20 | 1 | 5 | 0.20 | 0.80 | 100.00 |
| 4 | WBPH | T65 | 20 | 20 | 2 | 5 | 1.25 | 8.50 | 80.00 |
| 4 | WBPH | T65 | 20 | 20 | 3 | 3 | 1.00 | 0.00 | 100.00 |
| 4 | WBPH | T65 | 20 | 20 | 4 | 3 | 1.33 | 1.33 | 100.00 |
| 4 | WBPH | T65 | 20 | 25 | 1 | 5 | 1.60 | 6.40 | 100.00 |
| 4 | WBPH | T65 | 20 | 25 | 2 | 5 | 1.00 | 2.00 | 40.00 |
| 4 | WBPH | T65 | 20 | 25 | 3 | 3 | 1.00 | 0.00 | 33.33 |
| 4 | WBPH | T65 | 20 | 25 | 4 | 3 | 5.33 | 41.33 | 100.00 |
| 4 | WBPH | T65 | 20 | 30 | 1 | 4 | 1.50 | 6.00 | 80.00 |
| 4 | WBPH | T65 | 20 | 30 | 2 | 5 | 0.00 | 0.00 | 0.00 |
| 4 | WBPH | T65 | 20 | 30 | 3 | 3 | 1.50 | 2.00 | 66.67 |
| 4 | WBPH | T65 | 20 | 30 | 4 | 3 | 4.00 | 36.50 | 66.67 |
| 4 | WBPH | T65 | 20 | 35 | 1 | 5 | 0.00 | 0.00 | 40.00 |
| 4 | WBPH | T65 | 20 | 35 | 2 | 3 | 1.00 | 0.00 | 33.33 |
| 4 | WBPH | T65 | 20 | 35 | 3 | 3 | 5.00 | 14.00 | 33.33 |
| 4 | WBPH | T65 | 20 | 35 | 4 | 3 | 5.33 | 35.00 | 100.00 |
| 4 | WBPH | T65 | 30 | 15 | 1 | 1 | 0.50 | 2.50 | 100.00 |
| 4 | WBPH | T65 | 30 | 15 | 2 | 1 | 0.00 | 0.00 | 100.00 |
| 4 | WBPH | T65 | 30 | 15 | 3 | 1 | 0.00 | 0.00 | 100.00 |
| 4 | WBPH | T65 | 30 | 15 | 4 | 1 | 0.00 | 0.00 | 100.00 |
| 4 | WBPH | T65 | 30 | 20 | 1 | 5 | 0.40 | 0.60 | 100.00 |
| 4 | WBPH | T65 | 30 | 20 | 2 | 5 | 1.00 | 0.00 | 20.00 |
| 4 | WBPH | T65 | 30 | 20 | 3 | 3 | 1.67 | 11.33 | 100.00 |
| 4 | WBPH | T65 | 30 | 20 | 4 | 3 | 1.00 | 0.00 | 33.33 |
| 4 | WBPH | T65 | 30 | 25 | 1 | 5 | 0.00 | 0.00 | 100.00 |
| 4 | WBPH | T65 | 30 | 25 | 2 | 5 | 0.00 | 0.00 | 0.00 |
| 4 | WBPH | T65 | 30 | 25 | 3 | 5 | 0.00 | 0.00 | 0.00 |
| 4 | WBPH | T65 | 30 | 25 | 4 | 5 | 0.00 | 0.00 | 0.00 |
| 4 | WBPH | T65 | 30 | 30 | 1 | 5 | 1.33 | 5.67 | 60.00 |
| 4 | WBPH | T65 | 30 | 30 | 2 | 5 | 0.00 | 0.00 | 0.00 |
| 4 | WBPH | T65 | 30 | 30 | 3 | 3 | 1.00 | 0.00 | 33.33 |
| 4 | WBPH | T65 | 30 | 30 | 4 | 3 | 1.00 | 0.00 | 33.33 |
| 4 | WBPH | T65 | 30 | 35 | 1 | 5 | 1.00 | 0.00 | 20.00 |
| 4 | WBPH | T65 | 30 | 35 | 2 | 3 | 1.00 | 0.00 | 33.33 |
| 4 | WBPH | T65 | 30 | 35 | 3 | 3 | 1.00 | 0.00 | 33.33 |
| 4 | WBPH | T65 | 30 | 35 | 4 | 3 | 0.00 | 0.00 | 0.00 |
| 5 | BPH | IR22 | 20 | 15 | 1 | 5 | 2.00 | 7.40 | 100.00 |
| 5 | BPH | IR22 | 20 | 15 | 2 | 3 | 2.00 | 7.00 | 66.67 |
| 5 | BPH | IR22 | 20 | 15 | 3 | 3 | 1.33 | 2.00 | 100.00 |
| 5 | BPH | IR22 | 20 | 15 | 4 | 3 | 1.33 | 5.67 | 100.00 |
| 5 | BPH | IR22 | 20 | 20 | 1 | 5 | 5.60 | 16.40 | 100.00 |
| 5 | BPH | IR22 | 20 | 20 | 2 | 5 | 3.25 | 14.00 | 80.00 |
| 5 | BPH | IR22 | 20 | 20 | 3 | 3 | 3.33 | 10.67 | 100.00 |
| 5 | BPH | IR22 | 20 | 20 | 4 | 3 | 1.00 | 6.00 | 33.33 |
| 5 | BPH | IR22 | 20 | 25 | 1 | 5 | 5.60 | 17.80 | 100.00 |
| 5 | BPH | IR22 | 20 | 25 | 2 | 5 | 5.75 | 32.25 | 80.00 |
| 5 | BPH | IR22 | 20 | 25 | 3 | 3 | 1.00 | 0.00 | 66.67 |
| 5 | BPH | IR22 | 20 | 25 | 4 | 3 | 1.00 | 0.00 | 66.67 |
| 5 | BPH | IR22 | 20 | 30 | 1 | 5 | 5.40 | 19.60 | 100.00 |
| 5 | BPH | IR22 | 20 | 30 | 2 | 5 | 7.67 | 28.33 | 60.00 |
| 5 | BPH | IR22 | 20 | 30 | 3 | 3 | 2.00 | 10.00 | 33.33 |
| 5 | BPH | IR22 | 20 | 30 | 4 | 3 | 8.00 | 49.00 | 33.33 |
| 5 | BPH | IR22 | 20 | 35 | 1 | 5 | 2.67 | 8.67 | 60.00 |
| 5 | BPH | IR22 | 20 | 35 | 2 | 3 | 1.00 | 0.00 | 33.33 |
| 5 | BPH | IR22 | 20 | 35 | 3 | 3 | 1.00 | 4.00 | 66.67 |
| 5 | BPH | IR22 | 20 | 35 | 4 | 3 | 1.00 | 11.00 | 66.67 |
| 5 | BPH | IR22 | 30 | 15 | 1 | 5 | 1.00 | 2.00 | 40.00 |
| 5 | BPH | IR22 | 30 | 15 | 2 | 3 | 1.33 | 2.33 | 100.00 |
| 5 | BPH | IR22 | 30 | 15 | 3 | 3 | 2.00 | 13.00 | 66.67 |
| 5 | BPH | IR22 | 30 | 15 | 4 | 3 | 1.00 | 6.67 | 100.00 |
| 5 | BPH | IR22 | 30 | 20 | 1 | 5 | 2.00 | 11.00 | 80.00 |
| 5 | BPH | IR22 | 30 | 20 | 2 | 5 | 2.00 | 10.50 | 80.00 |
| 5 | BPH | IR22 | 30 | 20 | 3 | 3 | 1.00 | 6.50 | 66.67 |
| 5 | BPH | IR22 | 30 | 20 | 4 | 3 | 2.00 | 14.00 | 33.33 |
| 5 | BPH | IR22 | 30 | 25 | 1 | 5 | 4.25 | 16.25 | 80.00 |
| 5 | BPH | IR22 | 30 | 25 | 2 | 5 | 4.20 | 26.40 | 100.00 |
| 5 | BPH | IR22 | 30 | 25 | 3 | 3 | 2.50 | 6.50 | 66.67 |
| 5 | BPH | IR22 | 30 | 25 | 4 | 3 | 1.00 | 0.00 | 33.33 |
| 5 | BPH | IR22 | 30 | 30 | 1 | 5 | 5.25 | 23.50 | 80.00 |
| 5 | BPH | IR22 | 30 | 30 | 2 | 5 | 4.00 | 15.33 | 60.00 |
| 5 | BPH | IR22 | 30 | 30 | 3 | 3 | 1.00 | 0.00 | 33.33 |
| 5 | BPH | IR22 | 30 | 30 | 4 | 3 | 0.00 | 0.00 | 0.00 |
| 5 | BPH | IR22 | 30 | 35 | 1 | 5 | 1.60 | 4.20 | 100.00 |
| 5 | BPH | IR22 | 30 | 35 | 2 | 3 | 0.00 | 0.00 | 0.00 |
| 5 | BPH | IR22 | 30 | 35 | 3 | 3 | 1.33 | 2.67 | 100.00 |
| 5 | BPH | IR22 | 30 | 35 | 4 | 3 | 1.00 | 0.00 | 33.33 |
| 5 | BPH | T65 | 20 | 15 | 1 | 5 | 1.00 | 2.75 | 80.00 |
| 5 | BPH | T65 | 20 | 15 | 2 | 3 | 2.33 | 3.33 | 100.00 |
| 5 | BPH | T65 | 20 | 15 | 3 | 3 | 1.00 | 0.00 | 33.33 |
| 5 | BPH | T65 | 20 | 15 | 4 | 3 | 1.67 | 2.33 | 100.00 |
| 5 | BPH | T65 | 20 | 20 | 1 | 4 | 5.50 | 14.75 | 80.00 |
| 5 | BPH | T65 | 20 | 20 | 2 | 5 | 4.67 | 22.33 | 60.00 |
| 5 | BPH | T65 | 20 | 20 | 3 | 3 | 4.00 | 15.00 | 66.67 |
| 5 | BPH | T65 | 20 | 20 | 4 | 3 | 1.00 | 0.00 | 33.33 |
| 5 | BPH | T65 | 20 | 25 | 1 | 5 | 8.20 | 32.20 | 100.00 |
| 5 | BPH | T65 | 20 | 25 | 2 | 5 | 4.00 | 7.33 | 60.00 |
| 5 | BPH | T65 | 20 | 25 | 3 | 3 | 8.00 | 24.00 | 33.33 |
| 5 | BPH | T65 | 20 | 25 | 4 | 3 | 1.00 | 0.00 | 33.33 |
| 5 | BPH | T65 | 20 | 30 | 1 | 5 | 8.50 | 22.00 | 40.00 |
| 5 | BPH | T65 | 20 | 30 | 2 | 5 | 9.33 | 52.67 | 60.00 |
| 5 | BPH | T65 | 20 | 30 | 3 | 3 | 1.00 | 11.00 | 33.33 |
| 5 | BPH | T65 | 20 | 30 | 4 | 3 | 13.00 | 32.00 | 66.67 |
| 5 | BPH | T65 | 20 | 35 | 1 | 5 | 1.00 | 2.00 | 40.00 |
| 5 | BPH | T65 | 20 | 35 | 2 | 3 | 5.00 | 24.00 | 33.33 |
| 5 | BPH | T65 | 20 | 35 | 3 | 3 | 0.00 | 0.00 | 0.00 |
| 5 | BPH | T65 | 20 | 35 | 4 | 3 | 1.50 | 4.00 | 66.67 |
| 5 | BPH | T65 | 30 | 15 | 1 | 5 | 1.40 | 4.60 | 100.00 |
| 5 | BPH | T65 | 30 | 15 | 2 | 3 | 1.00 | 9.50 | 66.67 |
| 5 | BPH | T65 | 30 | 15 | 3 | 3 | 6.50 | 19.00 | 66.67 |
| 5 | BPH | T65 | 30 | 15 | 4 | 3 | 4.00 | 27.33 | 100.00 |
| 5 | BPH | T65 | 30 | 20 | 1 | 5 | 1.60 | 5.60 | 100.00 |
| 5 | BPH | T65 | 30 | 20 | 2 | 5 | 4.20 | 23.80 | 100.00 |
| 5 | BPH | T65 | 30 | 20 | 3 | 3 | 2.00 | 23.00 | 33.33 |
| 5 | BPH | T65 | 30 | 20 | 4 | 3 | 1.00 | 0.00 | 66.67 |
| 5 | BPH | T65 | 30 | 25 | 1 | 5 | 8.40 | 30.40 | 100.00 |
| 5 | BPH | T65 | 30 | 25 | 2 | 5 | 3.20 | 28.00 | 100.00 |
| 5 | BPH | T65 | 30 | 25 | 3 | 3 | 5.00 | 8.00 | 66.67 |
| 5 | BPH | T65 | 30 | 25 | 4 | 3 | 4.00 | 24.00 | 33.33 |
| 5 | BPH | T65 | 30 | 30 | 1 | 5 | 5.75 | 26.00 | 80.00 |
| 5 | BPH | T65 | 30 | 30 | 2 | 5 | 6.75 | 53.50 | 80.00 |
| 5 | BPH | T65 | 30 | 30 | 3 | 3 | 7.00 | 26.00 | 33.33 |
| 5 | BPH | T65 | 30 | 30 | 4 | 3 | 1.00 | 0.00 | 33.33 |
| 5 | BPH | T65 | 30 | 35 | 1 | 5 | 1.67 | 4.33 | 60.00 |
| 5 | BPH | T65 | 30 | 35 | 2 | 3 | 1.50 | 3.50 | 66.67 |
| 5 | BPH | T65 | 30 | 35 | 3 | 3 | 0.00 | 0.00 | 0.00 |
| 5 | BPH | T65 | 30 | 35 | 4 | 3 | 1.00 | 0.00 | 66.67 |
| 5 | WBPH | IR22 | 20 | 15 | 1 | 5 | 0.00 | 0.00 | 80.00 |
| 5 | WBPH | IR22 | 20 | 15 | 2 | 3 | 1.00 | 4.00 | 66.67 |
| 5 | WBPH | IR22 | 20 | 15 | 3 | 3 | 1.00 | 0.00 | 66.67 |
| 5 | WBPH | IR22 | 20 | 15 | 4 | 3 | 1.00 | 0.00 | 100.00 |
| 5 | WBPH | IR22 | 20 | 20 | 1 | 5 | 0.33 | 0.33 | 60.00 |
| 5 | WBPH | IR22 | 20 | 20 | 2 | 5 | 0.50 | 0.50 | 40.00 |
| 5 | WBPH | IR22 | 20 | 20 | 3 | 3 | 1.00 | 0.00 | 66.67 |
| 5 | WBPH | IR22 | 20 | 20 | 4 | 3 | 1.33 | 5.67 | 100.00 |
| 5 | WBPH | IR22 | 20 | 25 | 1 | 5 | 1.00 | 2.50 | 40.00 |
| 5 | WBPH | IR22 | 20 | 25 | 2 | 5 | 2.00 | 16.50 | 40.00 |
| 5 | WBPH | IR22 | 20 | 25 | 3 | 3 | 0.00 | 0.00 | 0.00 |
| 5 | WBPH | IR22 | 20 | 25 | 4 | 3 | 1.00 | 17.00 | 33.33 |
| 5 | WBPH | IR22 | 20 | 30 | 1 | 5 | 2.00 | 18.00 | 20.00 |
| 5 | WBPH | IR22 | 20 | 30 | 2 | 5 | 0.00 | 0.00 | 0.00 |
| 5 | WBPH | IR22 | 20 | 30 | 3 | 3 | 1.00 | 2.00 | 66.67 |
| 5 | WBPH | IR22 | 20 | 30 | 4 | 3 | 0.00 | 0.00 | 0.00 |
| 5 | WBPH | IR22 | 20 | 35 | 1 | 5 | 0.00 | 0.00 | 0.00 |
| 5 | WBPH | IR22 | 20 | 35 | 2 | 3 | 0.00 | 0.00 | 0.00 |
| 5 | WBPH | IR22 | 20 | 35 | 3 | 3 | 1.00 | 0.00 | 33.33 |
| 5 | WBPH | IR22 | 20 | 35 | 4 | 3 | 1.00 | 0.00 | 33.33 |
| 5 | WBPH | IR22 | 30 | 15 | 1 | 1 | 0.50 | 1.50 | 100.00 |
| 5 | WBPH | IR22 | 30 | 15 | 2 | 1 | 0.00 | 0.00 | 100.00 |
| 5 | WBPH | IR22 | 30 | 15 | 3 | 1 | 0.00 | 0.00 | 80.00 |
| 5 | WBPH | IR22 | 30 | 15 | 4 | 1 | 1.00 | 2.00 | 100.00 |
| 5 | WBPH | IR22 | 30 | 20 | 1 | 5 | 0.40 | 2.60 | 100.00 |
| 5 | WBPH | IR22 | 30 | 20 | 2 | 5 | 1.00 | 0.00 | 20.00 |
| 5 | WBPH | IR22 | 30 | 20 | 3 | 3 | 1.00 | 0.00 | 100.00 |
| 5 | WBPH | IR22 | 30 | 20 | 4 | 3 | 1.67 | 17.67 | 100.00 |
| 5 | WBPH | IR22 | 30 | 25 | 1 | 5 | 0.00 | 0.00 | 0.00 |
| 5 | WBPH | IR22 | 30 | 25 | 2 | 5 | 0.00 | 0.00 | 0.00 |
| 5 | WBPH | IR22 | 30 | 25 | 3 | 5 | 0.00 | 0.00 | 0.00 |
| 5 | WBPH | IR22 | 30 | 25 | 4 | 5 | 0.00 | 0.00 | 0.00 |
| 5 | WBPH | IR22 | 30 | 30 | 1 | 1 | 0.00 | 0.00 | 20.00 |
| 5 | WBPH | IR22 | 30 | 30 | 2 | 5 | 0.00 | 0.00 | 0.00 |
| 5 | WBPH | IR22 | 30 | 30 | 3 | 3 | 0.00 | 0.00 | 0.00 |
| 5 | WBPH | IR22 | 30 | 30 | 4 | 3 | 0.00 | 0.00 | 0.00 |
| 5 | WBPH | IR22 | 30 | 35 | 1 | 5 | 0.00 | 0.00 | 0.00 |
| 5 | WBPH | IR22 | 30 | 35 | 2 | 3 | 1.00 | 0.00 | 33.33 |
| 5 | WBPH | IR22 | 30 | 35 | 3 | 3 | 0.00 | 0.00 | 0.00 |
| 5 | WBPH | IR22 | 30 | 35 | 4 | 3 | 0.00 | 0.00 | 0.00 |
| 5 | WBPH | T65 | 20 | 15 | 1 | 5 | 0.40 | 1.20 | 100.00 |
| 5 | WBPH | T65 | 20 | 15 | 2 | 3 | 1.00 | 2.00 | 100.00 |
| 5 | WBPH | T65 | 20 | 15 | 3 | 3 | 2.00 | 16.67 | 100.00 |
| 5 | WBPH | T65 | 20 | 15 | 4 | 3 | 1.00 | 7.50 | 66.67 |
| 5 | WBPH | T65 | 20 | 20 | 1 | 5 | 0.00 | 0.00 | 100.00 |
| 5 | WBPH | T65 | 20 | 20 | 2 | 5 | 0.00 | 0.00 | 40.00 |
| 5 | WBPH | T65 | 20 | 20 | 3 | 3 | 1.00 | 5.33 | 100.00 |
| 5 | WBPH | T65 | 20 | 20 | 4 | 3 | 2.00 | 21.50 | 66.67 |
| 5 | WBPH | T65 | 20 | 25 | 1 | 5 | 2.80 | 10.80 | 100.00 |
| 5 | WBPH | T65 | 20 | 25 | 2 | 5 | 0.00 | 0.00 | 40.00 |
| 5 | WBPH | T65 | 20 | 25 | 3 | 3 | 0.00 | 0.00 | 0.00 |
| 5 | WBPH | T65 | 20 | 25 | 4 | 3 | 9.33 | 53.00 | 100.00 |
| 5 | WBPH | T65 | 20 | 30 | 1 | 5 | 1.00 | 1.00 | 40.00 |
| 5 | WBPH | T65 | 20 | 30 | 2 | 5 | 0.00 | 0.00 | 0.00 |
| 5 | WBPH | T65 | 20 | 30 | 3 | 3 | 1.50 | 3.50 | 66.67 |
| 5 | WBPH | T65 | 20 | 30 | 4 | 3 | 5.00 | 54.50 | 66.67 |
| 5 | WBPH | T65 | 20 | 35 | 1 | 5 | 0.00 | 0.00 | 20.00 |
| 5 | WBPH | T65 | 20 | 35 | 2 | 3 | 1.00 | 7.00 | 33.33 |
| 5 | WBPH | T65 | 20 | 35 | 3 | 3 | 1.00 | 0.00 | 33.33 |
| 5 | WBPH | T65 | 20 | 35 | 4 | 3 | 3.33 | 16.67 | 100.00 |
| 5 | WBPH | T65 | 30 | 15 | 1 | 1 | 0.00 | 0.00 | 100.00 |
| 5 | WBPH | T65 | 30 | 15 | 2 | 1 | 1.00 | 4.00 | 100.00 |
| 5 | WBPH | T65 | 30 | 15 | 3 | 1 | 0.00 | 0.00 | 100.00 |
| 5 | WBPH | T65 | 30 | 15 | 4 | 1 | 0.00 | 0.00 | 100.00 |
| 5 | WBPH | T65 | 30 | 20 | 1 | 5 | 0.33 | 1.00 | 60.00 |
| 5 | WBPH | T65 | 30 | 20 | 2 | 5 | 1.00 | 0.00 | 20.00 |
| 5 | WBPH | T65 | 30 | 20 | 3 | 3 | 1.67 | 10.33 | 100.00 |
| 5 | WBPH | T65 | 30 | 20 | 4 | 3 | 3.00 | 15.00 | 33.33 |
| 5 | WBPH | T65 | 30 | 25 | 1 | 5 | 0.00 | 0.00 | 80.00 |
| 5 | WBPH | T65 | 30 | 25 | 2 | 5 | 0.00 | 0.00 | 0.00 |
| 5 | WBPH | T65 | 30 | 25 | 3 | 5 | 0.00 | 0.00 | 0.00 |
| 5 | WBPH | T65 | 30 | 25 | 4 | 5 | 0.00 | 0.00 | 0.00 |
| 5 | WBPH | T65 | 30 | 30 | 1 | 5 | 0.67 | 0.67 | 60.00 |
| 5 | WBPH | T65 | 30 | 30 | 2 | 5 | 0.00 | 0.00 | 0.00 |
| 5 | WBPH | T65 | 30 | 30 | 3 | 3 | 0.00 | 0.00 | 0.00 |
| 5 | WBPH | T65 | 30 | 30 | 4 | 3 | 1.00 | 0.00 | 33.33 |
| 5 | WBPH | T65 | 30 | 35 | 1 | 5 | 3.00 | 10.00 | 20.00 |
| 5 | WBPH | T65 | 30 | 35 | 2 | 3 | 1.00 | 0.00 | 33.33 |
| 5 | WBPH | T65 | 30 | 35 | 3 | 3 | 1.00 | 0.00 | 33.33 |
| 5 | WBPH | T65 | 30 | 35 | 4 | 3 | 0.00 | 0.00 | 0.00 |
| 6 | BPH | IR22 | 20 | 15 | 1 | 5 | 1.80 | 4.60 | 100.00 |
| 6 | BPH | IR22 | 20 | 15 | 2 | 3 | 3.00 | 17.00 | 66.67 |
| 6 | BPH | IR22 | 20 | 15 | 3 | 3 | 1.00 | 1.00 | 66.67 |
| 6 | BPH | IR22 | 20 | 15 | 4 | 3 | 1.67 | 6.67 | 100.00 |
| 6 | BPH | IR22 | 20 | 20 | 1 | 5 | 3.80 | 8.60 | 100.00 |
| 6 | BPH | IR22 | 20 | 20 | 2 | 5 | 3.67 | 14.00 | 60.00 |
| 6 | BPH | IR22 | 20 | 20 | 3 | 3 | 2.33 | 6.00 | 100.00 |
| 6 | BPH | IR22 | 20 | 20 | 4 | 3 | 1.00 | 0.00 | 33.33 |
| 6 | BPH | IR22 | 20 | 25 | 1 | 5 | 7.40 | 16.80 | 100.00 |
| 6 | BPH | IR22 | 20 | 25 | 2 | 5 | 5.00 | 23.75 | 80.00 |
| 6 | BPH | IR22 | 20 | 25 | 3 | 3 | 0.00 | 0.00 | 0.00 |
| 6 | BPH | IR22 | 20 | 25 | 4 | 3 | 1.00 | 0.00 | 33.33 |
| 6 | BPH | IR22 | 20 | 30 | 1 | 5 | 5.50 | 16.50 | 80.00 |
| 6 | BPH | IR22 | 20 | 30 | 2 | 5 | 5.67 | 26.67 | 60.00 |
| 6 | BPH | IR22 | 20 | 30 | 3 | 3 | 1.00 | 5.00 | 33.33 |
| 6 | BPH | IR22 | 20 | 30 | 4 | 3 | 1.00 | 0.00 | 33.33 |
| 6 | BPH | IR22 | 20 | 35 | 1 | 5 | 1.33 | 2.33 | 60.00 |
| 6 | BPH | IR22 | 20 | 35 | 2 | 3 | 1.00 | 0.00 | 33.33 |
| 6 | BPH | IR22 | 20 | 35 | 3 | 3 | 1.00 | 1.50 | 66.67 |
| 6 | BPH | IR22 | 20 | 35 | 4 | 3 | 0.00 | 0.00 | 0.00 |
| 6 | BPH | IR22 | 30 | 15 | 1 | 5 | 0.89 | 1.89 | 40.00 |
| 6 | BPH | IR22 | 30 | 15 | 2 | 3 | 2.00 | 4.67 | 100.00 |
| 6 | BPH | IR22 | 30 | 15 | 3 | 3 | 6.50 | 29.50 | 66.67 |
| 6 | BPH | IR22 | 30 | 15 | 4 | 3 | 1.00 | 1.00 | 100.00 |
| 6 | BPH | IR22 | 30 | 20 | 1 | 5 | 2.00 | 6.75 | 80.00 |
| 6 | BPH | IR22 | 30 | 20 | 2 | 5 | 1.50 | 7.75 | 80.00 |
| 6 | BPH | IR22 | 30 | 20 | 3 | 3 | 3.00 | 13.00 | 33.33 |
| 6 | BPH | IR22 | 30 | 20 | 4 | 3 | 1.00 | 0.00 | 33.33 |
| 6 | BPH | IR22 | 30 | 25 | 1 | 5 | 10.00 | 43.75 | 80.00 |
| 6 | BPH | IR22 | 30 | 25 | 2 | 5 | 2.60 | 13.20 | 100.00 |
| 6 | BPH | IR22 | 30 | 25 | 3 | 3 | 3.00 | 16.00 | 33.33 |
| 6 | BPH | IR22 | 30 | 25 | 4 | 3 | 2.00 | 5.00 | 33.33 |
| 6 | BPH | IR22 | 30 | 30 | 1 | 5 | 2.25 | 9.00 | 80.00 |
| 6 | BPH | IR22 | 30 | 30 | 2 | 5 | 4.33 | 25.67 | 60.00 |
| 6 | BPH | IR22 | 30 | 30 | 3 | 3 | 0.00 | 0.00 | 0.00 |
| 6 | BPH | IR22 | 30 | 30 | 4 | 3 | 0.00 | 0.00 | 0.00 |
| 6 | BPH | IR22 | 30 | 35 | 1 | 5 | 1.00 | 1.00 | 60.00 |
| 6 | BPH | IR22 | 30 | 35 | 2 | 3 | 0.00 | 0.00 | 0.00 |
| 6 | BPH | IR22 | 30 | 35 | 3 | 3 | 1.00 | 0.00 | 100.00 |
| 6 | BPH | IR22 | 30 | 35 | 4 | 3 | 2.00 | 2.00 | 33.33 |
| 6 | BPH | T65 | 20 | 15 | 1 | 5 | 1.00 | 3.67 | 60.00 |
| 6 | BPH | T65 | 20 | 15 | 2 | 3 | 1.33 | 2.00 | 100.00 |
| 6 | BPH | T65 | 20 | 15 | 3 | 3 | 1.00 | 0.00 | 33.33 |
| 6 | BPH | T65 | 20 | 15 | 4 | 3 | 1.00 | 1.67 | 100.00 |
| 6 | BPH | T65 | 20 | 20 | 1 | 5 | 3.00 | 8.50 | 80.00 |
| 6 | BPH | T65 | 20 | 20 | 2 | 5 | 3.67 | 14.67 | 60.00 |
| 6 | BPH | T65 | 20 | 20 | 3 | 3 | 3.50 | 10.50 | 66.67 |
| 6 | BPH | T65 | 20 | 20 | 4 | 3 | 3.00 | 12.00 | 33.33 |
| 6 | BPH | T65 | 20 | 25 | 1 | 5 | 15.20 | 46.00 | 100.00 |
| 6 | BPH | T65 | 20 | 25 | 2 | 5 | 3.00 | 6.50 | 40.00 |
| 6 | BPH | T65 | 20 | 25 | 3 | 3 | 5.00 | 17.00 | 33.33 |
| 6 | BPH | T65 | 20 | 25 | 4 | 3 | 0.00 | 0.00 | 0.00 |
| 6 | BPH | T65 | 20 | 30 | 1 | 5 | 5.00 | 20.50 | 40.00 |
| 6 | BPH | T65 | 20 | 30 | 2 | 5 | 11.33 | 41.00 | 60.00 |
| 6 | BPH | T65 | 20 | 30 | 3 | 3 | 8.00 | 21.00 | 33.33 |
| 6 | BPH | T65 | 20 | 30 | 4 | 3 | 0.00 | 0.00 | 0.00 |
| 6 | BPH | T65 | 20 | 35 | 1 | 5 | 0.00 | 0.00 | 20.00 |
| 6 | BPH | T65 | 20 | 35 | 2 | 3 | 1.00 | 0.00 | 33.33 |
| 6 | BPH | T65 | 20 | 35 | 3 | 3 | 0.00 | 0.00 | 0.00 |
| 6 | BPH | T65 | 20 | 35 | 4 | 3 | 0.00 | 0.00 | 0.00 |
| 6 | BPH | T65 | 30 | 15 | 1 | 5 | 1.20 | 4.60 | 100.00 |
| 6 | BPH | T65 | 30 | 15 | 2 | 3 | 1.00 | 2.50 | 66.67 |
| 6 | BPH | T65 | 30 | 15 | 3 | 3 | 4.00 | 6.50 | 66.67 |
| 6 | BPH | T65 | 30 | 15 | 4 | 3 | 2.00 | 2.50 | 66.67 |
| 6 | BPH | T65 | 30 | 20 | 1 | 5 | 5.40 | 12.80 | 100.00 |
| 6 | BPH | T65 | 30 | 20 | 2 | 5 | 3.60 | 22.40 | 100.00 |
| 6 | BPH | T65 | 30 | 20 | 3 | 3 | 1.00 | 0.00 | 33.33 |
| 6 | BPH | T65 | 30 | 20 | 4 | 3 | 1.00 | 0.00 | 66.67 |
| 6 | BPH | T65 | 30 | 25 | 1 | 5 | 6.80 | 18.60 | 100.00 |
| 6 | BPH | T65 | 30 | 25 | 2 | 5 | 4.00 | 19.60 | 100.00 |
| 6 | BPH | T65 | 30 | 25 | 3 | 3 | 1.00 | 0.00 | 66.67 |
| 6 | BPH | T65 | 30 | 25 | 4 | 3 | 2.00 | 25.00 | 33.33 |
| 6 | BPH | T65 | 30 | 30 | 1 | 5 | 5.33 | 29.00 | 60.00 |
| 6 | BPH | T65 | 30 | 30 | 2 | 5 | 6.00 | 46.75 | 80.00 |
| 6 | BPH | T65 | 30 | 30 | 3 | 3 | 11.00 | 44.00 | 33.33 |
| 6 | BPH | T65 | 30 | 30 | 4 | 3 | 1.00 | 5.00 | 33.33 |
| 6 | BPH | T65 | 30 | 35 | 1 | 5 | 0.67 | 1.33 | 60.00 |
| 6 | BPH | T65 | 30 | 35 | 2 | 3 | 1.00 | 5.00 | 66.67 |
| 6 | BPH | T65 | 30 | 35 | 3 | 3 | 0.00 | 0.00 | 0.00 |
| 6 | BPH | T65 | 30 | 35 | 4 | 3 | 1.00 | 0.00 | 33.33 |
| 6 | WBPH | IR22 | 20 | 15 | 1 | 5 | 0.25 | 1.00 | 80.00 |
| 6 | WBPH | IR22 | 20 | 15 | 2 | 3 | 1.00 | 2.00 | 66.67 |
| 6 | WBPH | IR22 | 20 | 15 | 3 | 3 | 1.00 | 0.00 | 33.33 |
| 6 | WBPH | IR22 | 20 | 15 | 4 | 3 | 1.00 | 1.33 | 100.00 |
| 6 | WBPH | IR22 | 20 | 20 | 1 | 5 | 0.00 | 0.00 | 40.00 |
| 6 | WBPH | IR22 | 20 | 20 | 2 | 5 | 0.00 | 0.00 | 20.00 |
| 6 | WBPH | IR22 | 20 | 20 | 3 | 3 | 1.00 | 0.00 | 33.33 |
| 6 | WBPH | IR22 | 20 | 20 | 4 | 3 | 1.00 | 1.33 | 100.00 |
| 6 | WBPH | IR22 | 20 | 25 | 1 | 5 | 0.00 | 0.00 | 20.00 |
| 6 | WBPH | IR22 | 20 | 25 | 2 | 5 | 6.00 | 16.00 | 20.00 |
| 6 | WBPH | IR22 | 20 | 25 | 3 | 3 | 0.00 | 0.00 | 0.00 |
| 6 | WBPH | IR22 | 20 | 25 | 4 | 3 | 2.00 | 31.00 | 33.33 |
| 6 | WBPH | IR22 | 20 | 30 | 1 | 5 | 0.00 | 0.00 | 0.00 |
| 6 | WBPH | IR22 | 20 | 30 | 2 | 5 | 0.00 | 0.00 | 0.00 |
| 6 | WBPH | IR22 | 20 | 30 | 3 | 3 | 1.00 | 2.00 | 66.67 |
| 6 | WBPH | IR22 | 20 | 30 | 4 | 3 | 0.00 | 0.00 | 0.00 |
| 6 | WBPH | IR22 | 20 | 35 | 1 | 5 | 0.00 | 0.00 | 0.00 |
| 6 | WBPH | IR22 | 20 | 35 | 2 | 3 | 0.00 | 0.00 | 0.00 |
| 6 | WBPH | IR22 | 20 | 35 | 3 | 3 | 1.00 | 0.00 | 33.33 |
| 6 | WBPH | IR22 | 20 | 35 | 4 | 3 | 1.00 | 0.00 | 33.33 |
| 6 | WBPH | IR22 | 30 | 15 | 1 | 1 | 0.00 | 0.00 | 100.00 |
| 6 | WBPH | IR22 | 30 | 15 | 2 | 1 | 0.00 | 0.00 | 100.00 |
| 6 | WBPH | IR22 | 30 | 15 | 3 | 1 | 0.00 | 0.00 | 80.00 |
| 6 | WBPH | IR22 | 30 | 15 | 4 | 1 | 0.00 | 0.00 | 80.00 |
| 6 | WBPH | IR22 | 30 | 20 | 1 | 5 | 0.00 | 0.00 | 100.00 |
| 6 | WBPH | IR22 | 30 | 20 | 2 | 5 | 0.00 | 0.00 | 0.00 |
| 6 | WBPH | IR22 | 30 | 20 | 3 | 3 | 1.00 | 0.00 | 100.00 |
| 6 | WBPH | IR22 | 30 | 20 | 4 | 3 | 2.33 | 4.33 | 100.00 |
| 6 | WBPH | IR22 | 30 | 25 | 1 | 5 | 0.00 | 0.00 | 0.00 |
| 6 | WBPH | IR22 | 30 | 25 | 2 | 5 | 0.00 | 0.00 | 0.00 |
| 6 | WBPH | IR22 | 30 | 25 | 3 | 5 | 0.00 | 0.00 | 0.00 |
| 6 | WBPH | IR22 | 30 | 25 | 4 | 5 | 0.00 | 0.00 | 0.00 |
| 6 | WBPH | IR22 | 30 | 30 | 1 | 5 | 0.00 | 0.00 | 20.00 |
| 6 | WBPH | IR22 | 30 | 30 | 2 | 5 | 0.00 | 0.00 | 0.00 |
| 6 | WBPH | IR22 | 30 | 30 | 3 | 3 | 0.00 | 0.00 | 0.00 |
| 6 | WBPH | IR22 | 30 | 30 | 4 | 3 | 0.00 | 0.00 | 0.00 |
| 6 | WBPH | IR22 | 30 | 35 | 1 | 5 | 0.00 | 0.00 | 0.00 |
| 6 | WBPH | IR22 | 30 | 35 | 2 | 3 | 1.00 | 0.00 | 33.33 |
| 6 | WBPH | IR22 | 30 | 35 | 3 | 3 | 0.00 | 0.00 | 0.00 |
| 6 | WBPH | IR22 | 30 | 35 | 4 | 3 | 0.00 | 0.00 | 0.00 |
| 6 | WBPH | T65 | 20 | 15 | 1 | 5 | 0.40 | 1.40 | 100.00 |
| 6 | WBPH | T65 | 20 | 15 | 2 | 3 | 1.33 | 4.67 | 100.00 |
| 6 | WBPH | T65 | 20 | 15 | 3 | 3 | 1.33 | 7.00 | 100.00 |
| 6 | WBPH | T65 | 20 | 15 | 4 | 3 | 1.00 | 0.00 | 66.67 |
| 6 | WBPH | T65 | 20 | 20 | 1 | 5 | 0.00 | 0.00 | 60.00 |
| 6 | WBPH | T65 | 20 | 20 | 2 | 5 | 1.50 | 5.00 | 40.00 |
| 6 | WBPH | T65 | 20 | 20 | 3 | 3 | 1.00 | 8.00 | 66.67 |
| 6 | WBPH | T65 | 20 | 20 | 4 | 3 | 1.00 | 0.00 | 66.67 |
| 6 | WBPH | T65 | 20 | 25 | 1 | 5 | 2.40 | 11.20 | 100.00 |
| 6 | WBPH | T65 | 20 | 25 | 2 | 5 | 0.00 | 0.00 | 20.00 |
| 6 | WBPH | T65 | 20 | 25 | 3 | 3 | 0.00 | 0.00 | 0.00 |
| 6 | WBPH | T65 | 20 | 25 | 4 | 3 | 3.67 | 30.67 | 100.00 |
| 6 | WBPH | T65 | 20 | 30 | 1 | 5 | 1.00 | 4.00 | 20.00 |
| 6 | WBPH | T65 | 20 | 30 | 2 | 5 | 0.00 | 0.00 | 0.00 |
| 6 | WBPH | T65 | 20 | 30 | 3 | 3 | 1.00 | 3.00 | 66.67 |
| 6 | WBPH | T65 | 20 | 30 | 4 | 3 | 1.00 | 4.00 | 66.67 |
| 6 | WBPH | T65 | 20 | 35 | 1 | 5 | 0.00 | 0.00 | 0.00 |
| 6 | WBPH | T65 | 20 | 35 | 2 | 3 | 1.00 | 0.00 | 33.33 |
| 6 | WBPH | T65 | 20 | 35 | 3 | 3 | 0.00 | 0.00 | 0.00 |
| 6 | WBPH | T65 | 20 | 35 | 4 | 3 | 1.00 | 2.67 | 100.00 |
| 6 | WBPH | T65 | 30 | 15 | 1 | 1 | 1.00 | 4.00 | 100.00 |
| 6 | WBPH | T65 | 30 | 15 | 2 | 1 | 0.00 | 0.00 | 100.00 |
| 6 | WBPH | T65 | 30 | 15 | 3 | 1 | 0.00 | 0.00 | 80.00 |
| 6 | WBPH | T65 | 30 | 15 | 4 | 1 | 0.00 | 0.00 | 100.00 |
| 6 | WBPH | T65 | 30 | 20 | 1 | 5 | 0.67 | 2.00 | 60.00 |
| 6 | WBPH | T65 | 30 | 20 | 2 | 5 | 1.00 | 0.00 | 20.00 |
| 6 | WBPH | T65 | 30 | 20 | 3 | 3 | 1.00 | 3.50 | 66.67 |
| 6 | WBPH | T65 | 30 | 20 | 4 | 3 | 1.00 | 0.00 | 33.33 |
| 6 | WBPH | T65 | 30 | 25 | 1 | 5 | 0.25 | 0.75 | 80.00 |
| 6 | WBPH | T65 | 30 | 25 | 2 | 5 | 0.00 | 0.00 | 0.00 |
| 6 | WBPH | T65 | 30 | 25 | 3 | 5 | 0.00 | 0.00 | 0.00 |
| 6 | WBPH | T65 | 30 | 25 | 4 | 5 | 0.00 | 0.00 | 0.00 |
| 6 | WBPH | T65 | 30 | 30 | 1 | 5 | 2.00 | 7.00 | 40.00 |
| 6 | WBPH | T65 | 30 | 30 | 2 | 5 | 0.00 | 0.00 | 0.00 |
| 6 | WBPH | T65 | 30 | 30 | 3 | 3 | 0.00 | 0.00 | 0.00 |
| 6 | WBPH | T65 | 30 | 30 | 4 | 3 | 0.00 | 0.00 | 0.00 |
| 6 | WBPH | T65 | 30 | 35 | 1 | 5 | 1.00 | 0.00 | 20.00 |
| 6 | WBPH | T65 | 30 | 35 | 2 | 3 | 1.00 | 0.00 | 33.33 |
| 6 | WBPH | T65 | 30 | 35 | 3 | 3 | 1.00 | 0.00 | 33.33 |
| 6 | WBPH | T65 | 30 | 35 | 4 | 3 | 0.00 | 0.00 | 0.00 |
| 7 | BPH | IR22 | 20 | 15 | 1 | 5 | 0.75 | 1.50 | 80.00 |
| 7 | BPH | IR22 | 20 | 15 | 2 | 3 | 1.50 | 2.00 | 66.67 |
| 7 | BPH | IR22 | 20 | 15 | 3 | 3 | 1.00 | 0.00 | 33.33 |
| 7 | BPH | IR22 | 20 | 15 | 4 | 3 | 2.00 | 9.67 | 100.00 |
| 7 | BPH | IR22 | 20 | 20 | 1 | 5 | 3.75 | 9.75 | 80.00 |
| 7 | BPH | IR22 | 20 | 20 | 2 | 5 | 4.67 | 16.33 | 60.00 |
| 7 | BPH | IR22 | 20 | 20 | 3 | 3 | 4.00 | 10.00 | 66.67 |
| 7 | BPH | IR22 | 20 | 20 | 4 | 3 | 0.00 | 0.00 | 0.00 |
| 7 | BPH | IR22 | 20 | 25 | 1 | 5 | 5.00 | 14.80 | 100.00 |
| 7 | BPH | IR22 | 20 | 25 | 2 | 5 | 4.50 | 20.50 | 80.00 |
| 7 | BPH | IR22 | 20 | 25 | 3 | 3 | 0.00 | 0.00 | 0.00 |
| 7 | BPH | IR22 | 20 | 25 | 4 | 3 | 0.00 | 0.00 | 0.00 |
| 7 | BPH | IR22 | 20 | 30 | 1 | 5 | 4.00 | 13.75 | 80.00 |
| 7 | BPH | IR22 | 20 | 30 | 2 | 5 | 2.67 | 9.67 | 60.00 |
| 7 | BPH | IR22 | 20 | 30 | 3 | 3 | 1.00 | 0.00 | 33.33 |
| 7 | BPH | IR22 | 20 | 30 | 4 | 3 | 0.00 | 0.00 | 0.00 |
| 7 | BPH | IR22 | 20 | 35 | 1 | 5 | 1.00 | 2.00 | 20.00 |
| 7 | BPH | IR22 | 20 | 35 | 2 | 3 | 0.00 | 0.00 | 0.00 |
| 7 | BPH | IR22 | 20 | 35 | 3 | 3 | 0.00 | 0.00 | 0.00 |
| 7 | BPH | IR22 | 20 | 35 | 4 | 3 | 0.00 | 0.00 | 0.00 |
| 7 | BPH | IR22 | 30 | 15 | 1 | 5 | 0.50 | 2.50 | 40.00 |
| 7 | BPH | IR22 | 30 | 15 | 2 | 3 | 1.33 | 5.00 | 100.00 |
| 7 | BPH | IR22 | 30 | 15 | 3 | 3 | 3.00 | 8.00 | 66.67 |
| 7 | BPH | IR22 | 30 | 15 | 4 | 3 | 1.00 | 0.00 | 100.00 |
| 7 | BPH | IR22 | 30 | 20 | 1 | 5 | 2.00 | 7.67 | 60.00 |
| 7 | BPH | IR22 | 30 | 20 | 2 | 5 | 2.00 | 8.00 | 80.00 |
| 7 | BPH | IR22 | 30 | 20 | 3 | 3 | 2.00 | 5.00 | 33.33 |
| 7 | BPH | IR22 | 30 | 20 | 4 | 3 | 2.00 | 16.00 | 33.33 |
| 7 | BPH | IR22 | 30 | 25 | 1 | 5 | 5.33 | 18.33 | 60.00 |
| 7 | BPH | IR22 | 30 | 25 | 2 | 5 | 2.00 | 15.40 | 100.00 |
| 7 | BPH | IR22 | 30 | 25 | 3 | 3 | 2.00 | 14.00 | 33.33 |
| 7 | BPH | IR22 | 30 | 25 | 4 | 3 | 1.00 | 0.00 | 33.33 |
| 7 | BPH | IR22 | 30 | 30 | 1 | 5 | 1.00 | 5.00 | 40.00 |
| 7 | BPH | IR22 | 30 | 30 | 2 | 5 | 3.00 | 10.00 | 60.00 |
| 7 | BPH | IR22 | 30 | 30 | 3 | 3 | 0.00 | 0.00 | 0.00 |
| 7 | BPH | IR22 | 30 | 30 | 4 | 3 | 0.00 | 0.00 | 0.00 |
| 7 | BPH | IR22 | 30 | 35 | 1 | 5 | 1.33 | 2.33 | 60.00 |
| 7 | BPH | IR22 | 30 | 35 | 2 | 3 | 0.00 | 0.00 | 0.00 |
| 7 | BPH | IR22 | 30 | 35 | 3 | 3 | 1.00 | 0.00 | 33.33 |
| 7 | BPH | IR22 | 30 | 35 | 4 | 3 | 1.00 | 0.00 | 33.33 |
| 7 | BPH | T65 | 20 | 15 | 1 | 5 | 1.00 | 3.00 | 60.00 |
| 7 | BPH | T65 | 20 | 15 | 2 | 3 | 1.00 | 0.67 | 100.00 |
| 7 | BPH | T65 | 20 | 15 | 3 | 3 | 1.00 | 0.00 | 33.33 |
| 7 | BPH | T65 | 20 | 15 | 4 | 3 | 1.00 | 1.33 | 100.00 |
| 7 | BPH | T65 | 20 | 20 | 1 | 5 | 4.00 | 11.00 | 60.00 |
| 7 | BPH | T65 | 20 | 20 | 2 | 5 | 4.67 | 20.33 | 60.00 |
| 7 | BPH | T65 | 20 | 20 | 3 | 3 | 4.00 | 13.00 | 66.67 |
| 7 | BPH | T65 | 20 | 20 | 4 | 3 | 4.00 | 24.00 | 33.33 |
| 7 | BPH | T65 | 20 | 25 | 1 | 5 | 9.20 | 30.60 | 100.00 |
| 7 | BPH | T65 | 20 | 25 | 2 | 5 | 1.00 | 2.00 | 20.00 |
| 7 | BPH | T65 | 20 | 25 | 3 | 3 | 8.00 | 17.00 | 33.33 |
| 7 | BPH | T65 | 20 | 25 | 4 | 3 | 0.00 | 0.00 | 0.00 |
| 7 | BPH | T65 | 20 | 30 | 1 | 5 | 13.50 | 27.00 | 40.00 |
| 7 | BPH | T65 | 20 | 30 | 2 | 5 | 10.67 | 39.33 | 60.00 |
| 7 | BPH | T65 | 20 | 30 | 3 | 3 | 3.00 | 6.00 | 33.33 |
| 7 | BPH | T65 | 20 | 30 | 4 | 3 | 0.00 | 0.00 | 0.00 |
| 7 | BPH | T65 | 20 | 35 | 1 | 5 | 0.00 | 0.00 | 0.00 |
| 7 | BPH | T65 | 20 | 35 | 2 | 3 | 1.00 | 0.00 | 33.33 |
| 7 | BPH | T65 | 20 | 35 | 3 | 3 | 0.00 | 0.00 | 0.00 |
| 7 | BPH | T65 | 20 | 35 | 4 | 3 | 0.00 | 0.00 | 0.00 |
| 7 | BPH | T65 | 30 | 15 | 1 | 5 | 1.60 | 7.00 | 100.00 |
| 7 | BPH | T65 | 30 | 15 | 2 | 3 | 1.00 | 0.00 | 33.33 |
| 7 | BPH | T65 | 30 | 15 | 3 | 3 | 2.00 | 4.50 | 66.67 |
| 7 | BPH | T65 | 30 | 15 | 4 | 3 | 2.00 | 13.50 | 66.67 |
| 7 | BPH | T65 | 30 | 20 | 1 | 5 | 3.00 | 8.60 | 100.00 |
| 7 | BPH | T65 | 30 | 20 | 2 | 5 | 2.80 | 18.60 | 100.00 |
| 7 | BPH | T65 | 30 | 20 | 3 | 3 | 1.00 | 0.00 | 33.33 |
| 7 | BPH | T65 | 30 | 20 | 4 | 3 | 0.00 | 0.00 | 0.00 |
| 7 | BPH | T65 | 30 | 25 | 1 | 5 | 5.60 | 19.60 | 100.00 |
| 7 | BPH | T65 | 30 | 25 | 2 | 5 | 4.00 | 33.25 | 80.00 |
| 7 | BPH | T65 | 30 | 25 | 3 | 3 | 1.00 | 0.00 | 33.33 |
| 7 | BPH | T65 | 30 | 25 | 4 | 3 | 5.00 | 36.00 | 33.33 |
| 7 | BPH | T65 | 30 | 30 | 1 | 5 | 5.00 | 19.33 | 60.00 |
| 7 | BPH | T65 | 30 | 30 | 2 | 5 | 6.75 | 44.50 | 80.00 |
| 7 | BPH | T65 | 30 | 30 | 3 | 3 | 7.00 | 48.00 | 33.33 |
| 7 | BPH | T65 | 30 | 30 | 4 | 3 | 1.00 | 16.00 | 33.33 |
| 7 | BPH | T65 | 30 | 35 | 1 | 5 | 1.00 | 0.00 | 40.00 |
| 7 | BPH | T65 | 30 | 35 | 2 | 3 | 1.00 | 1.00 | 33.33 |
| 7 | BPH | T65 | 30 | 35 | 3 | 3 | 0.00 | 0.00 | 0.00 |
| 7 | BPH | T65 | 30 | 35 | 4 | 3 | 1.00 | 0.00 | 33.33 |
| 7 | WBPH | IR22 | 20 | 15 | 1 | 5 | 0.00 | 0.00 | 60.00 |
| 7 | WBPH | IR22 | 20 | 15 | 2 | 3 | 1.00 | 0.00 | 33.33 |
| 7 | WBPH | IR22 | 20 | 15 | 3 | 3 | 1.00 | 0.00 | 33.33 |
| 7 | WBPH | IR22 | 20 | 15 | 4 | 3 | 1.00 | 2.67 | 100.00 |
| 7 | WBPH | IR22 | 20 | 20 | 1 | 5 | 0.00 | 0.00 | 20.00 |
| 7 | WBPH | IR22 | 20 | 20 | 2 | 5 | 0.00 | 0.00 | 20.00 |
| 7 | WBPH | IR22 | 20 | 20 | 3 | 3 | 0.00 | 0.00 | 0.00 |
| 7 | WBPH | IR22 | 20 | 20 | 4 | 3 | 1.33 | 7.33 | 100.00 |
| 7 | WBPH | IR22 | 20 | 25 | 1 | 5 | 0.00 | 0.00 | 20.00 |
| 7 | WBPH | IR22 | 20 | 25 | 2 | 5 | 0.00 | 0.00 | 20.00 |
| 7 | WBPH | IR22 | 20 | 25 | 3 | 3 | 0.00 | 0.00 | 0.00 |
| 7 | WBPH | IR22 | 20 | 25 | 4 | 3 | 3.00 | 26.00 | 33.33 |
| 7 | WBPH | IR22 | 20 | 30 | 1 | 5 | 0.00 | 0.00 | 0.00 |
| 7 | WBPH | IR22 | 20 | 30 | 2 | 5 | 0.00 | 0.00 | 0.00 |
| 7 | WBPH | IR22 | 20 | 30 | 3 | 3 | 1.00 | 0.00 | 33.33 |
| 7 | WBPH | IR22 | 20 | 30 | 4 | 3 | 0.00 | 0.00 | 0.00 |
| 7 | WBPH | IR22 | 20 | 35 | 1 | 5 | 0.00 | 0.00 | 0.00 |
| 7 | WBPH | IR22 | 20 | 35 | 2 | 3 | 0.00 | 0.00 | 0.00 |
| 7 | WBPH | IR22 | 20 | 35 | 3 | 3 | 1.00 | 0.00 | 33.33 |
| 7 | WBPH | IR22 | 20 | 35 | 4 | 3 | 0.00 | 0.00 | 0.00 |
| 7 | WBPH | IR22 | 30 | 15 | 1 | 1 | 0.00 | 0.00 | 100.00 |
| 7 | WBPH | IR22 | 30 | 15 | 2 | 1 | 0.00 | 0.00 | 100.00 |
| 7 | WBPH | IR22 | 30 | 15 | 3 | 1 | 0.00 | 0.00 | 80.00 |
| 7 | WBPH | IR22 | 30 | 15 | 4 | 1 | 0.00 | 0.00 | 80.00 |
| 7 | WBPH | IR22 | 30 | 20 | 1 | 5 | 0.00 | 0.00 | 40.00 |
| 7 | WBPH | IR22 | 30 | 20 | 2 | 5 | 0.00 | 0.00 | 0.00 |
| 7 | WBPH | IR22 | 30 | 20 | 3 | 3 | 1.00 | 0.00 | 66.67 |
| 7 | WBPH | IR22 | 30 | 20 | 4 | 3 | 1.00 | 8.67 | 100.00 |
| 7 | WBPH | IR22 | 30 | 25 | 1 | 5 | 0.00 | 0.00 | 0.00 |
| 7 | WBPH | IR22 | 30 | 25 | 2 | 5 | 0.00 | 0.00 | 0.00 |
| 7 | WBPH | IR22 | 30 | 25 | 3 | 5 | 0.00 | 0.00 | 0.00 |
| 7 | WBPH | IR22 | 30 | 25 | 4 | 5 | 0.00 | 0.00 | 0.00 |
| 7 | WBPH | IR22 | 30 | 30 | 1 | 5 | 2.00 | 12.00 | 20.00 |
| 7 | WBPH | IR22 | 30 | 30 | 2 | 5 | 0.00 | 0.00 | 0.00 |
| 7 | WBPH | IR22 | 30 | 30 | 3 | 3 | 0.00 | 0.00 | 0.00 |
| 7 | WBPH | IR22 | 30 | 30 | 4 | 3 | 0.00 | 0.00 | 0.00 |
| 7 | WBPH | IR22 | 30 | 35 | 1 | 5 | 0.00 | 0.00 | 0.00 |
| 7 | WBPH | IR22 | 30 | 35 | 2 | 3 | 0.00 | 0.00 | 0.00 |
| 7 | WBPH | IR22 | 30 | 35 | 3 | 3 | 0.00 | 0.00 | 0.00 |
| 7 | WBPH | IR22 | 30 | 35 | 4 | 3 | 0.00 | 0.00 | 0.00 |
| 7 | WBPH | T65 | 20 | 15 | 1 | 5 | 0.40 | 0.40 | 100.00 |
| 7 | WBPH | T65 | 20 | 15 | 2 | 3 | 1.33 | 3.00 | 100.00 |
| 7 | WBPH | T65 | 20 | 15 | 3 | 3 | 1.33 | 12.33 | 100.00 |
| 7 | WBPH | T65 | 20 | 15 | 4 | 3 | 1.00 | 15.50 | 66.67 |
| 7 | WBPH | T65 | 20 | 20 | 1 | 5 | 0.00 | 0.00 | 60.00 |
| 7 | WBPH | T65 | 20 | 20 | 2 | 5 | 1.00 | 6.00 | 20.00 |
| 7 | WBPH | T65 | 20 | 20 | 3 | 3 | 1.50 | 15.00 | 66.67 |
| 7 | WBPH | T65 | 20 | 20 | 4 | 3 | 1.00 | 7.00 | 66.67 |
| 7 | WBPH | T65 | 20 | 25 | 1 | 5 | 2.00 | 5.00 | 80.00 |
| 7 | WBPH | T65 | 20 | 25 | 2 | 5 | 1.00 | 7.00 | 20.00 |
| 7 | WBPH | T65 | 20 | 25 | 3 | 3 | 0.00 | 0.00 | 0.00 |
| 7 | WBPH | T65 | 20 | 25 | 4 | 3 | 4.00 | 28.33 | 100.00 |
| 7 | WBPH | T65 | 20 | 30 | 1 | 5 | 0.00 | 0.00 | 20.00 |
| 7 | WBPH | T65 | 20 | 30 | 2 | 5 | 0.00 | 0.00 | 0.00 |
| 7 | WBPH | T65 | 20 | 30 | 3 | 3 | 2.00 | 13.00 | 33.33 |
| 7 | WBPH | T65 | 20 | 30 | 4 | 3 | 1.00 | 20.00 | 33.33 |
| 7 | WBPH | T65 | 20 | 35 | 1 | 5 | 0.00 | 0.00 | 0.00 |
| 7 | WBPH | T65 | 20 | 35 | 2 | 3 | 1.00 | 4.00 | 33.33 |
| 7 | WBPH | T65 | 20 | 35 | 3 | 3 | 0.00 | 0.00 | 0.00 |
| 7 | WBPH | T65 | 20 | 35 | 4 | 3 | 1.00 | 0.00 | 66.67 |
| 7 | WBPH | T65 | 30 | 15 | 1 | 1 | 0.00 | 0.00 | 100.00 |
| 7 | WBPH | T65 | 30 | 15 | 2 | 1 | 0.00 | 0.00 | 100.00 |
| 7 | WBPH | T65 | 30 | 15 | 3 | 1 | 0.00 | 0.00 | 80.00 |
| 7 | WBPH | T65 | 30 | 15 | 4 | 1 | 0.00 | 0.00 | 100.00 |
| 7 | WBPH | T65 | 30 | 20 | 1 | 5 | 0.33 | 1.33 | 60.00 |
| 7 | WBPH | T65 | 30 | 20 | 2 | 5 | 0.00 | 0.00 | 0.00 |
| 7 | WBPH | T65 | 30 | 20 | 3 | 3 | 1.50 | 12.50 | 66.67 |
| 7 | WBPH | T65 | 30 | 20 | 4 | 3 | 2.00 | 17.00 | 33.33 |
| 7 | WBPH | T65 | 30 | 25 | 1 | 5 | 0.00 | 0.00 | 60.00 |
| 7 | WBPH | T65 | 30 | 25 | 2 | 5 | 0.00 | 0.00 | 0.00 |
| 7 | WBPH | T65 | 30 | 25 | 3 | 5 | 0.00 | 0.00 | 0.00 |
| 7 | WBPH | T65 | 30 | 25 | 4 | 5 | 0.00 | 0.00 | 0.00 |
| 7 | WBPH | T65 | 30 | 30 | 1 | 5 | 2.00 | 5.50 | 40.00 |
| 7 | WBPH | T65 | 30 | 30 | 2 | 5 | 0.00 | 0.00 | 0.00 |
| 7 | WBPH | T65 | 30 | 30 | 3 | 3 | 0.00 | 0.00 | 0.00 |
| 7 | WBPH | T65 | 30 | 30 | 4 | 3 | 0.00 | 0.00 | 0.00 |
| 7 | WBPH | T65 | 30 | 35 | 1 | 5 | 1.00 | 0.00 | 20.00 |
| 7 | WBPH | T65 | 30 | 35 | 2 | 3 | 0.00 | 0.00 | 0.00 |
| 7 | WBPH | T65 | 30 | 35 | 3 | 3 | 0.00 | 0.00 | 0.00 |
| 7 | WBPH | T65 | 30 | 35 | 4 | 3 | 0.00 | 0.00 | 0.00 |
| 8 | BPH | IR22 | 20 | 15 | 1 | 3 | 1.00 | 0.44 | 66.67 |
| 8 | BPH | IR22 | 20 | 15 | 2 | 3 | 1.00 | 0.00 | 66.67 |
| 8 | BPH | IR22 | 20 | 15 | 3 | 3 | 1.00 | 0.00 | 33.33 |
| 8 | BPH | IR22 | 20 | 15 | 4 | 3 | 1.00 | 1.33 | 100.00 |
| 8 | BPH | IR22 | 20 | 20 | 1 | 5 | 2.00 | 7.22 | 42.22 |
| 8 | BPH | IR22 | 20 | 20 | 2 | 5 | 2.00 | 6.67 | 60.00 |
| 8 | BPH | IR22 | 20 | 20 | 3 | 3 | 4.00 | 15.00 | 66.67 |
| 8 | BPH | IR22 | 20 | 20 | 4 | 3 | 0.00 | 0.00 | 0.00 |
| 8 | BPH | IR22 | 20 | 25 | 1 | 5 | 1.22 | 6.78 | 20.00 |
| 8 | BPH | IR22 | 20 | 25 | 2 | 5 | 3.67 | 20.33 | 60.00 |
| 8 | BPH | IR22 | 20 | 25 | 3 | 3 | 0.00 | 0.00 | 0.00 |
| 8 | BPH | IR22 | 20 | 25 | 4 | 3 | 0.00 | 0.00 | 0.00 |
| 8 | BPH | IR22 | 20 | 30 | 1 | 5 | 1.00 | 4.50 | 13.33 |
| 8 | BPH | IR22 | 20 | 30 | 2 | 5 | 3.00 | 13.50 | 40.00 |
| 8 | BPH | IR22 | 20 | 30 | 3 | 3 | 0.00 | 0.00 | 0.00 |
| 8 | BPH | IR22 | 20 | 30 | 4 | 3 | 0.00 | 0.00 | 0.00 |
| 8 | BPH | IR22 | 20 | 35 | 1 | 5 | 0.00 | 0.00 | 0.00 |
| 8 | BPH | IR22 | 20 | 35 | 2 | 3 | 0.00 | 0.00 | 0.00 |
| 8 | BPH | IR22 | 20 | 35 | 3 | 3 | 0.00 | 0.00 | 0.00 |
| 8 | BPH | IR22 | 20 | 35 | 4 | 3 | 0.00 | 0.00 | 0.00 |
| 8 | BPH | IR22 | 30 | 15 | 1 | 3 | 1.22 | 1.50 | 88.89 |
| 8 | BPH | IR22 | 30 | 15 | 2 | 3 | 1.67 | 4.00 | 100.00 |
| 8 | BPH | IR22 | 30 | 15 | 3 | 3 | 1.00 | 0.50 | 66.67 |
| 8 | BPH | IR22 | 30 | 15 | 4 | 3 | 1.00 | 0.00 | 100.00 |
| 8 | BPH | IR22 | 30 | 20 | 1 | 5 | 1.33 | 3.25 | 48.89 |
| 8 | BPH | IR22 | 30 | 20 | 2 | 5 | 2.00 | 5.75 | 80.00 |
| 8 | BPH | IR22 | 30 | 20 | 3 | 3 | 1.00 | 4.00 | 33.33 |
| 8 | BPH | IR22 | 30 | 20 | 4 | 3 | 1.00 | 0.00 | 33.33 |
| 8 | BPH | IR22 | 30 | 25 | 1 | 5 | 1.60 | 9.00 | 44.44 |
| 8 | BPH | IR22 | 30 | 25 | 2 | 5 | 2.80 | 19.00 | 100.00 |
| 8 | BPH | IR22 | 30 | 25 | 3 | 3 | 2.00 | 8.00 | 33.33 |
| 8 | BPH | IR22 | 30 | 25 | 4 | 3 | 0.00 | 0.00 | 0.00 |
| 8 | BPH | IR22 | 30 | 30 | 1 | 5 | 0.44 | 0.89 | 20.00 |
| 8 | BPH | IR22 | 30 | 30 | 2 | 5 | 1.33 | 2.67 | 60.00 |
| 8 | BPH | IR22 | 30 | 30 | 3 | 3 | 0.00 | 0.00 | 0.00 |
| 8 | BPH | IR22 | 30 | 30 | 4 | 3 | 0.00 | 0.00 | 0.00 |
| 8 | BPH | IR22 | 30 | 35 | 1 | 5 | 0.67 | 0.00 | 40.00 |
| 8 | BPH | IR22 | 30 | 35 | 2 | 3 | 0.00 | 0.00 | 0.00 |
| 8 | BPH | IR22 | 30 | 35 | 3 | 3 | 4.00 | 32.00 | 33.33 |
| 8 | BPH | IR22 | 30 | 35 | 4 | 3 | 1.00 | 0.00 | 33.33 |
| 8 | BPH | T65 | 20 | 15 | 1 | 3 | 1.11 | 4.44 | 77.78 |
| 8 | BPH | T65 | 20 | 15 | 2 | 3 | 1.33 | 5.33 | 100.00 |
| 8 | BPH | T65 | 20 | 15 | 3 | 3 | 1.00 | 5.00 | 33.33 |
| 8 | BPH | T65 | 20 | 15 | 4 | 3 | 1.00 | 3.00 | 100.00 |
| 8 | BPH | T65 | 20 | 20 | 1 | 5 | 2.72 | 14.39 | 53.33 |
| 8 | BPH | T65 | 20 | 20 | 2 | 5 | 1.67 | 13.67 | 60.00 |
| 8 | BPH | T65 | 20 | 20 | 3 | 3 | 3.50 | 14.50 | 66.67 |
| 8 | BPH | T65 | 20 | 20 | 4 | 3 | 3.00 | 15.00 | 33.33 |
| 8 | BPH | T65 | 20 | 25 | 1 | 5 | 2.33 | 5.33 | 11.11 |
| 8 | BPH | T65 | 20 | 25 | 2 | 5 | 0.00 | 0.00 | 0.00 |
| 8 | BPH | T65 | 20 | 25 | 3 | 3 | 7.00 | 16.00 | 33.33 |
| 8 | BPH | T65 | 20 | 25 | 4 | 3 | 0.00 | 0.00 | 0.00 |
| 8 | BPH | T65 | 20 | 30 | 1 | 5 | 2.89 | 9.78 | 31.11 |
| 8 | BPH | T65 | 20 | 30 | 2 | 5 | 3.67 | 14.33 | 60.00 |
| 8 | BPH | T65 | 20 | 30 | 3 | 3 | 5.00 | 15.00 | 33.33 |
| 8 | BPH | T65 | 20 | 30 | 4 | 3 | 0.00 | 0.00 | 0.00 |
| 8 | BPH | T65 | 20 | 35 | 1 | 5 | 0.00 | 0.00 | 0.00 |
| 8 | BPH | T65 | 20 | 35 | 2 | 3 | 1.00 | 0.00 | 33.33 |
| 8 | BPH | T65 | 20 | 35 | 3 | 3 | 0.00 | 0.00 | 0.00 |
| 8 | BPH | T65 | 20 | 35 | 4 | 3 | 0.00 | 0.00 | 0.00 |
| 8 | BPH | T65 | 30 | 15 | 1 | 3 | 1.67 | 3.00 | 55.56 |
| 8 | BPH | T65 | 30 | 15 | 2 | 3 | 1.00 | 0.00 | 33.33 |
| 8 | BPH | T65 | 30 | 15 | 3 | 3 | 3.00 | 9.00 | 66.67 |
| 8 | BPH | T65 | 30 | 15 | 4 | 3 | 1.00 | 0.00 | 66.67 |
| 8 | BPH | T65 | 30 | 20 | 1 | 5 | 1.00 | 6.50 | 26.67 |
| 8 | BPH | T65 | 30 | 20 | 2 | 5 | 3.00 | 19.50 | 80.00 |
| 8 | BPH | T65 | 30 | 20 | 3 | 3 | 0.00 | 0.00 | 0.00 |
| 8 | BPH | T65 | 30 | 20 | 4 | 3 | 0.00 | 0.00 | 0.00 |
| 8 | BPH | T65 | 30 | 25 | 1 | 5 | 2.92 | 20.00 | 37.78 |
| 8 | BPH | T65 | 30 | 25 | 2 | 5 | 2.75 | 23.00 | 80.00 |
| 8 | BPH | T65 | 30 | 25 | 3 | 3 | 0.00 | 0.00 | 0.00 |
| 8 | BPH | T65 | 30 | 25 | 4 | 3 | 6.00 | 37.00 | 33.33 |
| 8 | BPH | T65 | 30 | 30 | 1 | 5 | 6.42 | 29.25 | 37.78 |
| 8 | BPH | T65 | 30 | 30 | 2 | 5 | 7.25 | 32.75 | 80.00 |
| 8 | BPH | T65 | 30 | 30 | 3 | 3 | 12.00 | 55.00 | 33.33 |
| 8 | BPH | T65 | 30 | 30 | 4 | 3 | 0.00 | 0.00 | 0.00 |
| 8 | BPH | T65 | 30 | 35 | 1 | 5 | 1.00 | 0.00 | 40.00 |
| 8 | BPH | T65 | 30 | 35 | 2 | 3 | 1.00 | 0.00 | 33.33 |
| 8 | BPH | T65 | 30 | 35 | 3 | 3 | 0.00 | 0.00 | 0.00 |
| 8 | BPH | T65 | 30 | 35 | 4 | 3 | 1.00 | 0.00 | 33.33 |
| 8 | WBPH | IR22 | 20 | 15 | 1 | 3 | 1.00 | 2.33 | 55.56 |
| 8 | WBPH | IR22 | 20 | 15 | 2 | 3 | 1.00 | 7.00 | 33.33 |
| 8 | WBPH | IR22 | 20 | 15 | 3 | 3 | 1.00 | 0.00 | 33.33 |
| 8 | WBPH | IR22 | 20 | 15 | 4 | 3 | 1.00 | 0.00 | 100.00 |
| 8 | WBPH | IR22 | 20 | 20 | 1 | 5 | 0.67 | 5.33 | 33.33 |
| 8 | WBPH | IR22 | 20 | 20 | 2 | 5 | 0.00 | 0.00 | 0.00 |
| 8 | WBPH | IR22 | 20 | 20 | 3 | 3 | 0.00 | 0.00 | 0.00 |
| 8 | WBPH | IR22 | 20 | 20 | 4 | 3 | 2.00 | 16.00 | 100.00 |
| 8 | WBPH | IR22 | 20 | 25 | 1 | 5 | 0.33 | 1.33 | 11.11 |
| 8 | WBPH | IR22 | 20 | 25 | 2 | 5 | 0.00 | 0.00 | 0.00 |
| 8 | WBPH | IR22 | 20 | 25 | 3 | 3 | 0.00 | 0.00 | 0.00 |
| 8 | WBPH | IR22 | 20 | 25 | 4 | 3 | 1.00 | 4.00 | 33.33 |
| 8 | WBPH | IR22 | 20 | 30 | 1 | 5 | 0.00 | 0.00 | 0.00 |
| 8 | WBPH | IR22 | 20 | 30 | 2 | 5 | 0.00 | 0.00 | 0.00 |
| 8 | WBPH | IR22 | 20 | 30 | 3 | 3 | 0.00 | 0.00 | 0.00 |
| 8 | WBPH | IR22 | 20 | 30 | 4 | 3 | 0.00 | 0.00 | 0.00 |
| 8 | WBPH | IR22 | 20 | 35 | 1 | 5 | 0.00 | 0.00 | 0.00 |
| 8 | WBPH | IR22 | 20 | 35 | 2 | 3 | 0.00 | 0.00 | 0.00 |
| 8 | WBPH | IR22 | 20 | 35 | 3 | 3 | 1.00 | 0.00 | 33.33 |
| 8 | WBPH | IR22 | 20 | 35 | 4 | 3 | 0.00 | 0.00 | 0.00 |
| 8 | WBPH | IR22 | 30 | 15 | 1 | 1 | 0.00 | 0.00 | 67.00 |
| 8 | WBPH | IR22 | 30 | 15 | 2 | 1 | 0.00 | 0.00 | 100.00 |
| 8 | WBPH | IR22 | 30 | 15 | 3 | 1 | 0.00 | 0.00 | 80.00 |
| 8 | WBPH | IR22 | 30 | 15 | 4 | 1 | 0.00 | 0.00 | 80.00 |
| 8 | WBPH | IR22 | 30 | 20 | 1 | 5 | 1.00 | 6.67 | 22.22 |
| 8 | WBPH | IR22 | 30 | 20 | 2 | 5 | 0.00 | 0.00 | 0.00 |
| 8 | WBPH | IR22 | 30 | 20 | 3 | 3 | 1.00 | 0.00 | 33.33 |
| 8 | WBPH | IR22 | 30 | 20 | 4 | 3 | 2.00 | 20.00 | 33.33 |
| 8 | WBPH | IR22 | 30 | 25 | 1 | 5 | 0.00 | 0.00 | 0.00 |
| 8 | WBPH | IR22 | 30 | 25 | 2 | 5 | 0.00 | 0.00 | 0.00 |
| 8 | WBPH | IR22 | 30 | 25 | 3 | 5 | 0.00 | 0.00 | 0.00 |
| 8 | WBPH | IR22 | 30 | 25 | 4 | 5 | 0.00 | 0.00 | 0.00 |
| 8 | WBPH | IR22 | 30 | 30 | 1 | 5 | 0.00 | 0.00 | 0.00 |
| 8 | WBPH | IR22 | 30 | 30 | 2 | 5 | 0.00 | 0.00 | 0.00 |
| 8 | WBPH | IR22 | 30 | 30 | 3 | 3 | 0.00 | 0.00 | 0.00 |
| 8 | WBPH | IR22 | 30 | 30 | 4 | 3 | 0.00 | 0.00 | 0.00 |
| 8 | WBPH | IR22 | 30 | 35 | 1 | 5 | 0.00 | 0.00 | 0.00 |
| 8 | WBPH | IR22 | 30 | 35 | 2 | 3 | 0.00 | 0.00 | 0.00 |
| 8 | WBPH | IR22 | 30 | 35 | 3 | 3 | 0.00 | 0.00 | 0.00 |
| 8 | WBPH | IR22 | 30 | 35 | 4 | 3 | 0.00 | 0.00 | 0.00 |
| 8 | WBPH | T65 | 20 | 15 | 1 | 3 | 1.17 | 3.83 | 66.67 |
| 8 | WBPH | T65 | 20 | 15 | 2 | 3 | 1.00 | 0.00 | 66.67 |
| 8 | WBPH | T65 | 20 | 15 | 3 | 3 | 1.50 | 9.00 | 66.67 |
| 8 | WBPH | T65 | 20 | 15 | 4 | 3 | 1.00 | 2.50 | 66.67 |
| 8 | WBPH | T65 | 20 | 20 | 1 | 5 | 1.00 | 5.83 | 46.67 |
| 8 | WBPH | T65 | 20 | 20 | 2 | 5 | 0.00 | 0.00 | 40.00 |
| 8 | WBPH | T65 | 20 | 20 | 3 | 3 | 1.00 | 0.50 | 66.67 |
| 8 | WBPH | T65 | 20 | 20 | 4 | 3 | 2.00 | 17.00 | 33.33 |
| 8 | WBPH | T65 | 20 | 25 | 1 | 5 | 1.67 | 5.67 | 22.22 |
| 8 | WBPH | T65 | 20 | 25 | 2 | 5 | 0.00 | 0.00 | 0.00 |
| 8 | WBPH | T65 | 20 | 25 | 3 | 3 | 0.00 | 0.00 | 0.00 |
| 8 | WBPH | T65 | 20 | 25 | 4 | 3 | 5.00 | 17.00 | 66.67 |
| 8 | WBPH | T65 | 20 | 30 | 1 | 5 | 0.33 | 0.00 | 11.11 |
| 8 | WBPH | T65 | 20 | 30 | 2 | 5 | 0.00 | 0.00 | 0.00 |
| 8 | WBPH | T65 | 20 | 30 | 3 | 3 | 0.00 | 0.00 | 0.00 |
| 8 | WBPH | T65 | 20 | 30 | 4 | 3 | 1.00 | 0.00 | 33.33 |
| 8 | WBPH | T65 | 20 | 35 | 1 | 5 | 0.00 | 0.00 | 0.00 |
| 8 | WBPH | T65 | 20 | 35 | 2 | 3 | 2.00 | 11.00 | 33.33 |
| 8 | WBPH | T65 | 20 | 35 | 3 | 3 | 0.00 | 0.00 | 0.00 |
| 8 | WBPH | T65 | 20 | 35 | 4 | 3 | 2.50 | 8.00 | 66.67 |
| 8 | WBPH | T65 | 30 | 15 | 1 | 1 | 0.00 | 0.00 | 67.00 |
| 8 | WBPH | T65 | 30 | 15 | 2 | 1 | 0.00 | 0.00 | 100.00 |
| 8 | WBPH | T65 | 30 | 15 | 3 | 1 | 0.00 | 0.00 | 80.00 |
| 8 | WBPH | T65 | 30 | 15 | 4 | 1 | 0.00 | 0.00 | 60.00 |
| 8 | WBPH | T65 | 30 | 20 | 1 | 5 | 0.83 | 5.17 | 33.33 |
| 8 | WBPH | T65 | 30 | 20 | 2 | 5 | 0.00 | 0.00 | 0.00 |
| 8 | WBPH | T65 | 30 | 20 | 3 | 3 | 1.50 | 8.50 | 66.67 |
| 8 | WBPH | T65 | 30 | 20 | 4 | 3 | 1.00 | 7.00 | 33.33 |
| 8 | WBPH | T65 | 30 | 25 | 1 | 5 | 0.00 | 0.00 | 0.00 |
| 8 | WBPH | T65 | 30 | 25 | 2 | 5 | 0.00 | 0.00 | 0.00 |
| 8 | WBPH | T65 | 30 | 25 | 3 | 5 | 0.00 | 0.00 | 0.00 |
| 8 | WBPH | T65 | 30 | 25 | 4 | 5 | 0.00 | 0.00 | 0.00 |
| 8 | WBPH | T65 | 30 | 30 | 1 | 5 | 0.00 | 0.00 | 0.00 |
| 8 | WBPH | T65 | 30 | 30 | 2 | 5 | 0.00 | 0.00 | 0.00 |
| 8 | WBPH | T65 | 30 | 30 | 3 | 3 | 0.00 | 0.00 | 0.00 |
| 8 | WBPH | T65 | 30 | 30 | 4 | 3 | 0.00 | 0.00 | 0.00 |
| 8 | WBPH | T65 | 30 | 35 | 1 | 5 | 1.00 | 0.00 | 20.00 |
| 8 | WBPH | T65 | 30 | 35 | 2 | 3 | 0.00 | 0.00 | 0.00 |
| 8 | WBPH | T65 | 30 | 35 | 3 | 3 | 0.00 | 0.00 | 0.00 |
| 8 | WBPH | T65 | 30 | 35 | 4 | 3 | 0.00 | 0.00 | 0.00 |
| 9 | BPH | IR22 | 20 | 15 | 1 | 3 | 1.56 | 10.67 | 55.56 |
| 9 | BPH | IR22 | 20 | 15 | 2 | 3 | 2.00 | 26.00 | 33.33 |
| 9 | BPH | IR22 | 20 | 15 | 3 | 3 | 1.00 | 0.00 | 33.33 |
| 9 | BPH | IR22 | 20 | 15 | 4 | 3 | 1.67 | 6.00 | 100.00 |
| 9 | BPH | IR22 | 20 | 20 | 1 | 5 | 2.00 | 8.00 | 42.22 |
| 9 | BPH | IR22 | 20 | 20 | 2 | 5 | 3.00 | 13.00 | 60.00 |
| 9 | BPH | IR22 | 20 | 20 | 3 | 3 | 3.00 | 11.00 | 66.67 |
| 9 | BPH | IR22 | 20 | 20 | 4 | 3 | 0.00 | 0.00 | 0.00 |
| 9 | BPH | IR22 | 20 | 25 | 1 | 5 | 2.17 | 10.67 | 13.33 |
| 9 | BPH | IR22 | 20 | 25 | 2 | 5 | 6.50 | 32.00 | 40.00 |
| 9 | BPH | IR22 | 20 | 25 | 3 | 3 | 0.00 | 0.00 | 0.00 |
| 9 | BPH | IR22 | 20 | 25 | 4 | 3 | 0.00 | 0.00 | 0.00 |
| 9 | BPH | IR22 | 20 | 30 | 1 | 5 | 2.00 | 9.00 | 13.33 |
| 9 | BPH | IR22 | 20 | 30 | 2 | 5 | 6.00 | 27.00 | 40.00 |
| 9 | BPH | IR22 | 20 | 30 | 3 | 3 | 0.00 | 0.00 | 0.00 |
| 9 | BPH | IR22 | 20 | 30 | 4 | 3 | 0.00 | 0.00 | 0.00 |
| 9 | BPH | IR22 | 20 | 35 | 1 | 5 | 0.00 | 0.00 | 0.00 |
| 9 | BPH | IR22 | 20 | 35 | 2 | 3 | 0.00 | 0.00 | 0.00 |
| 9 | BPH | IR22 | 20 | 35 | 3 | 3 | 0.00 | 0.00 | 0.00 |
| 9 | BPH | IR22 | 20 | 35 | 4 | 3 | 0.00 | 0.00 | 0.00 |
| 9 | BPH | IR22 | 30 | 15 | 1 | 3 | 1.83 | 3.67 | 88.89 |
| 9 | BPH | IR22 | 30 | 15 | 2 | 3 | 1.00 | 0.00 | 100.00 |
| 9 | BPH | IR22 | 30 | 15 | 3 | 3 | 3.50 | 11.00 | 66.67 |
| 9 | BPH | IR22 | 30 | 15 | 4 | 3 | 1.00 | 0.00 | 100.00 |
| 9 | BPH | IR22 | 30 | 20 | 1 | 5 | 2.42 | 12.67 | 48.89 |
| 9 | BPH | IR22 | 30 | 20 | 2 | 5 | 2.25 | 11.00 | 80.00 |
| 9 | BPH | IR22 | 30 | 20 | 3 | 3 | 2.00 | 8.00 | 33.33 |
| 9 | BPH | IR22 | 30 | 20 | 4 | 3 | 3.00 | 19.00 | 33.33 |
| 9 | BPH | IR22 | 30 | 25 | 1 | 5 | 1.27 | 5.53 | 44.44 |
| 9 | BPH | IR22 | 30 | 25 | 2 | 5 | 2.80 | 16.60 | 100.00 |
| 9 | BPH | IR22 | 30 | 25 | 3 | 3 | 1.00 | 0.00 | 33.33 |
| 9 | BPH | IR22 | 30 | 25 | 4 | 3 | 0.00 | 0.00 | 0.00 |
| 9 | BPH | IR22 | 30 | 30 | 1 | 5 | 0.33 | 0.00 | 13.33 |
| 9 | BPH | IR22 | 30 | 30 | 2 | 5 | 1.00 | 0.00 | 40.00 |
| 9 | BPH | IR22 | 30 | 30 | 3 | 3 | 0.00 | 0.00 | 0.00 |
| 9 | BPH | IR22 | 30 | 30 | 4 | 3 | 0.00 | 0.00 | 0.00 |
| 9 | BPH | IR22 | 30 | 35 | 1 | 5 | 1.00 | 0.00 | 40.00 |
| 9 | BPH | IR22 | 30 | 35 | 2 | 3 | 0.00 | 0.00 | 0.00 |
| 9 | BPH | IR22 | 30 | 35 | 3 | 3 | 0.00 | 0.00 | 0.00 |
| 9 | BPH | IR22 | 30 | 35 | 4 | 3 | 0.00 | 0.00 | 0.00 |
| 9 | BPH | T65 | 20 | 15 | 1 | 3 | 0.67 | 0.00 | 44.44 |
| 9 | BPH | T65 | 20 | 15 | 2 | 3 | 1.00 | 0.00 | 100.00 |
| 9 | BPH | T65 | 20 | 15 | 3 | 3 | 0.00 | 0.00 | 0.00 |
| 9 | BPH | T65 | 20 | 15 | 4 | 3 | 1.00 | 0.00 | 33.33 |
| 9 | BPH | T65 | 20 | 20 | 1 | 5 | 6.72 | 25.44 | 53.33 |
| 9 | BPH | T65 | 20 | 20 | 2 | 5 | 7.67 | 24.33 | 60.00 |
| 9 | BPH | T65 | 20 | 20 | 3 | 3 | 6.50 | 17.00 | 66.67 |
| 9 | BPH | T65 | 20 | 20 | 4 | 3 | 6.00 | 35.00 | 33.33 |
| 9 | BPH | T65 | 20 | 25 | 1 | 5 | 0.00 | 0.00 | 0.00 |
| 9 | BPH | T65 | 20 | 25 | 2 | 5 | 0.00 | 0.00 | 0.00 |
| 9 | BPH | T65 | 20 | 25 | 3 | 3 | 3.00 | 7.00 | 33.33 |
| 9 | BPH | T65 | 20 | 25 | 4 | 3 | 0.00 | 0.00 | 0.00 |
| 9 | BPH | T65 | 20 | 30 | 1 | 5 | 4.17 | 11.33 | 24.44 |
| 9 | BPH | T65 | 20 | 30 | 2 | 5 | 11.50 | 29.00 | 40.00 |
| 9 | BPH | T65 | 20 | 30 | 3 | 3 | 1.00 | 5.00 | 33.33 |
| 9 | BPH | T65 | 20 | 30 | 4 | 3 | 0.00 | 0.00 | 0.00 |
| 9 | BPH | T65 | 20 | 35 | 1 | 5 | 0.00 | 0.00 | 0.00 |
| 9 | BPH | T65 | 20 | 35 | 2 | 3 | 0.00 | 0.00 | 0.00 |
| 9 | BPH | T65 | 20 | 35 | 3 | 3 | 0.00 | 0.00 | 0.00 |
| 9 | BPH | T65 | 20 | 35 | 4 | 3 | 0.00 | 0.00 | 0.00 |
| 9 | BPH | T65 | 30 | 15 | 1 | 3 | 1.67 | 3.17 | 33.33 |
| 9 | BPH | T65 | 30 | 15 | 2 | 3 | 0.00 | 0.00 | 0.00 |
| 9 | BPH | T65 | 30 | 15 | 3 | 3 | 3.00 | 6.50 | 66.67 |
| 9 | BPH | T65 | 30 | 15 | 4 | 3 | 2.00 | 3.00 | 33.33 |
| 9 | BPH | T65 | 30 | 20 | 1 | 5 | 1.25 | 6.17 | 26.67 |
| 9 | BPH | T65 | 30 | 20 | 2 | 5 | 3.75 | 18.50 | 80.00 |
| 9 | BPH | T65 | 30 | 20 | 3 | 3 | 0.00 | 0.00 | 0.00 |
| 9 | BPH | T65 | 30 | 20 | 4 | 3 | 0.00 | 0.00 | 0.00 |
| 9 | BPH | T65 | 30 | 25 | 1 | 5 | 1.50 | 11.75 | 37.78 |
| 9 | BPH | T65 | 30 | 25 | 2 | 5 | 3.50 | 23.25 | 80.00 |
| 9 | BPH | T65 | 30 | 25 | 3 | 3 | 0.00 | 0.00 | 0.00 |
| 9 | BPH | T65 | 30 | 25 | 4 | 3 | 1.00 | 12.00 | 33.33 |
| 9 | BPH | T65 | 30 | 30 | 1 | 5 | 5.58 | 24.75 | 37.78 |
| 9 | BPH | T65 | 30 | 30 | 2 | 5 | 4.75 | 28.25 | 80.00 |
| 9 | BPH | T65 | 30 | 30 | 3 | 3 | 12.00 | 46.00 | 33.33 |
| 9 | BPH | T65 | 30 | 30 | 4 | 3 | 0.00 | 0.00 | 0.00 |
| 9 | BPH | T65 | 30 | 35 | 1 | 5 | 1.00 | 0.00 | 20.00 |
| 9 | BPH | T65 | 30 | 35 | 2 | 3 | 0.00 | 0.00 | 0.00 |
| 9 | BPH | T65 | 30 | 35 | 3 | 3 | 0.00 | 0.00 | 0.00 |
| 9 | BPH | T65 | 30 | 35 | 4 | 3 | 0.00 | 0.00 | 0.00 |
| 9 | WBPH | IR22 | 20 | 15 | 1 | 3 | 1.00 | 0.00 | 44.44 |
| 9 | WBPH | IR22 | 20 | 15 | 2 | 3 | 1.00 | 0.00 | 33.33 |
| 9 | WBPH | IR22 | 20 | 15 | 3 | 3 | 1.00 | 0.00 | 33.33 |
| 9 | WBPH | IR22 | 20 | 15 | 4 | 3 | 1.00 | 0.00 | 66.67 |
| 9 | WBPH | IR22 | 20 | 20 | 1 | 5 | 0.33 | 2.11 | 33.33 |
| 9 | WBPH | IR22 | 20 | 20 | 2 | 5 | 0.00 | 0.00 | 0.00 |
| 9 | WBPH | IR22 | 20 | 20 | 3 | 3 | 0.00 | 0.00 | 0.00 |
| 9 | WBPH | IR22 | 20 | 20 | 4 | 3 | 1.00 | 6.33 | 100.00 |
| 9 | WBPH | IR22 | 20 | 25 | 1 | 5 | 1.00 | 7.00 | 11.11 |
| 9 | WBPH | IR22 | 20 | 25 | 2 | 5 | 0.00 | 0.00 | 0.00 |
| 9 | WBPH | IR22 | 20 | 25 | 3 | 3 | 0.00 | 0.00 | 0.00 |
| 9 | WBPH | IR22 | 20 | 25 | 4 | 3 | 3.00 | 21.00 | 33.33 |
| 9 | WBPH | IR22 | 20 | 30 | 1 | 5 | 0.00 | 0.00 | 0.00 |
| 9 | WBPH | IR22 | 20 | 30 | 2 | 5 | 0.00 | 0.00 | 0.00 |
| 9 | WBPH | IR22 | 20 | 30 | 3 | 3 | 0.00 | 0.00 | 0.00 |
| 9 | WBPH | IR22 | 20 | 30 | 4 | 3 | 0.00 | 0.00 | 0.00 |
| 9 | WBPH | IR22 | 20 | 35 | 1 | 5 | 0.00 | 0.00 | 0.00 |
| 9 | WBPH | IR22 | 20 | 35 | 2 | 3 | 0.00 | 0.00 | 0.00 |
| 9 | WBPH | IR22 | 20 | 35 | 3 | 3 | 0.00 | 0.00 | 0.00 |
| 9 | WBPH | IR22 | 20 | 35 | 4 | 3 | 0.00 | 0.00 | 0.00 |
| 9 | WBPH | IR22 | 30 | 15 | 1 | 1 | 0.00 | 0.00 | 67.00 |
| 9 | WBPH | IR22 | 30 | 15 | 2 | 1 | 0.00 | 0.00 | 100.00 |
| 9 | WBPH | IR22 | 30 | 15 | 3 | 1 | 0.00 | 0.00 | 80.00 |
| 9 | WBPH | IR22 | 30 | 15 | 4 | 1 | 0.00 | 0.00 | 60.00 |
| 9 | WBPH | IR22 | 30 | 20 | 1 | 5 | 1.33 | 3.67 | 22.22 |
| 9 | WBPH | IR22 | 30 | 20 | 2 | 5 | 0.00 | 0.00 | 0.00 |
| 9 | WBPH | IR22 | 30 | 20 | 3 | 3 | 1.00 | 0.00 | 33.33 |
| 9 | WBPH | IR22 | 30 | 20 | 4 | 3 | 3.00 | 11.00 | 33.33 |
| 9 | WBPH | IR22 | 30 | 25 | 1 | 5 | 0.00 | 0.00 | 0.00 |
| 9 | WBPH | IR22 | 30 | 25 | 2 | 5 | 0.00 | 0.00 | 0.00 |
| 9 | WBPH | IR22 | 30 | 25 | 3 | 5 | 0.00 | 0.00 | 0.00 |
| 9 | WBPH | IR22 | 30 | 25 | 4 | 5 | 0.00 | 0.00 | 0.00 |
| 9 | WBPH | IR22 | 30 | 30 | 1 | 5 | 0.00 | 0.00 | 0.00 |
| 9 | WBPH | IR22 | 30 | 30 | 2 | 5 | 0.00 | 0.00 | 0.00 |
| 9 | WBPH | IR22 | 30 | 30 | 3 | 3 | 0.00 | 0.00 | 0.00 |
| 9 | WBPH | IR22 | 30 | 30 | 4 | 3 | 0.00 | 0.00 | 0.00 |
| 9 | WBPH | IR22 | 30 | 35 | 1 | 5 | 0.00 | 0.00 | 0.00 |
| 9 | WBPH | IR22 | 30 | 35 | 2 | 3 | 0.00 | 0.00 | 0.00 |
| 9 | WBPH | IR22 | 30 | 35 | 3 | 3 | 0.00 | 0.00 | 0.00 |
| 9 | WBPH | IR22 | 30 | 35 | 4 | 3 | 0.00 | 0.00 | 0.00 |
| 9 | WBPH | T65 | 20 | 15 | 1 | 3 | 1.00 | 3.50 | 66.67 |
| 9 | WBPH | T65 | 20 | 15 | 2 | 3 | 1.00 | 2.50 | 66.67 |
| 9 | WBPH | T65 | 20 | 15 | 3 | 3 | 1.00 | 4.50 | 66.67 |
| 9 | WBPH | T65 | 20 | 15 | 4 | 3 | 1.00 | 3.50 | 66.67 |
| 9 | WBPH | T65 | 20 | 20 | 1 | 5 | 1.00 | 5.67 | 22.22 |
| 9 | WBPH | T65 | 20 | 20 | 2 | 5 | 0.00 | 0.00 | 0.00 |
| 9 | WBPH | T65 | 20 | 20 | 3 | 3 | 2.00 | 6.00 | 33.33 |
| 9 | WBPH | T65 | 20 | 20 | 4 | 3 | 1.00 | 11.00 | 33.33 |
| 9 | WBPH | T65 | 20 | 25 | 1 | 5 | 0.33 | 0.67 | 22.22 |
| 9 | WBPH | T65 | 20 | 25 | 2 | 5 | 0.00 | 0.00 | 0.00 |
| 9 | WBPH | T65 | 20 | 25 | 3 | 3 | 0.00 | 0.00 | 0.00 |
| 9 | WBPH | T65 | 20 | 25 | 4 | 3 | 1.00 | 2.00 | 66.67 |
| 9 | WBPH | T65 | 20 | 30 | 1 | 5 | 1.00 | 9.00 | 11.11 |
| 9 | WBPH | T65 | 20 | 30 | 2 | 5 | 0.00 | 0.00 | 0.00 |
| 9 | WBPH | T65 | 20 | 30 | 3 | 3 | 0.00 | 0.00 | 0.00 |
| 9 | WBPH | T65 | 20 | 30 | 4 | 3 | 3.00 | 27.00 | 33.33 |
| 9 | WBPH | T65 | 20 | 35 | 1 | 5 | 0.00 | 0.00 | 0.00 |
| 9 | WBPH | T65 | 20 | 35 | 2 | 3 | 0.00 | 0.00 | 0.00 |
| 9 | WBPH | T65 | 20 | 35 | 3 | 3 | 0.00 | 0.00 | 0.00 |
| 9 | WBPH | T65 | 20 | 35 | 4 | 3 | 1.00 | 2.00 | 33.33 |
| 9 | WBPH | T65 | 30 | 15 | 1 | 1 | 0.00 | 0.00 | 67.00 |
| 9 | WBPH | T65 | 30 | 15 | 2 | 1 | 0.00 | 0.00 | 60.00 |
| 9 | WBPH | T65 | 30 | 15 | 3 | 1 | 0.00 | 0.00 | 80.00 |
| 9 | WBPH | T65 | 30 | 15 | 4 | 1 | 0.00 | 0.00 | 60.00 |
| 9 | WBPH | T65 | 30 | 20 | 1 | 5 | 0.67 | 1.00 | 33.33 |
| 9 | WBPH | T65 | 30 | 20 | 2 | 5 | 0.00 | 0.00 | 0.00 |
| 9 | WBPH | T65 | 30 | 20 | 3 | 3 | 1.00 | 3.00 | 66.67 |
| 9 | WBPH | T65 | 30 | 20 | 4 | 3 | 1.00 | 0.00 | 33.33 |
| 9 | WBPH | T65 | 30 | 25 | 1 | 5 | 0.00 | 0.00 | 0.00 |
| 9 | WBPH | T65 | 30 | 25 | 2 | 5 | 0.00 | 0.00 | 0.00 |
| 9 | WBPH | T65 | 30 | 25 | 3 | 5 | 0.00 | 0.00 | 0.00 |
| 9 | WBPH | T65 | 30 | 25 | 4 | 5 | 0.00 | 0.00 | 0.00 |
| 9 | WBPH | T65 | 30 | 30 | 1 | 5 | 0.00 | 0.00 | 0.00 |
| 9 | WBPH | T65 | 30 | 30 | 2 | 5 | 0.00 | 0.00 | 0.00 |
| 9 | WBPH | T65 | 30 | 30 | 3 | 3 | 0.00 | 0.00 | 0.00 |
| 9 | WBPH | T65 | 30 | 30 | 4 | 3 | 0.00 | 0.00 | 0.00 |
| 9 | WBPH | T65 | 30 | 35 | 1 | 5 | 0.00 | 0.00 | 0.00 |
| 9 | WBPH | T65 | 30 | 35 | 2 | 3 | 0.00 | 0.00 | 0.00 |
| 9 | WBPH | T65 | 30 | 35 | 3 | 3 | 0.00 | 0.00 | 0.00 |
| 9 | WBPH | T65 | 30 | 35 | 4 | 3 | 0.00 | 0.00 | 0.00 |
| 10 | BPH | IR22 | 20 | 15 | 1 | 3 | 1.00 | 0.56 | 55.56 |
| 10 | BPH | IR22 | 20 | 15 | 2 | 3 | 1.00 | 0.00 | 33.33 |
| 10 | BPH | IR22 | 20 | 15 | 3 | 3 | 1.00 | 0.00 | 33.33 |
| 10 | BPH | IR22 | 20 | 15 | 4 | 3 | 1.00 | 1.67 | 100.00 |
| 10 | BPH | IR22 | 20 | 20 | 1 | 5 | 0.89 | 2.22 | 42.22 |
| 10 | BPH | IR22 | 20 | 20 | 2 | 5 | 1.67 | 6.67 | 60.00 |
| 10 | BPH | IR22 | 20 | 20 | 3 | 3 | 1.00 | 0.00 | 66.67 |
| 10 | BPH | IR22 | 20 | 20 | 4 | 3 | 0.00 | 0.00 | 0.00 |
| 10 | BPH | IR22 | 20 | 25 | 1 | 5 | 1.83 | 12.17 | 13.33 |
| 10 | BPH | IR22 | 20 | 25 | 2 | 5 | 5.50 | 36.50 | 40.00 |
| 10 | BPH | IR22 | 20 | 25 | 3 | 3 | 0.00 | 0.00 | 0.00 |
| 10 | BPH | IR22 | 20 | 25 | 4 | 3 | 0.00 | 0.00 | 0.00 |
| 10 | BPH | IR22 | 20 | 30 | 1 | 5 | 1.22 | 6.78 | 13.33 |
| 10 | BPH | IR22 | 20 | 30 | 2 | 5 | 3.67 | 20.33 | 40.00 |
| 10 | BPH | IR22 | 20 | 30 | 3 | 3 | 0.00 | 0.00 | 0.00 |
| 10 | BPH | IR22 | 20 | 30 | 4 | 3 | 0.00 | 0.00 | 0.00 |
| 10 | BPH | IR22 | 20 | 35 | 1 | 5 | 0.00 | 0.00 | 0.00 |
| 10 | BPH | IR22 | 20 | 35 | 2 | 3 | 0.00 | 0.00 | 0.00 |
| 10 | BPH | IR22 | 20 | 35 | 3 | 3 | 0.00 | 0.00 | 0.00 |
| 10 | BPH | IR22 | 20 | 35 | 4 | 3 | 0.00 | 0.00 | 0.00 |
| 10 | BPH | IR22 | 30 | 15 | 1 | 3 | 1.00 | 1.94 | 88.89 |
| 10 | BPH | IR22 | 30 | 15 | 2 | 3 | 1.00 | 0.00 | 100.00 |
| 10 | BPH | IR22 | 30 | 15 | 3 | 3 | 1.00 | 3.50 | 66.67 |
| 10 | BPH | IR22 | 30 | 15 | 4 | 3 | 1.00 | 2.33 | 100.00 |
| 10 | BPH | IR22 | 30 | 20 | 1 | 5 | 1.00 | 4.67 | 37.78 |
| 10 | BPH | IR22 | 30 | 20 | 2 | 5 | 2.00 | 10.00 | 80.00 |
| 10 | BPH | IR22 | 30 | 20 | 3 | 3 | 1.00 | 4.00 | 33.33 |
| 10 | BPH | IR22 | 30 | 20 | 4 | 3 | 0.00 | 0.00 | 0.00 |
| 10 | BPH | IR22 | 30 | 25 | 1 | 5 | 0.87 | 5.40 | 33.33 |
| 10 | BPH | IR22 | 30 | 25 | 2 | 5 | 2.60 | 16.20 | 100.00 |
| 10 | BPH | IR22 | 30 | 25 | 3 | 3 | 0.00 | 0.00 | 0.00 |
| 10 | BPH | IR22 | 30 | 25 | 4 | 3 | 0.00 | 0.00 | 0.00 |
| 10 | BPH | IR22 | 30 | 30 | 1 | 5 | 0.33 | 1.00 | 13.33 |
| 10 | BPH | IR22 | 30 | 30 | 2 | 5 | 1.00 | 3.00 | 40.00 |
| 10 | BPH | IR22 | 30 | 30 | 3 | 3 | 0.00 | 0.00 | 0.00 |
| 10 | BPH | IR22 | 30 | 30 | 4 | 3 | 0.00 | 0.00 | 0.00 |
| 10 | BPH | IR22 | 30 | 35 | 1 | 5 | 1.00 | 0.00 | 20.00 |
| 10 | BPH | IR22 | 30 | 35 | 2 | 3 | 0.00 | 0.00 | 0.00 |
| 10 | BPH | IR22 | 30 | 35 | 3 | 3 | 0.00 | 0.00 | 0.00 |
| 10 | BPH | IR22 | 30 | 35 | 4 | 3 | 0.00 | 0.00 | 0.00 |
| 10 | BPH | T65 | 20 | 15 | 1 | 3 | 0.67 | 0.00 | 33.33 |
| 10 | BPH | T65 | 20 | 15 | 2 | 3 | 1.00 | 0.00 | 66.67 |
| 10 | BPH | T65 | 20 | 15 | 3 | 3 | 0.00 | 0.00 | 0.00 |
| 10 | BPH | T65 | 20 | 15 | 4 | 3 | 1.00 | 0.00 | 33.33 |
| 10 | BPH | T65 | 20 | 20 | 1 | 5 | 3.67 | 16.44 | 53.33 |
| 10 | BPH | T65 | 20 | 20 | 2 | 5 | 6.00 | 18.33 | 60.00 |
| 10 | BPH | T65 | 20 | 20 | 3 | 3 | 1.00 | 3.00 | 66.67 |
| 10 | BPH | T65 | 20 | 20 | 4 | 3 | 4.00 | 28.00 | 33.33 |
| 10 | BPH | T65 | 20 | 25 | 1 | 5 | 0.00 | 0.00 | 0.00 |
| 10 | BPH | T65 | 20 | 25 | 2 | 5 | 0.00 | 0.00 | 0.00 |
| 10 | BPH | T65 | 20 | 25 | 3 | 3 | 0.00 | 0.00 | 0.00 |
| 10 | BPH | T65 | 20 | 25 | 4 | 3 | 0.00 | 0.00 | 0.00 |
| 10 | BPH | T65 | 20 | 30 | 1 | 5 | 3.00 | 8.67 | 17.78 |
| 10 | BPH | T65 | 20 | 30 | 2 | 5 | 6.00 | 18.00 | 20.00 |
| 10 | BPH | T65 | 20 | 30 | 3 | 3 | 3.00 | 8.00 | 33.33 |
| 10 | BPH | T65 | 20 | 30 | 4 | 3 | 0.00 | 0.00 | 0.00 |
| 10 | BPH | T65 | 20 | 35 | 1 | 5 | 0.00 | 0.00 | 0.00 |
| 10 | BPH | T65 | 20 | 35 | 2 | 3 | 0.00 | 0.00 | 0.00 |
| 10 | BPH | T65 | 20 | 35 | 3 | 3 | 0.00 | 0.00 | 0.00 |
| 10 | BPH | T65 | 20 | 35 | 4 | 3 | 0.00 | 0.00 | 0.00 |
| 10 | BPH | T65 | 30 | 15 | 1 | 3 | 1.17 | 1.00 | 33.33 |
| 10 | BPH | T65 | 30 | 15 | 2 | 3 | 0.00 | 0.00 | 0.00 |
| 10 | BPH | T65 | 30 | 15 | 3 | 3 | 2.50 | 3.00 | 66.67 |
| 10 | BPH | T65 | 30 | 15 | 4 | 3 | 1.00 | 0.00 | 33.33 |
| 10 | BPH | T65 | 30 | 20 | 1 | 5 | 0.58 | 5.33 | 26.67 |
| 10 | BPH | T65 | 30 | 20 | 2 | 5 | 1.75 | 16.00 | 80.00 |
| 10 | BPH | T65 | 30 | 20 | 3 | 3 | 0.00 | 0.00 | 0.00 |
| 10 | BPH | T65 | 30 | 20 | 4 | 3 | 0.00 | 0.00 | 0.00 |
| 10 | BPH | T65 | 30 | 25 | 1 | 5 | 1.67 | 9.58 | 37.78 |
| 10 | BPH | T65 | 30 | 25 | 2 | 5 | 4.00 | 20.75 | 80.00 |
| 10 | BPH | T65 | 30 | 25 | 3 | 3 | 0.00 | 0.00 | 0.00 |
| 10 | BPH | T65 | 30 | 25 | 4 | 3 | 1.00 | 8.00 | 33.33 |
| 10 | BPH | T65 | 30 | 30 | 1 | 5 | 1.50 | 5.67 | 37.78 |
| 10 | BPH | T65 | 30 | 30 | 2 | 5 | 3.50 | 17.00 | 80.00 |
| 10 | BPH | T65 | 30 | 30 | 3 | 3 | 1.00 | 0.00 | 33.33 |
| 10 | BPH | T65 | 30 | 30 | 4 | 3 | 0.00 | 0.00 | 0.00 |
| 10 | BPH | T65 | 30 | 35 | 1 | 5 | 0.00 | 0.00 | 0.00 |
| 10 | BPH | T65 | 30 | 35 | 2 | 3 | 0.00 | 0.00 | 0.00 |
| 10 | BPH | T65 | 30 | 35 | 3 | 3 | 0.00 | 0.00 | 0.00 |
| 10 | BPH | T65 | 30 | 35 | 4 | 3 | 0.00 | 0.00 | 0.00 |
| 10 | WBPH | IR22 | 20 | 15 | 1 | 3 | 1.00 | 0.00 | 44.44 |
| 10 | WBPH | IR22 | 20 | 15 | 2 | 3 | 1.00 | 0.00 | 33.33 |
| 10 | WBPH | IR22 | 20 | 15 | 3 | 3 | 1.00 | 0.00 | 33.33 |
| 10 | WBPH | IR22 | 20 | 15 | 4 | 3 | 1.00 | 0.00 | 66.67 |
| 10 | WBPH | IR22 | 20 | 20 | 1 | 5 | 0.83 | 5.00 | 22.22 |
| 10 | WBPH | IR22 | 20 | 20 | 2 | 5 | 0.00 | 0.00 | 0.00 |
| 10 | WBPH | IR22 | 20 | 20 | 3 | 3 | 0.00 | 0.00 | 0.00 |
| 10 | WBPH | IR22 | 20 | 20 | 4 | 3 | 2.50 | 15.00 | 66.67 |
| 10 | WBPH | IR22 | 20 | 25 | 1 | 5 | 0.33 | 1.67 | 11.11 |
| 10 | WBPH | IR22 | 20 | 25 | 2 | 5 | 0.00 | 0.00 | 0.00 |
| 10 | WBPH | IR22 | 20 | 25 | 3 | 3 | 0.00 | 0.00 | 0.00 |
| 10 | WBPH | IR22 | 20 | 25 | 4 | 3 | 1.00 | 5.00 | 33.33 |
| 10 | WBPH | IR22 | 20 | 30 | 1 | 5 | 0.00 | 0.00 | 0.00 |
| 10 | WBPH | IR22 | 20 | 30 | 2 | 5 | 0.00 | 0.00 | 0.00 |
| 10 | WBPH | IR22 | 20 | 30 | 3 | 3 | 0.00 | 0.00 | 0.00 |
| 10 | WBPH | IR22 | 20 | 30 | 4 | 3 | 0.00 | 0.00 | 0.00 |
| 10 | WBPH | IR22 | 20 | 35 | 1 | 5 | 0.00 | 0.00 | 0.00 |
| 10 | WBPH | IR22 | 20 | 35 | 2 | 3 | 0.00 | 0.00 | 0.00 |
| 10 | WBPH | IR22 | 20 | 35 | 3 | 3 | 0.00 | 0.00 | 0.00 |
| 10 | WBPH | IR22 | 20 | 35 | 4 | 3 | 0.00 | 0.00 | 0.00 |
| 10 | WBPH | IR22 | 30 | 15 | 1 | 1 | 0.00 | 0.00 | 67.00 |
| 10 | WBPH | IR22 | 30 | 15 | 2 | 1 | 0.00 | 0.00 | 60.00 |
| 10 | WBPH | IR22 | 30 | 15 | 3 | 1 | 0.00 | 0.00 | 80.00 |
| 10 | WBPH | IR22 | 30 | 15 | 4 | 1 | 0.00 | 0.00 | 60.00 |
| 10 | WBPH | IR22 | 30 | 20 | 1 | 5 | 0.67 | 1.67 | 22.22 |
| 10 | WBPH | IR22 | 30 | 20 | 2 | 5 | 0.00 | 0.00 | 0.00 |
| 10 | WBPH | IR22 | 30 | 20 | 3 | 3 | 1.00 | 0.00 | 33.33 |
| 10 | WBPH | IR22 | 30 | 20 | 4 | 3 | 1.00 | 5.00 | 33.33 |
| 10 | WBPH | IR22 | 30 | 25 | 1 | 5 | 0.00 | 0.00 | 0.00 |
| 10 | WBPH | IR22 | 30 | 25 | 2 | 5 | 0.00 | 0.00 | 0.00 |
| 10 | WBPH | IR22 | 30 | 25 | 3 | 5 | 0.00 | 0.00 | 0.00 |
| 10 | WBPH | IR22 | 30 | 25 | 4 | 5 | 0.00 | 0.00 | 0.00 |
| 10 | WBPH | IR22 | 30 | 30 | 1 | 5 | 0.00 | 0.00 | 0.00 |
| 10 | WBPH | IR22 | 30 | 30 | 2 | 5 | 0.00 | 0.00 | 0.00 |
| 10 | WBPH | IR22 | 30 | 30 | 3 | 3 | 0.00 | 0.00 | 0.00 |
| 10 | WBPH | IR22 | 30 | 30 | 4 | 3 | 0.00 | 0.00 | 0.00 |
| 10 | WBPH | IR22 | 30 | 35 | 1 | 5 | 0.00 | 0.00 | 0.00 |
| 10 | WBPH | IR22 | 30 | 35 | 2 | 3 | 0.00 | 0.00 | 0.00 |
| 10 | WBPH | IR22 | 30 | 35 | 3 | 3 | 0.00 | 0.00 | 0.00 |
| 10 | WBPH | IR22 | 30 | 35 | 4 | 3 | 0.00 | 0.00 | 0.00 |
| 10 | WBPH | T65 | 20 | 15 | 1 | 3 | 1.00 | 2.50 | 66.67 |
| 10 | WBPH | T65 | 20 | 15 | 2 | 3 | 1.00 | 0.00 | 66.67 |
| 10 | WBPH | T65 | 20 | 15 | 3 | 3 | 1.00 | 7.50 | 66.67 |
| 10 | WBPH | T65 | 20 | 15 | 4 | 3 | 1.00 | 0.00 | 66.67 |
| 10 | WBPH | T65 | 20 | 20 | 1 | 5 | 1.33 | 7.33 | 22.22 |
| 10 | WBPH | T65 | 20 | 20 | 2 | 5 | 0.00 | 0.00 | 0.00 |
| 10 | WBPH | T65 | 20 | 20 | 3 | 3 | 1.00 | 0.00 | 33.33 |
| 10 | WBPH | T65 | 20 | 20 | 4 | 3 | 3.00 | 22.00 | 33.33 |
| 10 | WBPH | T65 | 20 | 25 | 1 | 5 | 0.50 | 2.83 | 22.22 |
| 10 | WBPH | T65 | 20 | 25 | 2 | 5 | 0.00 | 0.00 | 0.00 |
| 10 | WBPH | T65 | 20 | 25 | 3 | 3 | 0.00 | 0.00 | 0.00 |
| 10 | WBPH | T65 | 20 | 25 | 4 | 3 | 1.50 | 8.50 | 66.67 |
| 10 | WBPH | T65 | 20 | 30 | 1 | 5 | 0.00 | 0.00 | 0.00 |
| 10 | WBPH | T65 | 20 | 30 | 2 | 5 | 0.00 | 0.00 | 0.00 |
| 10 | WBPH | T65 | 20 | 30 | 3 | 3 | 0.00 | 0.00 | 0.00 |
| 10 | WBPH | T65 | 20 | 30 | 4 | 3 | 1.00 | 4.00 | 33.33 |
| 10 | WBPH | T65 | 20 | 35 | 1 | 5 | 0.00 | 0.00 | 0.00 |
| 10 | WBPH | T65 | 20 | 35 | 2 | 3 | 0.00 | 0.00 | 0.00 |
| 10 | WBPH | T65 | 20 | 35 | 3 | 3 | 0.00 | 0.00 | 0.00 |
| 10 | WBPH | T65 | 20 | 35 | 4 | 3 | 0.00 | 0.00 | 0.00 |
| 10 | WBPH | T65 | 30 | 15 | 1 | 1 | 0.00 | 0.00 | 20.00 |
| 10 | WBPH | T65 | 30 | 15 | 2 | 1 | 0.00 | 0.00 | 60.00 |
| 10 | WBPH | T65 | 30 | 15 | 3 | 1 | 0.00 | 0.00 | 80.00 |
| 10 | WBPH | T65 | 30 | 15 | 4 | 1 | 0.00 | 0.00 | 33.00 |
| 10 | WBPH | T65 | 30 | 20 | 1 | 5 | 0.67 | 0.00 | 22.22 |
| 10 | WBPH | T65 | 30 | 20 | 2 | 5 | 0.00 | 0.00 | 0.00 |
| 10 | WBPH | T65 | 30 | 20 | 3 | 3 | 1.00 | 0.00 | 33.33 |
| 10 | WBPH | T65 | 30 | 20 | 4 | 3 | 1.00 | 0.00 | 33.33 |
| 10 | WBPH | T65 | 30 | 25 | 1 | 5 | 0.00 | 0.00 | 0.00 |
| 10 | WBPH | T65 | 30 | 25 | 2 | 5 | 0.00 | 0.00 | 0.00 |
| 10 | WBPH | T65 | 30 | 25 | 3 | 5 | 0.00 | 0.00 | 0.00 |
| 10 | WBPH | T65 | 30 | 25 | 4 | 5 | 0.00 | 0.00 | 0.00 |
| 10 | WBPH | T65 | 30 | 30 | 1 | 5 | 0.00 | 0.00 | 0.00 |
| 10 | WBPH | T65 | 30 | 30 | 2 | 5 | 0.00 | 0.00 | 0.00 |
| 10 | WBPH | T65 | 30 | 30 | 3 | 3 | 0.00 | 0.00 | 0.00 |
| 10 | WBPH | T65 | 30 | 30 | 4 | 3 | 0.00 | 0.00 | 0.00 |
| 10 | WBPH | T65 | 30 | 35 | 1 | 5 | 0.00 | 0.00 | 0.00 |
| 10 | WBPH | T65 | 30 | 35 | 2 | 3 | 0.00 | 0.00 | 0.00 |
| 10 | WBPH | T65 | 30 | 35 | 3 | 3 | 0.00 | 0.00 | 0.00 |
| 10 | WBPH | T65 | 30 | 35 | 4 | 3 | 0.00 | 0.00 | 0.00 |
| 11 | BPH | IR22 | 20 | 15 | 1 | 3 | 0.67 | 0.67 | 44.44 |
| 11 | BPH | IR22 | 20 | 15 | 2 | 3 | 1.00 | 0.00 | 33.33 |
| 11 | BPH | IR22 | 20 | 15 | 3 | 3 | 0.00 | 0.00 | 0.00 |
| 11 | BPH | IR22 | 20 | 15 | 4 | 3 | 1.00 | 2.00 | 100.00 |
| 11 | BPH | IR22 | 20 | 20 | 1 | 5 | 1.67 | 2.39 | 42.22 |
| 11 | BPH | IR22 | 20 | 20 | 2 | 5 | 1.00 | 1.67 | 60.00 |
| 11 | BPH | IR22 | 20 | 20 | 3 | 3 | 4.00 | 5.50 | 66.67 |
| 11 | BPH | IR22 | 20 | 20 | 4 | 3 | 0.00 | 0.00 | 0.00 |
| 11 | BPH | IR22 | 20 | 25 | 1 | 5 | 1.67 | 6.50 | 13.33 |
| 11 | BPH | IR22 | 20 | 25 | 2 | 5 | 5.00 | 19.50 | 40.00 |
| 11 | BPH | IR22 | 20 | 25 | 3 | 3 | 0.00 | 0.00 | 0.00 |
| 11 | BPH | IR22 | 20 | 25 | 4 | 3 | 0.00 | 0.00 | 0.00 |
| 11 | BPH | IR22 | 20 | 30 | 1 | 5 | 2.67 | 9.50 | 13.33 |
| 11 | BPH | IR22 | 20 | 30 | 2 | 5 | 8.00 | 28.50 | 40.00 |
| 11 | BPH | IR22 | 20 | 30 | 3 | 3 | 0.00 | 0.00 | 0.00 |
| 11 | BPH | IR22 | 20 | 30 | 4 | 3 | 0.00 | 0.00 | 0.00 |
| 11 | BPH | IR22 | 20 | 35 | 1 | 5 | 0.00 | 0.00 | 0.00 |
| 11 | BPH | IR22 | 20 | 35 | 2 | 3 | 0.00 | 0.00 | 0.00 |
| 11 | BPH | IR22 | 20 | 35 | 3 | 3 | 0.00 | 0.00 | 0.00 |
| 11 | BPH | IR22 | 20 | 35 | 4 | 3 | 0.00 | 0.00 | 0.00 |
| 11 | BPH | IR22 | 30 | 15 | 1 | 3 | 1.00 | 0.56 | 77.78 |
| 11 | BPH | IR22 | 30 | 15 | 2 | 3 | 1.00 | 0.00 | 66.67 |
| 11 | BPH | IR22 | 30 | 15 | 3 | 3 | 1.00 | 0.00 | 66.67 |
| 11 | BPH | IR22 | 30 | 15 | 4 | 3 | 1.00 | 1.67 | 100.00 |
| 11 | BPH | IR22 | 30 | 20 | 1 | 5 | 1.00 | 3.44 | 31.11 |
| 11 | BPH | IR22 | 30 | 20 | 2 | 5 | 2.00 | 10.33 | 60.00 |
| 11 | BPH | IR22 | 30 | 20 | 3 | 3 | 1.00 | 0.00 | 33.33 |
| 11 | BPH | IR22 | 30 | 20 | 4 | 3 | 0.00 | 0.00 | 0.00 |
| 11 | BPH | IR22 | 30 | 25 | 1 | 5 | 0.60 | 4.67 | 33.33 |
| 11 | BPH | IR22 | 30 | 25 | 2 | 5 | 1.80 | 14.00 | 100.00 |
| 11 | BPH | IR22 | 30 | 25 | 3 | 3 | 0.00 | 0.00 | 0.00 |
| 11 | BPH | IR22 | 30 | 25 | 4 | 3 | 0.00 | 0.00 | 0.00 |
| 11 | BPH | IR22 | 30 | 30 | 1 | 5 | 0.00 | 0.00 | 0.00 |
| 11 | BPH | IR22 | 30 | 30 | 2 | 5 | 1.00 | 0.00 | 40.00 |
| 11 | BPH | IR22 | 30 | 30 | 3 | 3 | 0.00 | 0.00 | 0.00 |
| 11 | BPH | IR22 | 30 | 30 | 4 | 3 | 0.00 | 0.00 | 0.00 |
| 11 | BPH | IR22 | 30 | 35 | 1 | 5 | 0.00 | 0.00 | 0.00 |
| 11 | BPH | IR22 | 30 | 35 | 2 | 3 | 0.00 | 0.00 | 0.00 |
| 11 | BPH | IR22 | 30 | 35 | 3 | 3 | 0.00 | 0.00 | 0.00 |
| 11 | BPH | IR22 | 30 | 35 | 4 | 3 | 0.00 | 0.00 | 0.00 |
| 11 | BPH | T65 | 20 | 15 | 1 | 3 | 0.33 | 0.00 | 11.11 |
| 11 | BPH | T65 | 20 | 15 | 2 | 3 | 1.00 | 0.00 | 33.33 |
| 11 | BPH | T65 | 20 | 15 | 3 | 3 | 0.00 | 0.00 | 0.00 |
| 11 | BPH | T65 | 20 | 15 | 4 | 3 | 0.00 | 0.00 | 0.00 |
| 11 | BPH | T65 | 20 | 20 | 1 | 5 | 2.89 | 9.89 | 53.33 |
| 11 | BPH | T65 | 20 | 20 | 2 | 5 | 5.67 | 16.67 | 60.00 |
| 11 | BPH | T65 | 20 | 20 | 3 | 3 | 1.00 | 1.00 | 66.67 |
| 11 | BPH | T65 | 20 | 20 | 4 | 3 | 2.00 | 12.00 | 33.33 |
| 11 | BPH | T65 | 20 | 25 | 1 | 5 | 0.00 | 0.00 | 0.00 |
| 11 | BPH | T65 | 20 | 25 | 2 | 5 | 0.00 | 0.00 | 0.00 |
| 11 | BPH | T65 | 20 | 25 | 3 | 3 | 0.00 | 0.00 | 0.00 |
| 11 | BPH | T65 | 20 | 25 | 4 | 3 | 0.00 | 0.00 | 0.00 |
| 11 | BPH | T65 | 20 | 30 | 1 | 5 | 1.00 | 3.67 | 17.78 |
| 11 | BPH | T65 | 20 | 30 | 2 | 5 | 0.00 | 0.00 | 20.00 |
| 11 | BPH | T65 | 20 | 30 | 3 | 3 | 3.00 | 11.00 | 33.33 |
| 11 | BPH | T65 | 20 | 30 | 4 | 3 | 0.00 | 0.00 | 0.00 |
| 11 | BPH | T65 | 20 | 35 | 1 | 5 | 0.00 | 0.00 | 0.00 |
| 11 | BPH | T65 | 20 | 35 | 2 | 3 | 0.00 | 0.00 | 0.00 |
| 11 | BPH | T65 | 20 | 35 | 3 | 3 | 0.00 | 0.00 | 0.00 |
| 11 | BPH | T65 | 20 | 35 | 4 | 3 | 0.00 | 0.00 | 0.00 |
| 11 | BPH | T65 | 30 | 15 | 1 | 3 | 0.67 | 0.00 | 33.33 |
| 11 | BPH | T65 | 30 | 15 | 2 | 3 | 0.00 | 0.00 | 0.00 |
| 11 | BPH | T65 | 30 | 15 | 3 | 3 | 1.00 | 0.00 | 66.67 |
| 11 | BPH | T65 | 30 | 15 | 4 | 3 | 1.00 | 0.00 | 33.33 |
| 11 | BPH | T65 | 30 | 20 | 1 | 5 | 0.67 | 6.08 | 26.67 |
| 11 | BPH | T65 | 30 | 20 | 2 | 5 | 2.00 | 18.25 | 80.00 |
| 11 | BPH | T65 | 30 | 20 | 3 | 3 | 0.00 | 0.00 | 0.00 |
| 11 | BPH | T65 | 30 | 20 | 4 | 3 | 0.00 | 0.00 | 0.00 |
| 11 | BPH | T65 | 30 | 25 | 1 | 5 | 1.83 | 15.50 | 37.78 |
| 11 | BPH | T65 | 30 | 25 | 2 | 5 | 2.50 | 17.50 | 80.00 |
| 11 | BPH | T65 | 30 | 25 | 3 | 3 | 0.00 | 0.00 | 0.00 |
| 11 | BPH | T65 | 30 | 25 | 4 | 3 | 3.00 | 29.00 | 33.33 |
| 11 | BPH | T65 | 30 | 30 | 1 | 5 | 0.44 | 1.00 | 20.00 |
| 11 | BPH | T65 | 30 | 30 | 2 | 5 | 1.33 | 3.00 | 60.00 |
| 11 | BPH | T65 | 30 | 30 | 3 | 3 | 0.00 | 0.00 | 0.00 |
| 11 | BPH | T65 | 30 | 30 | 4 | 3 | 0.00 | 0.00 | 0.00 |
| 11 | BPH | T65 | 30 | 35 | 1 | 5 | 0.00 | 0.00 | 0.00 |
| 11 | BPH | T65 | 30 | 35 | 2 | 3 | 0.00 | 0.00 | 0.00 |
| 11 | BPH | T65 | 30 | 35 | 3 | 3 | 0.00 | 0.00 | 0.00 |
| 11 | BPH | T65 | 30 | 35 | 4 | 3 | 0.00 | 0.00 | 0.00 |
| 11 | WBPH | IR22 | 20 | 15 | 1 | 3 | 0.33 | 0.00 | 22.22 |
| 11 | WBPH | IR22 | 20 | 15 | 2 | 3 | 0.00 | 0.00 | 0.00 |
| 11 | WBPH | IR22 | 20 | 15 | 3 | 3 | 0.00 | 0.00 | 0.00 |
| 11 | WBPH | IR22 | 20 | 15 | 4 | 3 | 1.00 | 0.00 | 66.67 |
| 11 | WBPH | IR22 | 20 | 20 | 1 | 5 | 0.33 | 0.00 | 11.11 |
| 11 | WBPH | IR22 | 20 | 20 | 2 | 5 | 0.00 | 0.00 | 0.00 |
| 11 | WBPH | IR22 | 20 | 20 | 3 | 3 | 0.00 | 0.00 | 0.00 |
| 11 | WBPH | IR22 | 20 | 20 | 4 | 3 | 1.00 | 0.00 | 33.33 |
| 11 | WBPH | IR22 | 20 | 25 | 1 | 5 | 0.33 | 0.00 | 11.11 |
| 11 | WBPH | IR22 | 20 | 25 | 2 | 5 | 0.00 | 0.00 | 0.00 |
| 11 | WBPH | IR22 | 20 | 25 | 3 | 3 | 0.00 | 0.00 | 0.00 |
| 11 | WBPH | IR22 | 20 | 25 | 4 | 3 | 1.00 | 0.00 | 33.33 |
| 11 | WBPH | IR22 | 20 | 30 | 1 | 5 | 0.00 | 0.00 | 0.00 |
| 11 | WBPH | IR22 | 20 | 30 | 2 | 5 | 0.00 | 0.00 | 0.00 |
| 11 | WBPH | IR22 | 20 | 30 | 3 | 3 | 0.00 | 0.00 | 0.00 |
| 11 | WBPH | IR22 | 20 | 30 | 4 | 3 | 0.00 | 0.00 | 0.00 |
| 11 | WBPH | IR22 | 20 | 35 | 1 | 5 | 0.00 | 0.00 | 0.00 |
| 11 | WBPH | IR22 | 20 | 35 | 2 | 3 | 0.00 | 0.00 | 0.00 |
| 11 | WBPH | IR22 | 20 | 35 | 3 | 3 | 0.00 | 0.00 | 0.00 |
| 11 | WBPH | IR22 | 20 | 35 | 4 | 3 | 0.00 | 0.00 | 0.00 |
| 11 | WBPH | IR22 | 30 | 15 | 1 | 1 | 0.00 | 0.00 | 67.00 |
| 11 | WBPH | IR22 | 30 | 15 | 2 | 1 | 0.00 | 0.00 | 67.00 |
| 11 | WBPH | IR22 | 30 | 15 | 3 | 1 | 0.00 | 0.00 | 80.00 |
| 11 | WBPH | IR22 | 30 | 15 | 4 | 1 | 0.00 | 0.00 | 60.00 |
| 11 | WBPH | IR22 | 30 | 20 | 1 | 5 | 0.33 | 0.00 | 11.11 |
| 11 | WBPH | IR22 | 30 | 20 | 2 | 5 | 0.00 | 0.00 | 0.00 |
| 11 | WBPH | IR22 | 30 | 20 | 3 | 3 | 0.00 | 0.00 | 0.00 |
| 11 | WBPH | IR22 | 30 | 20 | 4 | 3 | 1.00 | 0.00 | 33.33 |
| 11 | WBPH | IR22 | 30 | 25 | 1 | 5 | 0.00 | 0.00 | 0.00 |
| 11 | WBPH | IR22 | 30 | 25 | 2 | 5 | 0.00 | 0.00 | 0.00 |
| 11 | WBPH | IR22 | 30 | 25 | 3 | 5 | 0.00 | 0.00 | 0.00 |
| 11 | WBPH | IR22 | 30 | 25 | 4 | 5 | 0.00 | 0.00 | 0.00 |
| 11 | WBPH | IR22 | 30 | 30 | 1 | 5 | 0.00 | 0.00 | 0.00 |
| 11 | WBPH | IR22 | 30 | 30 | 2 | 5 | 0.00 | 0.00 | 0.00 |
| 11 | WBPH | IR22 | 30 | 30 | 3 | 3 | 0.00 | 0.00 | 0.00 |
| 11 | WBPH | IR22 | 30 | 30 | 4 | 3 | 0.00 | 0.00 | 0.00 |
| 11 | WBPH | IR22 | 30 | 35 | 1 | 5 | 0.00 | 0.00 | 0.00 |
| 11 | WBPH | IR22 | 30 | 35 | 2 | 3 | 0.00 | 0.00 | 0.00 |
| 11 | WBPH | IR22 | 30 | 35 | 3 | 3 | 0.00 | 0.00 | 0.00 |
| 11 | WBPH | IR22 | 30 | 35 | 4 | 3 | 0.00 | 0.00 | 0.00 |
| 11 | WBPH | T65 | 20 | 15 | 1 | 3 | 1.00 | 1.67 | 55.56 |
| 11 | WBPH | T65 | 20 | 15 | 2 | 3 | 1.00 | 2.00 | 66.67 |
| 11 | WBPH | T65 | 20 | 15 | 3 | 3 | 1.00 | 0.00 | 33.33 |
| 11 | WBPH | T65 | 20 | 15 | 4 | 3 | 1.00 | 3.00 | 66.67 |
| 11 | WBPH | T65 | 20 | 20 | 1 | 5 | 0.67 | 0.00 | 22.22 |
| 11 | WBPH | T65 | 20 | 20 | 2 | 5 | 0.00 | 0.00 | 0.00 |
| 11 | WBPH | T65 | 20 | 20 | 3 | 3 | 1.00 | 0.00 | 33.33 |
| 11 | WBPH | T65 | 20 | 20 | 4 | 3 | 1.00 | 0.00 | 33.33 |
| 11 | WBPH | T65 | 20 | 25 | 1 | 5 | 1.67 | 6.50 | 22.22 |
| 11 | WBPH | T65 | 20 | 25 | 2 | 5 | 0.00 | 0.00 | 0.00 |
| 11 | WBPH | T65 | 20 | 25 | 3 | 3 | 0.00 | 0.00 | 0.00 |
| 11 | WBPH | T65 | 20 | 25 | 4 | 3 | 5.00 | 19.50 | 66.67 |
| 11 | WBPH | T65 | 20 | 30 | 1 | 5 | 0.00 | 0.00 | 0.00 |
| 11 | WBPH | T65 | 20 | 30 | 2 | 5 | 0.00 | 0.00 | 0.00 |
| 11 | WBPH | T65 | 20 | 30 | 3 | 3 | 0.00 | 0.00 | 0.00 |
| 11 | WBPH | T65 | 20 | 30 | 4 | 3 | 0.00 | 0.00 | 0.00 |
| 11 | WBPH | T65 | 20 | 35 | 1 | 5 | 0.00 | 0.00 | 0.00 |
| 11 | WBPH | T65 | 20 | 35 | 2 | 3 | 0.00 | 0.00 | 0.00 |
| 11 | WBPH | T65 | 20 | 35 | 3 | 3 | 0.00 | 0.00 | 0.00 |
| 11 | WBPH | T65 | 20 | 35 | 4 | 3 | 0.00 | 0.00 | 0.00 |
| 11 | WBPH | T65 | 30 | 15 | 1 | 1 | 0.00 | 0.00 | 0.00 |
| 11 | WBPH | T65 | 30 | 15 | 2 | 1 | 0.00 | 0.00 | 33.00 |
| 11 | WBPH | T65 | 30 | 15 | 3 | 1 | 0.00 | 0.00 | 80.00 |
| 11 | WBPH | T65 | 30 | 15 | 4 | 1 | 0.00 | 0.00 | 33.00 |
| 11 | WBPH | T65 | 30 | 20 | 1 | 5 | 0.00 | 0.00 | 0.00 |
| 11 | WBPH | T65 | 30 | 20 | 2 | 5 | 0.00 | 0.00 | 0.00 |
| 11 | WBPH | T65 | 30 | 20 | 3 | 3 | 1.00 | 0.00 | 33.33 |
| 11 | WBPH | T65 | 30 | 20 | 4 | 3 | 1.00 | 0.00 | 33.33 |
| 11 | WBPH | T65 | 30 | 25 | 1 | 5 | 0.00 | 0.00 | 0.00 |
| 11 | WBPH | T65 | 30 | 25 | 2 | 5 | 0.00 | 0.00 | 0.00 |
| 11 | WBPH | T65 | 30 | 25 | 3 | 5 | 0.00 | 0.00 | 0.00 |
| 11 | WBPH | T65 | 30 | 25 | 4 | 5 | 0.00 | 0.00 | 0.00 |
| 11 | WBPH | T65 | 30 | 30 | 1 | 5 | 0.00 | 0.00 | 0.00 |
| 11 | WBPH | T65 | 30 | 30 | 2 | 5 | 0.00 | 0.00 | 0.00 |
| 11 | WBPH | T65 | 30 | 30 | 3 | 3 | 0.00 | 0.00 | 0.00 |
| 11 | WBPH | T65 | 30 | 30 | 4 | 3 | 0.00 | 0.00 | 0.00 |
| 11 | WBPH | T65 | 30 | 35 | 1 | 5 | 0.00 | 0.00 | 0.00 |
| 11 | WBPH | T65 | 30 | 35 | 2 | 3 | 0.00 | 0.00 | 0.00 |
| 11 | WBPH | T65 | 30 | 35 | 3 | 3 | 0.00 | 0.00 | 0.00 |
| 11 | WBPH | T65 | 30 | 35 | 4 | 3 | 0.00 | 0.00 | 0.00 |
| 12 | BPH | IR22 | 20 | 15 | 1 | 3 | 0.67 | 2.33 | 22.22 |
| 12 | BPH | IR22 | 20 | 15 | 2 | 3 | 1.00 | 0.00 | 33.33 |
| 12 | BPH | IR22 | 20 | 15 | 3 | 3 | 0.00 | 0.00 | 0.00 |
| 12 | BPH | IR22 | 20 | 15 | 4 | 3 | 1.00 | 7.00 | 33.33 |
| 12 | BPH | IR22 | 20 | 20 | 1 | 5 | 1.33 | 5.00 | 42.22 |
| 12 | BPH | IR22 | 20 | 20 | 2 | 5 | 3.00 | 11.00 | 60.00 |
| 12 | BPH | IR22 | 20 | 20 | 3 | 3 | 1.00 | 4.00 | 66.67 |
| 12 | BPH | IR22 | 20 | 20 | 4 | 3 | 0.00 | 0.00 | 0.00 |
| 12 | BPH | IR22 | 20 | 25 | 1 | 5 | 0.33 | 1.17 | 13.33 |
| 12 | BPH | IR22 | 20 | 25 | 2 | 5 | 1.00 | 3.50 | 40.00 |
| 12 | BPH | IR22 | 20 | 25 | 3 | 3 | 0.00 | 0.00 | 0.00 |
| 12 | BPH | IR22 | 20 | 25 | 4 | 3 | 0.00 | 0.00 | 0.00 |
| 12 | BPH | IR22 | 20 | 30 | 1 | 5 | 0.50 | 5.17 | 13.33 |
| 12 | BPH | IR22 | 20 | 30 | 2 | 5 | 1.50 | 15.50 | 40.00 |
| 12 | BPH | IR22 | 20 | 30 | 3 | 3 | 0.00 | 0.00 | 0.00 |
| 12 | BPH | IR22 | 20 | 30 | 4 | 3 | 0.00 | 0.00 | 0.00 |
| 12 | BPH | IR22 | 20 | 35 | 1 | 5 | 0.00 | 0.00 | 0.00 |
| 12 | BPH | IR22 | 20 | 35 | 2 | 3 | 0.00 | 0.00 | 0.00 |
| 12 | BPH | IR22 | 20 | 35 | 3 | 3 | 0.00 | 0.00 | 0.00 |
| 12 | BPH | IR22 | 20 | 35 | 4 | 3 | 0.00 | 0.00 | 0.00 |
| 12 | BPH | IR22 | 30 | 15 | 1 | 3 | 1.00 | 0.00 | 66.67 |
| 12 | BPH | IR22 | 30 | 15 | 2 | 3 | 1.00 | 0.00 | 33.33 |
| 12 | BPH | IR22 | 30 | 15 | 3 | 3 | 1.00 | 0.00 | 66.67 |
| 12 | BPH | IR22 | 30 | 15 | 4 | 3 | 1.00 | 0.00 | 100.00 |
| 12 | BPH | IR22 | 30 | 20 | 1 | 5 | 0.67 | 1.89 | 31.11 |
| 12 | BPH | IR22 | 30 | 20 | 2 | 5 | 1.00 | 5.67 | 60.00 |
| 12 | BPH | IR22 | 30 | 20 | 3 | 3 | 1.00 | 0.00 | 33.33 |
| 12 | BPH | IR22 | 30 | 20 | 4 | 3 | 0.00 | 0.00 | 0.00 |
| 12 | BPH | IR22 | 30 | 25 | 1 | 5 | 1.40 | 6.80 | 50.00 |
| 12 | BPH | IR22 | 30 | 25 | 2 | 5 | 2.80 | 13.60 | 100.00 |
| 12 | BPH | IR22 | 30 | 25 | 3 | 3 | 1.40 | 6.80 | 50.00 |
| 12 | BPH | IR22 | 30 | 25 | 4 | 3 | 0.00 | 0.00 | 0.00 |
| 12 | BPH | IR22 | 30 | 30 | 1 | 5 | 0.00 | 0.00 | 0.00 |
| 12 | BPH | IR22 | 30 | 30 | 2 | 5 | 1.00 | 0.00 | 40.00 |
| 12 | BPH | IR22 | 30 | 30 | 3 | 3 | 0.00 | 0.00 | 0.00 |
| 12 | BPH | IR22 | 30 | 30 | 4 | 3 | 0.00 | 0.00 | 0.00 |
| 12 | BPH | IR22 | 30 | 35 | 1 | 5 | 0.00 | 0.00 | 0.00 |
| 12 | BPH | IR22 | 30 | 35 | 2 | 3 | 0.00 | 0.00 | 0.00 |
| 12 | BPH | IR22 | 30 | 35 | 3 | 3 | 0.00 | 0.00 | 0.00 |
| 12 | BPH | IR22 | 30 | 35 | 4 | 3 | 0.00 | 0.00 | 0.00 |
| 12 | BPH | T65 | 20 | 15 | 1 | 3 | 0.00 | 0.00 | 0.00 |
| 12 | BPH | T65 | 20 | 15 | 2 | 3 | 1.00 | 0.00 | 33.33 |
| 12 | BPH | T65 | 20 | 15 | 3 | 3 | 0.00 | 0.00 | 0.00 |
| 12 | BPH | T65 | 20 | 15 | 4 | 3 | 0.00 | 0.00 | 0.00 |
| 12 | BPH | T65 | 20 | 20 | 1 | 5 | 0.56 | 0.33 | 31.11 |
| 12 | BPH | T65 | 20 | 20 | 2 | 5 | 0.67 | 1.00 | 60.00 |
| 12 | BPH | T65 | 20 | 20 | 3 | 3 | 0.00 | 0.00 | 0.00 |
| 12 | BPH | T65 | 20 | 20 | 4 | 3 | 1.00 | 0.00 | 33.33 |
| 12 | BPH | T65 | 20 | 25 | 1 | 5 | 0.00 | 0.00 | 0.00 |
| 12 | BPH | T65 | 20 | 25 | 2 | 5 | 0.00 | 0.00 | 0.00 |
| 12 | BPH | T65 | 20 | 25 | 3 | 3 | 0.00 | 0.00 | 0.00 |
| 12 | BPH | T65 | 20 | 25 | 4 | 3 | 0.00 | 0.00 | 0.00 |
| 12 | BPH | T65 | 20 | 30 | 1 | 5 | 2.00 | 5.67 | 17.78 |
| 12 | BPH | T65 | 20 | 30 | 2 | 5 | 5.00 | 16.00 | 20.00 |
| 12 | BPH | T65 | 20 | 30 | 3 | 3 | 1.00 | 1.00 | 33.33 |
| 12 | BPH | T65 | 20 | 30 | 4 | 3 | 0.00 | 0.00 | 0.00 |
| 12 | BPH | T65 | 20 | 35 | 1 | 5 | 0.00 | 0.00 | 0.00 |
| 12 | BPH | T65 | 20 | 35 | 2 | 3 | 0.00 | 0.00 | 0.00 |
| 12 | BPH | T65 | 20 | 35 | 3 | 3 | 0.00 | 0.00 | 0.00 |
| 12 | BPH | T65 | 20 | 35 | 4 | 3 | 0.00 | 0.00 | 0.00 |
| 12 | BPH | T65 | 30 | 15 | 1 | 3 | 0.33 | 0.00 | 22.22 |
| 12 | BPH | T65 | 30 | 15 | 2 | 3 | 0.00 | 0.00 | 0.00 |
| 12 | BPH | T65 | 30 | 15 | 3 | 3 | 1.00 | 0.00 | 66.67 |
| 12 | BPH | T65 | 30 | 15 | 4 | 3 | 0.00 | 0.00 | 0.00 |
| 12 | BPH | T65 | 30 | 20 | 1 | 5 | 1.58 | 5.50 | 26.67 |
| 12 | BPH | T65 | 30 | 20 | 2 | 5 | 4.75 | 16.50 | 80.00 |
| 12 | BPH | T65 | 30 | 20 | 3 | 3 | 0.00 | 0.00 | 0.00 |
| 12 | BPH | T65 | 30 | 20 | 4 | 3 | 0.00 | 0.00 | 0.00 |
| 12 | BPH | T65 | 30 | 25 | 1 | 5 | 1.88 | 8.38 | 56.67 |
| 12 | BPH | T65 | 30 | 25 | 2 | 5 | 2.75 | 12.75 | 80.00 |
| 12 | BPH | T65 | 30 | 25 | 3 | 3 | 0.00 | 0.00 | 0.00 |
| 12 | BPH | T65 | 30 | 25 | 4 | 3 | 1.00 | 4.00 | 33.33 |
| 12 | BPH | T65 | 30 | 30 | 1 | 5 | 0.00 | 0.00 | 0.00 |
| 12 | BPH | T65 | 30 | 30 | 2 | 5 | 2.67 | 8.33 | 60.00 |
| 12 | BPH | T65 | 30 | 30 | 3 | 3 | 0.00 | 0.00 | 0.00 |
| 12 | BPH | T65 | 30 | 30 | 4 | 3 | 0.00 | 0.00 | 0.00 |
| 12 | BPH | T65 | 30 | 35 | 1 | 5 | 0.00 | 0.00 | 0.00 |
| 12 | BPH | T65 | 30 | 35 | 2 | 3 | 0.00 | 0.00 | 0.00 |
| 12 | BPH | T65 | 30 | 35 | 3 | 3 | 0.00 | 0.00 | 0.00 |
| 12 | BPH | T65 | 30 | 35 | 4 | 3 | 0.00 | 0.00 | 0.00 |
| 12 | WBPH | IR22 | 20 | 15 | 1 | 3 | 0.50 | 7.83 | 22.22 |
| 12 | WBPH | IR22 | 20 | 15 | 2 | 3 | 0.00 | 0.00 | 0.00 |
| 12 | WBPH | IR22 | 20 | 15 | 3 | 3 | 0.00 | 0.00 | 0.00 |
| 12 | WBPH | IR22 | 20 | 15 | 4 | 3 | 1.50 | 23.50 | 66.67 |
| 12 | WBPH | IR22 | 20 | 20 | 1 | 5 | 0.00 | 0.00 | 0.00 |
| 12 | WBPH | IR22 | 20 | 20 | 2 | 5 | 0.00 | 0.00 | 0.00 |
| 12 | WBPH | IR22 | 20 | 20 | 3 | 3 | 0.00 | 0.00 | 0.00 |
| 12 | WBPH | IR22 | 20 | 20 | 4 | 3 | 0.00 | 0.00 | 0.00 |
| 12 | WBPH | IR22 | 20 | 25 | 1 | 5 | 0.33 | 0.00 | 11.11 |
| 12 | WBPH | IR22 | 20 | 25 | 2 | 5 | 0.00 | 0.00 | 0.00 |
| 12 | WBPH | IR22 | 20 | 25 | 3 | 3 | 0.00 | 0.00 | 0.00 |
| 12 | WBPH | IR22 | 20 | 25 | 4 | 3 | 1.00 | 0.00 | 33.33 |
| 12 | WBPH | IR22 | 20 | 30 | 1 | 5 | 0.00 | 0.00 | 0.00 |
| 12 | WBPH | IR22 | 20 | 30 | 2 | 5 | 0.00 | 0.00 | 0.00 |
| 12 | WBPH | IR22 | 20 | 30 | 3 | 3 | 0.00 | 0.00 | 0.00 |
| 12 | WBPH | IR22 | 20 | 30 | 4 | 3 | 0.00 | 0.00 | 0.00 |
| 12 | WBPH | IR22 | 20 | 35 | 1 | 5 | 0.00 | 0.00 | 0.00 |
| 12 | WBPH | IR22 | 20 | 35 | 2 | 3 | 0.00 | 0.00 | 0.00 |
| 12 | WBPH | IR22 | 20 | 35 | 3 | 3 | 0.00 | 0.00 | 0.00 |
| 12 | WBPH | IR22 | 20 | 35 | 4 | 3 | 0.00 | 0.00 | 0.00 |
| 12 | WBPH | IR22 | 30 | 15 | 1 | 1 | 0.00 | 0.00 | 67.00 |
| 12 | WBPH | IR22 | 30 | 15 | 2 | 1 | 0.00 | 0.00 | 67.00 |
| 12 | WBPH | IR22 | 30 | 15 | 3 | 1 | 0.00 | 0.00 | 67.00 |
| 12 | WBPH | IR22 | 30 | 15 | 4 | 1 | 0.00 | 0.00 | 33.00 |
| 12 | WBPH | IR22 | 30 | 20 | 1 | 5 | 0.33 | 0.00 | 11.11 |
| 12 | WBPH | IR22 | 30 | 20 | 2 | 5 | 0.00 | 0.00 | 0.00 |
| 12 | WBPH | IR22 | 30 | 20 | 3 | 3 | 0.00 | 0.00 | 0.00 |
| 12 | WBPH | IR22 | 30 | 20 | 4 | 3 | 1.00 | 0.00 | 33.33 |
| 12 | WBPH | IR22 | 30 | 25 | 1 | 5 | 0.00 | 0.00 | 0.00 |
| 12 | WBPH | IR22 | 30 | 25 | 2 | 5 | 0.00 | 0.00 | 0.00 |
| 12 | WBPH | IR22 | 30 | 25 | 3 | 5 | 0.00 | 0.00 | 0.00 |
| 12 | WBPH | IR22 | 30 | 25 | 4 | 5 | 0.00 | 0.00 | 0.00 |
| 12 | WBPH | IR22 | 30 | 30 | 1 | 5 | 0.00 | 0.00 | 0.00 |
| 12 | WBPH | IR22 | 30 | 30 | 2 | 5 | 0.00 | 0.00 | 0.00 |
| 12 | WBPH | IR22 | 30 | 30 | 3 | 5 | 0.00 | 0.00 | 0.00 |
| 12 | WBPH | IR22 | 30 | 30 | 4 | 3 | 0.00 | 0.00 | 0.00 |
| 12 | WBPH | IR22 | 30 | 35 | 1 | 5 | 0.00 | 0.00 | 0.00 |
| 12 | WBPH | IR22 | 30 | 35 | 2 | 3 | 0.00 | 0.00 | 0.00 |
| 12 | WBPH | IR22 | 30 | 35 | 3 | 3 | 0.00 | 0.00 | 0.00 |
| 12 | WBPH | IR22 | 30 | 35 | 4 | 3 | 0.00 | 0.00 | 0.00 |
| 12 | WBPH | T65 | 20 | 15 | 1 | 3 | 1.00 | 1.33 | 55.56 |
| 12 | WBPH | T65 | 20 | 15 | 2 | 3 | 1.00 | 0.00 | 66.67 |
| 12 | WBPH | T65 | 20 | 15 | 3 | 3 | 1.00 | 4.00 | 33.33 |
| 12 | WBPH | T65 | 20 | 15 | 4 | 3 | 1.00 | 0.00 | 66.67 |
| 12 | WBPH | T65 | 20 | 20 | 1 | 5 | 1.00 | 1.67 | 22.22 |
| 12 | WBPH | T65 | 20 | 20 | 2 | 5 | 0.00 | 0.00 | 0.00 |
| 12 | WBPH | T65 | 20 | 20 | 3 | 3 | 1.00 | 0.00 | 33.33 |
| 12 | WBPH | T65 | 20 | 20 | 4 | 3 | 2.00 | 5.00 | 33.33 |
| 12 | WBPH | T65 | 20 | 25 | 1 | 5 | 0.50 | 3.33 | 22.22 |
| 12 | WBPH | T65 | 20 | 25 | 2 | 5 | 0.00 | 0.00 | 0.00 |
| 12 | WBPH | T65 | 20 | 25 | 3 | 3 | 0.00 | 0.00 | 0.00 |
| 12 | WBPH | T65 | 20 | 25 | 4 | 3 | 1.50 | 10.00 | 66.67 |
| 12 | WBPH | T65 | 20 | 30 | 1 | 5 | 0.00 | 0.00 | 0.00 |
| 12 | WBPH | T65 | 20 | 30 | 2 | 5 | 0.00 | 0.00 | 0.00 |
| 12 | WBPH | T65 | 20 | 30 | 3 | 3 | 0.00 | 0.00 | 0.00 |
| 12 | WBPH | T65 | 20 | 30 | 4 | 3 | 0.00 | 0.00 | 0.00 |
| 12 | WBPH | T65 | 20 | 35 | 1 | 5 | 0.00 | 0.00 | 0.00 |
| 12 | WBPH | T65 | 20 | 35 | 2 | 3 | 0.00 | 0.00 | 0.00 |
| 12 | WBPH | T65 | 20 | 35 | 3 | 3 | 0.00 | 0.00 | 0.00 |
| 12 | WBPH | T65 | 20 | 35 | 4 | 3 | 0.00 | 0.00 | 0.00 |
| 12 | WBPH | T65 | 30 | 15 | 1 | 1 | 0.00 | 0.00 | 0.00 |
| 12 | WBPH | T65 | 30 | 15 | 2 | 1 | 0.00 | 0.00 | 33.00 |
| 12 | WBPH | T65 | 30 | 15 | 3 | 1 | 0.00 | 0.00 | 80.00 |
| 12 | WBPH | T65 | 30 | 15 | 4 | 1 | 0.00 | 0.00 | 33.00 |
| 12 | WBPH | T65 | 30 | 20 | 1 | 5 | 0.00 | 0.00 | 0.00 |
| 12 | WBPH | T65 | 30 | 20 | 2 | 5 | 0.00 | 0.00 | 0.00 |
| 12 | WBPH | T65 | 30 | 20 | 3 | 3 | 1.00 | 0.00 | 33.33 |
| 12 | WBPH | T65 | 30 | 20 | 4 | 3 | 0.00 | 0.00 | 0.00 |
| 12 | WBPH | T65 | 30 | 25 | 1 | 5 | 0.00 | 0.00 | 0.00 |
| 12 | WBPH | T65 | 30 | 25 | 2 | 5 | 0.00 | 0.00 | 0.00 |
| 12 | WBPH | T65 | 30 | 25 | 3 | 5 | 0.00 | 0.00 | 0.00 |
| 12 | WBPH | T65 | 30 | 25 | 4 | 5 | 0.00 | 0.00 | 0.00 |
| 12 | WBPH | T65 | 30 | 30 | 1 | 5 | 0.00 | 0.00 | 0.00 |
| 12 | WBPH | T65 | 30 | 30 | 2 | 5 | 0.00 | 0.00 | 0.00 |
| 12 | WBPH | T65 | 30 | 30 | 3 | 3 | 0.00 | 0.00 | 0.00 |
| 12 | WBPH | T65 | 30 | 30 | 4 | 3 | 0.00 | 0.00 | 0.00 |
| 12 | WBPH | T65 | 30 | 35 | 1 | 5 | 0.00 | 0.00 | 0.00 |
| 12 | WBPH | T65 | 30 | 35 | 2 | 3 | 0.00 | 0.00 | 0.00 |
| 12 | WBPH | T65 | 30 | 35 | 3 | 3 | 0.00 | 0.00 | 0.00 |
| 12 | WBPH | T65 | 30 | 35 | 4 | 3 | 0.00 | 0.00 | 0.00 |
| 13 | BPH | IR22 | 20 | 15 | 1 | 3 | 0.67 | 0.00 | 22.22 |
| 13 | BPH | IR22 | 20 | 15 | 2 | 3 | 1.00 | 0.00 | 33.33 |
| 13 | BPH | IR22 | 20 | 15 | 3 | 3 | 0.00 | 0.00 | 0.00 |
| 13 | BPH | IR22 | 20 | 15 | 4 | 3 | 1.00 | 0.00 | 33.33 |
| 13 | BPH | IR22 | 20 | 20 | 1 | 5 | 0.67 | 5.00 | 31.11 |
| 13 | BPH | IR22 | 20 | 20 | 2 | 5 | 1.00 | 2.00 | 60.00 |
| 13 | BPH | IR22 | 20 | 20 | 3 | 3 | 1.00 | 13.00 | 33.33 |
| 13 | BPH | IR22 | 20 | 20 | 4 | 3 | 0.00 | 0.00 | 0.00 |
| 13 | BPH | IR22 | 20 | 25 | 1 | 5 | 0.50 | 1.67 | 13.33 |
| 13 | BPH | IR22 | 20 | 25 | 2 | 5 | 1.50 | 5.00 | 40.00 |
| 13 | BPH | IR22 | 20 | 25 | 3 | 3 | 0.00 | 0.00 | 0.00 |
| 13 | BPH | IR22 | 20 | 25 | 4 | 3 | 0.00 | 0.00 | 0.00 |
| 13 | BPH | IR22 | 20 | 30 | 1 | 5 | 1.83 | 8.17 | 13.33 |
| 13 | BPH | IR22 | 20 | 30 | 2 | 5 | 5.50 | 24.50 | 40.00 |
| 13 | BPH | IR22 | 20 | 30 | 3 | 3 | 0.00 | 0.00 | 0.00 |
| 13 | BPH | IR22 | 20 | 30 | 4 | 3 | 0.00 | 0.00 | 0.00 |
| 13 | BPH | IR22 | 20 | 35 | 1 | 5 | 0.00 | 0.00 | 0.00 |
| 13 | BPH | IR22 | 20 | 35 | 2 | 3 | 0.00 | 0.00 | 0.00 |
| 13 | BPH | IR22 | 20 | 35 | 3 | 3 | 0.00 | 0.00 | 0.00 |
| 13 | BPH | IR22 | 20 | 35 | 4 | 3 | 0.00 | 0.00 | 0.00 |
| 13 | BPH | IR22 | 30 | 15 | 1 | 1 | 1.00 | 0.50 | 55.56 |
| 13 | BPH | IR22 | 30 | 15 | 2 | 1 | 1.00 | 0.00 | 33.33 |
| 13 | BPH | IR22 | 30 | 15 | 3 | 3 | 1.00 | 1.50 | 66.67 |
| 13 | BPH | IR22 | 30 | 15 | 4 | 3 | 1.00 | 0.00 | 66.67 |
| 13 | BPH | IR22 | 30 | 20 | 1 | 5 | 0.33 | 0.22 | 20.00 |
| 13 | BPH | IR22 | 30 | 20 | 2 | 5 | 1.00 | 0.67 | 60.00 |
| 13 | BPH | IR22 | 30 | 20 | 3 | 3 | 0.00 | 0.00 | 0.00 |
| 13 | BPH | IR22 | 30 | 20 | 4 | 3 | 0.00 | 0.00 | 0.00 |
| 13 | BPH | IR22 | 30 | 25 | 1 | 5 | 1.00 | 5.50 | 50.00 |
| 13 | BPH | IR22 | 30 | 25 | 2 | 5 | 2.00 | 11.00 | 100.00 |
| 13 | BPH | IR22 | 30 | 25 | 3 | 3 | 0.00 | 0.00 | 0.00 |
| 13 | BPH | IR22 | 30 | 25 | 4 | 3 | 0.00 | 0.00 | 0.00 |
| 13 | BPH | IR22 | 30 | 30 | 1 | 5 | 0.00 | 0.00 | 0.00 |
| 13 | BPH | IR22 | 30 | 30 | 2 | 5 | 1.00 | 0.00 | 20.00 |
| 13 | BPH | IR22 | 30 | 30 | 3 | 3 | 0.00 | 0.00 | 0.00 |
| 13 | BPH | IR22 | 30 | 30 | 4 | 3 | 0.00 | 0.00 | 0.00 |
| 13 | BPH | IR22 | 30 | 35 | 1 | 5 | 0.00 | 0.00 | 0.00 |
| 13 | BPH | IR22 | 30 | 35 | 2 | 3 | 0.00 | 0.00 | 0.00 |
| 13 | BPH | IR22 | 30 | 35 | 3 | 3 | 0.00 | 0.00 | 0.00 |
| 13 | BPH | IR22 | 30 | 35 | 4 | 3 | 0.00 | 0.00 | 0.00 |
| 13 | BPH | T65 | 20 | 15 | 1 | 3 | 0.33 | 0.00 | 11.11 |
| 13 | BPH | T65 | 20 | 15 | 2 | 3 | 1.00 | 0.00 | 33.33 |
| 13 | BPH | T65 | 20 | 15 | 3 | 3 | 0.00 | 0.00 | 0.00 |
| 13 | BPH | T65 | 20 | 15 | 4 | 3 | 0.00 | 0.00 | 0.00 |
| 13 | BPH | T65 | 20 | 20 | 1 | 5 | 1.50 | 4.50 | 24.44 |
| 13 | BPH | T65 | 20 | 20 | 2 | 5 | 3.50 | 13.50 | 40.00 |
| 13 | BPH | T65 | 20 | 20 | 3 | 3 | 0.00 | 0.00 | 0.00 |
| 13 | BPH | T65 | 20 | 20 | 4 | 3 | 1.00 | 0.00 | 33.33 |
| 13 | BPH | T65 | 20 | 25 | 1 | 5 | 0.00 | 0.00 | 0.00 |
| 13 | BPH | T65 | 20 | 25 | 2 | 5 | 0.00 | 0.00 | 0.00 |
| 13 | BPH | T65 | 20 | 25 | 3 | 3 | 0.00 | 0.00 | 0.00 |
| 13 | BPH | T65 | 20 | 25 | 4 | 3 | 0.00 | 0.00 | 0.00 |
| 13 | BPH | T65 | 20 | 30 | 1 | 5 | 1.00 | 2.00 | 6.67 |
| 13 | BPH | T65 | 20 | 30 | 2 | 5 | 3.00 | 6.00 | 20.00 |
| 13 | BPH | T65 | 20 | 30 | 3 | 3 | 0.00 | 0.00 | 0.00 |
| 13 | BPH | T65 | 20 | 30 | 4 | 3 | 0.00 | 0.00 | 0.00 |
| 13 | BPH | T65 | 20 | 35 | 1 | 5 | 0.00 | 0.00 | 0.00 |
| 13 | BPH | T65 | 20 | 35 | 2 | 3 | 0.00 | 0.00 | 0.00 |
| 13 | BPH | T65 | 20 | 35 | 3 | 3 | 0.00 | 0.00 | 0.00 |
| 13 | BPH | T65 | 20 | 35 | 4 | 3 | 0.00 | 0.00 | 0.00 |
| 13 | BPH | T65 | 30 | 15 | 1 | 3 | 0.33 | 0.00 | 22.22 |
| 13 | BPH | T65 | 30 | 15 | 2 | 3 | 0.00 | 0.00 | 0.00 |
| 13 | BPH | T65 | 30 | 15 | 3 | 3 | 1.00 | 0.00 | 66.67 |
| 13 | BPH | T65 | 30 | 15 | 4 | 3 | 0.00 | 0.00 | 0.00 |
| 13 | BPH | T65 | 30 | 20 | 1 | 5 | 0.75 | 3.33 | 26.67 |
| 13 | BPH | T65 | 30 | 20 | 2 | 5 | 2.25 | 10.00 | 80.00 |
| 13 | BPH | T65 | 30 | 20 | 3 | 3 | 0.00 | 0.00 | 0.00 |
| 13 | BPH | T65 | 30 | 20 | 4 | 3 | 0.00 | 0.00 | 0.00 |
| 13 | BPH | T65 | 30 | 25 | 1 | 5 | 2.38 | 11.88 | 40.00 |
| 13 | BPH | T65 | 30 | 25 | 2 | 5 | 4.75 | 23.75 | 80.00 |
| 13 | BPH | T65 | 30 | 25 | 3 | 3 | 0.00 | 0.00 | 0.00 |
| 13 | BPH | T65 | 30 | 25 | 4 | 3 | 0.00 | 0.00 | 0.00 |
| 13 | BPH | T65 | 30 | 30 | 1 | 5 | 0.00 | 0.00 | 0.00 |
| 13 | BPH | T65 | 30 | 30 | 2 | 5 | 1.33 | 4.00 | 60.00 |
| 13 | BPH | T65 | 30 | 30 | 3 | 3 | 0.00 | 0.00 | 0.00 |
| 13 | BPH | T65 | 30 | 30 | 4 | 3 | 0.00 | 0.00 | 0.00 |
| 13 | BPH | T65 | 30 | 35 | 1 | 5 | 0.00 | 0.00 | 0.00 |
| 13 | BPH | T65 | 30 | 35 | 2 | 3 | 0.00 | 0.00 | 0.00 |
| 13 | BPH | T65 | 30 | 35 | 3 | 3 | 0.00 | 0.00 | 0.00 |
| 13 | BPH | T65 | 30 | 35 | 4 | 3 | 0.00 | 0.00 | 0.00 |
| 13 | WBPH | IR22 | 20 | 15 | 1 | 3 | 0.33 | 0.00 | 22.22 |
| 13 | WBPH | IR22 | 20 | 15 | 2 | 3 | 0.00 | 0.00 | 0.00 |
| 13 | WBPH | IR22 | 20 | 15 | 3 | 3 | 0.00 | 0.00 | 0.00 |
| 13 | WBPH | IR22 | 20 | 15 | 4 | 3 | 1.00 | 0.00 | 66.67 |
| 13 | WBPH | IR22 | 20 | 20 | 1 | 5 | 0.00 | 0.00 | 0.00 |
| 13 | WBPH | IR22 | 20 | 20 | 2 | 5 | 0.00 | 0.00 | 0.00 |
| 13 | WBPH | IR22 | 20 | 20 | 3 | 3 | 0.00 | 0.00 | 0.00 |
| 13 | WBPH | IR22 | 20 | 20 | 4 | 3 | 0.00 | 0.00 | 0.00 |
| 13 | WBPH | IR22 | 20 | 25 | 1 | 5 | 0.33 | 1.00 | 11.11 |
| 13 | WBPH | IR22 | 20 | 25 | 2 | 5 | 0.00 | 0.00 | 0.00 |
| 13 | WBPH | IR22 | 20 | 25 | 3 | 3 | 0.00 | 0.00 | 0.00 |
| 13 | WBPH | IR22 | 20 | 25 | 4 | 3 | 1.00 | 3.00 | 33.33 |
| 13 | WBPH | IR22 | 20 | 30 | 1 | 5 | 0.00 | 0.00 | 0.00 |
| 13 | WBPH | IR22 | 20 | 30 | 2 | 5 | 0.00 | 0.00 | 0.00 |
| 13 | WBPH | IR22 | 20 | 30 | 3 | 3 | 0.00 | 0.00 | 0.00 |
| 13 | WBPH | IR22 | 20 | 30 | 4 | 3 | 0.00 | 0.00 | 0.00 |
| 13 | WBPH | IR22 | 20 | 35 | 1 | 5 | 0.00 | 0.00 | 0.00 |
| 13 | WBPH | IR22 | 20 | 35 | 2 | 3 | 0.00 | 0.00 | 0.00 |
| 13 | WBPH | IR22 | 20 | 35 | 3 | 3 | 0.00 | 0.00 | 0.00 |
| 13 | WBPH | IR22 | 20 | 35 | 4 | 3 | 0.00 | 0.00 | 0.00 |
| 13 | WBPH | IR22 | 30 | 15 | 1 | 1 | 0.00 | 0.00 | 33.00 |
| 13 | WBPH | IR22 | 30 | 15 | 2 | 1 | 0.00 | 0.00 | 67.00 |
| 13 | WBPH | IR22 | 30 | 15 | 3 | 1 | 0.00 | 0.00 | 67.00 |
| 13 | WBPH | IR22 | 30 | 15 | 4 | 1 | 0.00 | 0.00 | 0.00 |
| 13 | WBPH | IR22 | 30 | 20 | 1 | 5 | 0.00 | 0.00 | 0.00 |
| 13 | WBPH | IR22 | 30 | 20 | 2 | 5 | 0.00 | 0.00 | 0.00 |
| 13 | WBPH | IR22 | 30 | 20 | 3 | 3 | 0.00 | 0.00 | 0.00 |
| 13 | WBPH | IR22 | 30 | 20 | 4 | 3 | 1.00 | 0.00 | 33.33 |
| 13 | WBPH | IR22 | 30 | 25 | 1 | 5 | 0.00 | 0.00 | 0.00 |
| 13 | WBPH | IR22 | 30 | 25 | 2 | 5 | 0.00 | 0.00 | 0.00 |
| 13 | WBPH | IR22 | 30 | 25 | 3 | 5 | 0.00 | 0.00 | 0.00 |
| 13 | WBPH | IR22 | 30 | 25 | 4 | 5 | 0.00 | 0.00 | 0.00 |
| 13 | WBPH | IR22 | 30 | 30 | 1 | 5 | 0.00 | 0.00 | 0.00 |
| 13 | WBPH | IR22 | 30 | 30 | 2 | 5 | 0.00 | 0.00 | 0.00 |
| 13 | WBPH | IR22 | 30 | 30 | 3 | 5 | 0.00 | 0.00 | 0.00 |
| 13 | WBPH | IR22 | 30 | 30 | 4 | 3 | 0.00 | 0.00 | 0.00 |
| 13 | WBPH | IR22 | 30 | 35 | 1 | 5 | 0.00 | 0.00 | 0.00 |
| 13 | WBPH | IR22 | 30 | 35 | 2 | 3 | 0.00 | 0.00 | 0.00 |
| 13 | WBPH | IR22 | 30 | 35 | 3 | 3 | 0.00 | 0.00 | 0.00 |
| 13 | WBPH | IR22 | 30 | 35 | 4 | 3 | 0.00 | 0.00 | 0.00 |
| 13 | WBPH | T65 | 20 | 15 | 1 | 3 | 1.17 | 2.50 | 55.56 |
| 13 | WBPH | T65 | 20 | 15 | 2 | 3 | 1.50 | 3.50 | 66.67 |
| 13 | WBPH | T65 | 20 | 15 | 3 | 3 | 1.00 | 0.00 | 33.33 |
| 13 | WBPH | T65 | 20 | 15 | 4 | 3 | 1.00 | 4.00 | 66.67 |
| 13 | WBPH | T65 | 20 | 20 | 1 | 5 | 0.67 | 0.00 | 22.22 |
| 13 | WBPH | T65 | 20 | 20 | 2 | 5 | 0.00 | 0.00 | 0.00 |
| 13 | WBPH | T65 | 20 | 20 | 3 | 3 | 1.00 | 0.00 | 33.33 |
| 13 | WBPH | T65 | 20 | 20 | 4 | 3 | 1.00 | 0.00 | 33.33 |
| 13 | WBPH | T65 | 20 | 25 | 1 | 5 | 0.33 | 1.50 | 22.22 |
| 13 | WBPH | T65 | 20 | 25 | 2 | 5 | 0.00 | 0.00 | 0.00 |
| 13 | WBPH | T65 | 20 | 25 | 3 | 3 | 0.00 | 0.00 | 0.00 |
| 13 | WBPH | T65 | 20 | 25 | 4 | 3 | 1.00 | 4.50 | 66.67 |
| 13 | WBPH | T65 | 20 | 30 | 1 | 5 | 0.00 | 0.00 | 0.00 |
| 13 | WBPH | T65 | 20 | 30 | 2 | 5 | 0.00 | 0.00 | 0.00 |
| 13 | WBPH | T65 | 20 | 30 | 3 | 3 | 0.00 | 0.00 | 0.00 |
| 13 | WBPH | T65 | 20 | 30 | 4 | 3 | 0.00 | 0.00 | 0.00 |
| 13 | WBPH | T65 | 20 | 35 | 1 | 5 | 0.00 | 0.00 | 0.00 |
| 13 | WBPH | T65 | 20 | 35 | 2 | 3 | 0.00 | 0.00 | 0.00 |
| 13 | WBPH | T65 | 20 | 35 | 3 | 3 | 0.00 | 0.00 | 0.00 |
| 13 | WBPH | T65 | 20 | 35 | 4 | 3 | 0.00 | 0.00 | 0.00 |
| 13 | WBPH | T65 | 30 | 15 | 1 | 1 | 0.00 | 0.00 | 0.00 |
| 13 | WBPH | T65 | 30 | 15 | 2 | 1 | 0.00 | 0.00 | 33.00 |
| 13 | WBPH | T65 | 30 | 15 | 3 | 1 | 0.00 | 0.00 | 80.00 |
| 13 | WBPH | T65 | 30 | 15 | 4 | 1 | 0.00 | 0.00 | 33.00 |
| 13 | WBPH | T65 | 30 | 20 | 1 | 5 | 0.00 | 0.00 | 0.00 |
| 13 | WBPH | T65 | 30 | 20 | 2 | 5 | 0.00 | 0.00 | 0.00 |
| 13 | WBPH | T65 | 30 | 20 | 3 | 3 | 0.00 | 0.00 | 0.00 |
| 13 | WBPH | T65 | 30 | 20 | 4 | 3 | 0.00 | 0.00 | 0.00 |
| 13 | WBPH | T65 | 30 | 25 | 1 | 5 | 0.00 | 0.00 | 0.00 |
| 13 | WBPH | T65 | 30 | 25 | 2 | 5 | 0.00 | 0.00 | 0.00 |
| 13 | WBPH | T65 | 30 | 25 | 3 | 5 | 0.00 | 0.00 | 0.00 |
| 13 | WBPH | T65 | 30 | 25 | 4 | 5 | 0.00 | 0.00 | 0.00 |
| 13 | WBPH | T65 | 30 | 30 | 1 | 5 | 0.00 | 0.00 | 0.00 |
| 13 | WBPH | T65 | 30 | 30 | 2 | 5 | 0.00 | 0.00 | 0.00 |
| 13 | WBPH | T65 | 30 | 30 | 3 | 3 | 0.00 | 0.00 | 0.00 |
| 13 | WBPH | T65 | 30 | 30 | 4 | 3 | 0.00 | 0.00 | 0.00 |
| 13 | WBPH | T65 | 30 | 35 | 1 | 5 | 0.00 | 0.00 | 0.00 |
| 13 | WBPH | T65 | 30 | 35 | 2 | 3 | 0.00 | 0.00 | 0.00 |
| 13 | WBPH | T65 | 30 | 35 | 3 | 3 | 0.00 | 0.00 | 0.00 |
| 13 | WBPH | T65 | 30 | 35 | 4 | 3 | 0.00 | 0.00 | 0.00 |
| 14 | BPH | IR22 | 20 | 15 | 1 | 3 | 0.67 | 3.33 | 22.22 |
| 14 | BPH | IR22 | 20 | 15 | 2 | 3 | 1.00 | 0.00 | 33.33 |
| 14 | BPH | IR22 | 20 | 15 | 3 | 3 | 0.00 | 0.00 | 0.00 |
| 14 | BPH | IR22 | 20 | 15 | 4 | 3 | 1.00 | 10.00 | 33.33 |
| 14 | BPH | IR22 | 20 | 20 | 1 | 5 | 0.67 | 1.11 | 31.11 |
| 14 | BPH | IR22 | 20 | 20 | 2 | 5 | 1.00 | 3.33 | 60.00 |
| 14 | BPH | IR22 | 20 | 20 | 3 | 3 | 1.00 | 0.00 | 33.33 |
| 14 | BPH | IR22 | 20 | 20 | 4 | 3 | 0.00 | 0.00 | 0.00 |
| 14 | BPH | IR22 | 20 | 25 | 1 | 5 | 1.50 | 7.50 | 20.00 |
| 14 | BPH | IR22 | 20 | 25 | 2 | 5 | 3.00 | 15.00 | 40.00 |
| 14 | BPH | IR22 | 20 | 25 | 3 | 3 | 1.50 | 7.50 | 20.00 |
| 14 | BPH | IR22 | 20 | 25 | 4 | 3 | 0.00 | 0.00 | 0.00 |
| 14 | BPH | IR22 | 20 | 30 | 1 | 5 | 0.00 | 0.00 | 40.00 |
| 14 | BPH | IR22 | 20 | 30 | 2 | 5 | 0.00 | 0.00 | 40.00 |
| 14 | BPH | IR22 | 20 | 30 | 3 | 5 | 0.00 | 0.00 | 0.00 |
| 14 | BPH | IR22 | 20 | 30 | 4 | 3 | 0.00 | 0.00 | 0.00 |
| 14 | BPH | IR22 | 20 | 35 | 1 | 5 | 0.00 | 0.00 | 0.00 |
| 14 | BPH | IR22 | 20 | 35 | 2 | 3 | 0.00 | 0.00 | 0.00 |
| 14 | BPH | IR22 | 20 | 35 | 3 | 3 | 0.00 | 0.00 | 0.00 |
| 14 | BPH | IR22 | 20 | 35 | 4 | 3 | 0.00 | 0.00 | 0.00 |
| 14 | BPH | IR22 | 30 | 15 | 1 | 1 | 0.33 | 0.00 | 22.22 |
| 14 | BPH | IR22 | 30 | 15 | 2 | 1 | 0.00 | 0.00 | 0.00 |
| 14 | BPH | IR22 | 30 | 15 | 3 | 3 | 1.00 | 0.00 | 66.67 |
| 14 | BPH | IR22 | 30 | 15 | 4 | 3 | 0.00 | 0.00 | 0.00 |
| 14 | BPH | IR22 | 30 | 20 | 1 | 5 | 0.44 | 1.56 | 20.00 |
| 14 | BPH | IR22 | 30 | 20 | 2 | 5 | 1.33 | 4.67 | 60.00 |
| 14 | BPH | IR22 | 30 | 20 | 3 | 3 | 0.00 | 0.00 | 0.00 |
| 14 | BPH | IR22 | 30 | 20 | 4 | 3 | 0.00 | 0.00 | 0.00 |
| 14 | BPH | IR22 | 30 | 25 | 1 | 5 | 0.00 | 0.00 | 0.00 |
| 14 | BPH | IR22 | 30 | 25 | 2 | 5 | 1.25 | 7.00 | 80.00 |
| 14 | BPH | IR22 | 30 | 25 | 3 | 5 | 0.00 | 0.00 | 0.00 |
| 14 | BPH | IR22 | 30 | 25 | 4 | 5 | 0.00 | 0.00 | 0.00 |
| 14 | BPH | IR22 | 30 | 30 | 1 | 5 | 0.00 | 0.00 | 0.00 |
| 14 | BPH | IR22 | 30 | 30 | 2 | 5 | 1.00 | 0.00 | 20.00 |
| 14 | BPH | IR22 | 30 | 30 | 3 | 5 | 0.00 | 0.00 | 0.00 |
| 14 | BPH | IR22 | 30 | 30 | 4 | 5 | 0.00 | 0.00 | 0.00 |
| 14 | BPH | IR22 | 30 | 35 | 1 | 5 | 0.00 | 0.00 | 0.00 |
| 14 | BPH | IR22 | 30 | 35 | 2 | 3 | 0.00 | 0.00 | 0.00 |
| 14 | BPH | IR22 | 30 | 35 | 3 | 3 | 0.00 | 0.00 | 0.00 |
| 14 | BPH | IR22 | 30 | 35 | 4 | 3 | 0.00 | 0.00 | 0.00 |
| 14 | BPH | T65 | 20 | 15 | 1 | 3 | 0.00 | 0.00 | 0.00 |
| 14 | BPH | T65 | 20 | 15 | 2 | 3 | 1.00 | 0.00 | 33.33 |
| 14 | BPH | T65 | 20 | 15 | 3 | 3 | 0.00 | 0.00 | 0.00 |
| 14 | BPH | T65 | 20 | 15 | 4 | 3 | 0.00 | 0.00 | 0.00 |
| 14 | BPH | T65 | 20 | 20 | 1 | 5 | 1.50 | 5.67 | 13.33 |
| 14 | BPH | T65 | 20 | 20 | 2 | 5 | 4.50 | 17.00 | 40.00 |
| 14 | BPH | T65 | 20 | 20 | 3 | 3 | 0.00 | 0.00 | 0.00 |
| 14 | BPH | T65 | 20 | 20 | 4 | 3 | 0.00 | 0.00 | 0.00 |
| 14 | BPH | T65 | 20 | 25 | 1 | 5 | 0.00 | 0.00 | 0.00 |
| 14 | BPH | T65 | 20 | 25 | 2 | 5 | 0.00 | 0.00 | 0.00 |
| 14 | BPH | T65 | 20 | 25 | 3 | 3 | 0.00 | 0.00 | 0.00 |
| 14 | BPH | T65 | 20 | 25 | 4 | 3 | 0.00 | 0.00 | 0.00 |
| 14 | BPH | T65 | 20 | 30 | 1 | 5 | 0.00 | 0.00 | 0.00 |
| 14 | BPH | T65 | 20 | 30 | 2 | 5 | 0.00 | 0.00 | 0.00 |
| 14 | BPH | T65 | 20 | 30 | 3 | 3 | 0.00 | 0.00 | 0.00 |
| 14 | BPH | T65 | 20 | 30 | 4 | 3 | 0.00 | 0.00 | 0.00 |
| 14 | BPH | T65 | 20 | 35 | 1 | 5 | 0.00 | 0.00 | 0.00 |
| 14 | BPH | T65 | 20 | 35 | 2 | 3 | 0.00 | 0.00 | 0.00 |
| 14 | BPH | T65 | 20 | 35 | 3 | 3 | 0.00 | 0.00 | 0.00 |
| 14 | BPH | T65 | 20 | 35 | 4 | 3 | 0.00 | 0.00 | 0.00 |
| 14 | BPH | T65 | 30 | 15 | 1 | 3 | 1.67 | 6.33 | 22.22 |
| 14 | BPH | T65 | 30 | 15 | 2 | 3 | 0.00 | 0.00 | 0.00 |
| 14 | BPH | T65 | 30 | 15 | 3 | 3 | 5.00 | 19.00 | 66.67 |
| 14 | BPH | T65 | 30 | 15 | 4 | 3 | 0.00 | 0.00 | 0.00 |
| 14 | BPH | T65 | 30 | 20 | 1 | 5 | 0.58 | 2.17 | 26.67 |
| 14 | BPH | T65 | 30 | 20 | 2 | 5 | 1.75 | 6.50 | 80.00 |
| 14 | BPH | T65 | 30 | 20 | 3 | 3 | 0.00 | 0.00 | 0.00 |
| 14 | BPH | T65 | 30 | 20 | 4 | 3 | 0.00 | 0.00 | 0.00 |
| 14 | BPH | T65 | 30 | 25 | 1 | 5 | 0.00 | 0.00 | 0.00 |
| 14 | BPH | T65 | 30 | 25 | 2 | 5 | 4.50 | 21.25 | 80.00 |
| 14 | BPH | T65 | 30 | 25 | 3 | 5 | 0.00 | 0.00 | 0.00 |
| 14 | BPH | T65 | 30 | 25 | 4 | 5 | 0.00 | 0.00 | 0.00 |
| 14 | BPH | T65 | 30 | 30 | 1 | 5 | 0.00 | 0.00 | 0.00 |
| 14 | BPH | T65 | 30 | 30 | 2 | 5 | 4.00 | 15.00 | 40.00 |
| 14 | BPH | T65 | 30 | 30 | 3 | 5 | 0.00 | 0.00 | 0.00 |
| 14 | BPH | T65 | 30 | 30 | 4 | 5 | 0.00 | 0.00 | 0.00 |
| 14 | BPH | T65 | 30 | 35 | 1 | 5 | 0.00 | 0.00 | 0.00 |
| 14 | BPH | T65 | 30 | 35 | 2 | 3 | 0.00 | 0.00 | 0.00 |
| 14 | BPH | T65 | 30 | 35 | 3 | 3 | 0.00 | 0.00 | 0.00 |
| 14 | BPH | T65 | 30 | 35 | 4 | 3 | 0.00 | 0.00 | 0.00 |
| 14 | WBPH | IR22 | 20 | 15 | 1 | 3 | 0.33 | 0.00 | 11.11 |
| 14 | WBPH | IR22 | 20 | 15 | 2 | 3 | 0.00 | 0.00 | 0.00 |
| 14 | WBPH | IR22 | 20 | 15 | 3 | 3 | 0.00 | 0.00 | 0.00 |
| 14 | WBPH | IR22 | 20 | 15 | 4 | 3 | 1.00 | 0.00 | 33.33 |
| 14 | WBPH | IR22 | 20 | 20 | 1 | 5 | 0.00 | 0.00 | 0.00 |
| 14 | WBPH | IR22 | 20 | 20 | 2 | 5 | 0.00 | 0.00 | 0.00 |
| 14 | WBPH | IR22 | 20 | 20 | 3 | 3 | 0.00 | 0.00 | 0.00 |
| 14 | WBPH | IR22 | 20 | 20 | 4 | 3 | 0.00 | 0.00 | 0.00 |
| 14 | WBPH | IR22 | 20 | 25 | 1 | 5 | 0.00 | 0.00 | 0.00 |
| 14 | WBPH | IR22 | 20 | 25 | 2 | 5 | 0.00 | 0.00 | 0.00 |
| 14 | WBPH | IR22 | 20 | 25 | 3 | 3 | 0.00 | 0.00 | 0.00 |
| 14 | WBPH | IR22 | 20 | 25 | 4 | 3 | 1.00 | 0.00 | 33.33 |
| 14 | WBPH | IR22 | 20 | 30 | 1 | 5 | 0.00 | 0.00 | 0.00 |
| 14 | WBPH | IR22 | 20 | 30 | 2 | 5 | 0.00 | 0.00 | 0.00 |
| 14 | WBPH | IR22 | 20 | 30 | 3 | 3 | 0.00 | 0.00 | 0.00 |
| 14 | WBPH | IR22 | 20 | 30 | 4 | 3 | 0.00 | 0.00 | 0.00 |
| 14 | WBPH | IR22 | 20 | 35 | 1 | 5 | 0.00 | 0.00 | 0.00 |
| 14 | WBPH | IR22 | 20 | 35 | 2 | 3 | 0.00 | 0.00 | 0.00 |
| 14 | WBPH | IR22 | 20 | 35 | 3 | 3 | 0.00 | 0.00 | 0.00 |
| 14 | WBPH | IR22 | 20 | 35 | 4 | 3 | 0.00 | 0.00 | 0.00 |
| 14 | WBPH | IR22 | 30 | 15 | 1 | 1 | 0.00 | 0.00 | 33.00 |
| 14 | WBPH | IR22 | 30 | 15 | 2 | 1 | 0.00 | 0.00 | 67.00 |
| 14 | WBPH | IR22 | 30 | 15 | 3 | 1 | 0.00 | 0.00 | 33.00 |
| 14 | WBPH | IR22 | 30 | 15 | 4 | 1 | 0.00 | 0.00 | 0.00 |
| 14 | WBPH | IR22 | 30 | 20 | 1 | 5 | 0.00 | 0.00 | 0.00 |
| 14 | WBPH | IR22 | 30 | 20 | 2 | 5 | 0.00 | 0.00 | 0.00 |
| 14 | WBPH | IR22 | 30 | 20 | 3 | 3 | 0.00 | 0.00 | 0.00 |
| 14 | WBPH | IR22 | 30 | 20 | 4 | 3 | 0.00 | 0.00 | 0.00 |
| 14 | WBPH | IR22 | 30 | 25 | 1 | 5 | 0.00 | 0.00 | 0.00 |
| 14 | WBPH | IR22 | 30 | 25 | 2 | 5 | 0.00 | 0.00 | 0.00 |
| 14 | WBPH | IR22 | 30 | 25 | 3 | 5 | 0.00 | 0.00 | 0.00 |
| 14 | WBPH | IR22 | 30 | 25 | 4 | 5 | 0.00 | 0.00 | 0.00 |
| 14 | WBPH | IR22 | 30 | 30 | 1 | 5 | 0.00 | 0.00 | 0.00 |
| 14 | WBPH | IR22 | 30 | 30 | 2 | 5 | 0.00 | 0.00 | 0.00 |
| 14 | WBPH | IR22 | 30 | 30 | 3 | 5 | 0.00 | 0.00 | 0.00 |
| 14 | WBPH | IR22 | 30 | 30 | 4 | 5 | 0.00 | 0.00 | 0.00 |
| 14 | WBPH | IR22 | 30 | 35 | 1 | 5 | 0.00 | 0.00 | 0.00 |
| 14 | WBPH | IR22 | 30 | 35 | 2 | 3 | 0.00 | 0.00 | 0.00 |
| 14 | WBPH | IR22 | 30 | 35 | 3 | 3 | 0.00 | 0.00 | 0.00 |
| 14 | WBPH | IR22 | 30 | 35 | 4 | 3 | 0.00 | 0.00 | 0.00 |
| 14 | WBPH | T65 | 20 | 15 | 1 | 3 | 1.00 | 1.00 | 44.44 |
| 14 | WBPH | T65 | 20 | 15 | 2 | 3 | 1.00 | 0.00 | 33.33 |
| 14 | WBPH | T65 | 20 | 15 | 3 | 3 | 1.00 | 2.00 | 33.33 |
| 14 | WBPH | T65 | 20 | 15 | 4 | 3 | 1.00 | 1.00 | 66.67 |
| 14 | WBPH | T65 | 20 | 20 | 1 | 5 | 1.00 | 3.00 | 22.22 |
| 14 | WBPH | T65 | 20 | 20 | 2 | 5 | 0.00 | 0.00 | 0.00 |
| 14 | WBPH | T65 | 20 | 20 | 3 | 3 | 1.00 | 0.00 | 33.33 |
| 14 | WBPH | T65 | 20 | 20 | 4 | 3 | 2.00 | 9.00 | 33.33 |
| 14 | WBPH | T65 | 20 | 25 | 1 | 5 | 0.50 | 0.00 | 16.67 |
| 14 | WBPH | T65 | 20 | 25 | 2 | 5 | 0.00 | 0.00 | 0.00 |
| 14 | WBPH | T65 | 20 | 25 | 3 | 3 | 0.00 | 0.00 | 0.00 |
| 14 | WBPH | T65 | 20 | 25 | 4 | 3 | 1.00 | 0.00 | 33.33 |
| 14 | WBPH | T65 | 20 | 30 | 1 | 5 | 0.00 | 0.00 | 0.00 |
| 14 | WBPH | T65 | 20 | 30 | 2 | 5 | 0.00 | 0.00 | 0.00 |
| 14 | WBPH | T65 | 20 | 30 | 3 | 3 | 0.00 | 0.00 | 0.00 |
| 14 | WBPH | T65 | 20 | 30 | 4 | 3 | 0.00 | 0.00 | 0.00 |
| 14 | WBPH | T65 | 20 | 35 | 1 | 5 | 0.00 | 0.00 | 0.00 |
| 14 | WBPH | T65 | 20 | 35 | 2 | 3 | 0.00 | 0.00 | 0.00 |
| 14 | WBPH | T65 | 20 | 35 | 3 | 3 | 0.00 | 0.00 | 0.00 |
| 14 | WBPH | T65 | 20 | 35 | 4 | 3 | 0.00 | 0.00 | 0.00 |
| 14 | WBPH | T65 | 30 | 15 | 1 | 1 | 0.00 | 0.00 | 0.00 |
| 14 | WBPH | T65 | 30 | 15 | 2 | 1 | 0.00 | 0.00 | 10.00 |
| 14 | WBPH | T65 | 30 | 15 | 3 | 1 | 0.00 | 0.00 | 80.00 |
| 14 | WBPH | T65 | 30 | 15 | 4 | 1 | 0.00 | 0.00 | 33.00 |
| 14 | WBPH | T65 | 30 | 20 | 1 | 5 | 0.00 | 0.00 | 0.00 |
| 14 | WBPH | T65 | 30 | 20 | 2 | 5 | 0.00 | 0.00 | 0.00 |
| 14 | WBPH | T65 | 30 | 20 | 3 | 3 | 0.00 | 0.00 | 0.00 |
| 14 | WBPH | T65 | 30 | 20 | 4 | 3 | 0.00 | 0.00 | 0.00 |
| 14 | WBPH | T65 | 30 | 25 | 1 | 5 | 0.00 | 0.00 | 0.00 |
| 14 | WBPH | T65 | 30 | 25 | 2 | 5 | 0.00 | 0.00 | 0.00 |
| 14 | WBPH | T65 | 30 | 25 | 3 | 5 | 0.00 | 0.00 | 0.00 |
| 14 | WBPH | T65 | 30 | 25 | 4 | 5 | 0.00 | 0.00 | 0.00 |
| 14 | WBPH | T65 | 30 | 30 | 1 | 5 | 0.00 | 0.00 | 0.00 |
| 14 | WBPH | T65 | 30 | 30 | 2 | 5 | 0.00 | 0.00 | 0.00 |
| 14 | WBPH | T65 | 30 | 30 | 3 | 5 | 0.00 | 0.00 | 0.00 |
| 14 | WBPH | T65 | 30 | 30 | 4 | 5 | 0.00 | 0.00 | 0.00 |
| 14 | WBPH | T65 | 30 | 35 | 1 | 5 | 0.00 | 0.00 | 0.00 |
| 14 | WBPH | T65 | 30 | 35 | 2 | 3 | 0.00 | 0.00 | 0.00 |
| 14 | WBPH | T65 | 30 | 35 | 3 | 3 | 0.00 | 0.00 | 0.00 |
| 14 | WBPH | T65 | 30 | 35 | 4 | 3 | 0.00 | 0.00 | 0.00 |
| 15 | BPH | IR22 | 20 | 15 | 1 | 3 | 0.67 | 0.00 | 22.22 |
| 15 | BPH | IR22 | 20 | 15 | 2 | 3 | 1.00 | 0.00 | 33.33 |
| 15 | BPH | IR22 | 20 | 15 | 3 | 3 | 0.00 | 0.00 | 0.00 |
| 15 | BPH | IR22 | 20 | 15 | 4 | 3 | 1.00 | 0.00 | 33.33 |
| 15 | BPH | IR22 | 20 | 20 | 1 | 5 | 1.33 | 7.67 | 24.44 |
| 15 | BPH | IR22 | 20 | 20 | 2 | 5 | 1.00 | 3.00 | 40.00 |
| 15 | BPH | IR22 | 20 | 20 | 3 | 3 | 3.00 | 20.00 | 33.33 |
| 15 | BPH | IR22 | 20 | 20 | 4 | 3 | 0.00 | 0.00 | 0.00 |
| 15 | BPH | IR22 | 20 | 25 | 1 | 5 | 1.00 | 5.25 | 20.00 |
| 15 | BPH | IR22 | 20 | 25 | 2 | 5 | 2.00 | 10.50 | 40.00 |
| 15 | BPH | IR22 | 20 | 25 | 3 | 3 | 0.00 | 0.00 | 0.00 |
| 15 | BPH | IR22 | 20 | 25 | 4 | 3 | 0.00 | 0.00 | 0.00 |
| 15 | BPH | IR22 | 20 | 30 | 1 | 5 | 4.00 | 17.00 | 0.00 |
| 15 | BPH | IR22 | 20 | 30 | 2 | 5 | 4.00 | 17.00 | 20.00 |
| 15 | BPH | IR22 | 20 | 30 | 3 | 5 | 0.00 | 0.00 | 0.00 |
| 15 | BPH | IR22 | 20 | 30 | 4 | 3 | 0.00 | 0.00 | 0.00 |
| 15 | BPH | IR22 | 20 | 35 | 1 | 5 | 0.00 | 0.00 | 0.00 |
| 15 | BPH | IR22 | 20 | 35 | 2 | 3 | 0.00 | 0.00 | 0.00 |
| 15 | BPH | IR22 | 20 | 35 | 3 | 3 | 0.00 | 0.00 | 0.00 |
| 15 | BPH | IR22 | 20 | 35 | 4 | 3 | 0.00 | 0.00 | 0.00 |
| 15 | BPH | IR22 | 30 | 15 | 1 | 3 | 0.00 | 0.00 | 0.00 |
| 15 | BPH | IR22 | 30 | 15 | 2 | 3 | 0.00 | 0.00 | 0.00 |
| 15 | BPH | IR22 | 30 | 15 | 3 | 3 | 1.50 | 3.00 | 66.67 |
| 15 | BPH | IR22 | 30 | 15 | 4 | 3 | 0.00 | 0.00 | 0.00 |
| 15 | BPH | IR22 | 30 | 20 | 1 | 5 | 0.00 | 0.00 | 0.00 |
| 15 | BPH | IR22 | 30 | 20 | 2 | 5 | 2.33 | 6.00 | 60.00 |
| 15 | BPH | IR22 | 30 | 20 | 3 | 5 | 0.00 | 0.00 | 0.00 |
| 15 | BPH | IR22 | 30 | 20 | 4 | 5 | 0.00 | 0.00 | 0.00 |
| 15 | BPH | IR22 | 30 | 25 | 1 | 5 | 0.00 | 0.00 | 0.00 |
| 15 | BPH | IR22 | 30 | 25 | 2 | 5 | 1.33 | 7.33 | 60.00 |
| 15 | BPH | IR22 | 30 | 25 | 3 | 5 | 0.00 | 0.00 | 0.00 |
| 15 | BPH | IR22 | 30 | 25 | 4 | 5 | 0.00 | 0.00 | 0.00 |
| 15 | BPH | IR22 | 30 | 30 | 1 | 5 | 0.00 | 0.00 | 0.00 |
| 15 | BPH | IR22 | 30 | 30 | 2 | 5 | 0.00 | 0.00 | 0.00 |
| 15 | BPH | IR22 | 30 | 30 | 3 | 5 | 0.00 | 0.00 | 0.00 |
| 15 | BPH | IR22 | 30 | 30 | 4 | 5 | 0.00 | 0.00 | 0.00 |
| 15 | BPH | IR22 | 30 | 35 | 1 | 5 | 0.00 | 0.00 | 0.00 |
| 15 | BPH | IR22 | 30 | 35 | 2 | 3 | 0.00 | 0.00 | 0.00 |
| 15 | BPH | IR22 | 30 | 35 | 3 | 3 | 0.00 | 0.00 | 0.00 |
| 15 | BPH | IR22 | 30 | 35 | 4 | 3 | 0.00 | 0.00 | 0.00 |
| 15 | BPH | T65 | 20 | 15 | 1 | 3 | 0.00 | 0.00 | 0.00 |
| 15 | BPH | T65 | 20 | 15 | 2 | 3 | 0.00 | 0.00 | 0.00 |
| 15 | BPH | T65 | 20 | 15 | 3 | 3 | 0.00 | 0.00 | 0.00 |
| 15 | BPH | T65 | 20 | 15 | 4 | 3 | 0.00 | 0.00 | 0.00 |
| 15 | BPH | T65 | 20 | 20 | 1 | 5 | 1.17 | 6.83 | 20.00 |
| 15 | BPH | T65 | 20 | 20 | 2 | 5 | 3.50 | 20.50 | 60.00 |
| 15 | BPH | T65 | 20 | 20 | 3 | 3 | 0.00 | 0.00 | 0.00 |
| 15 | BPH | T65 | 20 | 20 | 4 | 3 | 0.00 | 0.00 | 0.00 |
| 15 | BPH | T65 | 20 | 25 | 1 | 5 | 0.00 | 0.00 | 0.00 |
| 15 | BPH | T65 | 20 | 25 | 2 | 5 | 0.00 | 0.00 | 0.00 |
| 15 | BPH | T65 | 20 | 25 | 3 | 3 | 0.00 | 0.00 | 0.00 |
| 15 | BPH | T65 | 20 | 25 | 4 | 3 | 0.00 | 0.00 | 0.00 |
| 15 | BPH | T65 | 20 | 30 | 1 | 5 | 0.00 | 0.00 | 0.00 |
| 15 | BPH | T65 | 20 | 30 | 2 | 5 | 0.00 | 0.00 | 0.00 |
| 15 | BPH | T65 | 20 | 30 | 3 | 3 | 0.00 | 0.00 | 0.00 |
| 15 | BPH | T65 | 20 | 30 | 4 | 3 | 0.00 | 0.00 | 0.00 |
| 15 | BPH | T65 | 20 | 35 | 1 | 5 | 0.00 | 0.00 | 0.00 |
| 15 | BPH | T65 | 20 | 35 | 2 | 3 | 0.00 | 0.00 | 0.00 |
| 15 | BPH | T65 | 20 | 35 | 3 | 3 | 0.00 | 0.00 | 0.00 |
| 15 | BPH | T65 | 20 | 35 | 4 | 3 | 0.00 | 0.00 | 0.00 |
| 15 | BPH | T65 | 30 | 15 | 1 | 3 | 0.00 | 0.00 | 0.00 |
| 15 | BPH | T65 | 30 | 15 | 2 | 3 | 0.00 | 0.00 | 0.00 |
| 15 | BPH | T65 | 30 | 15 | 3 | 3 | 1.00 | 0.00 | 66.67 |
| 15 | BPH | T65 | 30 | 15 | 4 | 3 | 0.00 | 0.00 | 0.00 |
| 15 | BPH | T65 | 30 | 20 | 1 | 5 | 0.00 | 0.00 | 0.00 |
| 15 | BPH | T65 | 30 | 20 | 2 | 5 | 1.75 | 4.00 | 80.00 |
| 15 | BPH | T65 | 30 | 20 | 3 | 5 | 0.00 | 0.00 | 0.00 |
| 15 | BPH | T65 | 30 | 20 | 4 | 5 | 0.00 | 0.00 | 0.00 |
| 15 | BPH | T65 | 30 | 25 | 1 | 5 | 0.00 | 0.00 | 0.00 |
| 15 | BPH | T65 | 30 | 25 | 2 | 5 | 1.50 | 4.25 | 80.00 |
| 15 | BPH | T65 | 30 | 25 | 3 | 5 | 0.00 | 0.00 | 0.00 |
| 15 | BPH | T65 | 30 | 25 | 4 | 5 | 0.00 | 0.00 | 0.00 |
| 15 | BPH | T65 | 30 | 30 | 1 | 5 | 0.00 | 0.00 | 0.00 |
| 15 | BPH | T65 | 30 | 30 | 2 | 5 | 1.50 | 5.00 | 40.00 |
| 15 | BPH | T65 | 30 | 30 | 3 | 5 | 0.00 | 0.00 | 0.00 |
| 15 | BPH | T65 | 30 | 30 | 4 | 5 | 0.00 | 0.00 | 0.00 |
| 15 | BPH | T65 | 30 | 35 | 1 | 5 | 0.00 | 0.00 | 0.00 |
| 15 | BPH | T65 | 30 | 35 | 2 | 3 | 0.00 | 0.00 | 0.00 |
| 15 | BPH | T65 | 30 | 35 | 3 | 3 | 0.00 | 0.00 | 0.00 |
| 15 | BPH | T65 | 30 | 35 | 4 | 3 | 0.00 | 0.00 | 0.00 |
| 15 | WBPH | IR22 | 20 | 15 | 1 | 3 | 0.33 | 0.00 | 11.11 |
| 15 | WBPH | IR22 | 20 | 15 | 2 | 3 | 0.00 | 0.00 | 0.00 |
| 15 | WBPH | IR22 | 20 | 15 | 3 | 3 | 0.00 | 0.00 | 0.00 |
| 15 | WBPH | IR22 | 20 | 15 | 4 | 3 | 1.00 | 0.00 | 33.33 |
| 15 | WBPH | IR22 | 20 | 20 | 1 | 5 | 0.00 | 0.00 | 0.00 |
| 15 | WBPH | IR22 | 20 | 20 | 2 | 5 | 0.00 | 0.00 | 0.00 |
| 15 | WBPH | IR22 | 20 | 20 | 3 | 3 | 0.00 | 0.00 | 0.00 |
| 15 | WBPH | IR22 | 20 | 20 | 4 | 3 | 0.00 | 0.00 | 0.00 |
| 15 | WBPH | IR22 | 20 | 25 | 1 | 5 | 0.00 | 0.00 | 0.00 |
| 15 | WBPH | IR22 | 20 | 25 | 2 | 5 | 0.00 | 0.00 | 0.00 |
| 15 | WBPH | IR22 | 20 | 25 | 3 | 3 | 0.00 | 0.00 | 0.00 |
| 15 | WBPH | IR22 | 20 | 25 | 4 | 3 | 0.00 | 0.00 | 0.00 |
| 15 | WBPH | IR22 | 20 | 30 | 1 | 5 | 0.00 | 0.00 | 0.00 |
| 15 | WBPH | IR22 | 20 | 30 | 2 | 5 | 0.00 | 0.00 | 0.00 |
| 15 | WBPH | IR22 | 20 | 30 | 3 | 3 | 0.00 | 0.00 | 0.00 |
| 15 | WBPH | IR22 | 20 | 30 | 4 | 3 | 0.00 | 0.00 | 0.00 |
| 15 | WBPH | IR22 | 20 | 35 | 1 | 5 | 0.00 | 0.00 | 0.00 |
| 15 | WBPH | IR22 | 20 | 35 | 2 | 3 | 0.00 | 0.00 | 0.00 |
| 15 | WBPH | IR22 | 20 | 35 | 3 | 3 | 0.00 | 0.00 | 0.00 |
| 15 | WBPH | IR22 | 20 | 35 | 4 | 3 | 0.00 | 0.00 | 0.00 |
| 15 | WBPH | IR22 | 30 | 15 | 1 | 1 | 0.00 | 0.00 | 0.00 |
| 15 | WBPH | IR22 | 30 | 15 | 2 | 1 | 0.00 | 0.00 | 67.00 |
| 15 | WBPH | IR22 | 30 | 15 | 3 | 1 | 0.00 | 0.00 | 33.00 |
| 15 | WBPH | IR22 | 30 | 15 | 4 | 1 | 0.00 | 0.00 | 0.00 |
| 15 | WBPH | IR22 | 30 | 20 | 1 | 5 | 0.00 | 0.00 | 0.00 |
| 15 | WBPH | IR22 | 30 | 20 | 2 | 5 | 0.00 | 0.00 | 0.00 |
| 15 | WBPH | IR22 | 30 | 20 | 3 | 5 | 0.00 | 0.00 | 0.00 |
| 15 | WBPH | IR22 | 30 | 20 | 4 | 5 | 0.00 | 0.00 | 0.00 |
| 15 | WBPH | IR22 | 30 | 25 | 1 | 5 | 0.00 | 0.00 | 0.00 |
| 15 | WBPH | IR22 | 30 | 25 | 2 | 5 | 0.00 | 0.00 | 0.00 |
| 15 | WBPH | IR22 | 30 | 25 | 3 | 5 | 0.00 | 0.00 | 0.00 |
| 15 | WBPH | IR22 | 30 | 25 | 4 | 5 | 0.00 | 0.00 | 0.00 |
| 15 | WBPH | IR22 | 30 | 30 | 1 | 5 | 0.00 | 0.00 | 0.00 |
| 15 | WBPH | IR22 | 30 | 30 | 2 | 5 | 0.00 | 0.00 | 0.00 |
| 15 | WBPH | IR22 | 30 | 30 | 3 | 5 | 0.00 | 0.00 | 0.00 |
| 15 | WBPH | IR22 | 30 | 30 | 4 | 5 | 0.00 | 0.00 | 0.00 |
| 15 | WBPH | IR22 | 30 | 35 | 1 | 5 | 0.00 | 0.00 | 0.00 |
| 15 | WBPH | IR22 | 30 | 35 | 2 | 3 | 0.00 | 0.00 | 0.00 |
| 15 | WBPH | IR22 | 30 | 35 | 3 | 3 | 0.00 | 0.00 | 0.00 |
| 15 | WBPH | IR22 | 30 | 35 | 4 | 3 | 0.00 | 0.00 | 0.00 |
| 15 | WBPH | T65 | 20 | 15 | 1 | 3 | 1.00 | 0.00 | 44.44 |
| 15 | WBPH | T65 | 20 | 15 | 2 | 3 | 1.00 | 0.00 | 33.33 |
| 15 | WBPH | T65 | 20 | 15 | 3 | 3 | 1.00 | 0.00 | 33.33 |
| 15 | WBPH | T65 | 20 | 15 | 4 | 3 | 1.00 | 0.00 | 66.67 |
| 15 | WBPH | T65 | 20 | 20 | 1 | 5 | 0.33 | 0.00 | 11.11 |
| 15 | WBPH | T65 | 20 | 20 | 2 | 5 | 0.00 | 0.00 | 0.00 |
| 15 | WBPH | T65 | 20 | 20 | 3 | 3 | 0.00 | 0.00 | 0.00 |
| 15 | WBPH | T65 | 20 | 20 | 4 | 3 | 1.00 | 0.00 | 33.33 |
| 15 | WBPH | T65 | 20 | 25 | 1 | 5 | 0.50 | 0.00 | 16.67 |
| 15 | WBPH | T65 | 20 | 25 | 2 | 5 | 0.00 | 0.00 | 0.00 |
| 15 | WBPH | T65 | 20 | 25 | 3 | 3 | 0.00 | 0.00 | 0.00 |
| 15 | WBPH | T65 | 20 | 25 | 4 | 3 | 1.00 | 0.00 | 33.33 |
| 15 | WBPH | T65 | 20 | 30 | 1 | 5 | 0.00 | 0.00 | 0.00 |
| 15 | WBPH | T65 | 20 | 30 | 2 | 5 | 0.00 | 0.00 | 0.00 |
| 15 | WBPH | T65 | 20 | 30 | 3 | 3 | 0.00 | 0.00 | 0.00 |
| 15 | WBPH | T65 | 20 | 30 | 4 | 3 | 0.00 | 0.00 | 0.00 |
| 15 | WBPH | T65 | 20 | 35 | 1 | 5 | 0.00 | 0.00 | 0.00 |
| 15 | WBPH | T65 | 20 | 35 | 2 | 3 | 0.00 | 0.00 | 0.00 |
| 15 | WBPH | T65 | 20 | 35 | 3 | 3 | 0.00 | 0.00 | 0.00 |
| 15 | WBPH | T65 | 20 | 35 | 4 | 3 | 0.00 | 0.00 | 0.00 |
| 15 | WBPH | T65 | 30 | 15 | 1 | 1 | 0.00 | 0.00 | 0.00 |
| 15 | WBPH | T65 | 30 | 15 | 2 | 1 | 0.00 | 0.00 | 0.00 |
| 15 | WBPH | T65 | 30 | 15 | 3 | 1 | 0.00 | 0.00 | 80.00 |
| 15 | WBPH | T65 | 30 | 15 | 4 | 1 | 0.00 | 0.00 | 33.00 |
| 15 | WBPH | T65 | 30 | 20 | 1 | 5 | 0.00 | 0.00 | 0.00 |
| 15 | WBPH | T65 | 30 | 20 | 2 | 5 | 0.00 | 0.00 | 0.00 |
| 15 | WBPH | T65 | 30 | 20 | 3 | 5 | 0.00 | 0.00 | 0.00 |
| 15 | WBPH | T65 | 30 | 20 | 4 | 5 | 0.00 | 0.00 | 0.00 |
| 15 | WBPH | T65 | 30 | 25 | 1 | 5 | 0.00 | 0.00 | 0.00 |
| 15 | WBPH | T65 | 30 | 25 | 2 | 5 | 0.00 | 0.00 | 0.00 |
| 15 | WBPH | T65 | 30 | 25 | 3 | 5 | 0.00 | 0.00 | 0.00 |
| 15 | WBPH | T65 | 30 | 25 | 4 | 5 | 0.00 | 0.00 | 0.00 |
| 15 | WBPH | T65 | 30 | 30 | 1 | 5 | 0.00 | 0.00 | 0.00 |
| 15 | WBPH | T65 | 30 | 30 | 2 | 5 | 0.00 | 0.00 | 0.00 |
| 15 | WBPH | T65 | 30 | 30 | 3 | 5 | 0.00 | 0.00 | 0.00 |
| 15 | WBPH | T65 | 30 | 30 | 4 | 5 | 0.00 | 0.00 | 0.00 |
| 15 | WBPH | T65 | 30 | 35 | 1 | 5 | 0.00 | 0.00 | 0.00 |
| 15 | WBPH | T65 | 30 | 35 | 2 | 3 | 0.00 | 0.00 | 0.00 |
| 15 | WBPH | T65 | 30 | 35 | 3 | 3 | 0.00 | 0.00 | 0.00 |
| 15 | WBPH | T65 | 30 | 35 | 4 | 3 | 0.00 | 0.00 | 0.00 |
| 16 | BPH | IR22 | 20 | 15 | 1 | 3 | 0.33 | 0.00 | 11.11 |
| 16 | BPH | IR22 | 20 | 15 | 2 | 3 | 0.00 | 0.00 | 0.00 |
| 16 | BPH | IR22 | 20 | 15 | 3 | 3 | 0.00 | 0.00 | 0.00 |
| 16 | BPH | IR22 | 20 | 15 | 4 | 3 | 1.00 | 0.00 | 33.33 |
| 16 | BPH | IR22 | 20 | 20 | 1 | 5 | 0.67 | 3.00 | 17.78 |
| 16 | BPH | IR22 | 20 | 20 | 2 | 5 | 0.00 | 0.00 | 20.00 |
| 16 | BPH | IR22 | 20 | 20 | 3 | 3 | 2.00 | 9.00 | 33.33 |
| 16 | BPH | IR22 | 20 | 20 | 4 | 3 | 0.00 | 0.00 | 0.00 |
| 16 | BPH | IR22 | 20 | 25 | 1 | 5 | 2.00 | 6.50 | 10.00 |
| 16 | BPH | IR22 | 20 | 25 | 2 | 5 | 4.00 | 13.00 | 20.00 |
| 16 | BPH | IR22 | 20 | 25 | 3 | 3 | 0.00 | 0.00 | 0.00 |
| 16 | BPH | IR22 | 20 | 25 | 4 | 3 | 0.00 | 0.00 | 0.00 |
| 16 | BPH | IR22 | 20 | 30 | 1 | 5 | 0.00 | 0.00 | 0.00 |
| 16 | BPH | IR22 | 20 | 30 | 2 | 5 | 1.00 | 7.00 | 20.00 |
| 16 | BPH | IR22 | 20 | 30 | 3 | 5 | 0.00 | 0.00 | 0.00 |
| 16 | BPH | IR22 | 20 | 30 | 4 | 3 | 0.00 | 0.00 | 0.00 |
| 16 | BPH | IR22 | 20 | 35 | 1 | 5 | 0.00 | 0.00 | 0.00 |
| 16 | BPH | IR22 | 20 | 35 | 2 | 3 | 0.00 | 0.00 | 0.00 |
| 16 | BPH | IR22 | 20 | 35 | 3 | 3 | 0.00 | 0.00 | 0.00 |
| 16 | BPH | IR22 | 20 | 35 | 4 | 3 | 0.00 | 0.00 | 0.00 |
| 16 | BPH | IR22 | 30 | 15 | 1 | 3 | 0.00 | 0.00 | 0.00 |
| 16 | BPH | IR22 | 30 | 15 | 2 | 3 | 0.00 | 0.00 | 0.00 |
| 16 | BPH | IR22 | 30 | 15 | 3 | 3 | 1.00 | 0.50 | 66.67 |
| 16 | BPH | IR22 | 30 | 15 | 4 | 3 | 0.00 | 0.00 | 0.00 |
| 16 | BPH | IR22 | 30 | 20 | 1 | 5 | 0.00 | 0.00 | 0.00 |
| 16 | BPH | IR22 | 30 | 20 | 2 | 5 | 1.00 | 1.67 | 60.00 |
| 16 | BPH | IR22 | 30 | 20 | 3 | 5 | 0.00 | 0.00 | 0.00 |
| 16 | BPH | IR22 | 30 | 20 | 4 | 5 | 0.00 | 0.00 | 0.00 |
| 16 | BPH | IR22 | 30 | 25 | 1 | 5 | 0.00 | 0.00 | 0.00 |
| 16 | BPH | IR22 | 30 | 25 | 2 | 5 | 2.33 | 19.67 | 60.00 |
| 16 | BPH | IR22 | 30 | 25 | 3 | 5 | 0.00 | 0.00 | 0.00 |
| 16 | BPH | IR22 | 30 | 25 | 4 | 5 | 0.00 | 0.00 | 0.00 |
| 16 | BPH | IR22 | 30 | 30 | 1 | 5 | 0.00 | 0.00 | 0.00 |
| 16 | BPH | IR22 | 30 | 30 | 2 | 5 | 0.00 | 0.00 | 0.00 |
| 16 | BPH | IR22 | 30 | 30 | 3 | 5 | 0.00 | 0.00 | 0.00 |
| 16 | BPH | IR22 | 30 | 30 | 4 | 5 | 0.00 | 0.00 | 0.00 |
| 16 | BPH | IR22 | 30 | 35 | 1 | 5 | 0.00 | 0.00 | 0.00 |
| 16 | BPH | IR22 | 30 | 35 | 2 | 3 | 0.00 | 0.00 | 0.00 |
| 16 | BPH | IR22 | 30 | 35 | 3 | 3 | 0.00 | 0.00 | 0.00 |
| 16 | BPH | IR22 | 30 | 35 | 4 | 3 | 0.00 | 0.00 | 0.00 |
| 16 | BPH | T65 | 20 | 15 | 1 | 3 | 0.00 | 0.00 | 0.00 |
| 16 | BPH | T65 | 20 | 15 | 2 | 3 | 0.00 | 0.00 | 0.00 |
| 16 | BPH | T65 | 20 | 15 | 3 | 3 | 0.00 | 0.00 | 0.00 |
| 16 | BPH | T65 | 20 | 15 | 4 | 3 | 0.00 | 0.00 | 0.00 |
| 16 | BPH | T65 | 20 | 20 | 1 | 5 | 0.17 | 0.33 | 13.33 |
| 16 | BPH | T65 | 20 | 20 | 2 | 5 | 0.50 | 1.00 | 40.00 |
| 16 | BPH | T65 | 20 | 20 | 3 | 3 | 0.00 | 0.00 | 0.00 |
| 16 | BPH | T65 | 20 | 20 | 4 | 3 | 0.00 | 0.00 | 0.00 |
| 16 | BPH | T65 | 20 | 25 | 1 | 5 | 0.00 | 0.00 | 0.00 |
| 16 | BPH | T65 | 20 | 25 | 2 | 5 | 0.00 | 0.00 | 0.00 |
| 16 | BPH | T65 | 20 | 25 | 3 | 3 | 0.00 | 0.00 | 0.00 |
| 16 | BPH | T65 | 20 | 25 | 4 | 3 | 0.00 | 0.00 | 0.00 |
| 16 | BPH | T65 | 20 | 30 | 1 | 5 | 0.00 | 0.00 | 0.00 |
| 16 | BPH | T65 | 20 | 30 | 2 | 5 | 0.00 | 0.00 | 0.00 |
| 16 | BPH | T65 | 20 | 30 | 3 | 3 | 0.00 | 0.00 | 0.00 |
| 16 | BPH | T65 | 20 | 30 | 4 | 3 | 0.00 | 0.00 | 0.00 |
| 16 | BPH | T65 | 20 | 35 | 1 | 5 | 0.00 | 0.00 | 0.00 |
| 16 | BPH | T65 | 20 | 35 | 2 | 3 | 0.00 | 0.00 | 0.00 |
| 16 | BPH | T65 | 20 | 35 | 3 | 3 | 0.00 | 0.00 | 0.00 |
| 16 | BPH | T65 | 20 | 35 | 4 | 3 | 0.00 | 0.00 | 0.00 |
| 16 | BPH | T65 | 30 | 15 | 1 | 3 | 0.00 | 0.00 | 0.00 |
| 16 | BPH | T65 | 30 | 15 | 2 | 3 | 0.00 | 0.00 | 0.00 |
| 16 | BPH | T65 | 30 | 15 | 3 | 3 | 1.00 | 0.00 | 33.33 |
| 16 | BPH | T65 | 30 | 15 | 4 | 3 | 0.00 | 0.00 | 0.00 |
| 16 | BPH | T65 | 30 | 20 | 1 | 5 | 0.00 | 0.00 | 0.00 |
| 16 | BPH | T65 | 30 | 20 | 2 | 5 | 1.50 | 4.50 | 40.00 |
| 16 | BPH | T65 | 30 | 20 | 3 | 5 | 0.00 | 0.00 | 0.00 |
| 16 | BPH | T65 | 30 | 20 | 4 | 5 | 0.00 | 0.00 | 0.00 |
| 16 | BPH | T65 | 30 | 25 | 1 | 5 | 0.00 | 0.00 | 0.00 |
| 16 | BPH | T65 | 30 | 25 | 2 | 5 | 1.00 | 3.00 | 80.00 |
| 16 | BPH | T65 | 30 | 25 | 3 | 5 | 0.00 | 0.00 | 0.00 |
| 16 | BPH | T65 | 30 | 25 | 4 | 5 | 0.00 | 0.00 | 0.00 |
| 16 | BPH | T65 | 30 | 30 | 1 | 5 | 0.00 | 0.00 | 0.00 |
| 16 | BPH | T65 | 30 | 30 | 2 | 5 | 1.00 | 0.00 | 20.00 |
| 16 | BPH | T65 | 30 | 30 | 3 | 5 | 0.00 | 0.00 | 0.00 |
| 16 | BPH | T65 | 30 | 30 | 4 | 5 | 0.00 | 0.00 | 0.00 |
| 16 | BPH | T65 | 30 | 35 | 1 | 5 | 0.00 | 0.00 | 0.00 |
| 16 | BPH | T65 | 30 | 35 | 2 | 3 | 0.00 | 0.00 | 0.00 |
| 16 | BPH | T65 | 30 | 35 | 3 | 3 | 0.00 | 0.00 | 0.00 |
| 16 | BPH | T65 | 30 | 35 | 4 | 3 | 0.00 | 0.00 | 0.00 |
| 16 | WBPH | IR22 | 20 | 15 | 1 | 3 | 0.33 | 0.00 | 11.11 |
| 16 | WBPH | IR22 | 20 | 15 | 2 | 3 | 0.00 | 0.00 | 0.00 |
| 16 | WBPH | IR22 | 20 | 15 | 3 | 3 | 0.00 | 0.00 | 0.00 |
| 16 | WBPH | IR22 | 20 | 15 | 4 | 3 | 1.00 | 0.00 | 33.33 |
| 16 | WBPH | IR22 | 20 | 20 | 1 | 5 | 0.00 | 0.00 | 0.00 |
| 16 | WBPH | IR22 | 20 | 20 | 2 | 5 | 0.00 | 0.00 | 0.00 |
| 16 | WBPH | IR22 | 20 | 20 | 3 | 3 | 0.00 | 0.00 | 0.00 |
| 16 | WBPH | IR22 | 20 | 20 | 4 | 3 | 0.00 | 0.00 | 0.00 |
| 16 | WBPH | IR22 | 20 | 25 | 1 | 5 | 0.00 | 0.00 | 0.00 |
| 16 | WBPH | IR22 | 20 | 25 | 2 | 5 | 0.00 | 0.00 | 0.00 |
| 16 | WBPH | IR22 | 20 | 25 | 3 | 3 | 0.00 | 0.00 | 0.00 |
| 16 | WBPH | IR22 | 20 | 25 | 4 | 3 | 0.00 | 0.00 | 0.00 |
| 16 | WBPH | IR22 | 20 | 30 | 1 | 5 | 0.00 | 0.00 | 0.00 |
| 16 | WBPH | IR22 | 20 | 30 | 2 | 5 | 0.00 | 0.00 | 0.00 |
| 16 | WBPH | IR22 | 20 | 30 | 3 | 3 | 0.00 | 0.00 | 0.00 |
| 16 | WBPH | IR22 | 20 | 30 | 4 | 3 | 0.00 | 0.00 | 0.00 |
| 16 | WBPH | IR22 | 20 | 35 | 1 | 5 | 0.00 | 0.00 | 0.00 |
| 16 | WBPH | IR22 | 20 | 35 | 2 | 3 | 0.00 | 0.00 | 0.00 |
| 16 | WBPH | IR22 | 20 | 35 | 3 | 3 | 0.00 | 0.00 | 0.00 |
| 16 | WBPH | IR22 | 20 | 35 | 4 | 3 | 0.00 | 0.00 | 0.00 |
| 16 | WBPH | IR22 | 30 | 15 | 1 | 1 | 0.00 | 0.00 | 0.00 |
| 16 | WBPH | IR22 | 30 | 15 | 2 | 1 | 0.00 | 0.00 | 67.00 |
| 16 | WBPH | IR22 | 30 | 15 | 3 | 1 | 0.00 | 0.00 | 33.00 |
| 16 | WBPH | IR22 | 30 | 15 | 4 | 1 | 0.00 | 0.00 | 0.00 |
| 16 | WBPH | IR22 | 30 | 20 | 1 | 5 | 0.00 | 0.00 | 0.00 |
| 16 | WBPH | IR22 | 30 | 20 | 2 | 5 | 0.00 | 0.00 | 0.00 |
| 16 | WBPH | IR22 | 30 | 20 | 3 | 5 | 0.00 | 0.00 | 0.00 |
| 16 | WBPH | IR22 | 30 | 20 | 4 | 5 | 0.00 | 0.00 | 0.00 |
| 16 | WBPH | IR22 | 30 | 25 | 1 | 5 | 0.00 | 0.00 | 0.00 |
| 16 | WBPH | IR22 | 30 | 25 | 2 | 5 | 0.00 | 0.00 | 0.00 |
| 16 | WBPH | IR22 | 30 | 25 | 3 | 5 | 0.00 | 0.00 | 0.00 |
| 16 | WBPH | IR22 | 30 | 25 | 4 | 5 | 0.00 | 0.00 | 0.00 |
| 16 | WBPH | IR22 | 30 | 30 | 1 | 5 | 0.00 | 0.00 | 0.00 |
| 16 | WBPH | IR22 | 30 | 30 | 2 | 5 | 0.00 | 0.00 | 0.00 |
| 16 | WBPH | IR22 | 30 | 30 | 3 | 5 | 0.00 | 0.00 | 0.00 |
| 16 | WBPH | IR22 | 30 | 30 | 4 | 5 | 0.00 | 0.00 | 0.00 |
| 16 | WBPH | IR22 | 30 | 35 | 1 | 5 | 0.00 | 0.00 | 0.00 |
| 16 | WBPH | IR22 | 30 | 35 | 2 | 3 | 0.00 | 0.00 | 0.00 |
| 16 | WBPH | IR22 | 30 | 35 | 3 | 3 | 0.00 | 0.00 | 0.00 |
| 16 | WBPH | IR22 | 30 | 35 | 4 | 3 | 0.00 | 0.00 | 0.00 |
| 16 | WBPH | T65 | 20 | 15 | 1 | 3 | 1.00 | 0.83 | 44.44 |
| 16 | WBPH | T65 | 20 | 15 | 2 | 3 | 1.00 | 0.00 | 33.33 |
| 16 | WBPH | T65 | 20 | 15 | 3 | 3 | 1.00 | 0.00 | 33.33 |
| 16 | WBPH | T65 | 20 | 15 | 4 | 3 | 1.00 | 2.50 | 66.67 |
| 16 | WBPH | T65 | 20 | 20 | 1 | 5 | 0.33 | 0.00 | 11.11 |
| 16 | WBPH | T65 | 20 | 20 | 2 | 5 | 0.00 | 0.00 | 0.00 |
| 16 | WBPH | T65 | 20 | 20 | 3 | 3 | 0.00 | 0.00 | 0.00 |
| 16 | WBPH | T65 | 20 | 20 | 4 | 3 | 1.00 | 0.00 | 33.33 |
| 16 | WBPH | T65 | 20 | 25 | 1 | 5 | 0.00 | 0.00 | 0.00 |
| 16 | WBPH | T65 | 20 | 25 | 2 | 5 | 0.00 | 0.00 | 0.00 |
| 16 | WBPH | T65 | 20 | 25 | 3 | 3 | 0.00 | 0.00 | 0.00 |
| 16 | WBPH | T65 | 20 | 25 | 4 | 3 | 0.00 | 0.00 | 0.00 |
| 16 | WBPH | T65 | 20 | 30 | 1 | 5 | 0.00 | 0.00 | 0.00 |
| 16 | WBPH | T65 | 20 | 30 | 2 | 5 | 0.00 | 0.00 | 0.00 |
| 16 | WBPH | T65 | 20 | 30 | 3 | 3 | 0.00 | 0.00 | 0.00 |
| 16 | WBPH | T65 | 20 | 30 | 4 | 3 | 0.00 | 0.00 | 0.00 |
| 16 | WBPH | T65 | 20 | 35 | 1 | 5 | 0.00 | 0.00 | 0.00 |
| 16 | WBPH | T65 | 20 | 35 | 2 | 3 | 0.00 | 0.00 | 0.00 |
| 16 | WBPH | T65 | 20 | 35 | 3 | 3 | 0.00 | 0.00 | 0.00 |
| 16 | WBPH | T65 | 20 | 35 | 4 | 3 | 0.00 | 0.00 | 0.00 |
| 16 | WBPH | T65 | 30 | 15 | 1 | 1 | 0.00 | 0.00 | 0.00 |
| 16 | WBPH | T65 | 30 | 15 | 2 | 1 | 0.00 | 0.00 | 0.00 |
| 16 | WBPH | T65 | 30 | 15 | 3 | 1 | 0.00 | 0.00 | 80.00 |
| 16 | WBPH | T65 | 30 | 15 | 4 | 1 | 0.00 | 0.00 | 33.00 |
| 16 | WBPH | T65 | 30 | 20 | 1 | 5 | 0.00 | 0.00 | 0.00 |
| 16 | WBPH | T65 | 30 | 20 | 2 | 5 | 0.00 | 0.00 | 0.00 |
| 16 | WBPH | T65 | 30 | 20 | 3 | 5 | 0.00 | 0.00 | 0.00 |
| 16 | WBPH | T65 | 30 | 20 | 4 | 5 | 0.00 | 0.00 | 0.00 |
| 16 | WBPH | T65 | 30 | 25 | 1 | 5 | 0.00 | 0.00 | 0.00 |
| 16 | WBPH | T65 | 30 | 25 | 2 | 5 | 0.00 | 0.00 | 0.00 |
| 16 | WBPH | T65 | 30 | 25 | 3 | 5 | 0.00 | 0.00 | 0.00 |
| 16 | WBPH | T65 | 30 | 25 | 4 | 5 | 0.00 | 0.00 | 0.00 |
| 16 | WBPH | T65 | 30 | 30 | 1 | 5 | 0.00 | 0.00 | 0.00 |
| 16 | WBPH | T65 | 30 | 30 | 2 | 5 | 0.00 | 0.00 | 0.00 |
| 16 | WBPH | T65 | 30 | 30 | 3 | 5 | 0.00 | 0.00 | 0.00 |
| 16 | WBPH | T65 | 30 | 30 | 4 | 5 | 0.00 | 0.00 | 0.00 |
| 16 | WBPH | T65 | 30 | 35 | 1 | 5 | 0.00 | 0.00 | 0.00 |
| 16 | WBPH | T65 | 30 | 35 | 2 | 3 | 0.00 | 0.00 | 0.00 |
| 16 | WBPH | T65 | 30 | 35 | 3 | 3 | 0.00 | 0.00 | 0.00 |
| 16 | WBPH | T65 | 30 | 35 | 4 | 3 | 0.00 | 0.00 | 0.00 |
| 17 | BPH | IR22 | 20 | 15 | 1 | 3 | 0.33 | 0.00 | 11.11 |
| 17 | BPH | IR22 | 20 | 15 | 2 | 3 | 0.00 | 0.00 | 0.00 |
| 17 | BPH | IR22 | 20 | 15 | 3 | 3 | 0.00 | 0.00 | 0.00 |
| 17 | BPH | IR22 | 20 | 15 | 4 | 3 | 1.00 | 0.00 | 33.33 |
| 17 | BPH | IR22 | 20 | 20 | 1 | 5 | 1.00 | 2.00 | 17.78 |
| 17 | BPH | IR22 | 20 | 20 | 2 | 5 | 2.00 | 6.00 | 20.00 |
| 17 | BPH | IR22 | 20 | 20 | 3 | 3 | 1.00 | 0.00 | 33.33 |
| 17 | BPH | IR22 | 20 | 20 | 4 | 3 | 0.00 | 0.00 | 0.00 |
| 17 | BPH | IR22 | 20 | 25 | 1 | 5 | 2.00 | 2.00 | 0.00 |
| 17 | BPH | IR22 | 20 | 25 | 2 | 5 | 2.00 | 2.00 | 20.00 |
| 17 | BPH | IR22 | 20 | 25 | 3 | 5 | 0.00 | 0.00 | 0.00 |
| 17 | BPH | IR22 | 20 | 25 | 4 | 5 | 0.00 | 0.00 | 0.00 |
| 17 | BPH | IR22 | 20 | 30 | 1 | 5 | 0.00 | 0.00 | 0.00 |
| 17 | BPH | IR22 | 20 | 30 | 2 | 5 | 4.00 | 36.00 | 20.00 |
| 17 | BPH | IR22 | 20 | 30 | 3 | 5 | 0.00 | 0.00 | 0.00 |
| 17 | BPH | IR22 | 20 | 30 | 4 | 5 | 0.00 | 0.00 | 0.00 |
| 17 | BPH | IR22 | 30 | 15 | 1 | 3 | 0.00 | 0.00 | 0.00 |
| 17 | BPH | IR22 | 30 | 15 | 2 | 3 | 0.00 | 0.00 | 0.00 |
| 17 | BPH | IR22 | 30 | 15 | 3 | 3 | 1.50 | 4.50 | 66.67 |
| 17 | BPH | IR22 | 30 | 15 | 4 | 3 | 0.00 | 0.00 | 0.00 |
| 17 | BPH | IR22 | 30 | 20 | 1 | 5 | 0.00 | 0.00 | 0.00 |
| 17 | BPH | IR22 | 30 | 20 | 2 | 5 | 2.33 | 11.00 | 60.00 |
| 17 | BPH | IR22 | 30 | 20 | 3 | 5 | 0.00 | 0.00 | 0.00 |
| 17 | BPH | IR22 | 30 | 20 | 4 | 5 | 0.00 | 0.00 | 0.00 |
| 17 | BPH | IR22 | 30 | 25 | 1 | 5 | 0.00 | 0.00 | 0.00 |
| 17 | BPH | IR22 | 30 | 25 | 2 | 5 | 2.00 | 14.33 | 60.00 |
| 17 | BPH | IR22 | 30 | 25 | 3 | 5 | 0.00 | 0.00 | 0.00 |
| 17 | BPH | IR22 | 30 | 25 | 4 | 5 | 0.00 | 0.00 | 0.00 |
| 17 | BPH | IR22 | 30 | 30 | 1 | 5 | 0.00 | 0.00 | 0.00 |
| 17 | BPH | IR22 | 30 | 30 | 2 | 5 | 0.00 | 0.00 | 0.00 |
| 17 | BPH | IR22 | 30 | 30 | 3 | 5 | 0.00 | 0.00 | 0.00 |
| 17 | BPH | IR22 | 30 | 30 | 4 | 5 | 0.00 | 0.00 | 0.00 |
| 17 | BPH | IR22 | 30 | 35 | 1 | 5 | 0.00 | 0.00 | 0.00 |
| 17 | BPH | IR22 | 30 | 35 | 2 | 3 | 0.00 | 0.00 | 0.00 |
| 17 | BPH | IR22 | 30 | 35 | 3 | 3 | 0.00 | 0.00 | 0.00 |
| 17 | BPH | IR22 | 30 | 35 | 4 | 3 | 0.00 | 0.00 | 0.00 |
| 17 | BPH | T65 | 20 | 15 | 1 | 3 | 0.00 | 0.00 | 0.00 |
| 17 | BPH | T65 | 20 | 15 | 2 | 3 | 0.00 | 0.00 | 0.00 |
| 17 | BPH | T65 | 20 | 15 | 3 | 3 | 0.00 | 0.00 | 0.00 |
| 17 | BPH | T65 | 20 | 15 | 4 | 3 | 0.00 | 0.00 | 0.00 |
| 17 | BPH | T65 | 20 | 20 | 1 | 5 | 0.00 | 0.00 | 0.00 |
| 17 | BPH | T65 | 20 | 20 | 2 | 5 | 1.00 | 5.00 | 40.00 |
| 17 | BPH | T65 | 20 | 20 | 3 | 3 | 0.00 | 0.00 | 0.00 |
| 17 | BPH | T65 | 20 | 20 | 4 | 3 | 0.00 | 0.00 | 0.00 |
| 17 | BPH | T65 | 20 | 25 | 1 | 5 | 0.00 | 0.00 | 0.00 |
| 17 | BPH | T65 | 20 | 25 | 2 | 5 | 0.00 | 0.00 | 0.00 |
| 17 | BPH | T65 | 20 | 25 | 3 | 5 | 0.00 | 0.00 | 0.00 |
| 17 | BPH | T65 | 20 | 25 | 4 | 5 | 0.00 | 0.00 | 0.00 |
| 17 | BPH | T65 | 20 | 30 | 1 | 5 | 0.00 | 0.00 | 0.00 |
| 17 | BPH | T65 | 20 | 30 | 2 | 5 | 0.00 | 0.00 | 0.00 |
| 17 | BPH | T65 | 20 | 30 | 3 | 5 | 0.00 | 0.00 | 0.00 |
| 17 | BPH | T65 | 20 | 30 | 4 | 5 | 0.00 | 0.00 | 0.00 |
| 17 | BPH | T65 | 20 | 35 | 1 | 5 | 0.00 | 0.00 | 0.00 |
| 17 | BPH | T65 | 20 | 35 | 2 | 3 | 0.00 | 0.00 | 0.00 |
| 17 | BPH | T65 | 20 | 35 | 3 | 3 | 0.00 | 0.00 | 0.00 |
| 17 | BPH | T65 | 20 | 35 | 4 | 3 | 0.00 | 0.00 | 0.00 |
| 17 | BPH | T65 | 30 | 15 | 1 | 3 | 0.00 | 0.00 | 0.00 |
| 17 | BPH | T65 | 30 | 15 | 2 | 3 | 0.00 | 0.00 | 0.00 |
| 17 | BPH | T65 | 30 | 15 | 3 | 3 | 0.00 | 0.00 | 0.00 |
| 17 | BPH | T65 | 30 | 15 | 4 | 3 | 0.00 | 0.00 | 0.00 |
| 17 | BPH | T65 | 30 | 20 | 1 | 5 | 0.00 | 0.00 | 0.00 |
| 17 | BPH | T65 | 30 | 20 | 2 | 5 | 2.00 | 13.50 | 40.00 |
| 17 | BPH | T65 | 30 | 20 | 3 | 5 | 0.00 | 0.00 | 0.00 |
| 17 | BPH | T65 | 30 | 20 | 4 | 5 | 0.00 | 0.00 | 0.00 |
| 17 | BPH | T65 | 30 | 25 | 1 | 5 | 0.00 | 0.00 | 0.00 |
| 17 | BPH | T65 | 30 | 25 | 2 | 5 | 1.33 | 2.67 | 60.00 |
| 17 | BPH | T65 | 30 | 25 | 3 | 5 | 0.00 | 0.00 | 0.00 |
| 17 | BPH | T65 | 30 | 25 | 4 | 5 | 0.00 | 0.00 | 0.00 |
| 17 | BPH | T65 | 30 | 30 | 1 | 5 | 0.00 | 0.00 | 0.00 |
| 17 | BPH | T65 | 30 | 30 | 2 | 5 | 3.00 | 12.00 | 20.00 |
| 17 | BPH | T65 | 30 | 30 | 3 | 5 | 0.00 | 0.00 | 0.00 |
| 17 | BPH | T65 | 30 | 30 | 4 | 5 | 0.00 | 0.00 | 0.00 |
| 17 | BPH | T65 | 30 | 35 | 1 | 5 | 0.00 | 0.00 | 0.00 |
| 17 | BPH | T65 | 30 | 35 | 2 | 3 | 0.00 | 0.00 | 0.00 |
| 17 | BPH | T65 | 30 | 35 | 3 | 3 | 0.00 | 0.00 | 0.00 |
| 17 | BPH | T65 | 30 | 35 | 4 | 3 | 0.00 | 0.00 | 0.00 |
| 17 | WBPH | IR22 | 20 | 15 | 1 | 3 | 0.33 | 0.00 | 11.11 |
| 17 | WBPH | IR22 | 20 | 15 | 2 | 3 | 0.00 | 0.00 | 0.00 |
| 17 | WBPH | IR22 | 20 | 15 | 3 | 3 | 0.00 | 0.00 | 0.00 |
| 17 | WBPH | IR22 | 20 | 15 | 4 | 3 | 1.00 | 0.00 | 33.33 |
| 17 | WBPH | IR22 | 20 | 20 | 1 | 5 | 0.00 | 0.00 | 0.00 |
| 17 | WBPH | IR22 | 20 | 20 | 2 | 5 | 0.00 | 0.00 | 0.00 |
| 17 | WBPH | IR22 | 20 | 20 | 3 | 3 | 0.00 | 0.00 | 0.00 |
| 17 | WBPH | IR22 | 20 | 20 | 4 | 3 | 0.00 | 0.00 | 0.00 |
| 17 | WBPH | IR22 | 20 | 25 | 1 | 5 | 0.00 | 0.00 | 0.00 |
| 17 | WBPH | IR22 | 20 | 25 | 2 | 5 | 0.00 | 0.00 | 0.00 |
| 17 | WBPH | IR22 | 20 | 25 | 3 | 5 | 0.00 | 0.00 | 0.00 |
| 17 | WBPH | IR22 | 20 | 25 | 4 | 5 | 0.00 | 0.00 | 0.00 |
| 17 | WBPH | IR22 | 20 | 30 | 1 | 5 | 0.00 | 0.00 | 0.00 |
| 17 | WBPH | IR22 | 20 | 30 | 2 | 5 | 0.00 | 0.00 | 0.00 |
| 17 | WBPH | IR22 | 20 | 30 | 3 | 5 | 0.00 | 0.00 | 0.00 |
| 17 | WBPH | IR22 | 20 | 30 | 4 | 5 | 0.00 | 0.00 | 0.00 |
| 17 | WBPH | IR22 | 20 | 35 | 1 | 5 | 0.00 | 0.00 | 0.00 |
| 17 | WBPH | IR22 | 20 | 35 | 2 | 3 | 0.00 | 0.00 | 0.00 |
| 17 | WBPH | IR22 | 20 | 35 | 3 | 3 | 0.00 | 0.00 | 0.00 |
| 17 | WBPH | IR22 | 20 | 35 | 4 | 3 | 0.00 | 0.00 | 0.00 |
| 17 | WBPH | IR22 | 30 | 15 | 1 | 1 | 0.00 | 0.00 | 0.00 |
| 17 | WBPH | IR22 | 30 | 15 | 2 | 1 | 0.00 | 0.00 | 67.00 |
| 17 | WBPH | IR22 | 30 | 15 | 3 | 1 | 0.00 | 0.00 | 33.00 |
| 17 | WBPH | IR22 | 30 | 15 | 4 | 1 | 0.00 | 0.00 | 0.00 |
| 17 | WBPH | IR22 | 30 | 20 | 1 | 5 | 0.00 | 0.00 | 0.00 |
| 17 | WBPH | IR22 | 30 | 20 | 2 | 5 | 0.00 | 0.00 | 0.00 |
| 17 | WBPH | IR22 | 30 | 20 | 3 | 5 | 0.00 | 0.00 | 0.00 |
| 17 | WBPH | IR22 | 30 | 20 | 4 | 5 | 0.00 | 0.00 | 0.00 |
| 17 | WBPH | IR22 | 30 | 25 | 1 | 5 | 0.00 | 0.00 | 0.00 |
| 17 | WBPH | IR22 | 30 | 25 | 2 | 5 | 0.00 | 0.00 | 0.00 |
| 17 | WBPH | IR22 | 30 | 25 | 3 | 5 | 0.00 | 0.00 | 0.00 |
| 17 | WBPH | IR22 | 30 | 25 | 4 | 5 | 0.00 | 0.00 | 0.00 |
| 17 | WBPH | IR22 | 30 | 30 | 1 | 5 | 0.00 | 0.00 | 0.00 |
| 17 | WBPH | IR22 | 30 | 30 | 2 | 5 | 0.00 | 0.00 | 0.00 |
| 17 | WBPH | IR22 | 30 | 30 | 3 | 5 | 0.00 | 0.00 | 0.00 |
| 17 | WBPH | IR22 | 30 | 30 | 4 | 5 | 0.00 | 0.00 | 0.00 |
| 17 | WBPH | IR22 | 30 | 35 | 1 | 5 | 0.00 | 0.00 | 0.00 |
| 17 | WBPH | IR22 | 30 | 35 | 2 | 3 | 0.00 | 0.00 | 0.00 |
| 17 | WBPH | IR22 | 30 | 35 | 3 | 3 | 0.00 | 0.00 | 0.00 |
| 17 | WBPH | IR22 | 30 | 35 | 4 | 3 | 0.00 | 0.00 | 0.00 |
| 17 | WBPH | T65 | 20 | 15 | 1 | 3 | 1.00 | 0.00 | 44.44 |
| 17 | WBPH | T65 | 20 | 15 | 2 | 3 | 1.00 | 0.00 | 33.33 |
| 17 | WBPH | T65 | 20 | 15 | 3 | 3 | 1.00 | 0.00 | 33.33 |
| 17 | WBPH | T65 | 20 | 15 | 4 | 3 | 1.00 | 0.00 | 66.67 |
| 17 | WBPH | T65 | 20 | 20 | 1 | 5 | 0.33 | 0.00 | 11.11 |
| 17 | WBPH | T65 | 20 | 20 | 2 | 5 | 0.00 | 0.00 | 0.00 |
| 17 | WBPH | T65 | 20 | 20 | 3 | 3 | 0.00 | 0.00 | 0.00 |
| 17 | WBPH | T65 | 20 | 20 | 4 | 3 | 1.00 | 0.00 | 33.33 |
| 17 | WBPH | T65 | 20 | 25 | 1 | 5 | 0.00 | 0.00 | 0.00 |
| 17 | WBPH | T65 | 20 | 25 | 2 | 5 | 0.00 | 0.00 | 0.00 |
| 17 | WBPH | T65 | 20 | 25 | 3 | 5 | 0.00 | 0.00 | 0.00 |
| 17 | WBPH | T65 | 20 | 25 | 4 | 5 | 0.00 | 0.00 | 0.00 |
| 17 | WBPH | T65 | 20 | 30 | 1 | 5 | 0.00 | 0.00 | 0.00 |
| 17 | WBPH | T65 | 20 | 30 | 2 | 5 | 0.00 | 0.00 | 0.00 |
| 17 | WBPH | T65 | 20 | 30 | 3 | 5 | 0.00 | 0.00 | 0.00 |
| 17 | WBPH | T65 | 20 | 30 | 4 | 5 | 0.00 | 0.00 | 0.00 |
| 17 | WBPH | T65 | 20 | 35 | 1 | 5 | 0.00 | 0.00 | 0.00 |
| 17 | WBPH | T65 | 20 | 35 | 2 | 3 | 0.00 | 0.00 | 0.00 |
| 17 | WBPH | T65 | 20 | 35 | 3 | 3 | 0.00 | 0.00 | 0.00 |
| 17 | WBPH | T65 | 20 | 35 | 4 | 3 | 0.00 | 0.00 | 0.00 |
| 17 | WBPH | T65 | 30 | 15 | 1 | 1 | 0.00 | 0.00 | 0.00 |
| 17 | WBPH | T65 | 30 | 15 | 2 | 1 | 0.00 | 0.00 | 0.00 |
| 17 | WBPH | T65 | 30 | 15 | 3 | 1 | 0.00 | 0.00 | 80.00 |
| 17 | WBPH | T65 | 30 | 15 | 4 | 1 | 0.00 | 0.00 | 33.00 |
| 17 | WBPH | T65 | 30 | 20 | 1 | 5 | 0.00 | 0.00 | 0.00 |
| 17 | WBPH | T65 | 30 | 20 | 2 | 5 | 0.00 | 0.00 | 0.00 |
| 17 | WBPH | T65 | 30 | 20 | 3 | 5 | 0.00 | 0.00 | 0.00 |
| 17 | WBPH | T65 | 30 | 20 | 4 | 5 | 0.00 | 0.00 | 0.00 |
| 17 | WBPH | T65 | 30 | 25 | 1 | 5 | 0.00 | 0.00 | 0.00 |
| 17 | WBPH | T65 | 30 | 25 | 2 | 5 | 0.00 | 0.00 | 0.00 |
| 17 | WBPH | T65 | 30 | 25 | 3 | 5 | 0.00 | 0.00 | 0.00 |
| 17 | WBPH | T65 | 30 | 25 | 4 | 5 | 0.00 | 0.00 | 0.00 |
| 17 | WBPH | T65 | 30 | 30 | 1 | 5 | 0.00 | 0.00 | 0.00 |
| 17 | WBPH | T65 | 30 | 30 | 2 | 5 | 0.00 | 0.00 | 0.00 |
| 17 | WBPH | T65 | 30 | 30 | 3 | 5 | 0.00 | 0.00 | 0.00 |
| 17 | WBPH | T65 | 30 | 30 | 4 | 5 | 0.00 | 0.00 | 0.00 |
| 17 | WBPH | T65 | 30 | 35 | 1 | 5 | 0.00 | 0.00 | 0.00 |
| 17 | WBPH | T65 | 30 | 35 | 2 | 3 | 0.00 | 0.00 | 0.00 |
| 17 | WBPH | T65 | 30 | 35 | 3 | 3 | 0.00 | 0.00 | 0.00 |
| 17 | WBPH | T65 | 30 | 35 | 4 | 3 | 0.00 | 0.00 | 0.00 |
| 18 | BPH | IR22 | 20 | 15 | 1 | 3 | 0.33 | 0.00 | 11.11 |
| 18 | BPH | IR22 | 20 | 15 | 2 | 3 | 0.00 | 0.00 | 0.00 |
| 18 | BPH | IR22 | 20 | 15 | 3 | 3 | 0.00 | 0.00 | 0.00 |
| 18 | BPH | IR22 | 20 | 15 | 4 | 3 | 1.00 | 0.00 | 33.33 |
| 18 | BPH | IR22 | 20 | 20 | 1 | 5 | 0.33 | 0.00 | 17.78 |
| 18 | BPH | IR22 | 20 | 20 | 2 | 5 | 0.00 | 0.00 | 20.00 |
| 18 | BPH | IR22 | 20 | 20 | 3 | 3 | 1.00 | 0.00 | 33.33 |
| 18 | BPH | IR22 | 20 | 20 | 4 | 3 | 0.00 | 0.00 | 0.00 |
| 18 | BPH | IR22 | 20 | 25 | 1 | 5 | 0.00 | 0.00 | 0.00 |
| 18 | BPH | IR22 | 20 | 25 | 2 | 5 | 0.00 | 0.00 | 20.00 |
| 18 | BPH | IR22 | 20 | 25 | 3 | 5 | 0.00 | 0.00 | 0.00 |
| 18 | BPH | IR22 | 20 | 25 | 4 | 5 | 0.00 | 0.00 | 0.00 |
| 18 | BPH | IR22 | 20 | 30 | 1 | 5 | 0.00 | 0.00 | 0.00 |
| 18 | BPH | IR22 | 20 | 30 | 2 | 5 | 0.00 | 0.00 | 20.00 |
| 18 | BPH | IR22 | 20 | 30 | 3 | 5 | 0.00 | 0.00 | 0.00 |
| 18 | BPH | IR22 | 20 | 30 | 4 | 5 | 0.00 | 0.00 | 0.00 |
| 18 | BPH | IR22 | 20 | 35 | 1 | 5 | 0.00 | 0.00 | 0.00 |
| 18 | BPH | IR22 | 20 | 35 | 2 | 3 | 0.00 | 0.00 | 0.00 |
| 18 | BPH | IR22 | 20 | 35 | 3 | 3 | 0.00 | 0.00 | 0.00 |
| 18 | BPH | IR22 | 20 | 35 | 4 | 3 | 0.00 | 0.00 | 0.00 |
| 18 | BPH | IR22 | 30 | 15 | 1 | 3 | 0.00 | 0.00 | 0.00 |
| 18 | BPH | IR22 | 30 | 15 | 2 | 3 | 0.00 | 0.00 | 0.00 |
| 18 | BPH | IR22 | 30 | 15 | 3 | 3 | 1.00 | 0.00 | 66.67 |
| 18 | BPH | IR22 | 30 | 15 | 4 | 3 | 0.00 | 0.00 | 0.00 |
| 18 | BPH | IR22 | 30 | 20 | 1 | 5 | 0.00 | 0.00 | 0.00 |
| 18 | BPH | IR22 | 30 | 20 | 2 | 5 | 2.33 | 6.33 | 60.00 |
| 18 | BPH | IR22 | 30 | 20 | 3 | 5 | 0.00 | 0.00 | 0.00 |
| 18 | BPH | IR22 | 30 | 20 | 4 | 5 | 0.00 | 0.00 | 0.00 |
| 18 | BPH | IR22 | 30 | 25 | 1 | 5 | 0.00 | 0.00 | 0.00 |
| 18 | BPH | IR22 | 30 | 25 | 2 | 5 | 1.33 | 6.33 | 60.00 |
| 18 | BPH | IR22 | 30 | 25 | 3 | 5 | 0.00 | 0.00 | 0.00 |
| 18 | BPH | IR22 | 30 | 25 | 4 | 5 | 0.00 | 0.00 | 0.00 |
| 18 | BPH | IR22 | 30 | 30 | 1 | 5 | 0.00 | 0.00 | 0.00 |
| 18 | BPH | IR22 | 30 | 30 | 2 | 5 | 0.00 | 0.00 | 0.00 |
| 18 | BPH | IR22 | 30 | 30 | 3 | 5 | 0.00 | 0.00 | 0.00 |
| 18 | BPH | IR22 | 30 | 30 | 4 | 5 | 0.00 | 0.00 | 0.00 |
| 18 | BPH | IR22 | 30 | 35 | 1 | 5 | 0.00 | 0.00 | 0.00 |
| 18 | BPH | IR22 | 30 | 35 | 2 | 3 | 0.00 | 0.00 | 0.00 |
| 18 | BPH | IR22 | 30 | 35 | 3 | 3 | 0.00 | 0.00 | 0.00 |
| 18 | BPH | IR22 | 30 | 35 | 4 | 3 | 0.00 | 0.00 | 0.00 |
| 18 | BPH | T65 | 20 | 15 | 1 | 3 | 0.00 | 0.00 | 0.00 |
| 18 | BPH | T65 | 20 | 15 | 2 | 3 | 0.00 | 0.00 | 0.00 |
| 18 | BPH | T65 | 20 | 15 | 3 | 3 | 0.00 | 0.00 | 0.00 |
| 18 | BPH | T65 | 20 | 15 | 4 | 3 | 0.00 | 0.00 | 0.00 |
| 18 | BPH | T65 | 20 | 20 | 1 | 5 | 0.00 | 0.00 | 0.00 |
| 18 | BPH | T65 | 20 | 20 | 2 | 5 | 0.00 | 0.00 | 20.00 |
| 18 | BPH | T65 | 20 | 20 | 3 | 3 | 0.00 | 0.00 | 0.00 |
| 18 | BPH | T65 | 20 | 20 | 4 | 3 | 0.00 | 0.00 | 0.00 |
| 18 | BPH | T65 | 20 | 25 | 1 | 5 | 0.00 | 0.00 | 0.00 |
| 18 | BPH | T65 | 20 | 25 | 2 | 5 | 0.00 | 0.00 | 0.00 |
| 18 | BPH | T65 | 20 | 25 | 3 | 5 | 0.00 | 0.00 | 0.00 |
| 18 | BPH | T65 | 20 | 25 | 4 | 5 | 0.00 | 0.00 | 0.00 |
| 18 | BPH | T65 | 20 | 30 | 1 | 5 | 0.00 | 0.00 | 0.00 |
| 18 | BPH | T65 | 20 | 30 | 2 | 5 | 0.00 | 0.00 | 0.00 |
| 18 | BPH | T65 | 20 | 30 | 3 | 5 | 0.00 | 0.00 | 0.00 |
| 18 | BPH | T65 | 20 | 30 | 4 | 5 | 0.00 | 0.00 | 0.00 |
| 18 | BPH | T65 | 20 | 35 | 1 | 5 | 0.00 | 0.00 | 0.00 |
| 18 | BPH | T65 | 20 | 35 | 2 | 3 | 0.00 | 0.00 | 0.00 |
| 18 | BPH | T65 | 20 | 35 | 3 | 3 | 0.00 | 0.00 | 0.00 |
| 18 | BPH | T65 | 20 | 35 | 4 | 3 | 0.00 | 0.00 | 0.00 |
| 18 | BPH | T65 | 30 | 15 | 1 | 3 | 0.00 | 0.00 | 0.00 |
| 18 | BPH | T65 | 30 | 15 | 2 | 3 | 0.00 | 0.00 | 0.00 |
| 18 | BPH | T65 | 30 | 15 | 3 | 3 | 0.00 | 0.00 | 0.00 |
| 18 | BPH | T65 | 30 | 15 | 4 | 3 | 0.00 | 0.00 | 0.00 |
| 18 | BPH | T65 | 30 | 20 | 1 | 5 | 0.00 | 0.00 | 0.00 |
| 18 | BPH | T65 | 30 | 20 | 2 | 5 | 2.00 | 7.00 | 40.00 |
| 18 | BPH | T65 | 30 | 20 | 3 | 5 | 0.00 | 0.00 | 0.00 |
| 18 | BPH | T65 | 30 | 20 | 4 | 5 | 0.00 | 0.00 | 0.00 |
| 18 | BPH | T65 | 30 | 25 | 1 | 5 | 0.00 | 0.00 | 0.00 |
| 18 | BPH | T65 | 30 | 25 | 2 | 5 | 1.00 | 3.00 | 40.00 |
| 18 | BPH | T65 | 30 | 25 | 3 | 5 | 0.00 | 0.00 | 0.00 |
| 18 | BPH | T65 | 30 | 25 | 4 | 5 | 0.00 | 0.00 | 0.00 |
| 18 | BPH | T65 | 30 | 30 | 1 | 5 | 0.00 | 0.00 | 0.00 |
| 18 | BPH | T65 | 30 | 30 | 2 | 5 | 1.00 | 0.00 | 20.00 |
| 18 | BPH | T65 | 30 | 30 | 3 | 5 | 0.00 | 0.00 | 0.00 |
| 18 | BPH | T65 | 30 | 30 | 4 | 5 | 0.00 | 0.00 | 0.00 |
| 18 | BPH | T65 | 30 | 35 | 1 | 5 | 0.00 | 0.00 | 0.00 |
| 18 | BPH | T65 | 30 | 35 | 2 | 3 | 0.00 | 0.00 | 0.00 |
| 18 | BPH | T65 | 30 | 35 | 3 | 3 | 0.00 | 0.00 | 0.00 |
| 18 | BPH | T65 | 30 | 35 | 4 | 3 | 0.00 | 0.00 | 0.00 |
| 18 | WBPH | IR22 | 20 | 15 | 1 | 3 | 0.33 | 0.00 | 11.11 |
| 18 | WBPH | IR22 | 20 | 15 | 2 | 3 | 0.00 | 0.00 | 0.00 |
| 18 | WBPH | IR22 | 20 | 15 | 3 | 3 | 0.00 | 0.00 | 0.00 |
| 18 | WBPH | IR22 | 20 | 15 | 4 | 3 | 1.00 | 0.00 | 33.33 |
| 18 | WBPH | IR22 | 20 | 20 | 1 | 5 | 0.00 | 0.00 | 0.00 |
| 18 | WBPH | IR22 | 20 | 20 | 2 | 5 | 0.00 | 0.00 | 0.00 |
| 18 | WBPH | IR22 | 20 | 20 | 3 | 3 | 0.00 | 0.00 | 0.00 |
| 18 | WBPH | IR22 | 20 | 20 | 4 | 3 | 0.00 | 0.00 | 0.00 |
| 18 | WBPH | IR22 | 20 | 25 | 1 | 5 | 0.00 | 0.00 | 0.00 |
| 18 | WBPH | IR22 | 20 | 25 | 2 | 5 | 0.00 | 0.00 | 0.00 |
| 18 | WBPH | IR22 | 20 | 25 | 3 | 5 | 0.00 | 0.00 | 0.00 |
| 18 | WBPH | IR22 | 20 | 25 | 4 | 5 | 0.00 | 0.00 | 0.00 |
| 18 | WBPH | IR22 | 20 | 30 | 1 | 5 | 0.00 | 0.00 | 0.00 |
| 18 | WBPH | IR22 | 20 | 30 | 2 | 5 | 0.00 | 0.00 | 0.00 |
| 18 | WBPH | IR22 | 20 | 30 | 3 | 5 | 0.00 | 0.00 | 0.00 |
| 18 | WBPH | IR22 | 20 | 30 | 4 | 5 | 0.00 | 0.00 | 0.00 |
| 18 | WBPH | IR22 | 20 | 35 | 1 | 5 | 0.00 | 0.00 | 0.00 |
| 18 | WBPH | IR22 | 20 | 35 | 2 | 3 | 0.00 | 0.00 | 0.00 |
| 18 | WBPH | IR22 | 20 | 35 | 3 | 3 | 0.00 | 0.00 | 0.00 |
| 18 | WBPH | IR22 | 20 | 35 | 4 | 3 | 0.00 | 0.00 | 0.00 |
| 18 | WBPH | IR22 | 30 | 15 | 1 | 1 | 0.00 | 0.00 | 0.00 |
| 18 | WBPH | IR22 | 30 | 15 | 2 | 1 | 0.00 | 0.00 | 67.00 |
| 18 | WBPH | IR22 | 30 | 15 | 3 | 1 | 0.00 | 0.00 | 0.00 |
| 18 | WBPH | IR22 | 30 | 15 | 4 | 1 | 0.00 | 0.00 | 0.00 |
| 18 | WBPH | IR22 | 30 | 20 | 1 | 5 | 0.00 | 0.00 | 0.00 |
| 18 | WBPH | IR22 | 30 | 20 | 2 | 5 | 0.00 | 0.00 | 0.00 |
| 18 | WBPH | IR22 | 30 | 20 | 3 | 5 | 0.00 | 0.00 | 0.00 |
| 18 | WBPH | IR22 | 30 | 20 | 4 | 5 | 0.00 | 0.00 | 0.00 |
| 18 | WBPH | IR22 | 30 | 25 | 1 | 5 | 0.00 | 0.00 | 0.00 |
| 18 | WBPH | IR22 | 30 | 25 | 2 | 5 | 0.00 | 0.00 | 0.00 |
| 18 | WBPH | IR22 | 30 | 25 | 3 | 5 | 0.00 | 0.00 | 0.00 |
| 18 | WBPH | IR22 | 30 | 25 | 4 | 5 | 0.00 | 0.00 | 0.00 |
| 18 | WBPH | IR22 | 30 | 30 | 1 | 5 | 0.00 | 0.00 | 0.00 |
| 18 | WBPH | IR22 | 30 | 30 | 2 | 5 | 0.00 | 0.00 | 0.00 |
| 18 | WBPH | IR22 | 30 | 30 | 3 | 5 | 0.00 | 0.00 | 0.00 |
| 18 | WBPH | IR22 | 30 | 30 | 4 | 5 | 0.00 | 0.00 | 0.00 |
| 18 | WBPH | IR22 | 30 | 35 | 1 | 5 | 0.00 | 0.00 | 0.00 |
| 18 | WBPH | IR22 | 30 | 35 | 2 | 3 | 0.00 | 0.00 | 0.00 |
| 18 | WBPH | IR22 | 30 | 35 | 3 | 3 | 0.00 | 0.00 | 0.00 |
| 18 | WBPH | IR22 | 30 | 35 | 4 | 3 | 0.00 | 0.00 | 0.00 |
| 18 | WBPH | T65 | 20 | 15 | 1 | 3 | 0.67 | 0.00 | 22.22 |
| 18 | WBPH | T65 | 20 | 15 | 2 | 3 | 0.00 | 0.00 | 0.00 |
| 18 | WBPH | T65 | 20 | 15 | 3 | 3 | 1.00 | 0.00 | 33.33 |
| 18 | WBPH | T65 | 20 | 15 | 4 | 3 | 1.00 | 0.00 | 33.33 |
| 18 | WBPH | T65 | 20 | 20 | 1 | 5 | 0.67 | 2.67 | 11.11 |
| 18 | WBPH | T65 | 20 | 20 | 2 | 5 | 0.00 | 0.00 | 0.00 |
| 18 | WBPH | T65 | 20 | 20 | 3 | 3 | 0.00 | 0.00 | 0.00 |
| 18 | WBPH | T65 | 20 | 20 | 4 | 3 | 2.00 | 8.00 | 33.33 |
| 18 | WBPH | T65 | 20 | 25 | 1 | 5 | 0.00 | 0.00 | 0.00 |
| 18 | WBPH | T65 | 20 | 25 | 2 | 5 | 0.00 | 0.00 | 0.00 |
| 18 | WBPH | T65 | 20 | 25 | 3 | 5 | 0.00 | 0.00 | 0.00 |
| 18 | WBPH | T65 | 20 | 25 | 4 | 5 | 0.00 | 0.00 | 0.00 |
| 18 | WBPH | T65 | 20 | 30 | 1 | 5 | 0.00 | 0.00 | 0.00 |
| 18 | WBPH | T65 | 20 | 30 | 2 | 5 | 0.00 | 0.00 | 0.00 |
| 18 | WBPH | T65 | 20 | 30 | 3 | 5 | 0.00 | 0.00 | 0.00 |
| 18 | WBPH | T65 | 20 | 30 | 4 | 5 | 0.00 | 0.00 | 0.00 |
| 18 | WBPH | T65 | 20 | 35 | 1 | 5 | 0.00 | 0.00 | 0.00 |
| 18 | WBPH | T65 | 20 | 35 | 2 | 3 | 0.00 | 0.00 | 0.00 |
| 18 | WBPH | T65 | 20 | 35 | 3 | 3 | 0.00 | 0.00 | 0.00 |
| 18 | WBPH | T65 | 20 | 35 | 4 | 3 | 0.00 | 0.00 | 0.00 |
| 18 | WBPH | T65 | 30 | 15 | 1 | 1 | 0.00 | 0.00 | 0.00 |
| 18 | WBPH | T65 | 30 | 15 | 2 | 1 | 0.00 | 0.00 | 0.00 |
| 18 | WBPH | T65 | 30 | 15 | 3 | 1 | 0.00 | 0.00 | 80.00 |
| 18 | WBPH | T65 | 30 | 15 | 4 | 1 | 0.00 | 0.00 | 33.00 |
| 18 | WBPH | T65 | 30 | 20 | 1 | 5 | 0.00 | 0.00 | 0.00 |
| 18 | WBPH | T65 | 30 | 20 | 2 | 5 | 0.00 | 0.00 | 0.00 |
| 18 | WBPH | T65 | 30 | 20 | 3 | 5 | 0.00 | 0.00 | 0.00 |
| 18 | WBPH | T65 | 30 | 20 | 4 | 5 | 0.00 | 0.00 | 0.00 |
| 18 | WBPH | T65 | 30 | 25 | 1 | 5 | 0.00 | 0.00 | 0.00 |
| 18 | WBPH | T65 | 30 | 25 | 2 | 5 | 0.00 | 0.00 | 0.00 |
| 18 | WBPH | T65 | 30 | 25 | 3 | 5 | 0.00 | 0.00 | 0.00 |
| 18 | WBPH | T65 | 30 | 25 | 4 | 5 | 0.00 | 0.00 | 0.00 |
| 18 | WBPH | T65 | 30 | 30 | 1 | 5 | 0.00 | 0.00 | 0.00 |
| 18 | WBPH | T65 | 30 | 30 | 2 | 5 | 0.00 | 0.00 | 0.00 |
| 18 | WBPH | T65 | 30 | 30 | 3 | 5 | 0.00 | 0.00 | 0.00 |
| 18 | WBPH | T65 | 30 | 30 | 4 | 5 | 0.00 | 0.00 | 0.00 |
| 18 | WBPH | T65 | 30 | 35 | 1 | 5 | 0.00 | 0.00 | 0.00 |
| 18 | WBPH | T65 | 30 | 35 | 2 | 3 | 0.00 | 0.00 | 0.00 |
| 18 | WBPH | T65 | 30 | 35 | 3 | 3 | 0.00 | 0.00 | 0.00 |
| 18 | WBPH | T65 | 30 | 35 | 4 | 3 | 0.00 | 0.00 | 0.00 |
| 19 | BPH | IR22 | 20 | 15 | 1 | 3 | 0.00 | 0.00 | 0.00 |
| 19 | BPH | IR22 | 20 | 15 | 2 | 3 | 0.00 | 0.00 | 0.00 |
| 19 | BPH | IR22 | 20 | 15 | 3 | 3 | 0.00 | 0.00 | 0.00 |
| 19 | BPH | IR22 | 20 | 15 | 4 | 3 | 0.00 | 0.00 | 0.00 |
| 19 | BPH | IR22 | 20 | 20 | 1 | 5 | 0.33 | 0.00 | 17.78 |
| 19 | BPH | IR22 | 20 | 20 | 2 | 5 | 0.00 | 0.00 | 20.00 |
| 19 | BPH | IR22 | 20 | 20 | 3 | 3 | 1.00 | 0.00 | 33.33 |
| 19 | BPH | IR22 | 20 | 20 | 4 | 3 | 0.00 | 0.00 | 0.00 |
| 19 | BPH | IR22 | 20 | 25 | 1 | 5 | 0.00 | 0.00 | 0.00 |
| 19 | BPH | IR22 | 20 | 25 | 2 | 5 | 0.00 | 0.00 | 20.00 |
| 19 | BPH | IR22 | 20 | 25 | 3 | 5 | 0.00 | 0.00 | 0.00 |
| 19 | BPH | IR22 | 20 | 25 | 4 | 5 | 0.00 | 0.00 | 0.00 |
| 19 | BPH | IR22 | 20 | 30 | 1 | 5 | 0.00 | 0.00 | 0.00 |
| 19 | BPH | IR22 | 20 | 30 | 2 | 5 | 0.00 | 0.00 | 0.00 |
| 19 | BPH | IR22 | 20 | 30 | 3 | 5 | 0.00 | 0.00 | 0.00 |
| 19 | BPH | IR22 | 20 | 30 | 4 | 5 | 0.00 | 0.00 | 0.00 |
| 19 | BPH | IR22 | 20 | 35 | 1 | 5 | 0.00 | 0.00 | 0.00 |
| 19 | BPH | IR22 | 20 | 35 | 2 | 3 | 0.00 | 0.00 | 0.00 |
| 19 | BPH | IR22 | 20 | 35 | 3 | 3 | 0.00 | 0.00 | 0.00 |
| 19 | BPH | IR22 | 20 | 35 | 4 | 3 | 0.00 | 0.00 | 0.00 |
| 19 | BPH | IR22 | 30 | 15 | 1 | 3 | 0.00 | 0.00 | 0.00 |
| 19 | BPH | IR22 | 30 | 15 | 2 | 3 | 0.00 | 0.00 | 0.00 |
| 19 | BPH | IR22 | 30 | 15 | 3 | 3 | 1.00 | 0.00 | 66.67 |
| 19 | BPH | IR22 | 30 | 15 | 4 | 3 | 0.00 | 0.00 | 0.00 |
| 19 | BPH | IR22 | 30 | 20 | 1 | 5 | 0.00 | 0.00 | 0.00 |
| 19 | BPH | IR22 | 30 | 20 | 2 | 5 | 2.33 | 6.00 | 60.00 |
| 19 | BPH | IR22 | 30 | 20 | 3 | 5 | 0.00 | 0.00 | 0.00 |
| 19 | BPH | IR22 | 30 | 20 | 4 | 5 | 0.00 | 0.00 | 0.00 |
| 19 | BPH | IR22 | 30 | 25 | 1 | 5 | 0.00 | 0.00 | 0.00 |
| 19 | BPH | IR22 | 30 | 25 | 2 | 5 | 1.00 | 3.33 | 60.00 |
| 19 | BPH | IR22 | 30 | 25 | 3 | 5 | 0.00 | 0.00 | 0.00 |
| 19 | BPH | IR22 | 30 | 25 | 4 | 5 | 0.00 | 0.00 | 0.00 |
| 19 | BPH | IR22 | 30 | 30 | 1 | 5 | 0.00 | 0.00 | 0.00 |
| 19 | BPH | IR22 | 30 | 30 | 2 | 5 | 0.00 | 0.00 | 0.00 |
| 19 | BPH | IR22 | 30 | 30 | 3 | 5 | 0.00 | 0.00 | 0.00 |
| 19 | BPH | IR22 | 30 | 30 | 4 | 5 | 0.00 | 0.00 | 0.00 |
| 19 | BPH | IR22 | 30 | 35 | 1 | 5 | 0.00 | 0.00 | 0.00 |
| 19 | BPH | IR22 | 30 | 35 | 2 | 3 | 0.00 | 0.00 | 0.00 |
| 19 | BPH | IR22 | 30 | 35 | 3 | 3 | 0.00 | 0.00 | 0.00 |
| 19 | BPH | IR22 | 30 | 35 | 4 | 3 | 0.00 | 0.00 | 0.00 |
| 19 | BPH | T65 | 20 | 15 | 1 | 3 | 0.00 | 0.00 | 0.00 |
| 19 | BPH | T65 | 20 | 15 | 2 | 3 | 0.00 | 0.00 | 0.00 |
| 19 | BPH | T65 | 20 | 15 | 3 | 3 | 0.00 | 0.00 | 0.00 |
| 19 | BPH | T65 | 20 | 15 | 4 | 3 | 0.00 | 0.00 | 0.00 |
| 19 | BPH | T65 | 20 | 20 | 1 | 5 | 0.00 | 0.00 | 0.00 |
| 19 | BPH | T65 | 20 | 20 | 2 | 5 | 0.00 | 0.00 | 20.00 |
| 19 | BPH | T65 | 20 | 20 | 3 | 3 | 0.00 | 0.00 | 0.00 |
| 19 | BPH | T65 | 20 | 20 | 4 | 3 | 0.00 | 0.00 | 0.00 |
| 19 | BPH | T65 | 20 | 25 | 1 | 5 | 0.00 | 0.00 | 0.00 |
| 19 | BPH | T65 | 20 | 25 | 2 | 5 | 0.00 | 0.00 | 0.00 |
| 19 | BPH | T65 | 20 | 25 | 3 | 5 | 0.00 | 0.00 | 0.00 |
| 19 | BPH | T65 | 20 | 25 | 4 | 5 | 0.00 | 0.00 | 0.00 |
| 19 | BPH | T65 | 20 | 30 | 1 | 5 | 0.00 | 0.00 | 0.00 |
| 19 | BPH | T65 | 20 | 30 | 2 | 5 | 0.00 | 0.00 | 0.00 |
| 19 | BPH | T65 | 20 | 30 | 3 | 5 | 0.00 | 0.00 | 0.00 |
| 19 | BPH | T65 | 20 | 30 | 4 | 5 | 0.00 | 0.00 | 0.00 |
| 19 | BPH | T65 | 20 | 35 | 1 | 5 | 0.00 | 0.00 | 0.00 |
| 19 | BPH | T65 | 20 | 35 | 2 | 3 | 0.00 | 0.00 | 0.00 |
| 19 | BPH | T65 | 20 | 35 | 3 | 3 | 0.00 | 0.00 | 0.00 |
| 19 | BPH | T65 | 20 | 35 | 4 | 3 | 0.00 | 0.00 | 0.00 |
| 19 | BPH | T65 | 30 | 15 | 1 | 3 | 0.00 | 0.00 | 0.00 |
| 19 | BPH | T65 | 30 | 15 | 2 | 3 | 0.00 | 0.00 | 0.00 |
| 19 | BPH | T65 | 30 | 15 | 3 | 3 | 0.00 | 0.00 | 0.00 |
| 19 | BPH | T65 | 30 | 15 | 4 | 3 | 0.00 | 0.00 | 0.00 |
| 19 | BPH | T65 | 30 | 20 | 1 | 5 | 0.00 | 0.00 | 0.00 |
| 19 | BPH | T65 | 30 | 20 | 2 | 5 | 1.00 | 2.50 | 40.00 |
| 19 | BPH | T65 | 30 | 20 | 3 | 5 | 0.00 | 0.00 | 0.00 |
| 19 | BPH | T65 | 30 | 20 | 4 | 5 | 0.00 | 0.00 | 0.00 |
| 19 | BPH | T65 | 30 | 25 | 1 | 5 | 0.00 | 0.00 | 0.00 |
| 19 | BPH | T65 | 30 | 25 | 2 | 5 | 1.00 | 0.00 | 20.00 |
| 19 | BPH | T65 | 30 | 25 | 3 | 5 | 0.00 | 0.00 | 0.00 |
| 19 | BPH | T65 | 30 | 25 | 4 | 5 | 0.00 | 0.00 | 0.00 |
| 19 | BPH | T65 | 30 | 30 | 1 | 5 | 0.00 | 0.00 | 0.00 |
| 19 | BPH | T65 | 30 | 30 | 2 | 5 | 0.00 | 0.00 | 0.00 |
| 19 | BPH | T65 | 30 | 30 | 3 | 5 | 0.00 | 0.00 | 0.00 |
| 19 | BPH | T65 | 30 | 30 | 4 | 5 | 0.00 | 0.00 | 0.00 |
| 19 | BPH | T65 | 30 | 35 | 1 | 5 | 0.00 | 0.00 | 0.00 |
| 19 | BPH | T65 | 30 | 35 | 2 | 3 | 0.00 | 0.00 | 0.00 |
| 19 | BPH | T65 | 30 | 35 | 3 | 3 | 0.00 | 0.00 | 0.00 |
| 19 | BPH | T65 | 30 | 35 | 4 | 3 | 0.00 | 0.00 | 0.00 |
| 19 | WBPH | IR22 | 20 | 15 | 1 | 3 | 0.33 | 0.00 | 11.11 |
| 19 | WBPH | IR22 | 20 | 15 | 2 | 3 | 0.00 | 0.00 | 0.00 |
| 19 | WBPH | IR22 | 20 | 15 | 3 | 3 | 0.00 | 0.00 | 0.00 |
| 19 | WBPH | IR22 | 20 | 15 | 4 | 3 | 1.00 | 0.00 | 33.33 |
| 19 | WBPH | IR22 | 20 | 20 | 1 | 5 | 0.00 | 0.00 | 0.00 |
| 19 | WBPH | IR22 | 20 | 20 | 2 | 5 | 0.00 | 0.00 | 0.00 |
| 19 | WBPH | IR22 | 20 | 20 | 3 | 3 | 0.00 | 0.00 | 0.00 |
| 19 | WBPH | IR22 | 20 | 20 | 4 | 3 | 0.00 | 0.00 | 0.00 |
| 19 | WBPH | IR22 | 20 | 25 | 1 | 5 | 0.00 | 0.00 | 0.00 |
| 19 | WBPH | IR22 | 20 | 25 | 2 | 5 | 0.00 | 0.00 | 0.00 |
| 19 | WBPH | IR22 | 20 | 25 | 3 | 5 | 0.00 | 0.00 | 0.00 |
| 19 | WBPH | IR22 | 20 | 25 | 4 | 5 | 0.00 | 0.00 | 0.00 |
| 19 | WBPH | IR22 | 20 | 30 | 1 | 5 | 0.00 | 0.00 | 0.00 |
| 19 | WBPH | IR22 | 20 | 30 | 2 | 5 | 0.00 | 0.00 | 0.00 |
| 19 | WBPH | IR22 | 20 | 30 | 3 | 5 | 0.00 | 0.00 | 0.00 |
| 19 | WBPH | IR22 | 20 | 30 | 4 | 5 | 0.00 | 0.00 | 0.00 |
| 19 | WBPH | IR22 | 20 | 35 | 1 | 5 | 0.00 | 0.00 | 0.00 |
| 19 | WBPH | IR22 | 20 | 35 | 2 | 3 | 0.00 | 0.00 | 0.00 |
| 19 | WBPH | IR22 | 20 | 35 | 3 | 3 | 0.00 | 0.00 | 0.00 |
| 19 | WBPH | IR22 | 20 | 35 | 4 | 3 | 0.00 | 0.00 | 0.00 |
| 19 | WBPH | IR22 | 30 | 15 | 1 | 1 | 0.00 | 0.00 | 0.00 |
| 19 | WBPH | IR22 | 30 | 15 | 2 | 1 | 0.00 | 0.00 | 67.00 |
| 19 | WBPH | IR22 | 30 | 15 | 3 | 1 | 0.00 | 0.00 | 0.00 |
| 19 | WBPH | IR22 | 30 | 15 | 4 | 1 | 0.00 | 0.00 | 0.00 |
| 19 | WBPH | IR22 | 30 | 20 | 1 | 5 | 0.00 | 0.00 | 0.00 |
| 19 | WBPH | IR22 | 30 | 20 | 2 | 5 | 0.00 | 0.00 | 0.00 |
| 19 | WBPH | IR22 | 30 | 20 | 3 | 5 | 0.00 | 0.00 | 0.00 |
| 19 | WBPH | IR22 | 30 | 20 | 4 | 5 | 0.00 | 0.00 | 0.00 |
| 19 | WBPH | IR22 | 30 | 25 | 1 | 5 | 0.00 | 0.00 | 0.00 |
| 19 | WBPH | IR22 | 30 | 25 | 2 | 5 | 0.00 | 0.00 | 0.00 |
| 19 | WBPH | IR22 | 30 | 25 | 3 | 5 | 0.00 | 0.00 | 0.00 |
| 19 | WBPH | IR22 | 30 | 25 | 4 | 5 | 0.00 | 0.00 | 0.00 |
| 19 | WBPH | IR22 | 30 | 30 | 1 | 5 | 0.00 | 0.00 | 0.00 |
| 19 | WBPH | IR22 | 30 | 30 | 2 | 5 | 0.00 | 0.00 | 0.00 |
| 19 | WBPH | IR22 | 30 | 30 | 3 | 5 | 0.00 | 0.00 | 0.00 |
| 19 | WBPH | IR22 | 30 | 30 | 4 | 5 | 0.00 | 0.00 | 0.00 |
| 19 | WBPH | IR22 | 30 | 35 | 1 | 5 | 0.00 | 0.00 | 0.00 |
| 19 | WBPH | IR22 | 30 | 35 | 2 | 3 | 0.00 | 0.00 | 0.00 |
| 19 | WBPH | IR22 | 30 | 35 | 3 | 3 | 0.00 | 0.00 | 0.00 |
| 19 | WBPH | IR22 | 30 | 35 | 4 | 3 | 0.00 | 0.00 | 0.00 |
| 19 | WBPH | T65 | 20 | 15 | 1 | 3 | 0.67 | 0.00 | 22.22 |
| 19 | WBPH | T65 | 20 | 15 | 2 | 3 | 0.00 | 0.00 | 0.00 |
| 19 | WBPH | T65 | 20 | 15 | 3 | 3 | 1.00 | 0.00 | 33.33 |
| 19 | WBPH | T65 | 20 | 15 | 4 | 3 | 1.00 | 0.00 | 33.33 |
| 19 | WBPH | T65 | 20 | 20 | 1 | 5 | 0.33 | 0.00 | 11.11 |
| 19 | WBPH | T65 | 20 | 20 | 2 | 5 | 0.00 | 0.00 | 0.00 |
| 19 | WBPH | T65 | 20 | 20 | 3 | 3 | 0.00 | 0.00 | 0.00 |
| 19 | WBPH | T65 | 20 | 20 | 4 | 3 | 1.00 | 0.00 | 33.33 |
| 19 | WBPH | T65 | 20 | 25 | 1 | 5 | 0.00 | 0.00 | 0.00 |
| 19 | WBPH | T65 | 20 | 25 | 2 | 5 | 0.00 | 0.00 | 0.00 |
| 19 | WBPH | T65 | 20 | 25 | 3 | 5 | 0.00 | 0.00 | 0.00 |
| 19 | WBPH | T65 | 20 | 25 | 4 | 5 | 0.00 | 0.00 | 0.00 |
| 19 | WBPH | T65 | 20 | 30 | 1 | 5 | 0.00 | 0.00 | 0.00 |
| 19 | WBPH | T65 | 20 | 30 | 2 | 5 | 0.00 | 0.00 | 0.00 |
| 19 | WBPH | T65 | 20 | 30 | 3 | 5 | 0.00 | 0.00 | 0.00 |
| 19 | WBPH | T65 | 20 | 30 | 4 | 5 | 0.00 | 0.00 | 0.00 |
| 19 | WBPH | T65 | 20 | 35 | 1 | 5 | 0.00 | 0.00 | 0.00 |
| 19 | WBPH | T65 | 20 | 35 | 2 | 3 | 0.00 | 0.00 | 0.00 |
| 19 | WBPH | T65 | 20 | 35 | 3 | 3 | 0.00 | 0.00 | 0.00 |
| 19 | WBPH | T65 | 20 | 35 | 4 | 3 | 0.00 | 0.00 | 0.00 |
| 19 | WBPH | T65 | 30 | 15 | 1 | 1 | 0.00 | 0.00 | 0.00 |
| 19 | WBPH | T65 | 30 | 15 | 2 | 1 | 0.00 | 0.00 | 0.00 |
| 19 | WBPH | T65 | 30 | 15 | 3 | 1 | 0.00 | 0.00 | 80.00 |
| 19 | WBPH | T65 | 30 | 15 | 4 | 1 | 0.00 | 0.00 | 33.00 |
| 19 | WBPH | T65 | 30 | 20 | 1 | 5 | 0.00 | 0.00 | 0.00 |
| 19 | WBPH | T65 | 30 | 20 | 2 | 5 | 0.00 | 0.00 | 0.00 |
| 19 | WBPH | T65 | 30 | 20 | 3 | 5 | 0.00 | 0.00 | 0.00 |
| 19 | WBPH | T65 | 30 | 20 | 4 | 5 | 0.00 | 0.00 | 0.00 |
| 19 | WBPH | T65 | 30 | 25 | 1 | 5 | 0.00 | 0.00 | 0.00 |
| 19 | WBPH | T65 | 30 | 25 | 2 | 5 | 0.00 | 0.00 | 0.00 |
| 19 | WBPH | T65 | 30 | 25 | 3 | 5 | 0.00 | 0.00 | 0.00 |
| 19 | WBPH | T65 | 30 | 25 | 4 | 5 | 0.00 | 0.00 | 0.00 |
| 19 | WBPH | T65 | 30 | 30 | 1 | 5 | 0.00 | 0.00 | 0.00 |
| 19 | WBPH | T65 | 30 | 30 | 2 | 5 | 0.00 | 0.00 | 0.00 |
| 19 | WBPH | T65 | 30 | 30 | 3 | 5 | 0.00 | 0.00 | 0.00 |
| 19 | WBPH | T65 | 30 | 30 | 4 | 5 | 0.00 | 0.00 | 0.00 |
| 19 | WBPH | T65 | 30 | 35 | 1 | 5 | 0.00 | 0.00 | 0.00 |
| 19 | WBPH | T65 | 30 | 35 | 2 | 3 | 0.00 | 0.00 | 0.00 |
| 19 | WBPH | T65 | 30 | 35 | 3 | 3 | 0.00 | 0.00 | 0.00 |
| 19 | WBPH | T65 | 30 | 35 | 4 | 3 | 0.00 | 0.00 | 0.00 |
| 20 | BPH | IR22 | 20 | 15 | 1 | 3 | 0.00 | 0.00 | 0.00 |
| 20 | BPH | IR22 | 20 | 15 | 2 | 3 | 0.00 | 0.00 | 0.00 |
| 20 | BPH | IR22 | 20 | 15 | 3 | 3 | 0.00 | 0.00 | 0.00 |
| 20 | BPH | IR22 | 20 | 15 | 4 | 3 | 0.00 | 0.00 | 0.00 |
| 20 | BPH | IR22 | 20 | 20 | 1 | 5 | 0.00 | 0.00 | 6.67 |
| 20 | BPH | IR22 | 20 | 20 | 2 | 5 | 0.00 | 0.00 | 20.00 |
| 20 | BPH | IR22 | 20 | 20 | 3 | 3 | 0.00 | 0.00 | 0.00 |
| 20 | BPH | IR22 | 20 | 20 | 4 | 3 | 0.00 | 0.00 | 0.00 |
| 20 | BPH | IR22 | 20 | 25 | 1 | 5 | 0.00 | 0.00 | 0.00 |
| 20 | BPH | IR22 | 20 | 25 | 2 | 5 | 0.00 | 0.00 | 20.00 |
| 20 | BPH | IR22 | 20 | 25 | 3 | 5 | 0.00 | 0.00 | 0.00 |
| 20 | BPH | IR22 | 20 | 25 | 4 | 5 | 0.00 | 0.00 | 0.00 |
| 20 | BPH | IR22 | 20 | 30 | 1 | 5 | 0.00 | 0.00 | 0.00 |
| 20 | BPH | IR22 | 20 | 30 | 2 | 5 | 0.00 | 0.00 | 0.00 |
| 20 | BPH | IR22 | 20 | 30 | 3 | 5 | 0.00 | 0.00 | 0.00 |
| 20 | BPH | IR22 | 20 | 30 | 4 | 5 | 0.00 | 0.00 | 0.00 |
| 20 | BPH | IR22 | 20 | 35 | 1 | 5 | 0.00 | 0.00 | 0.00 |
| 20 | BPH | IR22 | 20 | 35 | 2 | 3 | 0.00 | 0.00 | 0.00 |
| 20 | BPH | IR22 | 20 | 35 | 3 | 3 | 0.00 | 0.00 | 0.00 |
| 20 | BPH | IR22 | 20 | 35 | 4 | 3 | 0.00 | 0.00 | 0.00 |
| 20 | BPH | IR22 | 30 | 15 | 1 | 3 | 0.00 | 0.00 | 0.00 |
| 20 | BPH | IR22 | 30 | 15 | 2 | 3 | 0.00 | 0.00 | 0.00 |
| 20 | BPH | IR22 | 30 | 15 | 3 | 3 | 1.00 | 0.00 | 66.67 |
| 20 | BPH | IR22 | 30 | 15 | 4 | 3 | 0.00 | 0.00 | 0.00 |
| 20 | BPH | IR22 | 30 | 20 | 1 | 5 | 0.00 | 0.00 | 0.00 |
| 20 | BPH | IR22 | 30 | 20 | 2 | 5 | 1.00 | 0.00 | 60.00 |
| 20 | BPH | IR22 | 30 | 20 | 3 | 5 | 0.00 | 0.00 | 0.00 |
| 20 | BPH | IR22 | 30 | 20 | 4 | 5 | 0.00 | 0.00 | 0.00 |
| 20 | BPH | IR22 | 30 | 25 | 1 | 5 | 0.00 | 0.00 | 0.00 |
| 20 | BPH | IR22 | 30 | 25 | 2 | 5 | 1.00 | 0.00 | 60.00 |
| 20 | BPH | IR22 | 30 | 25 | 3 | 5 | 0.00 | 0.00 | 0.00 |
| 20 | BPH | IR22 | 30 | 25 | 4 | 5 | 0.00 | 0.00 | 0.00 |
| 20 | BPH | IR22 | 30 | 30 | 1 | 5 | 0.00 | 0.00 | 0.00 |
| 20 | BPH | IR22 | 30 | 30 | 2 | 5 | 0.00 | 0.00 | 0.00 |
| 20 | BPH | IR22 | 30 | 30 | 3 | 5 | 0.00 | 0.00 | 0.00 |
| 20 | BPH | IR22 | 30 | 30 | 4 | 5 | 0.00 | 0.00 | 0.00 |
| 20 | BPH | IR22 | 30 | 35 | 1 | 5 | 0.00 | 0.00 | 0.00 |
| 20 | BPH | IR22 | 30 | 35 | 2 | 3 | 0.00 | 0.00 | 0.00 |
| 20 | BPH | IR22 | 30 | 35 | 3 | 3 | 0.00 | 0.00 | 0.00 |
| 20 | BPH | IR22 | 30 | 35 | 4 | 3 | 0.00 | 0.00 | 0.00 |
| 20 | BPH | T65 | 20 | 15 | 1 | 3 | 0.00 | 0.00 | 0.00 |
| 20 | BPH | T65 | 20 | 15 | 2 | 3 | 0.00 | 0.00 | 0.00 |
| 20 | BPH | T65 | 20 | 15 | 3 | 3 | 0.00 | 0.00 | 0.00 |
| 20 | BPH | T65 | 20 | 15 | 4 | 3 | 0.00 | 0.00 | 0.00 |
| 20 | BPH | T65 | 20 | 20 | 1 | 5 | 0.00 | 0.00 | 0.00 |
| 20 | BPH | T65 | 20 | 20 | 2 | 5 | 0.00 | 0.00 | 20.00 |
| 20 | BPH | T65 | 20 | 20 | 3 | 3 | 0.00 | 0.00 | 0.00 |
| 20 | BPH | T65 | 20 | 20 | 4 | 3 | 0.00 | 0.00 | 0.00 |
| 20 | BPH | T65 | 20 | 25 | 1 | 5 | 0.00 | 0.00 | 0.00 |
| 20 | BPH | T65 | 20 | 25 | 2 | 5 | 0.00 | 0.00 | 0.00 |
| 20 | BPH | T65 | 20 | 25 | 3 | 5 | 0.00 | 0.00 | 0.00 |
| 20 | BPH | T65 | 20 | 25 | 4 | 5 | 0.00 | 0.00 | 0.00 |
| 20 | BPH | T65 | 20 | 30 | 1 | 5 | 0.00 | 0.00 | 0.00 |
| 20 | BPH | T65 | 20 | 30 | 2 | 5 | 0.00 | 0.00 | 0.00 |
| 20 | BPH | T65 | 20 | 30 | 3 | 5 | 0.00 | 0.00 | 0.00 |
| 20 | BPH | T65 | 20 | 30 | 4 | 5 | 0.00 | 0.00 | 0.00 |
| 20 | BPH | T65 | 20 | 35 | 1 | 5 | 0.00 | 0.00 | 0.00 |
| 20 | BPH | T65 | 20 | 35 | 2 | 3 | 0.00 | 0.00 | 0.00 |
| 20 | BPH | T65 | 20 | 35 | 3 | 3 | 0.00 | 0.00 | 0.00 |
| 20 | BPH | T65 | 20 | 35 | 4 | 3 | 0.00 | 0.00 | 0.00 |
| 20 | BPH | T65 | 30 | 15 | 1 | 3 | 0.00 | 0.00 | 0.00 |
| 20 | BPH | T65 | 30 | 15 | 2 | 3 | 0.00 | 0.00 | 0.00 |
| 20 | BPH | T65 | 30 | 15 | 3 | 3 | 0.00 | 0.00 | 0.00 |
| 20 | BPH | T65 | 30 | 15 | 4 | 3 | 0.00 | 0.00 | 0.00 |
| 20 | BPH | T65 | 30 | 20 | 1 | 5 | 0.00 | 0.00 | 0.00 |
| 20 | BPH | T65 | 30 | 20 | 2 | 5 | 1.00 | 0.00 | 40.00 |
| 20 | BPH | T65 | 30 | 20 | 3 | 5 | 0.00 | 0.00 | 0.00 |
| 20 | BPH | T65 | 30 | 20 | 4 | 5 | 0.00 | 0.00 | 0.00 |
| 20 | BPH | T65 | 30 | 25 | 1 | 5 | 0.00 | 0.00 | 0.00 |
| 20 | BPH | T65 | 30 | 25 | 2 | 5 | 1.00 | 4.00 | 20.00 |
| 20 | BPH | T65 | 30 | 25 | 3 | 5 | 0.00 | 0.00 | 0.00 |
| 20 | BPH | T65 | 30 | 25 | 4 | 5 | 0.00 | 0.00 | 0.00 |
| 20 | BPH | T65 | 30 | 30 | 1 | 5 | 0.00 | 0.00 | 0.00 |
| 20 | BPH | T65 | 30 | 30 | 2 | 5 | 0.00 | 0.00 | 0.00 |
| 20 | BPH | T65 | 30 | 30 | 3 | 5 | 0.00 | 0.00 | 0.00 |
| 20 | BPH | T65 | 30 | 30 | 4 | 5 | 0.00 | 0.00 | 0.00 |
| 20 | BPH | T65 | 30 | 35 | 1 | 5 | 0.00 | 0.00 | 0.00 |
| 20 | BPH | T65 | 30 | 35 | 2 | 3 | 0.00 | 0.00 | 0.00 |
| 20 | BPH | T65 | 30 | 35 | 3 | 3 | 0.00 | 0.00 | 0.00 |
| 20 | BPH | T65 | 30 | 35 | 4 | 3 | 0.00 | 0.00 | 0.00 |
| 20 | WBPH | IR22 | 20 | 15 | 1 | 3 | 0.33 | 0.00 | 11.11 |
| 20 | WBPH | IR22 | 20 | 15 | 2 | 3 | 0.00 | 0.00 | 0.00 |
| 20 | WBPH | IR22 | 20 | 15 | 3 | 3 | 0.00 | 0.00 | 0.00 |
| 20 | WBPH | IR22 | 20 | 15 | 4 | 3 | 1.00 | 0.00 | 33.33 |
| 20 | WBPH | IR22 | 20 | 20 | 1 | 5 | 0.00 | 0.00 | 0.00 |
| 20 | WBPH | IR22 | 20 | 20 | 2 | 5 | 0.00 | 0.00 | 0.00 |
| 20 | WBPH | IR22 | 20 | 20 | 3 | 3 | 0.00 | 0.00 | 0.00 |
| 20 | WBPH | IR22 | 20 | 20 | 4 | 3 | 0.00 | 0.00 | 0.00 |
| 20 | WBPH | IR22 | 20 | 25 | 1 | 5 | 0.00 | 0.00 | 0.00 |
| 20 | WBPH | IR22 | 20 | 25 | 2 | 5 | 0.00 | 0.00 | 0.00 |
| 20 | WBPH | IR22 | 20 | 25 | 3 | 5 | 0.00 | 0.00 | 0.00 |
| 20 | WBPH | IR22 | 20 | 25 | 4 | 5 | 0.00 | 0.00 | 0.00 |
| 20 | WBPH | IR22 | 20 | 30 | 1 | 5 | 0.00 | 0.00 | 0.00 |
| 20 | WBPH | IR22 | 20 | 30 | 2 | 5 | 0.00 | 0.00 | 0.00 |
| 20 | WBPH | IR22 | 20 | 30 | 3 | 5 | 0.00 | 0.00 | 0.00 |
| 20 | WBPH | IR22 | 20 | 30 | 4 | 5 | 0.00 | 0.00 | 0.00 |
| 20 | WBPH | IR22 | 20 | 35 | 1 | 5 | 0.00 | 0.00 | 0.00 |
| 20 | WBPH | IR22 | 20 | 35 | 2 | 3 | 0.00 | 0.00 | 0.00 |
| 20 | WBPH | IR22 | 20 | 35 | 3 | 3 | 0.00 | 0.00 | 0.00 |
| 20 | WBPH | IR22 | 20 | 35 | 4 | 3 | 0.00 | 0.00 | 0.00 |
| 20 | WBPH | IR22 | 30 | 15 | 1 | 1 | 0.00 | 0.00 | 0.00 |
| 20 | WBPH | IR22 | 30 | 15 | 2 | 1 | 0.00 | 0.00 | 33.00 |
| 20 | WBPH | IR22 | 30 | 15 | 3 | 1 | 0.00 | 0.00 | 0.00 |
| 20 | WBPH | IR22 | 30 | 15 | 4 | 1 | 0.00 | 0.00 | 0.00 |
| 20 | WBPH | IR22 | 30 | 20 | 1 | 5 | 0.00 | 0.00 | 0.00 |
| 20 | WBPH | IR22 | 30 | 20 | 2 | 5 | 0.00 | 0.00 | 0.00 |
| 20 | WBPH | IR22 | 30 | 20 | 3 | 5 | 0.00 | 0.00 | 0.00 |
| 20 | WBPH | IR22 | 30 | 20 | 4 | 5 | 0.00 | 0.00 | 0.00 |
| 20 | WBPH | IR22 | 30 | 25 | 1 | 5 | 0.00 | 0.00 | 0.00 |
| 20 | WBPH | IR22 | 30 | 25 | 2 | 5 | 0.00 | 0.00 | 0.00 |
| 20 | WBPH | IR22 | 30 | 25 | 3 | 5 | 0.00 | 0.00 | 0.00 |
| 20 | WBPH | IR22 | 30 | 25 | 4 | 5 | 0.00 | 0.00 | 0.00 |
| 20 | WBPH | IR22 | 30 | 30 | 1 | 5 | 0.00 | 0.00 | 0.00 |
| 20 | WBPH | IR22 | 30 | 30 | 2 | 5 | 0.00 | 0.00 | 0.00 |
| 20 | WBPH | IR22 | 30 | 30 | 3 | 5 | 0.00 | 0.00 | 0.00 |
| 20 | WBPH | IR22 | 30 | 30 | 4 | 5 | 0.00 | 0.00 | 0.00 |
| 20 | WBPH | IR22 | 30 | 35 | 1 | 5 | 0.00 | 0.00 | 0.00 |
| 20 | WBPH | IR22 | 30 | 35 | 2 | 3 | 0.00 | 0.00 | 0.00 |
| 20 | WBPH | IR22 | 30 | 35 | 3 | 3 | 0.00 | 0.00 | 0.00 |
| 20 | WBPH | IR22 | 30 | 35 | 4 | 3 | 0.00 | 0.00 | 0.00 |
| 20 | WBPH | T65 | 20 | 15 | 1 | 3 | 0.67 | 1.67 | 22.22 |
| 20 | WBPH | T65 | 20 | 15 | 2 | 3 | 0.00 | 0.00 | 0.00 |
| 20 | WBPH | T65 | 20 | 15 | 3 | 3 | 1.00 | 0.00 | 33.33 |
| 20 | WBPH | T65 | 20 | 15 | 4 | 3 | 1.00 | 5.00 | 33.33 |
| 20 | WBPH | T65 | 20 | 20 | 1 | 5 | 0.33 | 0.00 | 11.11 |
| 20 | WBPH | T65 | 20 | 20 | 2 | 5 | 0.00 | 0.00 | 0.00 |
| 20 | WBPH | T65 | 20 | 20 | 3 | 3 | 0.00 | 0.00 | 0.00 |
| 20 | WBPH | T65 | 20 | 20 | 4 | 3 | 1.00 | 0.00 | 33.33 |
| 20 | WBPH | T65 | 20 | 25 | 1 | 5 | 0.00 | 0.00 | 0.00 |
| 20 | WBPH | T65 | 20 | 25 | 2 | 5 | 0.00 | 0.00 | 0.00 |
| 20 | WBPH | T65 | 20 | 25 | 3 | 5 | 0.00 | 0.00 | 0.00 |
| 20 | WBPH | T65 | 20 | 25 | 4 | 5 | 0.00 | 0.00 | 0.00 |
| 20 | WBPH | T65 | 20 | 30 | 1 | 5 | 0.00 | 0.00 | 0.00 |
| 20 | WBPH | T65 | 20 | 30 | 2 | 5 | 0.00 | 0.00 | 0.00 |
| 20 | WBPH | T65 | 20 | 30 | 3 | 5 | 0.00 | 0.00 | 0.00 |
| 20 | WBPH | T65 | 20 | 30 | 4 | 5 | 0.00 | 0.00 | 0.00 |
| 20 | WBPH | T65 | 20 | 35 | 1 | 5 | 0.00 | 0.00 | 0.00 |
| 20 | WBPH | T65 | 20 | 35 | 2 | 3 | 0.00 | 0.00 | 0.00 |
| 20 | WBPH | T65 | 20 | 35 | 3 | 3 | 0.00 | 0.00 | 0.00 |
| 20 | WBPH | T65 | 20 | 35 | 4 | 3 | 0.00 | 0.00 | 0.00 |
| 20 | WBPH | T65 | 30 | 15 | 1 | 1 | 0.00 | 0.00 | 0.00 |
| 20 | WBPH | T65 | 30 | 15 | 2 | 1 | 0.00 | 0.00 | 0.00 |
| 20 | WBPH | T65 | 30 | 15 | 3 | 1 | 0.00 | 0.00 | 80.00 |
| 20 | WBPH | T65 | 30 | 15 | 4 | 1 | 0.00 | 0.00 | 33.00 |
| 20 | WBPH | T65 | 30 | 20 | 1 | 5 | 0.00 | 0.00 | 0.00 |
| 20 | WBPH | T65 | 30 | 20 | 2 | 5 | 0.00 | 0.00 | 0.00 |
| 20 | WBPH | T65 | 30 | 20 | 3 | 5 | 0.00 | 0.00 | 0.00 |
| 20 | WBPH | T65 | 30 | 20 | 4 | 5 | 0.00 | 0.00 | 0.00 |
| 20 | WBPH | T65 | 30 | 25 | 1 | 5 | 0.00 | 0.00 | 0.00 |
| 20 | WBPH | T65 | 30 | 25 | 2 | 5 | 0.00 | 0.00 | 0.00 |
| 20 | WBPH | T65 | 30 | 25 | 3 | 5 | 0.00 | 0.00 | 0.00 |
| 20 | WBPH | T65 | 30 | 25 | 4 | 5 | 0.00 | 0.00 | 0.00 |
| 20 | WBPH | T65 | 30 | 30 | 1 | 5 | 0.00 | 0.00 | 0.00 |
| 20 | WBPH | T65 | 30 | 30 | 2 | 5 | 0.00 | 0.00 | 0.00 |
| 20 | WBPH | T65 | 30 | 30 | 3 | 5 | 0.00 | 0.00 | 0.00 |
| 20 | WBPH | T65 | 30 | 30 | 4 | 5 | 0.00 | 0.00 | 0.00 |
| 20 | WBPH | T65 | 30 | 35 | 1 | 5 | 0.00 | 0.00 | 0.00 |
| 20 | WBPH | T65 | 30 | 35 | 2 | 3 | 0.00 | 0.00 | 0.00 |
| 20 | WBPH | T65 | 30 | 35 | 3 | 3 | 0.00 | 0.00 | 0.00 |
| 20 | WBPH | T65 | 30 | 35 | 4 | 3 | 0.00 | 0.00 | 0.00 |
